# Naturally occurring anti-cancer compounds: shining from Chinese herbal medicine

**DOI:** 10.1186/s13020-019-0270-9

**Published:** 2019-11-06

**Authors:** Hua Luo, Chi Teng Vong, Hanbin Chen, Yan Gao, Peng Lyu, Ling Qiu, Mingming Zhao, Qiao Liu, Zehua Cheng, Jian Zou, Peifen Yao, Caifang Gao, Jinchao Wei, Carolina Oi Lam Ung, Shengpeng Wang, Zhangfeng Zhong, Yitao Wang

**Affiliations:** Institute of Chinese Medical Sciences, State Key Laboratory of Quality Research in Chinese Medicine, University of Macau, Macao, China

**Keywords:** Cancer, Chinese herbal medicine, Natural products, Bioactive compounds, Traditional Chinese medicine

## Abstract

Numerous natural products originated from Chinese herbal medicine exhibit anti-cancer activities, including anti-proliferative, pro-apoptotic, anti-metastatic, anti-angiogenic effects, as well as regulate autophagy, reverse multidrug resistance, balance immunity, and enhance chemotherapy in vitro and in vivo. To provide new insights into the critical path ahead, we systemically reviewed the most recent advances (reported since 2011) on the key compounds with anti-cancer effects derived from Chinese herbal medicine (curcumin, epigallocatechin gallate, berberine, artemisinin, ginsenoside Rg3, ursolic acid, silibinin, emodin, triptolide, cucurbitacin B, tanshinone I, oridonin, shikonin, gambogic acid, artesunate, wogonin, β-elemene, and cepharanthine) in scientific databases (PubMed, Web of Science, Medline, Scopus, and Clinical Trials). With a broader perspective, we focused on their recently discovered and/or investigated pharmacological effects, novel mechanism of action, relevant clinical studies, and their innovative applications in combined therapy and immunomodulation. In addition, the present review has extended to describe other promising compounds including dihydroartemisinin, ginsenoside Rh2, compound K, cucurbitacins D, E, I, tanshinone IIA and cryptotanshinone in view of their potentials in cancer therapy. Up to now, the evidence about the immunomodulatory effects and clinical trials of natural anti-cancer compounds from Chinese herbal medicine is very limited, and further research is needed to monitor their immunoregulatory effects and explore their mechanisms of action as modulators of immune checkpoints.

## Background

Cancer is a leading public health problem worldwide with an estimated 18.1 million new cases and 9.6 million cancer deaths in 2018 [1]. Chinese herbal medicine has been used as anti-cancer agents for a long time, they exhibit anti-inflammatory activities and contain abundant anti-cancer compounds that exert direct cytotoxicity effects and indirect regulation in tumor microenvironment and cancer immunity, as well as improve chemotherapy [2–5]. For examples, *PNAS* reported that epigallocatechin gallate (EGCG) targeting Laminin receptor (Lam 67R) shows promising efficacy in treating prostate cancer [6]. *British Journal of Pharmacology* described that ginsenoside Rh2 inhibits P-glycoprotein (P-gp) activity to reverse multidrug resistance [7]. *The American Journal of Chinese Medicine* demonstrated that curcumin induces autophagy to enhance apoptotic cell death [8]. *Journal of Ethnopharmacology* reviewed that berberine potentially represses tumor progression and is expected to be safe, effective and affordable agent for cancer patients [9]. *Chinese Medicine* presented that shikonin exerts synergistic effects with chemotherapeutic agent [10]. However, the anti-cancer targets of these pharmacodynamic compounds are still not clear, and this is the major obstacle for the application and development of Chinese herbal medicine.

This review in Chinese herbal medicine and cancer focuses on summarizing experimental results and conclusions from English literatures reported since 2011. Literature search was conducted in peer-reviewed and clinical databases, which include PubMed (https://www.ncbi.nlm.nih.gov/pubmed), Web of Science (http://www.webofknowledge.com), Medline (https://www.medline.com), Scopus (https://www.scopus.com), and Clinical Trials (https://clinicaltrials.gov) using the following keywords: Cancer, Tumor, Neoplasm, Chinese herbs, Chinese medicine, Herbal medicine. To provide new insights into the critical path ahead, the pharmacological effects, novel mechanism of action, relevant clinical studies, innovative applications in combined therapy, and immunomodulation of the popular compounds originated from Chinese herbal medicine were reviewed systemically.

**Fig. 1:**
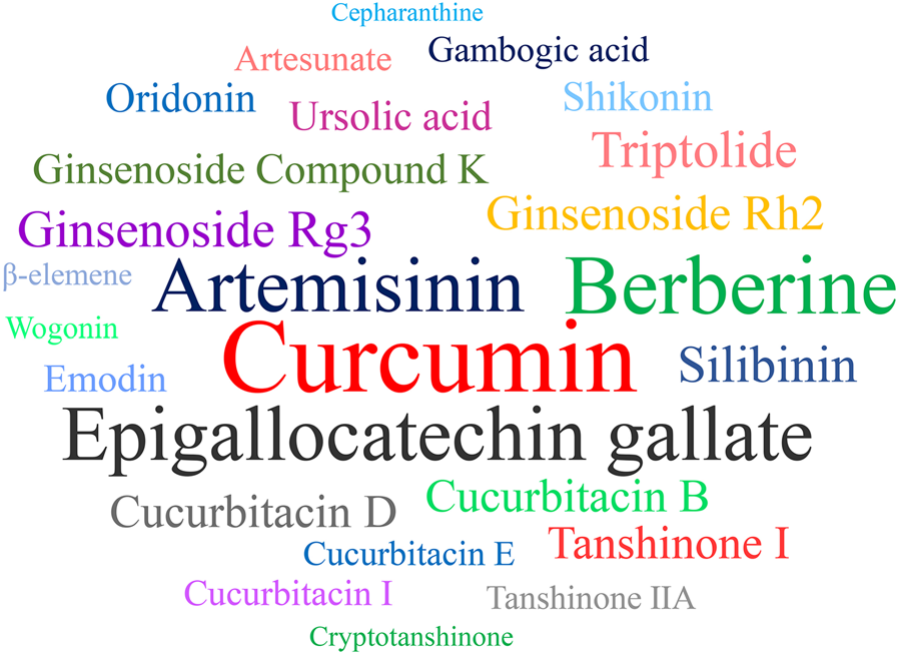
The anti-cancer compounds from Chinese herbal medicine (CHM). The popular anti-cancer compounds in CHM presented as a “word cloud”, in which the size of each name is proportional to the number of publications of the compounds

Different natural products derived from Chinese herbal medicine, including curcumin, EGCG, berberine, artemisinins, ginsenosides, ursolic acid (UA), silibinin, emodin, triptolide, cucurbitacins, tanshinones, ordonin, shikonin, gambogic acid (GA), artesunate, wogonin, β-elemene, and cepharanthine, were identified with emerging anti-cancer activities, such as anti-proliferative, pro-apoptotic, anti-metastatic, anti-angiogenic effects, as well as autophagy regulation, multidrug resistance reversal, immunity balance, and chemotherapy improvement in vitro and in vivo. These compounds are considered popular with over 100 supported publications and are selected to be discussed in more details. Figure 1 shows the word cloud of these compounds. In this review, the advantages and drawbacks of representative Chinese herbal medicine-derived compounds in different types of cancers were also highlighted and summarized.

### Curcumin

**Fig. 2:**
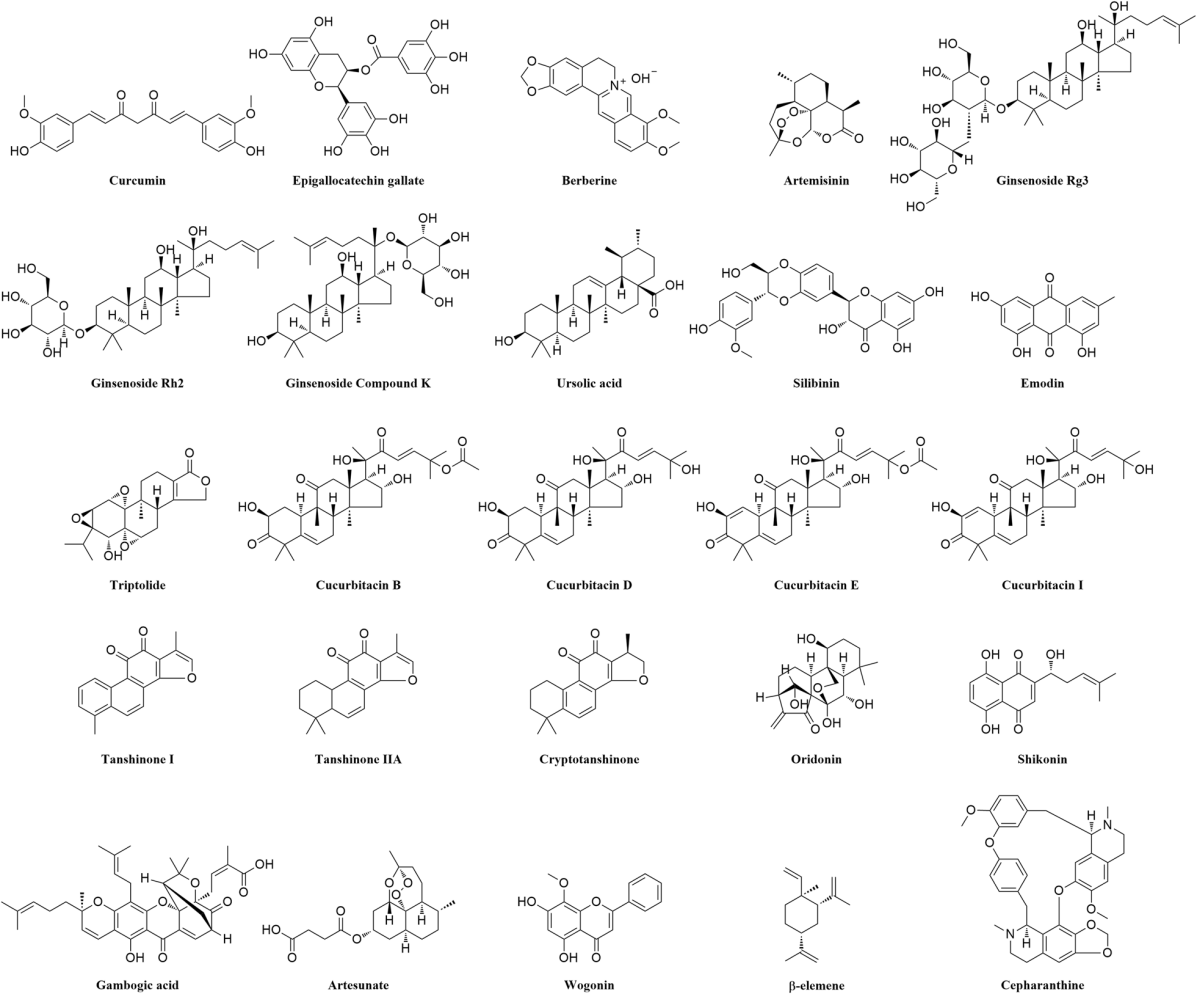
Chemical structures of anti-cancer compounds from Chinese herbal medicine

Curcumin (Fig. 2) is a polyphenol compound extracted mainly from the rhizomes of *Curcuma longa*, *Curcuma zedoaria* and *Acorus calamus* L. with many biological activities, but it has poor water solubility and stability [11]. Clinical evidence and extensive studies showed that curcumin has various pharmacology effects, including anti-cancer, anti-inflammatory, and anti-oxidative activities [12–14]. Curcumin and its analogues are shown to be emerging as effective agents for the treatment of several malignant diseases such as cancer. Numerous studies have shown that curcumin and its preparations can inhibit tumors in almost all parts of the body, including head and neck, ovarian, skin and gastric cancers [15–20]. Curcumin is shown to exhibit many anti-cancer effects through the inhibition of cell proliferation, promotion of cell apoptosis, prevention of tumor angiogenesis and metastasis, and the induction of autophagy [21–25].

Curcumin inhibits cell growth, induces cell cycle arrest and apoptosis in esophageal squamous cell carcinoma EC1, EC9706, KYSE450, TE13 cells through STAT3 activation [12]. It also induces oxidative stress, which disrupts the mitochondrial membrane potential and causes the release of cytochrome c, thus inducing apoptosis [26]. Besides, curcumin is shown to induce autophagy [8, 21, 27–30]. It induces autophagy through 5′AMP-activated protein kinase (AMPK) activation, leading to Akt degradation, thus inhibiting cell proliferation and migration in human breast cancer MDA-MB-231 cells [21], while it inhibits cell growth partially through autophagy induction in human hepatocellular carcinoma HepG2 cells [29]. Moreover, curcumin can ameliorate Warburg effect in human non-small cell lung cancer (NSCLC) H1299, breast cancer MCF-7, cervical cancer HeLa and prostate cancer PC-3 cells through pyruvate kinase M2 down-regulation, a key regulator of Warburg effect [18]. In addition, tumor metastasis has always been a frustrating problem for anti-cancer therapy, and curcumin also exhibits anti-metastasis effects [31–35]. Curcumin inhibits cell invasion via AMPK activation in human colorectal cancer SW-480 and LoVo cells [31], whilst low-toxic level of curcumin efficiently inhibits cell migration and invasion through the inhibition of Ras-related C3 botulinum toxin substrate 1/p21 (Rac1) activated kinase 1 (Rac1/PAK1) pathway in human NSCLC 801D cells, and this effect is also confirmed in 801D xenograft mice [32]. By pulmonary administration of curcumin in mice, it overcomes the problem of its low bioavailability, and inhibits lung metastasis of melanoma [35].

The main target molecules and signaling involved in the pharmacological processes include reactive oxygen species (ROS), matrix metalloproteinases (MMPs), nuclear factor kappa-light-chain-enhancer of activated B cells (NF-κB), signal transducer and activator of transcription and cell cycle-related proteins [36–46]. Curcumin is shown to induce anti-cancer activities through the disruption of mitochondrial membrane potential and blockade at G2/M phase of the cell cycle in human epidermoid carcinoma A-431 cells [47]. In addition, mammalian target of rapamycin (mTOR) plays a vital role in curcumin-induced autophagy and apoptosis [30, 48–50]. Curcumin induces apoptosis and autophagy through the inhibition of phosphoinositide 3-kinase (PI3K)/Akt/mTOR pathway in human NSCLC A549 cells [30], while it induces autophagy by reducing Akt phosphorylation and mTOR in human melanoma A375 and C8161 cells [49].

Curcumin can also exert immunomodulatory effects against cancer cells. Theracurmin, a highly bioavailable form of curcumin, decreases pro-inflammatory cytokine secretion from activated T cells, and enhances T cell-induced cytotoxicity in human esophageal adenocarcinoma OE33 and OE19 cells, so it increases the sensitivity of the cells to T cell-induced cytotoxicity [51]. The natural killing (NK) cells can directly kill cancer cells, and curcumin can enhance the cytotoxicity effect of NK cells when NK cells are co-cultured with human breast cancer MDA-MB-231 cells, which is highly associated with signal transducer and activator of transcription 4 (STAT4) and signal transducer and activator of transcription 5 (STAT5) activation [52]. Besides, myeloid-derived suppressor cells (MDSCs) are immune-suppressive cells which are found in most cancer patients. Curcumin decreases interleukin (IL)-6 levels in the tumor tissues and serum of Lewis lung carcinoma (LLC)-bearing mice to impair the growth of MDSCs, so targeting MDSCs is important for the treatment of lung cancer [13]. Moreover, the anti-tumor immune response of curcumin is mediated through increased cluster of differentiation (CD)8^+^ T cell population and decreased regulatory T cell (T_reg_) population in tongue squamous cell carcinoma [53–55].

In order to overcome the solubility issues of curcumin and facilitate its intracellular delivery, a curcumin-loaded nanoparticle, curcumin-PLGA-NP, is synthesized. It has a tenfold increase in water solubility compared to curcumin, and shows threefold increased anti-cancer activities in human breast cancer MDA-MB-231 and NSCLC A549 cells [56]. Another curcumin-capped nanoparticle exhibits promising anti-oxidative and selective anti-cancer activities in human colorectal cancer HT-29 and SW-948 cells [57]. Moreover, a curcumin analog, WZ35, has high chemical stability, and higher efficacy in anti-cancer effects compared to curcumin in human gastric cancer SGC-7901 cells and SGC-7901 xenograft mice [20]. Another analog, B63, induces cell death and reduces tumor growth through ROS and caspase-independent paraptosis in human gastric cancer SGC-7901, BGC-823 and SNU-216 cells, 5-fluorouracil-resistant gastric cancer cells, and SGC-7901 xenograft mice [58].

Curcumin can be used with other chemotherapeutic agents to achieve synergistic effects, reduce adverse effects and enhance sensitivity. Tamoxifen and curcumin are packed into a diblocknanopolymer, and this nanopolymer reduces the toxicity of tamoxifen in normal cells and exhibits better anti-proliferative and pro-apoptotic effects in human breast cancer tamoxifen-sensitive and -resistant MCF-7 cells [59]. Triptolide has strong liver and kidney toxicities, and when combined with curcumin, they exert synergistic anti-cancer effects in ovarian cancer, as well as reduce the side effects of triptolide [60]. In addition, adriamycin, sildenafil, 5-fluorouracil, irinotecan, doxorubicin, paclitaxel, sorafenib, Kruppel-like factor 4, emodin, docosahexaene acid and apigenin are shown to exhibit synergistic effects with curcumin [61–71]. Similarly, copper supplementation significantly enhances the anti-tumor effects of curcumin in several oral cancer cells [72], while epigallocatechin-3-gallic acid ester (EGCG) increases the ability of curcumin to inhibit cell growth and induce apoptosis in human uterine leiomyosarcoma SKN cells [73].

Clinical trials can confirm or reveal the effects, adverse reactions and pharmacokinetics of the drugs. As the bioavailability of curcumin is very poor, many curcumin preparations are synthesized and tested in clinical trials [74–76]. A phase I study was conducted to investigate the safety and pharmacokinetics of theracurmin in pancreatic and biliary tract cancer patients who failed with standard chemotherapy [76]. They administered theracurmin every day with standard gemcitabine-based chemotherapy. No new adverse effects and no increase in the incidence of adverse effects were observed among these patients. A pilot phase II study demonstrated encouraging results for the combination of docetaxel/prednisone and curcumin in patients with castration-resistant prostate cancer. It was found that 59% of patients had prostate-specific antigen response and 40% of patients achieved partial response. This study has provided additional evidence for a high response rate and better tolerability with the use of curcumin during cancer therapy [77].

### Epigallocatechin gallate (EGCG)

EGCG, also known as epigallocatechin-3-gallate (Fig. 2), is the main polyphenol in green tea (*Camellia sinensis*). Epidemiological studies have indicated that consumption of green tea has potential impact of reducing the risk of many chronic diseases, such as cardiovascular diseases and cancer [78, 79]. EGCG possesses various biological effects including anti-obesity and anti-hyperuricemia, anti-oxidative, anti-viral, anti-bacterial, anti-infective, anti-angiogenic, anti-inflammatory and anti-cancer activities [80–84]. It is reported to present anti-cancer effects in variety of cancer cells, including lung, colorectal, prostate, stomach, liver, cervical, breast, leukemia, gastric, bladder cancers [85–90]. Among its anti-cancer activities, EGCG exhibits multiple pharmacological actions, including the suppression of cell growth, proliferation, metastasis and angiogenesis, induction of apoptosis, and enhancement of anti-cancer immunity [85, 86, 91–94].

EGCG can inhibit cell proliferation through multiple ways in many types of cancer cells. It inhibits cell proliferation in human bladder cancer SW-780, breast cancer MDA-MB-231 and NSCLC A549 cells, and inhibits tumor growth in gastric cancer SGC-7901 xenograft mice [89, 94, 95]. It also induces apoptosis in human oral cancer KB, head and neck cancer FaDu, NSCLC A549, and breast cancer MCF-7 cells [96, 97]. Besides, EGCG induces autophagy, and inhibition of autophagy can enhance EGCG-induced cell death in human mesothelimoa ACC-meso, Y-meso, EHMES-10, EHMES-1 and MSTO-211H, and primary effusion lymphoma BCBL-1 and BC-1 cells [98, 99]. In contrast, it induces cell death via apoptosis and autophagy in oral squamous cell carcinoma SCC-4 cells [84], so autophagy plays a dual role in EGCG-induced cell death. It can also suppress metastasis in human melanoma SK-MEL-5, SK-MEL-28, A375 and G361, NSCLC CL1-5, A549 and H1299 cells, and lung metastasis mice [85, 93, 100]. In addition, EGCG suppresses tumor angiogenesis in human NSCLC A549 cells and A549 xenograft mice [101].

EGCG mediates apoptosis which involves pro- and anti-apoptotic proteins in various cancer cells. It up-regulates pro-apoptotic proteins such as Bcl-2-associated X protein (Bax), and down-regulates anti-apoptotic proteins including B-cell lymphoma 2 (Bcl-2), B-cell lymphoma-extra large (Bcl-xL) and survivin [97, 102–104]. ER stress also plays an important role in EGCG-induced cell death. EGCG inhibits endoplasmic reticulum (ER) stress-induced protein kinase R-like endoplasmic reticulum kinase (PERK) and eukaryotic translation-initiation factor 2α (eIF2α) phosphorylation [105]. Besides, poly (ADP-ribose) polymerase (PARP) 16 is shown to activate ER stress markers, PERK and inositol-requiring enzyme 1α (IRE1α) [106]. ER stress-induced apoptosis, PERK and eIF2α phosphorylation by EGCG are suppressed in PARP16-deficient hepatocellular carcinoma QGY-7703 cells, so EGCG mediates apoptosis through ER stress, which is dependent on PARP16 [105]. Similarly, EGCG causes 78-kDa glucose-regulated protein (GRP78) accumulation in the ER, which up-regulates ER stress markers such as activating transcription factor 4 (ATF-4), X-box binding protein 1 (XBP-1) and C/EBP homologous protein (CHOP), and shifts into pro-apoptotic ER stress, leading to increased caspase-3 and -8 activities [107]. Furthermore, it suppresses cell migration and invasion by blocking tumor necrosis factor (TNF) receptor-associated factor 6 (TRAF6), MMP-2/c-Jun N-terminal kinase (JNK) and transforming growth factor-β (TGF-β) pathways [85, 93, 100].

In addition to anti-cancer effects, EGCG shows a significant inhibitory effect on interferon-γ (IFN-γ)-induced indoleamine 2,3-dioxygenase (IDO) expression, an enzyme that guides cancer to regulate immune response, in human colorectal cancer SW-837 cells [108], so this suggests that EGCG might be useful for chemoprevention and colorectal cancer treatment, and could be a potential agent for anti-tumor immunotherapy. EGCG is also found to be a potential immune checkpoint inhibitor, which down-regulates IFN-γ-induced B7 homolog 1 (B7-H1) levels, an immunoglobulin-like immune suppressive molecule, in human NSCLC A549 cells [109].

Although EGCG has numerous biological activities through different pathways, its efficacy demonstrated in in vivo studies is not always consistent with the results of in vitro studies. This can be due to its low oil solubility, metabolic instability and poor bioavailability [110]. Therefore, EGCG analogs and EGCG-loaded nanoparticles by modifying EGCG are developed, and they have been reported to enhance anti-cancer effects [111–113]. The peracetate-protected (−)-EGCG, a prodrug of EGCG obtained by modifying the reactive hydroxyl groups with peracetate groups, is shown to increase the bioavailability of EGCG and inhibit angiogenesis in endometrial cancer xenograft mice [111]. Besides, EGCG-DHA (docosahexaenoic) ester, a lipophilic derivative of EGCG, shows improved anti-oxidative effects compared to EGCG, and suppresses colon carcinogenesis in mice [112, 113]. In the last decade, many studies were carried out using EGCG-loaded nanoparticles including FA-NPS-PEG and FA-PEG-NPS (epigallocatechin gallate-β-lactoglobulin nanoparticles), EGCG-SLN (solid lipid nanoparticle), DT-EGCG-nanoethosomes, FCS-EGCG-NPs (chitosan coated nanoparticles), EGCG-dispersed selenium nanoparticles, ^198^AuNP-EGCg (gold nanoparticles), EGCG-loaded microspheres (EGCG/MS), and FCMPs (ferritin-chitosan Maillard reaction products) [6, 110, 114–121]. These EGCG nanoparticles can improve the targeting ability and efficacy of EGCG, which greatly promote the clinical application and development of EGCG analogs.

EGCG antagonizes toxicity induced by anti-cancer chemotherapeutic agents, and sensitizes chemo-resistant cancer cells. It also exerts synergistic effects with anti-cancer agents in various cancer cells, such as cisplatin, oxaliplatin, temozolomide, resveratrol, doxorubicin, vardenafil, curcumin, erlotinib [122–129]. EGCG can enhance the sensitivity of cisplatin through copper transporter 1 (CTR1) up-regulation, which results in the accumulation of cellular cisplatin and cisplatin–DNA adducts in human ovarian cancer SKOV3 and OVCAR3 cells, and the combination of EGCG and cisplatin suppresses tumor growth in OVCAR3 xenograft mice [122]. The combined low concentration of EGCG and curcumin remarkably inhibits cell and tumor growth in human NSCLC A549 and NCI-H460 cells, and A549 xenograft mice through cell cycle arrest [123].

To evaluate the tolerance, safety, pharmacokinetics and efficacy of EGCG in humans, clinical trials have been or are currently being conducted for cancer treatment. During a phase I clinical trial for the treatment of radiation dermatitis, patients with breast cancer received adjuvant radiotherapy and EGCG solution. It was found that the maximum dose (660 μM) of EGCG was well tolerated and the maximum tolerated dose was undetermined [130]. It was concluded that EGCG was effective for treating radiation dermatitis. Moreover, a phase II clinical trial was conducted to investigate the benefits of EGCG as a treatment for acute radiation-induced esophagitis (ARIE) for patients with stage III lung cancer. The oral administration of EGCG was shown to be effective and phase III clinical trial to study the potential effects of EGCG to ARIE treatment was anticipated [131].

### Berberine

Berberine (Fig. 2) is an isoquinoline alkaloid mainly extracted from medicinal plants such as *Coptidis chinensis* Franch., *Mahonia bealei* (Fort.) Carr., and *Phellodendron chinense* Schneid. [132]. Berberine has diverse pharmacological effects and is normally used for the treatment of gastroenteritis [133, 134]. It exhibits significant anti-cancer effects in a wide spectrum of cancers including ovarian, breast, esophageal, and thyroid cancers, leukemia, multiple myeloma, nasopharyngeal carcinoma, and neuroblastoma, through inducing cell cycle arrest and apoptosis, inhibiting metastasis and angiogenesis [135–143].

Berberine can induce cell cycle arrest in various cancer cells [137, 144, 145]. Berberine induces G1 and G2/M phase arrest in murine prostate cancer RM-1 cells, and G1 cell arrest by regulating cyclins D1 and E expressions in human HER2-overexpressed breast cancer cells [144, 145]. However, berberine induces G1 phase arrest in human estrogen receptor positive breast cancer MCF-7 cells but not in estrogen receptor negative MDA-MB-231 cells [137]. Besides, it inhibits cell proliferation by inducing apoptosis in human colorectal cancer HCT-8 cells [146]. In p53-null leukemia EU-4 cells, berberine induces p53-independent and X-linked inhibitor of apoptosis protein (XIAP)-mediated apoptosis, which is associated with mouse double minute 2 homolog (MDM2) and proteasomal degradation [135]. Mitochondrial-mediated apoptosis with Bcl-2-like protein 11 (Bim) up-regulation and Forkhead box O (FoxO) nuclear retention is vital in berberine-induced apoptosis [147]. In addition, berberine can induce autophagic cancer cell death through increased GRP78 levels and enhancing the binding ability of GRP78 to VPS34 in human colorectal cancer HCT-116 cells [148], whilst it induces autophagy through inhibiting AMPK/mTOR/UNC-51-like kinase 1 (ULK-1) pathway in human glioma U251 and U87 cells [149]. In contrast, berberine induces protective autophagy in human malignant pleural mesothelioma NCI-H2452 cells, and inhibition of autophagy promotes berberine-induced apoptosis [150]. Therefore, autophagy plays a dual role in berberine-induced apoptosis. Furthermore, berberine also inhibits tumor migration and invasion [143, 151]. It up-regulates plasminogen activator inhibitor-1 (PAI-1), a tumor suppressor that down-regulates urokinase-type plasminogen activator (uPA) and antagonizes uPA receptor to suppress metastasis in human hepatocellular carcinoma Bel-7402 and SMMC-7721 cells [143]. Berberine also inhibits epithelial mesenchymal transition through PI3K/Akt pathway in murine melanoma B16 cells, [151], and suppresses angiogenesis in glioblastoma U87 xenograft mice and HUVECs [152, 153].

Berberine interacts with diverse molecular targets as it binds to nucleic acids via specific deoxyribonucleic acid (DNA) sequences [154]. Several mechanisms have been identified for the anti-proliferative effects of berberine, including down-regulation of cyclins A, D, cyclin-dependent kinase (CDK) 1, CDK4, MMP-2 and janus kinase 2 (Jak2)/vascular endothelial growth factor (VEGF)/NF-κB/activator protein 1 (AP-1) pathway, and induction of autophagic cell death via mTOR signaling pathway [149, 155, 156]. Berberine also induces mitochondrial-mediated apoptosis through the loss of mitochondrial membrane potential, cytochrome c release, caspase and PARP activation, up-regulation of pro-apoptotic Bcl-2 family proteins, and down-regulation of anti-apoptotic Bcl-2 family proteins [150, 157–159]. It can also activate apoptosis-inducing factor to induce ROS-mediated cell death in pancreatic, breast, and colon cancers [158, 160, 161].

Immunotherapy has made great progress to cancer treatment over the past few years. Toll-like receptors (TLRs) can activate innate immune responses for host defense [162]. Berberine inhibits proto-oncogene tyrosine kinase Src activation and TLR4-mediated chemotaxis in lipopolysaccharide (LPS)-induced macrophages [163]. Besides, IDO1 inhibitors are promising candidates for cancer immunotherapy [164]. Berberine and its derivatives are shown to exhibit anti-cancer activity through cell killing by NK cells via IDO1 [165]. IL-8 is associated with metastasis, and berberine decreases IL-8 levels to inhibit cell growth and invasion in triple-negative breast cancer cells [166].

Berberine has low oral bioavailability as well as poor intestinal absorption [167]. As it has pronounced anti-microbial activity against gut microbiota, high dosage can translates into adverse events [168]. This limits the clinical use of berberine, and different approaches have been applied to improve the bioavailability of berberine. d-α-Tocopheryl polyethylene glycol 1000 succinate enhances the intestinal absorption of berberine by inhibiting P-gp activity in rats [167]. A self-microemulsifying drug delivery system is developed to improve the bioavailability of berberine, the bioavailability is increased by 2.42-fold [169]. Ber8, a 9-alkylated derivative of berberine, has better cytotoxicity and cellular uptake than berberine, and further inhibits cell proliferation and induces cell cycle arrest in different cell lines, including SiHa, HL-60, and A549 cells [170].

The combination of berberine and chemo- or radio-therapies provides synergistic anti-cancer effects [171, 172]. Taxol combined with berberine significantly slows down cell growth in human epidermal growth factor receptor 2 (HER2)-overexpressed breast cancer cells [145], while the combined administration of berberine and caffeine enhances cell death through apoptosis and necroptosis in human ovarian cancer OVCAR3 cells [173]. The combination therapy of berberine and niraparib, a PARP inhibitor, markedly enhances apoptosis and inhibits tumor growth in ovarian cancer A2780 xenograft mice [174]. Therefore, combination of berberine with other therapies is a promising treatment for the alternative cancer therapy.

Previous pre-clinical research and animal studies have demonstrated the anti-tumor action of berberine hydrochloride. The people with a history of colorectal cancer might be at higher risk for adenomas, thus they are particularly suitable for the study of the chemopreventive effects of berberine hydrochloride in adenomas. A randomized, double-blind, placebo-controlled trial was designed to determine whether the daily intake of 300 mg of berberine hydrochloride could decrease the occurrence of new colorectal adenomas in patients with a history of colorectal cancer, and it is currently ongoing. Another phase II clinical trial of berberine and gefitinib is also ongoing in patients with advanced NSCLC and activating EGFR mutations.

### Artemisinins

Artemisinin (Fig. 2) is a sesquiterpene peroxide derived from annual wormwood (*Artemisia annua* L.), which was originally used as Traditional Chinese Medicine for treating malaria and related symptoms such as fever and chills [175]. Since the 2015 Nobel Prize in Physiology or Medicine conferred to Chinese scientist, Youyou Tu, artemisinin drew attention to worldwide [176]. Beside from their well-established anti-malarial effects, artemisinin and its derivatives (ARTs), including dihydroartemisinin (DHA), artesunate, artemether and arteether, are also found to exhibit potent anti-cancer activities in many studies [177–182]. DHA and artesunate are the most studied ART derivatives for cancer treatment, and artesunate will be discussed in a separate section. The anti-cancer effects of ARTs are demonstrated in a broad spectrum of cancer cells including lung, liver, pancreatic, colorectal, esophageal, breast, ovarian, cervical, head and neck, and prostate cancers [183–191]. The anti-cancer activities of ARTs include induction of apoptosis and cell cycle arrest, inhibition of cell proliferation and growth, metastasis and angiogenesis [189, 192–195].

ART inhibits cell proliferation, migration and invasion, and induces apoptosis in human breast cancer MCF-7 cells [193, 196], while DHA suppresses cell growth through cell cycle arrest and apoptosis in human hepatocellular carcinoma HepG2 cells and HepG2 xenograft mice [178]. Similarly, ART induces apoptosis in murine mastocytome P815 cells and hamster kidney adenocarcinoma BSR cells, and inhibits tumor growth in P815 xenograft mice [177]. Moreover, autophagy plays a vital role in ART-mediated anti-cancer activities [190, 197–201]. DHA can induce autophagy-dependent cell death in human cervical cancer HeLa cells, cholangiocarcinoma KKU-452, KKU-023 and KKU-100, and tongue squamous cell carcinoma Cal-27 cells [190, 198, 199], while ART induces autophagy-mediated cell cycle arrest in human ovarian cancer SKOV3 cells [200]. DHA is also shown to induce autophagy by suppressing NF-κB activation in several cancer cells including RPMI 8226, NB4, HCT-116, and HeLa cells [202]. Furthermore, ART and DHA can also inhibit metastasis in various cancer cells such as non-small-cell lung carcinoma (NSCLC), ovarian and lung cancer cells [184, 189, 203]. Apart from apoptosis and metastasis, the inhibition of angiogenesis is also a crucial approach in cancer treatment. ART inhibits angiogenesis through mitogen-activated protein kinase (MAPK) activation in osteosarcoma [204], whilst DHA exerts strong anti-angiogenic effect by repressing extracellular signal–regulated kinase (ERK) and NF-κB pathways in human umbilical vein endothelial cells (HUVECs) and pancreatic cancer, respectively [194, 195].

In the past decades, studies have been focused on studying the anti-cancer mechanisms of ARTs, but there are contentions. ARTs inhibit cancer cell proliferation mainly by the induction of apoptosis through mitochondrial-dependent pathways [196, 205, 206]. ART mediates the release of cytochrome c and caspase-9 cleavage, leading to increased apoptosis in human breast cancer MCF-7 cells [196]. DHA induces apoptosis through Bcl-2 down-regulation in human cervical cancer HeLa and Caski cells [205], and via Bim-dependent intrinsic pathway in human hepatocellular carcinoma HepG2 and Huh7 cells [206]. Interestingly, ART is demonstrated to be an inhibitor of anti-cancer target, histone deacetylases (HDAC) [196]. In addition, another mechanism of killing tumor cells by ARTs is iron-dependent cell death called ferroptosis, a new form of cell death, so ferroptosis becomes an attractive strategy for cancer treatment [183, 207].

DHA can enhance the anti-tumor cytolytic activity of γδ T cells against human pancreatic cancer SW1990, BxPC-3 and Panc-1 cells [208], and ART also potentiates the cytotoxicity of NK cells to mediate anti-tumor activity [209]. Similarly, ART inhibits tumor growth through T cell activation and T_reg_ suppression in breast cancer 4T1 xenograft mice [188]. Therefore, this provides a novel strategy for treating pancreatic cancer with immunotherapy.

ART has poor water solubility and bioavailability. In order to solve this issue, ART is encapsulated into micelles by nanoprecipitation to form ART-loaded micelles [210]. The ART-loaded micelles enhance the drug exposure time and accumulation in breast cancer 4T1 xenograft mice, and shows specific toxicity in human and murine breast cancer MCF-7 and 4T1 cells. A mitochondrial-targeting analog of ART is also synthesized to specifically target mitochondria for enhancing the inhibition of cell proliferation in various cancer cells including HCT-116, MDA-MB-231, HeLa and SKBR3 cells [211]. Moreover, dimmers of ART are also synthesized by polyamine linkers, and they further inhibit cell proliferation in human breast cancer MCF-7 cells and angiogenesis in HUVECs [212].

Many studies show the synergistic effects of ARTs with other compounds or therapeutic approaches. The combined treatment of ART and resveratrol markedly inhibits cell proliferation and migration, and enhances apoptosis and ROS production in human cervical cancer HeLa and hepatocellular carcinoma HepG2 cells [213]. Similarly, the use of combined DHA and gemcitabine exhibits strong synergistic effects on the loss of mitochondrial membrane potential and induction of apoptosis in human NSCLC A549 cells [214]. DHA also reinforces the anti-cancer activity of chemotherapeutic agent, cisplatin, in cisplatin-resistant ovarian cancer cells [215]. Studies also demonstrate the enhancement of sensitivity by DHA in photodynamic therapy in esophageal cancer [182, 216]. Therefore, this suggests that ARTs could be potential anti-cancer agents.

The population pharmacokinetic properties of DHA were investigated using the plasma and saliva of breast cancer patients for long-term treatment (> 3 weeks) [217]. The salivary DHA concentration was proportionally correlated with the plasma DHA concentration, so saliva is a good use for monitoring DHA levels in the body. An artemisinin analog, Artenimol-R, was shown to improve clinical symptoms and tolerability in patients with advanced cervical cancer [218].

### Ginsenosides

Ginsenosides (Fig. 2) are the main bioactive dammarane triterpenoids derived from the rhizomes of many plants including *Panax notoginseng* (Burk.) F. H. Chen, *Panax ginseng* and *Cinnamomum cassia* Presl., with various biological effects including anti-oxidative, anti-inflammatory, and anti-cancer activities [219–222]. Ginsenosides mainly exert anti-cancer effects in colorectal, breast, liver and lung cancers, through inhibiting cell proliferation and migration, angiogenesis, and reversing drug resistance [7, 223–230]. Ginsenoside Rg3, ginsenoside Rh2, and compound K are the primary bioactive compounds among ginsenosides for cancer prevention.

Ginsenoside Rg3 inhibits cell viability and induces cell apoptosis in human ovarian cancer HO8910 cells [231], hepatocellular carcinoma Hep1-6, HepG2 and SMMC-7721, breast cancer MCF-7, MDA-MB-231, MDA-MB-453 and BT-549, and NSCLC A549, H23 and Lewis lung carcinoma cells [232–238]. It induces cell cycle arrest at G1 phase in human melanoma A375, and multiple myeloma U266, RPMI 8226 and SKO-007 cells [239, 240], and inhibits cell migration in human colorectal cancer LoVo, SW-620 and HCT-116 cells [240]. Ginsenoside Rg3 can also modulate the tumor environment through inhibiting angiogenesis and enhancing anti-tumor immune responses [241]. Moreover, ginsenoside Rh2 exhibits anti-tumor activity in human NSCLC H1299 cells and H1299 xenograft mice, through the induction of ROS-mediated ER-stress-dependent apoptosis [242]. It also suppresses cell proliferation and migration, and induces cell cycle arrest in human hepatocellular carcinoma HepG2 and Hep3B cells, and inhibits tumor growth in HepG2 xenograft mice [243]. Compound K, an intestinal bacterial metabolite of ginsenosides, also induces cell cycle arrest and apoptosis in human colorectal cancer HCT-116 cells, and suppresses tumor growth in HCT-116 xenograft mice [244]. It also efficiently inhibits cell proliferation and induces apoptosis through mitochondrial-related pathways in human hepatocellular carcinoma MHCC97-H cells [245]. Furthermore, 20(S)-ginsenoside Rg3 induces autophagy to mediate cell migration and invasion in human ovarian cancer SKOV3 cells [246]. In contrast, it sensitizes NSCLC cells to icotinib and hepatocellular carcinoma cells to doxorubicin through the inhibition of autophagy [247, 248]. Besides, ginsenoside Rh2 inhibits cell growth partially through the coordination of autophagy and β-cateninin signaling in human heptocellular carcinoma HepG2 and Huh7 cells [249]. Compound K induces autophagy-mediated apoptosis through AMPK/mTOR and JNK pathways in human NSCLC A549 and H1975 cells [250], while it also induces autophagy and apoptosis through ROS and JNK pathways in human colorectal cancer HCT-116 cells [251]. Therefore, autophagy plays a dual role in cancer via different signaling routes.

In recent years, the potential anti-cancer mechanisms of ginsenoside Rg3 have been demonstrated in various cancer models, which include the inhibition of cell proliferation and induction of apoptosis via down-regulating PI3K/Akt, and activation of caspase-3 and -9 and Bcl-2 family proteins [234, 252], induction of cell cycle arrest by regulating CDK pathway [240], inhibition of metastasis through reducing the expressions of aquaporin 1, C–X–C chemokine receptor type 4 (CXCR4) and hypoxia-inducible factor 1α (HIF-1α) [253–255]. Moreover, 20(S)-ginsenoside Rh2 is shown to bind to recombinant and intracellular annexin A2 directly, and this inhibits the interaction between annexin A2 and NF-κB p50 subunit, which decreases NF-κB activation [256]. NF-κB is important in cell survival, and 20(S)-ginsenoside Rh2 can inhibit cell survival through NF-κB pathway. Furthermore, p53 also plays a vital role in ginsenoside-induced anti-cancer activities [244, 257, 258]. Ginsenoside Rh2 induces cell death through p53 activation in human colorectal cancer HCT-116 and SW-480 cells [257], while ginsenoside Rg3 and compound K induces apoptosis and cell cycle arrest through p53/p21 up-regulation in human colorectal cancer HCT-116, SW-480 and HT-29, and gallbladder cancer NOZ and GBC-SD cells, respectively [244, 258].

For the promotion of immunity, ginsenoside Rg3 can enhance lymphocyte proliferation and T helper type 1 cell (Th1)-related cytokine secretion including IL-2 and IFN-γ in hepatacellular carcinoma H22-bearing mice, and inhibit tumor growth partly through the induction this cellular immunity [259]. Ginsenoside Rg3 can also down-regulate the levels of B7-H1 and B7 homolog 3 (B7-H3), immunoglobulin-like immune suppressive molecules, to modulate tumor microenvironment and enhance anti-tumor immunity, and these molecules are negatively associated with overall survival in colorectal cancer patients [241]. It also ameliorates cisplatin resistance by down-regulating B7-H1 levels and resuming T cell cytotoxicity in human NSCLC A549 and A549/DDP cells [260]. In addition, ginsenoside Rh2 can also enhance anti-tumor immunity in melanoma mice by promoting T cell infiltration in the tumor and cytotoxicity in spleen lymphocytes [261].

The combination of ginsenosides with other chemotherapeutic agents provides significant advantages for cancer treatment. Ginsenoside Rg3 alone demonstrates modest anti-angiogenic effects, and displays additive anti-angiogenic effects in B6 glioblastoma rats when combined with temozolomide [262]. When it is combined with paclitaxel, it enhances cytotoxicity and apoptosis through NF-κB inhibition in human triple-negative breast cancer MDA-MB-231, MDA-MB-453 and BT-549 cells [233].

Ginsenosides have a long history of use as traditional medicine to treat many diseases in China. Relatively few clinical studies have been performed in humans eventhough ginseng products are widely recognized to have therapeutic effects when used alone or in combination with other chemotherapeutic agents. Therefore, clinical studies are needed to confirm the safety of such uses. A phase II clinical trial is conducting to assess the safety and efficacy of ginsenoside Rg3 in combination with first-line chemotherapy in advanced gastric cancer. Patients with advanced NSCLC and epidermal growth factor receptor-tyrosine kinase inhibitor (EGFR-TKI) mutation were recruited in a study that investigated the safety and efficacy of the combined therapy, ginsenoside Rg3 and EGFR-TKI. It was shown that this therapy increased progression-free survival, overall survival and objective response rate compared to EGFR-TKI alone [263]. In another study, the safety and efficacy of combined ginsenoside Rg3 and transcatheter arterial chemoembolization (TACE) were studied in patients with advanced hepatocellular carcinoma. The results showed that this therapy ameliorated TACE-induced adverse effects and prolonged the overall survival compared to the use of TACE alone [264].

### Ursolic acid (UA)

As an ursane-type pentacyclic triterpenic acid, UA (Fig. 2) can be found in the berries and leaves of a series of natural medicinal plants, including *Vaccinium macrocarpon* Ait. (cranberry), *Arctostaphylos uva*-*ursi* (L.) Spreng (bearberry), *Rhododendron hymenanthes* Makino*, Eriobotrya japonica, Rosemarinus officinalis, Calluna vulgaris, Eugenia jambolana* and *Ocimum sanctum,* as well as in the wax-like protective coatings of fruits such as pears, apples and prunes [265]. UA has numerous biochemical and pharmacological effects including anti-inflammatory, anti-oxidative, anti-proliferative, anti-atherosclerotic, anti-leukemic, anti-viral, and anti-diabetic effects [266–272]. It also exerts anti-cancer activities in ovarian, breast, gastric, prostate, lung, liver, bladder, pancreatic, and colorectal cancers [273–281].

UA can be used as a potential therapeutic agent for the treatment of various cancers [281–289]. It induces apoptosis through both extrinsic death receptor and mitochondrial death pathways in human breast cancer MDA-MB-231 cells [289], and inhibits cell proliferation and induces pro-apoptosis in human breast cancer MCF-7 cells by FoxM1 inhibition [282]. UA also inhibits cell and tumor growth through suppressing NF-κB and STAT3 pathways in human prostate cancer DU-145 and LNCaP cells, and DU-145 xenograft mice [283], and induces apoptosis in human prostate cancer PC-3 cells [284]. Similarly, UA induces apoptosis and inhibits cell proliferation in human colorectal cancer HCT-15, HCT-116, HT-29 and Caco-2 cells [286, 287]. UA is also shown to induce autophagy to mediate cell death in murine cervical cancer TC-1 cells [290], and promote cytotoxic autophagy and apoptosis in human breast cancer MCF-7, MD-MB-231 and SKBR3 cells [291]. It also inhibits cell growth by inducing autophagy and apoptosis in human breast cancer cells T47D, MCF-7 and MD-MB-231 cells [279]. In contrast, UA induces autophagy, but the inhibition of autophagy enhances UA-induced apoptosis in human oral cancer Ca922 and SCC2095, and prostate cancer PC-3 cells [265, 292]. Therefore, autophagy plays a dual role in UA-induced apoptosis via different signaling pathways. In addition, UA inhibits tumor angiogenesis through mitochondrial-dependent pathway in Ehrlich ascites carcinoma xenograft mice [293].

Increasing evidence has linked the anti-cancer activities of UA to the activation of mitochondrial-dependent signaling pathways, including mitochondrial energy metabolism, oxidative stress and p53‑mediated mitochondrial pathways [289, 291, 293]. UA is demonstrated to have apoptosis-promoting and anti-proliferative capacities via modulating the expressions of mitochondrial-related proteins such as Bax, Bcl-2, cytochrome c and caspase-9 [289, 293]. It can also induce oxidative stress and disruption of mitochondrial membrane permeability to mediate apoptosis in human osteosarcoma MG63 and cervical cancer HeLa cells [294, 295]. In addition, p53 pathway also contributes to the anti-cancer effects of UA. UA induces apoptosis and cell arrest through p21-mediated p53 activation in human colorectal cancer SW-480 and breast cancer MCF-7 cells [296, 297], and this p53 activation is through inhibiting negative regulators of p53, MDM2 and T-LAK cell-originated protein kinase (TOPK) [297].

Studies have reported the cancer immunomodulatory activities of UA [279, 293]. UA down-regulates NF-κB to inhibit cell growth and suppress inflammatory cytokine levels including TNF-α, IL-6, IL-1β, IL-18 and IFN-γ in human breast cancer T47D, MCF-7 and MDA-MB-231 cells [279]. It also modulates the tumor environment by modulating cytokine production such as TNF-α and IL-12 in ascites Ehrlich tumor [293].

UA is insoluble in water, with poor pharmacokinetic properties including poor oral bioavailability, low dissolution and weak membrane permeability [298]. Some new drug delivery technologies have been developed to overcome these problems including the uses of liposomes [280, 299–302], solid dispersions [303], niossomal gels [304], and nanoliposomes [278]. Liposome is the most commonly used drug delivery system. A chitosan-coated UA liposome is synthesized with tumor targeting and drug controlled release properties, and has fewer side effects [302]. It enhances the inhibition of cell proliferation and tumor growth in human cervical cancer HeLa cells and U14 xenograft mice. Besides, a pH-sensitive pro-drug delivery system is also synthesized, and this pro-drug enhances cellular uptake and bioavailability of UA [305]. It further inhibits cell proliferation, cell cycle arrest and induces apoptosis in human hepatocellular carcinoma HepG2 cells.

UA can also be used in combination with other drugs. The combined treatment of zoledronic acid and UA enhances the induction of apoptosis and inhibition of cell proliferation through oxidative stress and autophagy in human osteosarcoma U2OS and MG63 cells [306], whilst the combination of UA and curcumin inhibits tumor growth compared to UA alone in skin cancer mice [307]. Moreover, UA combined with doxorubicin enhances the cellular uptake of doxorubicin, and reverses multi-drug resistance (MDR) in human breast cancer MCF-7/ADR cells [308].

A human clinical study was conducted to investigate the toxicity and pharmacokinetics of UA-liposomes (UAL) including dose-limiting toxicity and maximum tolerated dose in healthy adult volunteers and patients with advanced solid tumors [309]. UAL had manageable toxicities under the dose of 98 mg/m^2^, as well as a linear pharmacokinetic profile, so it was suggested that UA could be developed as a potential and safe drug [309].

### Silibinin

Silibinin (Fig. 2), one of the flavonoids isolated from *Silybum marianum* L. Gaertn, is commonly exploited for the treatment hepatic diseases in China, Germany and Japan. In addition, silibinin is also found to display various biological activities including anti-oxidative, anti‑proliferative, anti-bacterial, anti-fungal, neuro-protective, anti-leishmanial, anti-osteoclastic and anti-metastatic activities [310–317]. Previous studies have reported that silibinin exerts remarkable effects in numerous cancers such as renal, hepatocellular and pancreatic carcinoma, bladder, breast, colorectal, ovarian, lung, salivary gland, prostate and gastric cancers, through the induction of apoptosis, inhibition of tumor growth, metastasis and angiogenesis [318–328].

Silibinin suppresses epidermal growth factor-induced cell adhesion, migration and oncogenic transformation through blocking STAT3 phosphorylation in triple negative breast cancer cells [329]. It strongly suppresses cell proliferation and induces apoptosis in human pancreatic cancer AsPC-1, BxPC-3 and Panc-1 cells, and induces cell cycle arrest at G1 phase in AsPC-1 cells [330]. It can also induce apoptosis via non-steroidal anti-inflammatory drug-activated gene-1 (NAG-1) up-regulation in human colorectal cancer HT-29 cells [331], and induces mitochondrial dysfunction to mediate apoptosis in human breast cancer MCF-7 and MDA-MB-123 cells [332]. Moreover, silibinin induces autophagic cell death via ROS-dependent mitochondrial dysfunction in human breast cancer MCF-7 cells [333]. In contrast, it induces autophagy to exert protective effect against apoptosis in human epidermoid carcinoma A-431, glioblastoma A172 and SR, and breast cancer MCF-7 cells [334–336], and autophagy inhibition enhances silibinin-induced apoptosis in human prostate cancer PC-3 cells [337]. Silibinin also induces autophagy to inhibit metastasis in human renal carcinoma ACHN and 786-O cells, and salivary gland adenoid cystic carcinoma cells [317]. Therefore, autophagy plays a dual role in silibinin-induced anti-cancer effects. In addition, silibinin inhibits angiogenesis in human prostate cancer PCa, LNCaP and 22Rv1 cells [327].

Silibinin exhibits anti-cancer activities mainly due to the cell cycle arrest [330, 338–341]. It induces G1 phase arrest in human pancreatic cancer SW1990 and AsPC-1, and breast cancer MCF-7 and MCF-10A cells [330, 339, 340], whilst it causes G2 phase arrest in human cervical cancer HeLa, and gastric cancer MGC-803 and SGC-7901 cells [338, 341]. It also decreases the expressions of CDKs such as CDK1, CDK2, CDK4 and CDK6 that are involved in G1 and G2 progression [338, 339]. Besides, silibinin suppresses metastasis through ERK1/2 and MMP-9 down-regulation in human thyroid cancer TPC-1, breast cancer MCF-7, renal carcinoma ACHN, OS-RC-2 and SW-839, and epidermoid carcinoma A-431 cells [342–344]. In addition, silibinin induces apoptosis and inhibits proliferation through the suppression of NF-κB activation [345–348]. On the other hand, silibinin is shown to induce apoptosis through the promotion of mitochondrial dysfunction, including increased cytochrome c and Bcl-2 levels, the loss of mitochondrial membrane potential, and decreased adenosine triphosphate (ATP) levels [332, 333, 349, 350].

Silibinin has immunomodulatory effects in cancer and immunity. The MDSCs are associated with immunosuppression in cancer, and silibinin increases the survival rate in breast cancer 4T1 xenograft mice, and reduces the population of MDSCs in their blood and tumor [351]. There was also a reduction in macrophage infiltration and neutrophil population in silibinin-treated prostate cancer TRAMPC1 xenograft mice [352]. These studies suggest a role of immunity in its anti-tumor effects.

Silibinin has poor water solubility and bioavailability, so it limits its efficacy in anti-cancer activities [353]. Advanced technologies such as nanoprecipitation technique are used to solve this issue [325, 353–356]. Silbinin is encapsulated in Eudragit^®^ E nanoparticles in the presence of polyvinyl alcohol, and these nanoparticles enhance apoptosis and cytotoxicity in human oral cancer KB cells [353]. The silibinin-loaded magnetic nanoparticles further inhibit cell proliferation in human NSCLC A549 cells [325], while silibinin-loaded chitosan nanoparticles enhances cytotoxicity compared to silibinin alone in human prostate cancer DU-145 cells [356].

The combination of silibinin and other drugs are used in cancer treatment to enhance the efficacy of anti-cancer effects [324, 357–359]. The combination of curcumin and silibinin enhances the inhibition of cell growth and reduction in telomerase gene expression compared to silibinin alone in human breast cancer T47D cells [357]. The mixture of luteolin and silibinin also shows synergistic effects on the attenuation of cell migration and invasion, and induction of apoptosis in human glioblastoma LN18 and SNB19 cells [358]. Silibinin and paclitaxel combination enhances apoptosis and up-regulates tumour suppressor genes, p53 and p21, in human ovarian cancer SKOV3 cells [324].

Silibinin has been widely used as anti-cancer drug in vitro and in vivo, and its combination with other therapies is a promising treatment for cancer, so clinical trials are needed to confirm its safety and efficacy in humans, and to develop as an anti-cancer drug.

### Emodin

Emodin (Fig. 2) is an anthraquinone derivative isolated from many plants including *Rheum palmatum*, *Polygonum cuspidatum*, *Polygonum multiflorum*, and *Cassia obtusifolia*. It exhibits remarkable biological effects such as anti-inflammation, anti-oxidant, prevention of intrahepatic fat accumulation and DNA damage [360–366]. Many studies have shown that emodin can attenuate numerous cancers including nasopharyngeal, gall bladder, lung, liver, colorectal, oral, ovarian, bladder, prostate, breast, stomach and pancreatic cancers, through the inhibition of cell proliferation and growth, metastasis, angiogenesis, and induction of apoptosis [367–379].

Emodin suppresses ATP-induced cell proliferation and migration through inhibiting NF-κB activation in human NSCLC A549 cells [380], and induces apoptosis through cell cycle arrest and ROS production in human hepatocellular carcinoma HepaRG cells [381]. It also induces autophagy to mediate apoptosis through ROS production in human colorectal cancer HCT-116 cells [382]. Moreover, emodin can inhibit tumor growth and metastasis in triple negative breast cancer cells, and human colorectal cancer HCT-116 cells [383, 384], whilst it suppresses cell migration and invasion through microRNA-1271 up-regulation in human pancreatic cancer SW1990 cells [385]. In addition, emodin can also inhibit angiogenesis in thyroid and pancreatic cancers [386–388].

Emodin exerts anti-cancer effects through various mechanisms. It effectively suppresses cell proliferation through inhibiting estrogen receptor α (ERα) genomic and PI3K/Akt non-genomic pathways in human breast cancer MCF-7 and MDA-MB-231 cells [389]. Besides, mitochondria and ER stress also play an important role in mediating emodin-induced anti-cancer effects [381, 390–392]. Emodin induces apoptosis through the loss of mitochondrial membrane potential, modulation of Bcl-2 family proteins, and caspase activation in human colorectal cancer CoCa cells and hepatocellular carcinoma HepaRG cells [381, 390]. ER stress is activated in emodin-treated human osteosarcoma U2OS cells, and emodin-induced apoptosis is suppressed by ER stress inhibition with 4-phenylbutyrate (4-PBA) in human NSCLC A549 and H1299 cells [391, 393].

Emodin has immunomodulatory effects in cancer and immunity. It inhibits cell growth and metastasis through blocking the tumor-promoting feed forward loop between macrophages and breast cancer cells [394]. It also down-regulates CXCR4 to suppress C–X–C motif chemokine 12 (CXCL-12)-induced cell migration and invasion in hepatocellular carcinoma HepG2 and HepG3 cells [395]. In addition, emodin inhibits the differentiation of maturation of DCs [396], and can modulate macrophage polarization to restore macrophage homeostasis [397].

Aloe-emodin is a derivate of emodin, which exhibits superior bioactivities in some cancers. It can inhibit cell proliferation through caspase-3 and caspase-9 activation in human oral squamous cell carcinoma SCC-15 cells [398], and induce apoptosis in human cervical cancer HeLa and SiHa cells, which is associated with glucose metabolism [399]. Another derivative of emodin, rhein, can also induce apoptosis in human pancreatic cancer Panc-1 cells, and inhibit tumor growth in pancreatic cancer xenograft mice [400]. It also inhibits cell migration and invasion through regulating Rac1/ROS/MAPK/AP-1 signaling pathway in human ovarian cancer SKOV3-PM4 cells [401].

The combination of emodin and other chemotherapies is widely used for cancer treatment. Emodin can promote the anti-tumor effects of gemcitabine in pancreatic cancer [402–404]. It enhances apoptosis in human pancreatic cancer SW1990 cells, and further inhibits tumor growth in SW1990 xenograft mice, through suppressing NF-κB pathway [402, 403]. The combination of emodin and curcumin can also enhance the inhibition of cell proliferation, survival, and invasion in human breast cancer MDA-MB-231, MDA-MB-435 and 184A1 cells [64]. Moreover, emodin enhances cisplatin-induced cytotoxicity through ROS production and multi-drug resistance-associated protein 1 (MRP1) down-regulation in human bladder cancer T24 and J82 cells [405].

Emodin has been shown to have remarkable anti-cancer effects in vitro and in vivo, and its combination with other therapies is very effective in treating cancer, therefore it is important to evaluate the safety and efficacy of emodin as an anti-cancer drug as the next step.

### Triptolide

Triptolide (Fig. 2) is a natural constituent derived from the root of a traditional Chinese medicine, *Tripterygium wilfordii* Hook. F., which possesses diverse effects including anti-inflammatory, anti-oxidative, and anti-cancer activities [60, 406, 407]. For cancer therapy, it has been used to treat breast, lung, bladder, liver, colorectal, pancreatic, ovarian, stomach, prostate, cervical, and oral cancers, melanoma, myeloma, leukemia, neuroblastoma, osteosarcoma, lymphoma, renal, nasopharyngeal, and endometrial carcinoma, through apoptosis, cell cycle arrest, inhibition of cell proliferation, metastasis and angiogenesis [406, 408–426].

Various effects have been disclosed as key contributions to the anti-cancer effects of triptolide. Triptolide is shown to exhibit pro-apoptosis effects in various cancers [427–431]. It induces mitochondrial apoptotic pathway to mediate apoptosis in Burkitt’s lumphoma Raji, NAMALWA and Daudi cells, and inhibits tumor growth in Daudi xenograft mice [432], and inhibits cell proliferation through microRNA-181a up-regulation in human neuroblastoma SH-SY5Y cells [433]. Moreover, triptolide induces autophagy to induce apoptosis and inhibit angiogenesis in human osteosarcoma MG63 cells, and breast cancer MCF-7 cells [431, 434]. In contrast, triptolide induces protective autophagy through calcium (Ca^2+^)/calmodulin-dependent protein kinase kinase β (CaMKKβ)-AMPK pathway in human prostate cancer PC-3, LNCaP and C4-2 cells, and through Akt/mTOR down-regulation in human cervical SiHa cells [420, 435]. Therefore, autophagy plays a dual role in triptolide-induced anti-cancer effects. In addition, triptolide is able to inhibit cell migration and invasion in human prostate cancer PC-3 and DU-145 cells, and in tongue squamous cell carcinoma SAS cells co-inoculated with human monocytes U937 cells [417, 419]. Furthermore, triptolide also possesses anti-angiogenic effect by inhibiting VEGFA expression in human breast cancer MDA-MB-231 and Hs578T cells, and through COX-2 and VEGF down-regulation in human pancreatic cancer Panc-1 cells [436, 437].

Triptolide is a natural substance, which exerts its anti-cancer effects through multiple targets. Triptolide is shown to induce mitochondrial-mediated apoptosis in various cancer cells, through decreased mitochondrial membrane potential, Bax and cytochrome c accumulation, PARP and caspase-3 activation, decreased ATP levels, and Bcl-2 down-regulation [432, 438–441]. Moreover, ERK is also shown to be important in mediating triptolide-induced anti-cancer activities. Triptolide induces apoptosis through ERK activation in human breast cancer MDA-MB-231 and MCF-7 cells [434, 442], and ERK activation leads to caspase activation, Bax up-regulation and Bcl-xL down-regulation [442]. On the other hand, it can also inhibit metastasis through ERK down-regulation in esophageal squamous cell cancer KYSE180 and KYSE150 cells, and murine melanoma B16F10 cells [443, 444]. Interestingly, ERα is shown to be a potential binding protein of triptolide and its analogues [445]. In addition, triptolide-induced metastasis is shown to be through MMP-2 and MMP-9 down-regulation in human neuroblastoma SH-SY5Y cells, via decreased MMP-3 and MMP-9 expressions in T-cell lymphoblastic lymphoma cells, and through MMP-2, MMP-7 and MMP-9 down-regulation in human prostate cancer PC-3 and DU-145 cells [417, 423, 433].

Indeed, immunology has been frequently validated to be associated with cancer. The combined use of triptolide and cisplatin enhances the plasma levels of IL-2 and TNF-α in ovarian cancer SKOV3/DDP xenograft mice, which can promote the differentiation of T cells and inhibit tumorigenesis respectively, thus resulting in an inflammatory microenvironment and leading to cancer cell death [446].

The derivatives of triptolide are always needed to improve its ant-cancer therapy. Triptolide derivative, MRx102, shows positive effects on anti-proliferation and anti-metastasis through Wnt inhibition in human NSCLC H460 and A549 cells, and H460 xenograft mice [447]. Minnelide, a water-soluble pro-drug of triptolide, can inhibit tumor growth in pancreatic cancer MIA PaCa-2 xenograft mice. Meanwhile, the combination of minnelide and oxaliplatin further inhibits tumor growth [448]. Moreover, triptolide is poorly soluble in water and exhibits hepatotoxicity and nephrotoxicity, selective delivery is an effective strategy for further application in cancer treatment. Triptolide loaded onto a peptide fragment (TPS-PF-A_299–585_) is specifically targeted to the kidney and with less toxicity [449]. Some modified triptolide-loaded liposomes are reported to contribute a targeted delivery with lower toxicity and better efficacy in lung cancer treatment [450]. Similarly, triptolide-loaded exosomes enhances apoptosis in human ovarian cancer SKOV3 cells [451].

Triptolide has some side effects in various organs because of excessive dosage, so researchers have been looking for alternative triptolide therapies, and combination therapy has become a hot spot. Triptolide combined with gemcitabine markedly enhances pro-apoptosis through Akt/glycogen synthase kinase 3β (GSK3β) pathway in human bladder cancer EJ and UMUC3 cells [452]. Triptolide plus ionizing radiation synergistically enhances apoptosis and anti-angiogenic effects through NF-κB p65 down-regulation in human nasopharyngeal carcinoma cells and xenograft mice, which provides a new chemotherapy to advanced nasopharyngeal malignancy [425]. The combined therapy of triptolide and 5-fluorouracil further promotes apoptosis and inhibits tumor growth through down-regulating vimentin in human pancreatic cancer AsPC-1 cells and AsPC-1 xenograft mice [453]. Besides, low concentration of triptolide potentiates cisplatin-induced apoptosis in human lung cancer HTB-182, A549 and CRL-5810 and CRL-5922 cells [454], and triptolide with cisplatin synergistically enhances apoptosis and induces cell cycle arrest in human bladder cancer cisplatin-resistant cells [409].

Triptolide has wide-spectrum activities in pre-clinical studies, but it has strong side effects and water insolubility, so it is not used in clinical studies. However, some of its derivatives and analogs have been used in clinical studies to test the safety and efficacy on anti-cancer effects [432, 455–457]. Omtriptolide, a derivative of triptolide, is highly water soluble, and a phase I clinical trial was conducted in Europe with patients who had refractory and relapsed acute leukemia [432]. Another phase I clinical trial was completed in patients with refractory gastrointestinal malignancies to study the dose escalation and pharmacokinectics of minnelide, a pro-drug of triptolide [457]. The doses used were 0.16 to 0.8 mg/m^2^ and they were well tolerated except from the common hematologic toxicity. LLDT-8, another triptolide derivative, has anti-cancer and immunosuppressive effects, and is going to proceed into phase II clinical trial to test its anti-cancer effects in China [455, 456]. Moreover, minnelide is currently under phase II clinical trial to test anti-cancer effects in patients with advanced pancreatic cancer [458].

### Cucurbitacins

Cucurbitacins (Fig. 2) is a cluster of tetracyclic triterpenoids originated from various plants like *Bryonia, Cucumis, Cucurbita and Lepidium sativum*. Cucurbitacins A–T are twelve main curcurbitacins belonging to this family. Cucurbitacins have multiple therapeutic effects such as anti-inflammation, anti-proliferation, anti-angiogenesis, and anti-cancer [452, 459–462]. Besides, cucurbitacins have also been elucidated as a potential candidate for various cancer therapies, including oral cell carcinoma, breast, ovarian, prostate, lung, gastric, bladder, and thyroid cancers, neuroastoma, hepatoma, and osteosarcoma [463–475]. Most of cucurbitacins have been reported with various anti-cancer activities, such as pro-apoptosis, anti-angiogenesis, autophagy induction, and inhibition of metastasis [452, 460–462, 476].

Cucurbitacin B is the most abundant source of cucurbitacins which can explain why it receives more attention from researchers than other cucurbitacins do. It suppresses cell proliferation and enhances apoptosis in human NSCLC A549 cells, colorectal cancer SW-480 and Caco-2 cells [462, 477], and induces G1 phase cell cycle arrest in human colorectal cancer SW-480 and Caco-2, and gastric cancer MKN45 cells [477, 478]. Cucurbitacin D inhibits cell survival in human gastric cancer AGS, SNU1 and Hs746T cells [479], while cucurbitacin E induces cell cycle arrest at G2/M phase in triple negative breast cancer cells [480]. Moreover, cucurbitacins B, E and I are shown to induce autophagy, however inhibition of autophagy can enhance cucurbitacin-induced apoptosis [481–483]. They also inhibit cell migration and invasion in human breast cancer MDA-MB-231 and SKBR3, NSCLC H2030-BrM3 and PC9-BrM3, and colorectal cancer COLO-205 cells [484–487], as well as angiogenesis in HUVECs [461, 488].

Various targets have been demonstrated to be responsible for the anti-cancer effects of cucurbitacins. STAT3 signaling is a very common target for cancer treatment. Cucurbitacins B and D are reported to inhibit proliferation and induce apoptosis through STAT3 suppression in human NSCLC A549 cells and doxorubicin-resistant breast cancer MCF-7/ADR cells, respectively [462, 489]. On the other hand, cucurbitacin E induces cell arrest and apoptosis via STAT3 inhibition in human breast cancer Bcap-37 and MDA-MB-231 cells [468], and cucurbitacin I can inhibit STAT3 pathway to suppress cancer stem cell properties in anaplastic thyroid cancer ATC–CD133^+^ cells [463]. Besides, cucurbitacin E induces cell cycle arrest through cyclins B1 and D1 down-regulation [480, 490], while cucurbitacin D inhibits cyclin B expression [491]. Moreover, mitochondria and ER stress also play an important role in cucurbitacin-induced anti-cancer effects. Cucurbitacins mediate apoptosis through mitochondrial-related pathway, which is characterized by the loss of the mitochondrial membrane potential, Bcl-2 down-regulation, Bax up-regulation, cytochrome c release, that eventually leads to caspase activation [470, 492]. Cucurbitacin I induces cell death through ER stress, by up-regulating ER stress markers such as IRE1α and PERK in human ovarian cancer SKOV3 cells and pancreatic cancer Panc-1 cells [493].

Cancer immunotherapy also plays a vital role in cucurbitacin treatment. Cucurbitacins may influence the production of cytokines and transcription factors that suppress the immune system, and these mechanisms may help to prevent the development of cancer. Cucurbitacin B is able to promote DC differentiation and anti-tumor immunity in patients with lung cancer [494]. The combined therapy of cucurbitacin I and recombinant IL-15 is also reported to exhibit immunologic anti-cancer activities in lymphoma with increased CD4^+^ and CD8^+^ T cell differentiation, and promote DC function through TNF-α up-regulation [495].

Although cucurbitacin B has very effective anti-tumor effects, it is shown to exhibit high toxicity, which restricts its clinical application on cancer therapy. Therefore, studies have been focused on tackling this side effect, and some cucurbitacin B derivatives have been synthesized to screen for effective cancer therapy with safety and tolerability. Compound 10b, one of the derivatives of cucurbitacin B, shows more potent anti-cancer activity than cucurbitacin B [496]. The in vivo acute toxicity study also shows that compound 10b has better tolerability and safety than cucurbitacin B. In addition, some other strategies have been applied to accelerate the clinical use of cucurbitacin B. The collagen peptide-modified nanomicelles with cucurbitacin B were synthesized to enhance the oral availability of cucurbitacin B, and these nanomicelles show a higher bioavailability and better tumor inhibition [497].

For a better cancer therapy, some combinations between cucurbitacins and other drugs have been employed. Low doses of cucurbitacin B or methotrexate cannot inhibit tumor growth in osteosarcoma xenograft mice, however when combined together, they synergistically inhibit tumor growth [498]. The combination therapy of cucurbitacin B and curcumin enhances apoptosis and reverses MDR in human hepatocellular carcinoma Bel-7402/5-Fu cells [499]. Recently, cucurbitacin B is suggested to be a potential candidate when it is applied with withanone, this combination can enhance cytotoxicity in human NSCLC A549 cells, and inhibit tumor growth and metastasis in A549 xenograft mice [500]. Cucurbitacin I is also shown to be a STAT3 inhibitor to mediate cell survival and proliferation, and when it is combined with irinotecan, and they further inhibit cell proliferation in human colorectal cancer SW-620 and LS174T cells [501].

The derivatives of cucurbitacins, cucurbitacin B-nanomicelles, and the combination therapies show promising treatment for cancer in vitro and in vivo, so clinical trials are needed to confirm their safety and efficacy in cancer treatment.

### Tanshinones

Tanshinone (Fig. 2) is a derivative of phenanthrenequinone isolated from the dried root or rhizomes of *Salvia miltiorrhiza* Bunge. Tanshinone IIA is the primary bioactive constituent of tanshinones [502], which has various pharmacological effects, including anti-inflammatory, anti-cancer and anti-atherosclerotic activities, and cardiovascular protection [503–506]. Tanshinone exhibits anti-cancer activities in stomach, prostate, lung, breast, and colon cancers, through inducing cell cycle arrest, apoptosis, autophagy, and inhibiting cell migration [507–515].

Tanshinone IIA suppresses cell proliferation and apoptosis in numerous cancer cells, including human breast cancer BT-20, MDA-MB-453, SKBR3, BT-474, MCF-7 and MD-MB-231 [508, 516, 517], and gastric cancer MKN45 and SGC-7901 cells [518]. It also induces cell cycle arrest at G1 phase in human breast cancer BT-20 cells [517], and inhibits cell migration in human gastric cancer SGC-7901 cells [514], and cell migration and invasion in cervix carcinoma stemness-likes cells [519]. Tanshinone I and cryptotanshinone are two other major bioactive compounds, which also induce cytotoxicity against cancer cells. Tanshinone I induces apoptosis and pro-survival autophagy in human gastric cancer BGC-823 and SGC-7901 cells [510], while cryptotanshinone suppresses cell proliferation and induces cell cycle arrest at G1 phase in murine melanoma B16 cells, and G2/M phase in melanoma B16BL6 cells [520]. In addition, tanshinones I and IIA and cryptotanshinone also inhibit tumor angiogenesis in endothelial and cancer cells [521–525]. Furthermore, tanshinone IIA induces autophagy to inhibit cell growth in human osteosarcoma 143B and MG63 cells and tumor growth in NOD/SCID mice [526], while it induces autophagy to mediate anti-cancer activities through activating beclin-1 pathway and inhibiting PI3K/Akt/mTOR pathway in human oral squamous cell carcinoma SCC-9, melanoma A375, and glioma U251 cells [527–529]. Moreover, tanshinone IIA is shown to exhibit anti-cancer activities through the interplay between autophagy and apoptosis in human prostate cancer PC-3 cells, mesothelioma H28 and H2452 cells [502, 530].

Tanshinone IIA induces apoptosis through mitochondrial- and caspase-dependent pathways, which includes caspase-3, -9 and PARP activation, cytochrome c release, and increased ratio of Bax/Bcl-2 in human gastric cancer MKN45 and SGC-7901 cells, and tumor-bearing mice [518]. It inhibits epithelial–mesenchymal transition by modulating STAT3-chemokine (C–C motif) ligand 2 (CCL2) pathway in human bladder cancer 5637, BFTC and T24 cells [531], and suppresses cell proliferation and migration via forkhead box protein M1 (FoxM1) down-regulation in human gastric cancer SGC-7901 cells [514]. On the other hand, tanshinone I induces apoptosis via Bcl-2 down-regulation in human gastric cancer BGC-823 and SGC-7901 cells [510], while cryptotanshinone induces apoptosis through mitochondrial-, cyclin- and caspase-dependent pathways in human NSCLC A549 and NCI-H460 cells [532], as well as via ER stress in human hepatocellular carcinoma HepG2 and breast cancer MCF-7 cells [533].

Tanshinone IIA is also shown to exhibit immunomdulatory effects in cancer [534]. The combination of tanshinone IIA with cyclophosphamide increases CD4^+^ T cell, CD4^+^/CD8^+^ T cell and NK cell populations compared to single treatment in NSCLC Lewis-bearing mice, so it can improve the immunological function in lung cancer [534]. Furthermore, cryptotanshinone becomes a new promising anti-tumor immunotherapeutic agent [535]. It induces mouse DC maturation and stimulates IL-1β, TNF-α, IL-12p70 secretion in DCs, and enhances T cell infiltration and Th1 polarization in Lewis-bearing tumor tissues [535].

Tanshinone IIA has poor bioavailability, so a mixed micelle system is developed to form a tanshinone-encapsulated micelle [536]. This micelle has higher cytotoxicity and pro-apoptotic effects in human hepatocellular carcinoma HepG2 cells compared to tanshinone IIA alone. The tanshinone IIA-loaded nanoparticles improve the bioavailability tanshinone IIA and enhance its leukemic activity in human leukemia NB4 cells [537], while the nanoparticles containing tanshinone IIA and α-mangostin show increased cytotoxicity in human prostate cancer PC-3 and DU-145 cells [538].

Tanshinone IIA is shown to enhance chemosensitivity and its efficacy when combined with other therapeutic agents. Tanshinone IIA can be an effective adjunctive agent in cancer, and it enhances the chemosensitivity to 5-fluorouracil therapy in human colorectal cancer HCT-1116 and COLO-205 cells through NF-κB inhibition [539]. The combination of tanshinone IIA with doxorubicin does not only enhance the chemosensitivity of doxorubicin, but also reduces the toxic side effects of doxorubicin in human breast cancer MCF-7 cells [540]. In addition, tanshinone IIA and cryptotanshinone synergistically enhance apoptosis in human leukemia K562 cells [541].

The anti-cancer effects of Tanshinone IIA have been demonstrated in various cancers in vitro and in vivo, and it can enhance chemosensitivity and its efficacy is very effective when combined with other therapeutic agents. Up to now, the clinical trials of Tanshinone IIA are completed only for the treatment of other diseases [542], so well-designed clinical trials should be done to further confirm its safety and efficacy in cancer treatment.

### Oridonin

Oridonin (Fig. 2) is an ent-kaurane diterpenoid isolated from *Rabdosia rubescens* (Hemsl.) Hara, which is also the main active constituent of *Rabdosia rubescens* (Hemsl.) Hara [543]. As an orally available drug, oridonin is demonstrated to have anti-cancer activities in multiple cancers over the past decades, including leukemia, lymphoma, osteosarcoma, myeloma, uveal melanoma, neuroblastoma, hepatocellular, laryngeal, esophageal, and oral squamous cell carcinoma, lung, colorectal, breast, gastric, pancreatic, and prostatic cancers [543–558]. The anti-cancer effects of oridonin are shown in many aspects, including the induction of cell apoptosis, autophagy, cell cycle arrest, and the suppression of angiogenesis, cell migration, invasion and adhesion [554, 559–564].

Oridonin induces apoptosis in human hepatocellular carcinoma HepG2 and Huh6, oral squamous cell carcinoma WSU-HN4, WSU-HN6 and CAL27, and laryngeal cancer HEp-2 cells [550, 559, 561, 565]. It also induces G2/M cell cycle arrest in human oral squamous cell carcinoma WSU-HN4, WSU-HN6 and CAL27, gastric cancer SGC-7901, prostate cancer PC-3 and DU-145, and breast cancer MCF-7 cells [555, 561, 566, 567]. Oridonin is also shown to induce autophagy in many cancer cells, which is associated positively or negatively with apoptosis. It induces autophagy to mediate apoptosis in human NSCLC A549 and neuroblastoma SHSY-5Y cells [558, 568]. On the other hand, autophagy provides a protective role against oridonin-induced apoptosis, as autophagy inhibitor enhances oridonin-induced apoptosis in human cervical carcinoma HeLa, multiple myeloma RPMI 8266, laryngeal cancer HEp-2 and Tu212, and epidermoid carcinoma A-431 cells [569–572]. The anti-cancer effects of oridonin are also shown to be through suppressing angiogenesis and metastasis, which are the primary causes of tumor growth and metastasis. It can inhibit cell migration and invasion, and tube formation in human breast cancer 4T1 and MDA-MB-231, human and murine melanoma A375 and B16F10, osteosarcoma MG63 and 143B, and HUVECs, as well as tumor metastasis in HepG2 xenograft zebrafish and mice, 4T1 xenograft mice, and 143B xenograft mice [554, 562–564, 573].

Proteomic and functional analyses reveal that ER stress and poly(rC)-binding protein 1 (α-CP1) are potential pathways involved in the anti-proliferative and pro-apoptotic activities of oridonin [546]. Oridonin inhibits cell growth and induces apoptosis through ER stress and ASK1/JNK signaling pathways in human hepatocellular carcinoma Huh6 cells [559]. Besides, the mitochondrial redox change is proved to be a potential mediator for the pro-apoptosis effect of oridonin [565]. The anti-proliferative effect of oridonin is also shown to be associated with mitochondrial-mediated apoptosis, which is characterized by mitochondrial membrane potential reduction, subsequent cytochrome c release, PARP, caspase-3 and -9 activation, and decreased Bcl-2/Bax ratio [551, 565, 574, 575]. Oridonin also inhibits cell proliferation through bone morphogenetic protein 7 (BMP7)/p38 MAPK/p53 pathway in human colorectal cancer HCT-116 and SW-620 cells [553, 576, 577], and induces apoptosis via hydrogen peroxide (H_2_O_2_) production and glutathione depletion in human colorectal cancer SW-1116 cells [578]. Furthermore, the down-regulation of *AP*-*1* is reported to be the initial response to oridonin treatment, which decreases the expressions of NF-κB and MAPK to inhibit cell proliferation [579].

Oridonin possesses an immunosuppressive effect which modulates microglia activation, enhances T cell proliferation, alters the balance of Th1-T helper type 2 cells (Th2), reduces inflammatory cytokine secretion such as IL-2, IL-4, IL-6, IL-10 and TNF-α, and modulates an anti-inflammatory target, B lymphocyte stimulator [580]. It also decreases inflammatory cytokine secretion in human pancreatic cancer BxPC-3 cells, including IL-1β, IL-6 and IL-33 [581].

The derivatives and analogs of oridonin usually exhibit more potent anti-cancer activities than oridonin. Geridonin, a novel derivative of oridonin, inhibits cell growth and induces G2/M phase arrest through ROS production in human gastric cancer MGC-803 cells and MGC-803 xenograft mice [582]. Oridonin phosphate, another derivative, is reported to induce autophagy, which can enhance apoptosis in human breast cancer MDA-MB-436 cells [583]. A novel analog of oridonin, CYD 6-17, inhibits tumor growth in bladder cancer UMUC3 xenograft mice and renal carcinoma 786-O xenograft mice [584, 585]. In addition, drug delivery system is also developed to improve the bioavailability of oridonin. The inhalable oridonin-loaded microparticles exhibit strong pro-apoptotic and anti-angiogenic effects through mitochondrial-related pathways in NSCLC rats [586], whilst the oridonin-loaded nanoparticles enhance cellular uptake and exert better anti-cancer effects in human hepatocellular carcinoma HepG2 cells [587].

The combination of oridonin with other agents plays a potential role in cancer therapy. AG1478, a specific epidermal growth factor receptor (EGFR) inhibitor, augments oridonin-induced apoptosis through oxidative stress and mitochondrial pathways in human epidermoid carcinoma A-431 cells [588]. The combination of γ-tocotrienol and oridonin exerts synergistic anti-cancer effects in murine + SA mammary adenocarcinoma epithelial cells, which are mainly through the induction of autophagy [589]. Moreover, oridonin can enhance the pro-apoptotic activity of NVP-BEZ235 in human neuroblastoma SHSY-5Y and SK-N-MC cells through autophagy [558], whilst the combination of oridonin and cetuximab exhibits potent pro-apoptotic effect in human laryngeal cancer HEp-2 and Tu212 cells [572].

Clinical trials are essential to test the safety and efficacy of oridonin before drug approval. A derivative of oridonin, HAO472, is currently under a phase I clinical trial for the treatment of acute myelogenous leukemia in China [590].

### Shikonin

Shikonin (Fig. 2) is an active naphthoquinone, which is derived from the dried root of *Lithospermum erythrorhizon*, *Arnebia euchroma* and *Arnebia guttata*, and it possesses anti-oxidative, anti-inflammatory, and anti-cancer activities [591–594]. It is effective in treating different kinds of cancers, including breast, prostate, ovarian and thyroid cancers, Ewing sarcoma, and myelomonocytic lymphoma [595–600]. Shikonin exerts anti-cancer effects mainly by inducing apoptosis, necroptosis, autophagy, cell cycle arrest, and by inhibiting cell proliferation, growth and metastasis [593, 601, 602].

Shikonin is reported to inhibit cell growth by inducing cell cycle arrest and promoting apoptosis in human NSCLC A549, gallbladder cancer NOZ and EHGB-1, esophageal cancer EC109, and epidermoid carcinoma A-431 cells [601, 603–605]. It can also induce necroptosis via autophagy inhibition in human NSCLC A549 cells [593], and through ROS overproduction in human nasopharyngeal carcinoma 5-8F, and glioma SHG-44, U87 and U251 cells [606, 607]. Moreover, shikonin induces autophagy in human melanoma A375, pancreatic cancer BxPC-3, and hepatocellular carcinoma Bel-7402 and Huh7 cells [608–610]. However, autophagy provides a protective role in shikonin-induced apoptosis in human melanoma A375 cells [608]. In addition, shikonin can suppress metastasis by the inhibition of tyrosine kinase c-Met and integrin (ITG) β1 in human NSCLC A549 cells [602, 611].

There are multiple mechanisms involved in the anti-cancer effects of shikonin, including ER stress, ROS generation, glutathione (GSH) depletion, mitochondrial membrane potential disruption, p53, superoxide dismutase (SOD) and Bax up-regulation, PARP cleavage, catalase and Bcl-2 down-regulation [591, 612–614]. The pro-apoptotic effect of shikonin is also caused by the disruption of intracellular Ca^2+^ homeostasis and mitochondrial dysfunction, which involves enhanced Ca^2+^ and potassium (K^+^) efflux, caspase-3, -8 and -9 activation, and Bcl-2 family protein modulation [615, 616]. ERK pathway also plays a role in shikonin-induced anti-cancer effects. Shikonin induces apoptosis and inhibits metastasis through suppressing ERK pathway in human NSCLC NCI-H460 and A549 cells, respectively [611, 617]. c-Myc down-regulation along with inhibition of ERK/JNK/MAPK and Akt pathways are also involved in shikonin-induced apoptosis and anti-proliferation in acute and chronic leukemia [618–620]. Moreover, the activation of necroptosis initiators, receptor interacting serine-threonine protein kinase (RIP) 1 and RIP3, by shikonin does not only contribute to DNA double strand breaks via ROS overproduction [621], but also facilitates glycolysis suppression via intracellular H_2_O_2_ production [622]. In addition, shikonin induces cell cycle arrest through p21 and p27 up-regulation, cyclin and CDK down-regulation [605]. Therefore, numerous pathways involved in shikonin-induced anti-cancer effects may explain the broad range of its activities.

Shikonin is also shown to modulate the function of the immune system. It can enhance the proliferation of NK cells and its cytotoxicity to human colorectal cancer Caco-2 cells by regulating ERK1/2 and Akt expressions [623]. It can also bind directly to heterogeneous nuclear ribonucleoprotein A1 to induce immunogenic cell death in human breast cancer MDA-MB-231 cells [624]. Shikonin is also reported to be used as an immunotherapy modifier in cell-based cancer vaccine systems, suggesting its potential application in cancer immunotherapy [625].

Derivatives are developed to enhance the anti-cancer and tumor targeting effects of shikonin. The naphthazarin ring of shikonin is modified to produce DMAKO-05, which can specifically target cancer cells instead of normal cells [626]. DMAKO-05 can also suppress cell survival in human colorectal cancer HCT-116 cells, and inhibits tumor growth in colorectal cancer CT-26 xenograft mice [627]. Besides, it inhibits cell proliferation and migration, and induces cell cycle arrest and apoptosis in murine melanoma B16F0 cells [626]. Another novel shikonin derivative, cyclopropylacetylshikonin, exhibits strong anti-tumor and pro-apoptotic effects in human melanoma WM164 and MUG-MEL2 cells [628]. In addition, drug delivery system is also developed to promote the intracellular delivery of shikonin. The shikonin-loaded nanogel enhances RIP1- and RIP3-dependent necroptosis in human osteosarcoma 143B cells [629]. There is an increased accumulation of shikonin-loaded nanogel in the tumor tissue, and this nanogel can further inhibit tumor growth and metastasis in 143B xenograft mice. Furthermore, the modified shikonin-loaded liposomes have higher cytotoxicity, and inhibit cell proliferation, metastasis in human breast cancer MDA-MB-231 cells [630].

The combination therapy is widely used to provide synergistic effects of anti-cancer activities. Shikonin can enhance the pro-apoptotic effect of taxol in human breast cancer MBA-MD-231 cells, and this combination improves mice survival and inhibits tumor growth in MDA-MB-231 xenograft mice [631]. Besides, shikonin can also potentiate the anti-cancer effects of gemcitabine through NF-kB suppression and by regulating RIP1 and RIP3 expressions in human pancreatic cancer [632, 633]. Shikonin is also reported to promote the efficacy of adriamycin in lung cancer and osteosarcoma [634, 635], and enhance sensitization to cisplatin in colorectal cancer [636]. Apart from the synergistic effect of shikonin, the combination of shikonin and paclitaxel reverses MDR in human ovarian cancer A2780 cells [10].

The single or combined therapies with shikonin show promising anti-cancer effects in vitro and in vivo, so pre-clinical data has confirmed its therapeutic use in cancer treatment, as a result, clinical trials will be carried out to further to confirm its safety and efficacy in humans.

### Gambogic acid (GA)

GA (Fig. 2) is one of the major compounds derived from gambogethe resin exuded from Garcinia species including *G. hanburyi* and *G. Morella* [637]. It has multiple biological activities such as anti-oxidative, anti-inflammatory, and anti-cancer activities [638, 639]. Plenty of evidence shows that GA inhibits cell proliferation, invasion, survival, metastasis and chemo-resistance, and induces angiogenesis in many types of cancers such as gastric and prostate cancers, leukemia, multiple myeloma, osteosarcoma, and renal carcinoma through multiple signaling mechanisms [640–646].

Many studies have reported the anti-cancer effects of GA in human breast cancer [647–650]. GA at low concentrations (0.3–1.2 μM) can inhibit cell invasion without affecting cell viability, while high concentrations of GA (3 and 6 μM) can induce apoptosis via ROS accumulation and mitochondrial apoptotic pathway in human breast cancer MDA-MB-231 cells [651]. GA also induces apoptosis via ROS production in human bladder T24 and UMUC3 cells [652]. At earlier time points, GA induces ROS-mediated autophagy, which produces a strong cell survival response. However, at later time points, caspases are activated which degrade autophagic proteins and cell survival proteins, and this eventually induces apoptosis. Similarly, GA-induced autophagy via ROS provides a cytoprotective effect to human pancreatic cancer Panc-1 and BxPC-3 cells [653], and ROS scavenger, N-acetylcysteine, can reverse GA-induced autophagy in human NSCLC NCI-H441 cells [654]. Moreover, GA inhibits cell invasion and migration through reversion-inducing-cysteine-rich protein with kazal motifs (RECK) up-regulation in human NSCLC A549 cells and A549 xenograft mice [655], and prevents TNF-α-induced invasion in human prostate cancer PC-3 cells [656]. It also inhibits angiogenesis in HUVECs, and prevents tumor growth through the inhibition of tumor angiogenesis [657].

ROS-related pathways play a vital role in GA-induced cell death [642, 646, 647, 651–654, 658]. GA induces apoptosis mainly through ROS accumulation in human pancreatic cancer Panc-1 and BxPC-3, NSCLC NCI-H441, castration-resistant prostate cancer PCAP-1, melanoma A375, breast cancer MCF-7 cells [642, 646, 647, 653, 654]. It also induces oxidative stress-dependent caspase activation to mediate apoptosis in human bladder cancer T24 and UMUC3 cells [652]. Moreover, GA increases the expressions of ER stress markers such as GRP78, CHOP, activating transcription factor 6 (ATF-6) and caspase-12, and co-treatment with chemical chaperone, 4-PBA, significantly reduces these expressions and apoptosis in human NSCLC A549 cells, so it is suggested that GA induces ER stress to mediate apoptosis [659].

Previous studies have shown some immunomodulatory activities of GA [660, 661]. The activation of TLRs is important to initiate immune responses, and TLR4 forms a complex with myeloid differentiation factor 2 (MD2) to recognize its ligand, like LPS. GA is shown to reduce pro-inflammatory cytokine production in LPS-primed primary macrophages such as TNF-α, IL-1β, IL-6 and IL-12, and also inhibit the activation of TLR4 by disrupting the interaction of TLR4/MD2 complex with LPS [660]. Similarly, it also reduces pro-inflammatory cytokine production including TNF-α, IL-1β and IL-6 by suppressing p38 pathway in murine macrophage RAW 264.7 cells [661].

GA has low solubility, instability and poor pharmacokinetic properties [662]. In order to increase its water solubility, GA is conjugated with a cell-penetrating peptide, trans-activator of transcription, to form GA-TAT [658]. This GA-TAT enhances apoptosis through ROS accumulation in human bladder cancer EJ cells. Another study uses a co-polymer to encapsulate GA to form GA micelles [639]. These GA micelles have better cellular uptake which can further enhance apoptosis in human breast cancer MCF-7 cells and the anti-tumor effects in MCF-7 xenograft mice. Moreover, GA is encapsulated into the core of the nanoparticles to enhance the stability of GA and its circulation time [662]. These nanoparticles have tumor targeting properties, and enhance the anti-tumor activities of GA without inducing higher toxicity.

The combination of GA and other chemotherapy agents has been widely used to improve the therapeutic effects against various cancers such as osteosarcoma, pancreatic and lung cancers [639, 653, 663, 664]. Cisplatin resistance is a main clinical problem for the treatment of lung cancer, and the treatment of cisplatin with GA is shown to enhance apoptosis and decrease the cisplatin resistance index in human NSCLC cisplatin-resistance A549/DDP cells [663]. Moreover, GA and retinoic acid chlorochalcone are loaded into glycol chitosan nanoparticles to form RGNP [639]. The RGNP exhibits synergistic effects to inhibit cell proliferation and induces apoptosis in osteosarcoma. The combination of GA with doxorubicin synergistically reduces cell viability in human ovarian cancer platinum-resistance SKOV3 cells, and this combination also suppresses tumor growth in SKOV3 xenograft mice [665].

The safety and efficacy of GA at different dosages in patients with advanced malignant tumors have been compared in a phase IIa clinical trial [666]. GA had a safety profile at a dosage of 45 mg/m^2^. The patients with GA administration on days 1–5 in a 2-week cycle showed a greater disease control rate and only Grades I and II adverse reactions. To further investigate the safety and efficacy of GA, a phase IIb clinical trial involving a larger sample size of patients would be needed.

### Artesunate

Artesunate (Fig. 2) is a semi-synthetic compound derived from ART, which is widely used as an anti-malarial agent [667]. As an analog of ART, artesunate exerts better water solubility and higher oral bioavailability, due to its special structure with an additional hemisuccinate group that makes it a better candidate for cancer treatment [668]. The anti-cancer effects of artesunate have been demonstrated in bladder, breast, cervical, colorectal, esophageal, gastric, ovarian and prostate cancer, renal carcinoma, leukemia, melanoma and multiple myeloma [179, 669–679]. Its anti-cancer effects include induction of cell cycle arrest and apoptosis, inhibition of cell proliferation and growth, metastasis and angiogenesis [670, 678, 680].

Artesunate can induce apoptosis in various cancers including human breast cancer MCF-7, MDA-MB-468 and SKBR3 cells, gastric cancer SGC-7901 and HGC-27, colorectal cancer HCT-116, and esophageal cancer Eca109 and Ec9706 cells [670, 672, 673, 681–683]. It also induces cell cycle arrest at ROS-dependent G2/M phase and ROS-independent G1 phase in human breast cancer MDA-MB-468 and SKBR3, and ovarian cancer HEY1 and HEY2 cells [670, 684], and induces G2/M cell cycle arrest through autophagy in human breast cancer MCF-7 and MDA-MB-231 cells [685]. Artesunate is also shown to induce autophagy to exert cytoprotective effects in human colorectal cancer HCT-116 cells, and the inhibition of autophagy enhances artesunate-mediated apoptosis [179]. Similarly, artesunate-induced mitophagy provides a protective effects against cell death in human cervical cancer HeLa cells [686]. Moreover, it inhibits cell invasion and migration in human prostate cancer DU-145 and LNCaP, cervical cancer Caski and HeLa cells, and uveal melanoma cells [675, 678, 687], and suppresses tumor angiogenesis in HUVECs and renal carcinoma 786-O xenograft mice [676, 680].

In most cases, the inhibition effects of artesunate against cancer cells are resulted from apoptosis. Artesunate induces apoptosis through cyclooxygenase-2 (COX-2) down-regulation in human bladder cancer T24 and RT4, and gastric cancer HGC-27 cells [669, 683]. Mitochondrial pathways also play an important role in artesunate-mediated anti-cancer effects [673, 681, 683]. Artesunate inhibits tumor growth through ROS- and p38 MAPK-mediated apoptosis in human rhabdomyosarcoma TE671 cells [688]. It also exerts anti-tumor activities through the loss of mitochondrial membrane potential, Bcl-2 down-regulation, Bax up-regulation, and caspase-3 activation in human gastric cancer SGC-7901 and HGC-27, esophageal cancer Eca109 and Ec9706 cells, and breast cancer MCF-7 xenograft mice [673, 681, 683]. In addition, gene expression analysis identifies that ER stress is the most relevant pathway for the anti-tumor activity of artesunate in B-cell lymphoma [689]. Interestingly, artesunate selectively inhibits cell growth through iron-dependent and ROS-mediated ferroptosis in human head and neck cancer HN9 cells [690].

Immunomodulation also plays a vital role in artesunate-mediated anti-cancer effects [671, 674, 691, 692]. Artesunate induces Th1 differentiation into CD4^+^ T cells to mediate apoptosis in murine ovarian cancer ID8 cells [674]. It also exerts anti-tumor effects through suppressing NK killing activity and lymphocyte proliferation, which results in decreased TGF-β1 and IL-10 levels in colorectal cancer Colon-26 and RKO cells [691]. Besides, artesunate also exerts immunosuppression through forkhead box P3 (Foxp3) down-regulation in T cells and decreases prostaglandin E_2_ (PGE_2_) production in human cervical cancer Caski and HeLa cells [671]. Moreover, it enhances γδ T cell-mediated anti-cancer effect through augmenting γδ T cell cytotoxicity and decreasing TGF-β1 levels to reverse immune escape in human hepatocellular carcinoma HepG2 cells [692].

The treatment of artesunate with other therapies shows promising anti-cancer effects in several studies [693–697]. Artesunate and cisplatin synergistically induce DNA double-strand breaks and inhibit clonogenic formation to mediate cytotoxic effects in human ovarian cancer A2780 and HO8910 cells [693]. The combined treatment of artesunate and erlotinib enhances the inhibition of cell growth in human glioblastoma multiforme U87MG cells [694].

Clinical studies are carried out to investigate the safety and efficacy of artesunate in patients with colorectal and breast cancers, and advanced solid tumor malignancies [698–701]. A phase I study is performed to evaluate the safety and the maximum tolerated dose of artesunate in patients with metastatic breast cancer, the oral administration of artesunate is safe and 2.2–3.9 mg/kg per day is well tolerated [701]. Another phase I study is assessed in patients with advanced solid tumor malignancies, and the maximum tolerated dose of intravenous artesunate is 18 mg/kg [698]. The tolerability and anti-proliferative properties of oral artesunate are also shown in patients with colorectal cancer [699]. Moreover, a study of long term treatment with oral artesunate is performed in patients with metastatic breast cancer, 2.3–4.1 mg/kg per day treatment for up to 1115 cumulative days does not show any major safety concerns [700]. An ongoing phase II clinical trial is carried out to study the safety and effectiveness of neoadjuvant artesunate in patients with stage II or III colorectal cancer awaiting surgical treatment.

### Wogonin

Wogonin (Fig. 2) is a plant flavonoid extracted from roots of *Scutellaria baicalensis*, *Scutellaria amoena* and *Scutellaria rivularis,* and stem of *Anodendron affine* Druce, and has many pharmacological effects including anti-viral, anti-oxidative, anti-inflammatory, anti-cancer and neuro-protective activities [702–705]. It has various anti-cancer effects in many cancers, including lung, breast, head and neck, gastric and colorectal cancers, glioma, leukemia, lymphoma, and osteosarcoma, through the induction of apoptosis and cell cycle arrest, and inhibition of cell growth, migration, invasion, and angiogenesis [706–716].

Wogonin can induce apoptosis and inhibit cell proliferation in human neuroblastoma SK-N-BE2 and IMR-32, NSCLC A549, glioma U251 and U87, and hepatocellular carcinoma HepG2 and Bel-7402 cells [704, 706, 711, 717]. It also induces cell cycle arrest in human colorectal cancer HCT-116, NSCLC A549, chronic myelogenous leukemia imatinib-resistant K562, and ovarian cancer A2780 cells [716, 718–720]. Besides, wogonin induces autophagy in human pancreatic cells Panc-1 and Colo-357, and nasopharyngeal carcinoma NPC-TW076 and NPC-TW039 cells [721, 722]. However, inhibition of autophagy promotes wogonin-induced apoptosis in human nasopharyngeal carcinoma NPC-TW076 and NPC-TW039 cells [722]. It also inhibits metastasis in human hepatocellular carcinoma Bel-7402 and HepG2 cells, and NSCLC A549 cells [717, 723], and through MMP-9 suppression in human hepatocellular carcinoma MHCC97-L and PLC/PRF/5 cells [724]. In addition, wogonin also represses multiple myeloma-stimulated angiogenesis through c-Myc/von Hippel-Lindau tumor suppressor (VHL)/HIF-1α signaling pathway [725], LPS- and H_2_O_2_-induced angiogenesis through PI3K/Akt/NF-κB pathway [726, 727].

Mitochondrial dysfunction, oxidative stress and ER stress play important roles in wogonin-induced anti-cancer effects. Wogonin activates mitochondrial and ER stress-related pathways including the modulation of Bcl-2 family proteins, cytochrome c release, GRP78 and 94-kDa glucose-regulated protein (GRP94) accumulation, and caspase activation in human neuroblastoma SK-N-BE2 and IMR-32 cells, and induces mitochondrial dysfunction through IRE1α-dependent pathway [704]. ER stress markers and downstream pathways are also activated following wogonin treatment in human leukemia HL-60 and osteosarcoma U2OS cells, including IRE1α, PERK-eIF2α, ATF-6, CHOP, GRP94 and GRP78 [714, 728]. Wogonin also enhances ROS production in human glioma U251 and U87, pancreatic cancer Panc-1 and Colo-357, and NSCLC A549 cells [711, 721, 729]. Moreover, it inhibits cell growth and induces apoptosis through NF-κB suppression in Epstein–Barr virus-positive lymphoma cells [730], and suppresses cell proliferation and invasion through NF-κB/Bcl-2 and EGFR pathways in human hepatocellular carcinoma HepG2 and Bel-7402 cells [717].

Wogonin has immunomodulatory effects in cancer cells. It enhances the recruitment of DCs, T and NK cells into the tumor tissues in gastric cancer MFC xenograft mice, and also down-regulates the level of B7-H1, an immunoglobulin-like immune suppressive molecule, to promote anti-tumor immunity [731]. It also inhibits cell migration through modulating inflammatory microenvironment via IL-6/STAT3 pathway in human NSCLC A549 cells [723]. Moreover, immunization with wogonin-treated tumor cell vaccine effectively inhibits tumor growth in MFC xenograft mice [732]. Targeting TNF receptor with wogonin is also suggested to be a potential strategy for the treatment of chronic lymphocytic leukemia [712].

In order to enhance the accumulation and retention of wogonin in cancer cells, wogonin-conjugated Pt(IV) pro-drug is developed [733]. This pro-drug enhances the anti-proliferative and pro-apoptotic effects through casein kinase 2 (CK2)-mediated NF-κB pathway in human gastric cancer SGC-7901 and cisplatin-resistant SGC-7901/cDDP cells, and reverses cisplatin resistance in cisplatin-resistant SGC-7901/cDDP xenograft mice. It also further induces cell cycle arrest, enhances ROS production and apoptosis, and decreases mitochondrial membrane potential compared to wogonin in SGC-7901 cells [734]. LW-213, a derivative of wogonin, inhibits cell proliferation and induces cell cycle arrest in human breast cancer MCF-7 and MDA-MB-231 cells, and suppresses tumor growth in MCF-7 xenograft mice [735]. A synthetic wogonin derivative, GL-V9, inhibits metastasis in human breast cancer MDA-MB-231 and MCF-7 cells [736], and induces apoptosis and cell cycle arrest in human hepatocellular carcinoma HepG2 and gastric cancer cells MGC-803 cells [737–739]. Moreover, targeting cancer cells specifically is an important strategy in cancer therapy, so wogonin-loaded liposomes are synthesized [740]. These liposomes accumulate in the liver and prolong its retention time and exert better inhibitory effects than wogonin in human hepatocellular carcinoma HepG2 cells.

The combination therapy has been widely used to enhance the anti-cancer effects of wogonin. The combined treatment of wogonin and oxaliplatin synergistically inhibits cell growth in human gastric cancer BGC-823 cells and BGC-823 xenograft zebrafish, through nitrosative stress and disruption of mitochondrial membrane potential [741]. Wogonin also suppresses sorafenib-induced autophagy to exacerbate apoptosis in human hepatocellular carcinoma Hep3B and Bel-7402 cells [742], and augments cisplatin-induced apoptosis through H_2_O_2_ accumulation in human NSCLC A549 and cervical cancer HeLa cells [743].

As wogonin has various anti-cancer activities, it is currently under phase I clinical trial to test the safety and efficacy as an anti-cancer drug in China [734].

### β-Elemene

β**-**Elemene (Fig. 2) is a sesquiterpene mixture isolated from various Chinese herbs such as *Curcuma wenyujin* Y. H. Chen *et* C. Ling, *Rhizoma zedoariae*, and *Curcuma Zedoary*. It has various pharmacological effects including anti-oxidative, anti-inflammatory and anti-cancer activities [744–746]. It exerts anti-cancer effects in many cancers, such as lung, gastric, cervical, breast and bladder cancers, osteosarcoma, through apoptosis, inhibition of cell proliferation, migration and invasion, angiogenesis [746–752].

β-Elemene is shown to induce apoptosis in human cervical cancer SiHa, NSCLC A549 cells, primary bladder cancer cells, and Burkitt’s lumphoma, and inhibit tumor growth in Lewis tumor-bearing mice [746, 747, 749, 753, 754]. It up-regulates insulin-like growth factor-binding protein 1 (IGFBP1) to induce a reciprocal interaction between microRNA 155-5p and FoxO3a, which leads to the inhibition of cell growth in human NSCLC A549 and H1975 cells [755]. β-Elemene also induces S phase arrest in human NSCLC A549 cells [754], while it induces G0/G1 phase arrest in human glioblastoma U87 cells [756]. Moreover, it induces protective autophagy in human gastric cells MGC-803 and SGC-7901, and NSCLC A549 cells, as autophagy inhibition promotes β-elemene-induced anti-tumor effects [748, 757]. However, autophagy inhibition attenuates β-elemene-induced apoptosis in human NSCLC cisplatin-resistant SPC-A-1 cells [758]. β-Elemene can also inhibit cell migration and invasion in human cervical cancer SiHa, murine breast cancer 4T1 and melanoma B16F10 cells [749, 752, 759], whilst it inhibits cell growth and metastasis through angiogenesis suppression in murine melanoma B16F10 cells [752]. In addition, β-elemene can reverse drug resistance in human NSCLC erlotinib-resistant A549/ER cells by inhibiting P-gp expression and P-gp dependent drug efflux [760].

β-Elemene exerts anti-tumor effects through phosphatase and tensin homolog (PTEN) up-regulation and Akt suppression in human primary bladder cancer cells [746]. It also inhibits cell proliferation and invasion, and induces apoptosis via inhibition of Wnt/β‑catenin signaling pathway in human cervical cancer SiHa cells [749]. β-elemene-induced apoptosis is also shown to be through mitochondrial-related pathways, including p21 and Bax up-regulation, caspase-9 activation, Bcl-2 and survivin down-regulation [754]. On the other hand, it reverses drug resistance through mitochondrial-mediated apoptosis in human NSCLC cisplatin-resistant A549/DDP cells, via cytochrome c release, caspase-3 activation, Bcl-2 associated agonist of cell death (Bad) up-regulation and Bcl-2 down-regulation [761]. ER stress also plays a role in β-elemene-induced apoptosis. β-Elemene up-regulates ER stress markers to induce apoptosis in human NSCLC A549 cells, including PERK, IRE1α, ATF-6, ATF-4 and CHOP [747]. Moreover, it also enhances ROS production in human NSCLC A549 cells [747], and up-regulates HIF-1α expression via ROS to induce apoptosis in human osteosarcoma MG63 and Saos-2 cells [751].

β-Elemene has immunomodulatory effects in cancer and immune cells. It inhibits LPS-induced IL-6, TNF-α, IL-1β and IL-10 secretion, as well as inducible nitric oxide synthase in murine RAW264.7 marcophages [745]. M2 macrophages are regarded as tumor-associated macrophages, which can promote tumorigenesis [762]. β-Elemene can induce the polarization of M2 to M1 macrophages, and can also suppress M2 macrophage-treated conditioned medium-induced cell proliferation, migration and invasion in mouse lung cancer Lewis cells [762].

β-Elemene has poor water solubility, low oral bioavailability and severe phlebitis, so different delivery systems have been developed to solve these issues [763–765]. β-Elemene-loaded nanostructured lipid carriers are synthesized to enhance the intravenous delivery of β-elemene, and have higher bioavailiabity [763]. They inhibit tumor growth compared to β-elemene in hepatocellular carcinoma H22 xenograft mice. ETME, a novel β-elemene derivative, synergizes with arsenic trioxide to induce cell cycle arrest and apoptosis in human hepatocellular carcinoma SMMC-7721 cells, which is dependent on p53 [766]. Another β-elemene derivative, 13,14-bis(cis-3,5-dimethyl-1-piperazinyl)-β-elemene (IIi), is shown to inhibit cell proliferation in human gastric cancer SGC-7901 and cervical cancer HeLa cells, and inhibit tumor growth in sarcoma S-180 xenograft mice [767]. It also induces autophagy in human breast cancer MCF-7 cells, so it can be a potential anti-tumor agent.

The combination therapy is commonly used to enhance the efficacy of β-elemene for cancer treatment. β-Elemene when combined with cisplatin synergistically enhances apoptosis and inhibits cell proliferation in human gingival squamous cell carcinoma YD-38 cells and YD-38 xenograft mice [768]. β-Elemene potentiates the anti-proliferation effect of gefitinib as well as the induction of apoptosis and autophagy in human glioblastoma multiforme U251 and U87MG cells, through inhibiting EGFR signaling pathway [769]. It also reverses drug resistance in chemo-resistant breast cancer cells by reducing resistance transmission via exosomes [770], and enhances the sensitivity to TNF‐related apoptosis‐inducing ligand (TRAIL) partly through death-inducing signaling complex formation in human gastric cancer BGC-823 and SGC-7901 cells [771].

The Elemene Emulsion mainly containing β-elemene has been approved by China’s State Food and Drug Administration, and now it is prescribed as an oral or injected drug to improve anti-cancer efficacy and reduce the side effects as adjuvant therapy.

### Cepharanthine (CEP)

CEP (Fig. 2), a natural product derived from Chinese herbs such as *Stephania cepharantha* Hayata and *Stephania japonica*, is a cationic and amphipathic alkaloid that has been reported to decrease the fluidity of biological membranes [772]. With the presence of a 1-benzylisoquinoline moiety on alkyl chain, CEP belongs to a class of compounds called biscoclaurine alkaloids that have attracted significant attentions to pharmacologists and clinicians due to their resemblance to polypeptides [773]. CEP is widely used in Japan for the treatment of many acute and chronic diseases [773]. It exhibits anti-malarial, anti-viral, anti-inflammatory, anti-metastatic, and anti-cancer activities in various cell lines and animal models [772, 774–776]. Among its anti-cancer activities, CEP exhibits multiple pharmacological actions, including apoptosis and radiation sensitization, inhibition of angiogenesis and metastasis, and reversing MDR [776–789].

CEP induces apoptosis and cell cycle arrest in many types of cancer cells [783–786, 790]. It induces autophagy to mediate apoptosis through suppressing Akt/mTOR signaling pathway in human breast cancer MCF-7 and MDA-MB-231 cells [785], and stimulates AMPK-mTOR-dependent autophagy to induce cell death in apoptosis-resistant cells [791]. In contrast, the inhibition of autophagy is an effective treatment for NSCLC, and CEP is identified as a novel autophagic inhibitor in human NSCLC NCI-H1975 cells [782]. It inhibits autophagy by preventing autophagosome–lysosome fusion and inhibiting lysosomal cathepsin B and cathepsin D maturation. Therefore, this suggests that autophagy plays a dual role in cancer via different signaling routes. Moreover, CEP is suggested to be a potential anti-angiogenic agent, it blocks angiogenesis in endothelial cells, zebrafish and xenograft mice by inhibiting cholesterol trafficking [777]. It can also suppress metastasis in a highly metastatic tumor, cholangiocarcinoma, and markedly inhibit cell migration in human cholangiocarcinoma KKU-M213 and KKU-M214 cells [776].

CEP has anti-tumor action mainly by inducing apoptosis and ROS production [783, 784, 786]. ROS is shown to be an important factor to determine cell fate, and it can be regulated by p21 [792]. CEP efficiently inhibits the growth of p53-mutated colorectal cancer cells that are often resistant to commonly used chemotherapeutic agents [783]. It also effectively induces cell cycle arrest and apoptosis through ROS production, p21 up-regulation, cyclin A and Bcl‑2 down-regulation [783]. Similarly, CEP triggers apoptosis via ROS production and reducing mitochondrial membrane potential, thus inducing caspase-3 and PARP activation in human NSCLC H1299 and A549 cells [786]. It also exerts anti-tumor activity through ROS production and JNK activation in human choroidal melanoma MEL15-1 cells and xenograft mice [784]. In addition, CEP is also a potential anti-cancer drug for ovarian cancer by markedly increasing p21 expression and decreasing cyclins A and D levels in human ovarian cancer CaOV-3 and OVCAR3 cells [787].

CEP also plays an important role in immunity. It is shown to reduce IL-6 and TNF-α secretion in LPS-stimulated DCs, and inhibits LPS-stimulated DC maturation and antigen uptake by DCs [793]. CEP-treated DCs becomes a poor stimulator of allogeneic T cell activation and reduces IFN-γ production [793]. Therefore, it is suggested that CEP may have potential to be a cancer immunomodulatory agent.

Targeting P-gp using P-gp inhibitors is one of the main strategies to reverse MDR, and cepharanthine hydrochloride (CEH), a salt form of CEP, is suggested to be a potent P-gp inhibitor [779]. CEH exhibits MDR reversal potency in various cancer cells [779–781, 788]. CEH can reverse MDR-mediated cisplatin resistance in esophageal squamous cell carcinoma [780]. It increases the sensitivity of the cells and induces apoptosis via c-Jun activation, thus down-regulating P-gp and enhancing p21 levels. Similarly, CEH also reverses P-gp-mediated MDR through suppressing PI3K/Akt pathway in human ovarian cancer A2780/Taxol cells [788]. In addition, by reversing MDR, CEH induces cell cycle arrest and apoptosis in human nasopharyngeal carcinoma CNE-1 and CNE-2 cells [789].

In addition to chemotherapy, CEP may act as a radiosensitizer. Radiotherapy in the presence of CEP exhibits significant enhancement of tumor responses in human oral squamous cell carcinoma [778]. This pre-clinical data indicates that CEP has the potential to be used in clinical settings in combination with radiotherapy to treat oral squamous cell carcinoma. Moreover, paclitaxel and CEP co-loaded nano-particles also enhance the anti-cancer effects in human gastric cancer MKN45 cells and xenograft mice, suggesting that these nano-particles could be a potential formulation for gastric cancer [794]. In addition, CEP enhances the anti-cancer effects of dacomitinib in human NSCLC NCI-H1975 cells and NCI-H1975 xenograft mice [782], and cisplatin in lung and breast xenograft mice [777].

Although CEP has not yet been translated into clinical use for the treatment of cancer, the pharmacological activities and pre-clinical data support its significant clinical potential for anti-cancer therapy.

## Conclusions

Chinese herbal medicine has played, and still plays, an important role in human health care in China and other Asian countries. Natural products orignianted from Chinese herbal medicine has also become a “hot topic” in anti-cancer research. Chinese herbal medicine is also recognized worldwide as a rich source for the discovery of novel drugs in the past decades. Table 1 illustrates the experimental models and conditions, pharmacological effects, as well as mechanistic actions of the natural compounds derived from Chinese herbal medicine. Despite the unique anti-cancer beneficial features of many compounds derived from Chinese herbal medicine, their clinical applications are disproportionally limited. As of 2019, only preliminary clinical studies have been performed with artemisinins, emodin, cucurbitacins, tanshiones, shikonin, and CEP in various cancers, without any approved clinical applications. The phase I safety studies of UA-liposomes, oridonin derivative (HAO472), and wogonin were evaluated in patients with advanced solid tumors. Curcumin, pro-drug of triptolide (minnelide™), triptolide derivative (LLDT-8), and GA have been investigated on cancer therapy in phase II clinical trials. The phase II clinical trials of berberine hydrochloride, ginsenoside Rg3, and artesunate are being conducted in patients with cancer. EGCG was shown to have potential anti-cancer effects in a phase III clinical trial. Elemene Emulsion mainly containing β-elemene was approved by China’s State Food and Drug Administration as a Class 2 new drug in China. Based on our critical review of those clinical studies, we conclude that Chinese herbal medicine is a promising source and could be used as a complementary approach for cancer therapy.

**Table 1:** List of anti-cancer natural compounds from Chinese herbal medicines

Compounds	Origins	Cancer types	In vitro models	In vivo models	Anti-cancer effects	Underlying mechanisms	Dosage	Combinational agents	References
Curcumin	*Curcuma longa*, *Curcuma zedoaria*, *Acorus calamus* L.	Bladder cancer; breast cancer; cervical cancer; colorectal cancer; esophageal squamous cell carcinoma; gastrointestinal cancer; glioma; hepatocellular carcinoma; laryngeal cancer; lung cancer; leukemia; liver cancer; mesothelioma; neuroblastoma; oral squamous cell carcinoma; pancreatic cancer; prostate cancer; renal carcinoma; retinoblastoma	T24, RT4, MDA-MB-231, HeLa, SiHa, HCT-116, HT-29, RKO, HCT-15, DLD-1, EC1, EC9706, KYSE450, TE13, AGS, U87, T98G, HepG2, Tu212, A549, H1299, H460, H292, NCI-H520, NCI-H1373, NCI-H2170, K562, HL-60, PLC/PRF5, WRL68, Huh7, KMCH, RN5, N2a, SCC-25, Patu8988, Panc-1, C4-2, PC-3, LNCaP, VCaP, Caki, O-Rb50, Y79	BxPC-3-GemR xenograft mice; C4-2 xenograft mice; PC-3 xenograft mice; RN5 xenograft mice; U87 xenograft mice	Anti-angiogenesis; anti-metastasis; anti-proliferation; induces cell cycle arrest; inhibits cell viability; pro-apoptosis	Activates caspase-3, -9, PARP; Down-regulates Akt, Bcl-2, Bcl-xL, CTGF, cyclin D1, cyclin E1, ERK1/2, EZH2, FoxM1, GLI1, ITGA5, Jak1, JNK, MMP-2, Mcl-1, NF-κB, Notch1, p15, p16, p62, p70S6 K, ROCK1, RhoA, SHH, SSAT, STAT1, STAT3, Suz12, TROP2, vimentin, WT1, XIAP, YAP/TAZ; Enhances cytochrome c release, ROS accumulation; Inhibits CDK2 activity, PI3K/Akt/mTOR, SHH/GLI1, STAT3, TGF-β pathways; Up-regulates AIF, Bax, Bex-1, -2, -3, -4, -6, HIF-1α, microRNA-15a, microRNA-16-1, microRNA-99a, p21, p53, p73, PKD1, SMOX	0–5 μM; 0–15 μM; 0–16 μM; 0–20 μM; 0–25 μM; 0–40 μM; 0–50 μM; 0–125 μM; 10–40 μM; 15, 25 μM; 25 μM; 30 μM; 0–6 μg/ml; 5 mg/kg; 60 mg/kg; 200 mg/kg; 500 mg/kg; 25 μg/mouse	Gemcitabine; NVP-BEZ235; α-Tomatine	[12, 21, 795–814]
EGCG	*Camellia sinensis*	Biliary tract cancer; bladder cancer; breast cancer; cervical cancer; colorectal cancer; gallbladder cancer; gastric cancer; glioblastoma; head and neck cancer; lung cancer; nasopharyngeal carcinoma; NSCLC; oral cancer; pancreatic cancer; pheochromocytoma; prostate cancer; skin cancer	BDC, CCSW-1, EGI-1, SkChA-1, TFK-1, SW-780, MCF-7, 4T1, T47D, MDA-MB-231, MDA-MB-436, SUM-149, SUM-190, HeLa, DLD-1, HT-29, HCT-116, GBC, MzChA-1, MzChA-2, SGC-7901/FU, MGC-803/FU, AGS, C6, U251, SHG-44, U87, K3, K4, K5, CL1-5, CL1-0, TW01, TW06, NCI-H1299, A549, H460, SCC-9, MIA PaCa-2, Panc-1, PC-12, BCaPT1, BCaPT10, BCaPM-T10, LNCaP, A431, SCC13	4T1 xenograft mice; A549 xenograft mice; BCaPT10 xenograft mice; BCaPM-T10 xenograft mice; CL1-5 xenograft mice; Oral squamous cell carcinoma xenograft mice; PC-12 xenograft mice; SCG-7901/FU xenograft mice; SSC-9 xenograft mice; SUM-149 xenograft mice; SW-780 xenograft mice	Anti-angiogenesis; anti-metastasis; anti-proliferation; induces autophagy, cell cycle arrest; inhibits cell viability, epithelial–mesenchymal transition; pro-apoptosis	Activates caspase-3, -7, PARP; Down-regulates ABCG2, Akt, AXL, Bcl-2, Bcl-xL, E-cadherin, β-catenin, CDK2, CDK4, COX-2, CTTN, cyclin B1, cyclin D1, cyclin D2, cyclin D3, DNMT1, EGFR, ERα, ERK1/2, FAK, FN1, GSK3β, HDAC1, HER2, HSP90, IKKα, JNK, MDR-1, MGMT, MMP-2, MMP-9, NANOG, NF-κB, Notch, Oct-4, u-PA, paxillin, P-gp, PI3K, Raf-1, Snail, SOX2, Sp1, Src, STAT3, survivin, TFAP2A, Tyro3, VEGF, vimentin; Enhances cytochrome c release, ROS accumulation; Induces mitochondrial depolarization; Inhibits MAPK/ERK, PI3K/Akt pathways; Reduces ATP levels; Represses DNA replication; Up-regulates Bax, CK1α, endostatin, microRNA-16, p21, p53, TIMP-1, TIMP-2	0–20 µM; 0–40 µM; 0–50 µM; 0–100 µM; 0–200 µM; 0–400 μM; 2–100 μM; 10 μM; 20 μM; 25, 50, 100 μM; 40 μM; 50, 100 μM; 80 µM; 0–60 μg/ml; 10 mg/kg; 10–20 mg/kg; 15 mg/kg; 16.5 mg/kg; 20 mg/kg; 25 mg/kg; 25–100 mg/kg; 50 mg/kg; 0.025%, 0.05%; 0.06%	Bleomycin; Cisplatin; Curcumin; Docetaxel; 5-Fluorouracil; Oxaliplatin; Pterostilbene; Temozolomide	[93–95, 100, 101, 103, 123–125, 815–834]
Berberine	*Coptidis cgubebsus* Franch., *Mahonia bealei* (Fort.) Carr., *Phellodendron chinense* Schneid	Breast cancer; cervical cancer; cholangiocarcinoma; colorectal cancer; endometrial carcinoma; esophageal squamous cancer; gastric cancer; glioblastoma; head and neck cancer; hepatocellular carcinoma; leukemia; lung cancer; medulloblastoma; melanoma; nasopharyngeal carcinoma; oral squamous cell carcinoma; osteosarcoma; ovarian cancer; pancreatic cancer; prostate cancer; skin cancer; uterine leiomyoma	MCF-7, MCF-7/HER2, MCF-7/TAM, MDA-MB157, MDA-MB231, MDA-MB453, BT20, BT549, Hs578T, T47D, SKBR3, BT474, HeLa, SiHa, QBC939, KKU-213, KKU-214, SW-480, SW-620, HT-29, DLD-1; HCT-116, LS174T, LoVo, Eca109, TE13, KYSE-70, EAC, SKGT4, AN3 CA, HEC-1-A, KLE, MGC-803, SGC-7901, AGS, BGC-823, MKN45, U87, U251, U118, SHG-44, FaDu, H22, Hepa1-6, HepG2, Bel-7404, Huh7, WRL68, MHCC97L, K562, A549, B16F10, HONE1, HK1-EBV, CNE-2, KB, U2OS, Panc-1, MIA PaCa-2, LNCaP, DU-145, LAPC-4, PC-3, 22RV1, C4-2B, C42, RM-1, A-431	22RV1 xenograft mice; A2780 xenograft mice; A549 xenograft mice; BGC-823 xenograft mice; Eca109 xenograft mice; H22 xenograft mice; HONE1 xenograft mice; LoVo xenograft mice; LNCaP xenograft mice; MDA-MB-231 xenograft mice; Medulloblastoma xenograft mice; MHCC97L xenograft mice; SGC-7901 xenograft mice; SW-620 xenograft mice; SiHa xenograft mice; U87 xenograft mice	Anti-angiogenesis; anti-proliferation; anti-metastasis; enhances radiosensitivity; induces autophagy, cell cycle arrest; inhibits cell viability, epithelial–mesenchymal transition; pro-apoptosis	Activates caspase-3, -7, -8, -9, PARP; Decreases mitochondrial membrane potential, catalase and superoxide dismutase activities; Down-regulates Akt, AR, Bcl-2, Bcl-xL, Bid, β-catenin, N-cadherin, CDK1, CDK2, CDK4, COX-2, PLA2, cyclin A1, cyclin B1, cyclin D1, cyclin E, DHCR24, DHFR, E2F1, EBNA1, EGFR, EF-Tu, ERK, Ezrin, FAK, FN, HER2, HIF-1α, HMGB1, HNF4α, ITGβ1, Jak2, JNK, Mcl-1, MEK, MMP-1, MMP-2, MMP-9, mTOR, c-Myc, NANOG, NF-κB, iNOS, occludin, Oct-4, p38, p50, p62, p100, p105, p70S6 K, paxillin, u-PA, PCNA, PDK1, PGE_2_, PKC-α, PSA, PTEN, PTTG-1, RAD51, b-Raf, c-Raf, Septin-8, Slug, Snail, SOX2, Sp1, Src, STAT3, survivin, UQCRC1, VEGF, vimentin, Wnt5α, ZEBRA; Enhances cytochrome c release, ROS accumulation, SSAT activity; Induces DNA damage; Inhibits Akt/mTOR/p70S6 K/S6, arachidonic acid metabolic, androgen receptor pathways; Reduces NO production; Suppresses Hedgehog signaling pathway; Up-regulates ACC, AIF, AMPKα, Apaf-1, ATF-6, Bad, Bak, Bax, Beclin-1, Bim, E-cadherin, DR5, FasL, FoxO1, FoxO3a, GRP78, HRK, Lig4, MST1, p21, p27, p53, PHLPP2, SSAT, TIMP-2, TRAIL, ULK1	0–10 µM; 0–20 μM; 0–25 μM; 0–40 μM; 0–50 µM; 0–80 μM; 0–90 µM; 0–100 µM; 0–120 µM; 0–150 μM; 0–160 µM; 0–200 µM; 0–250 μM; 0–350 μM; 0–1000 μM; 10–80 μM; 15 µM; 20 μM; 50 μM; 0–1 µg/ml; 0–80 μg/ml; 5 mg/kg; 10 mg/kg; 12.5–50 mg/kg; 20 mg/kg; 50, 100 mg/kg; 50–200 mg/kg; 200 mg/kg; 0.01136 g/kg	Caffeine; Cetuximab; Doxorubicin; Erlotinib; *d*-limonene; Niraparib; Tamoxifen; Taxol; TRAIL	[139, 140, 144, 145, 147–149, 153, 171, 174, 835–870]
Artemisinins	*Artemisia annua* L.	Breast cancer; cervical cancer; colorectal cancer; gallbladder cancer; gastric cancer; glioma; hepatocellular carcinoma; Ishikawa endometrial cancer; lung cancer; neuroblastoma; oral carcinoma; pancreatic cancer	MCF-7, MDA-MB-231, HeLa, HCT-116, SW-480, SW-620, GBC-SD, NOZ, MGC-803, C6, HepG2, Hep3B, SMMC-7721, Ishikawa, A375, A549, ASTC-a-1, H1299, BE(2) -C, SHEP1, SK-N-AS, SK-N-DZ, SCC25, RIN	A549 xenograft mice; BE(2)-C xenograft mice; C6 xenograft mice; GBC-SD xenograft mice; HCT-116 xenograft mice; HepG2 xenograft mice; NOZ xenograft mice	Anti-metastasis; anti-proliferation; induces apoptosis, autophagy, cell cycle arrest; inhibits cell viability	Activates caspase-3, -8, -9, PARP; Decreases mitochondrial membrane potential, MMP activity; Down-regulates Bcl-2, CDK2, CDK4, cyclin D1, cyclin E2, Dvl2, ERK1/2, LRP6, MMP-2, NANOG, Oct-4, p38, p62, SOX2, vimentin, Wnt5α/β; Enhances cytochrome c release, ROS accumulation; Induces DNA damage; Inhibits Wnt/β-catenin signaling pathway; Up-regulates Axin2, Bax, E-cadherin, β-catenin, NKD2, p16, TIMP-2	0–75 μM; 0–160 μM; 0–200 μM; 0–250 μM; 0–400 μM; 0–500 μM; 0–1000 μM; 0–1200 μM; 10–320 μM; 40–160 μM; 0–40 μg/ml; 10 mg/kg; 50 mg/kg; 60 mg/kg; 100 mg/kg	3CA; Halofuginone; Holotransferrin; Resveratrol	[184, 186, 213, 871–883]
Ginsenoside Rg3	*Panax notoginseng* (Burk.) F. H. Chen, *Panax ginseng*, *Cinnamomum cassia* Presl.	Breast cancer; colorectal cancer; esophageal carcinoma; gallbladder cancer; gastric cancer; glioblastoma; glioma; hepatocellular carcinoma; leukemia; lung cancer; melanoma; multiple myeloma; ovarian cancer; pancreatic cancer; prostate cancer	BT549, MDA-MB-231, MDA-MB-453, CT-26, HCT-116, LoVo, SW-480, SW-620, EC109, KYSE170, TE1, GBC-SD, Mz-ChA-1, QBC939, SGC-7901, U87MG, U87, Hep1-6, HepG2, Lewis, Jurkat, A549, A549/DDP, H23, H1299, A375, C8161, SK-MEL-28, RPMI 8226, SKO-007, U266, A2780, 3AO, SKOV3, AcPC-1, BxPC-3, Panc-1, SW1990, PC-3	A375 xenograft mice; A549 xenograft mice; BxPC-3 xenograft mice; CT-26 xenograft mice; GBC-SD xenograft mice; HCT-116 xenograft mice; Hep1-6 xenograft mice; H23 xenograft mice; Lewis tumor-bearing mice; LoVo xenograft mice; MDA-MB-231 xenograft mice; MCF- 7 xenograft mice; SKOV3 xenograft mice; SW1990 xenograft mice; SW-620 xenograft mice	Anti-angiogenesis; anti-proliferation; anti-metastasis; enhances radiosensitivity; increases cell survival; induces autophagy, cell cycle arrest; inhibits chemotaxis, epithelial–mesenchymal transition; pro-apoptosis	Activates caspase-3, -8, -9, 12, PARP; Decreases mitochondrial membrane potential; Down-regulates Akt, AQP1, B7-H1, B7-H3, Bcl-2, Bcl-xL, VE-cadherin, CDK2, COX-2, CXCR4, cyclin D1, cyclin E, DNMT3A, EGFR, EPHA2, ERK, FUT4, HDAC3, HIF-1α, HK2, IAP, JNK, LeY, MMP-2, MMP-9, mTOR, c-Myc, NF-κB, p38, p53, PCNA, PD-L1, PI3K, PKM2, Rb, STAT3, surviving, VEGF; Enhances cytochrome c release, ROS production; Inhibits the Warburg effect, Wnt/β-catenin pathway; Up-regulates Atg-5, Atg-7, Bax, CHOP, IRE1, microRNA-532-3p, p16, p21, p27, p53, PERK	0–10 μM; 0–30 μM; 0–35 μM; 0–60 μM; 0–80 μM; 0–100 μM; 0–150 μM; 0–160 μM; 0–200 μM; 0–400 μM; 0–600 μM; 25 μM; 0–600 ng/ml; 0–80 μg/ml; 0–100 μg/ml; 0–160 μg/ml; 0–200 μg/ml; 40, 80 μg/ml; 50 μg/ml; 80, 160 μg/ml; 80, 160 mg/ml; 3 mg/kg; 5 mg/kg; 5, 10, 20 mg/kg; 6 mg/kg; 7.5–30 mg/kg; 10 mg/kg; 20 mg/kg	Cisplatin; Cyclophosphamide; Erlotinib; 5-Fluorouracil; Oxaliplatin; Paclitaxel	[227, 232–234, 236–241, 246, 252–255, 260, 884–900]
Ursolic acid	*Vaccinium macrocarpon* Ait., *Arctostaphylos uva*-*ursi* (L.) *Spreng*, *Rhododendron hymenanthes* Makino, *Eriobotrya japonica*, *Rosemarinus officinalis*, *Calluna vulgaris*, *Eugenia jambolana*, *Ocimum sanctum*	Bladder cancer; breast cancer; cervical cancer; colorectal cancer; Ehrlick ascites carcinoma; leukemia; liver cancer; lung cancer; melanoma; ovarian cancer; prostate cancer; skin cancer	BIU-87, T24, MDA-MB-231, MCF-7, MCF-7/ADR, HeLa, HCT-8, HCT-116, HT-29, Caco-2, SW-480, SW-620, HCT-15, CO115, HL-60, HL-60/ADR, Jurkat, K562, K562/ADR, U937, HL-60/ADR, Hep3B, Huh7, HA22T, A549, H3255, Calu-6, M4Beu, SKOV3, DU-145, LNCaP, PC-3	12-dimethylbenz[a]anthracene-induced mice; DU-145 xenograft mice; Ehrlich ascites carcinoma xenograft mice; HCT-116 xenograft mice; HCT-15 xenograft mice; U937 xenograft mice	Anti-angiogenesis; anti-metastasis; anti-proliferation; enhances chemosensitivity; induces apoptosis, autophagy, cell cycle arrest; inhibits MDR	Activates caspase-3, -7, -8, -9, Fas receptor, PARP; Decreases mitochrondrial membrane potential; Down-regulates AEG-1, Akt, Bcl-2, Bcl-xL, Bid, β-catenin, CD31, cyclin D1, EGFR, ERK, cFLIP, FN, HIF-1α, cIAP-1, ICAM-1, IκBα, IKKα/β, IL-8, Jak2, Ki-67, Mcl-1, MMP-2, MMP-9, NF-κB, iNOS, p65, u-PA, P-gp, S6 K, Src, STAT3, survivin, mTOR, TNF-α, VEGF, Wnt5α/β, XIAP; Enhances cytochrome c release, PGE_2_ levels, ROS production; Inhibits NO production; Up-regulates ACC, AMPK, ASK1, Bax, CHOP, DR4, DR5, eIF2α, GRP78, GSK3β, IL-12, JNK, c-Jun, NADPH, p21, p52, p53, PERK	0–4 μM; 0–16 μM; 0–17.5 μM; 0–20 μM; 0–40 μM; 0–50 μM; 0–80 μM; 0–100 μM; 4 μM; 20 μM; 0–400 μg/ml; 10 mg/kg; 25–100 mg/kg; 50 mg/kg; 75 mg/kg; 250 mg/kg; 2 μmol/mouse	Capecitabine; 5-Fluorouracil; Oxaliplatin; Resveratrol; TRAIL	[274, 276, 281, 283–289, 293, 901–907]
Silibinin	*Silybum marianum* L. Gaertn	Breast cancer; colorectal cancer; epidermoid carcinoma; glioblastoma; hepatocellular carcinoma; osteosarcoma; pancreatic cancer; prostate cancer; renal carcinoma; thyroid cancer	BT-20, MCF-7, MDA-MB-231, MDA-MB-468, SKBR3, T47D, AsPC-1, BxPC-3, Panc-1, HT-29, HCT-116, LoVo, SW-480, Caco-2, A-431, LN18, SNB19, U87MG, Hep3B, HepG2, SK-Hep-1, SaOS2, PC-3, 769-P, 786-O, ACHN, OS-RC-2, SW839, Caki, TPC-1	786-O xenograft mice; Azoxymethane-induced rats; Diethylnitrosamine-induced mice	Anti-metastasis; anti-proliferation; induces apoptosis, autophagy, cell cycle arrest; inhibits cell viability	Activates caspase-3, -8, -9, PARP; Down-regulates Akt, Bcl-2, EGFR, ERK, GLI1, IL-1β, FN, MMP-2, MMP-7, MMP-9, NF-κB, iNOS, PLA2, TNF-α, mTOR; Enhances CYP2E1 activity, cytochrome c release, ROS production; Up-regulates AIF, Bax, Bid, calpain, EGR1, ICAD, NAG-1, PTEN	0–75 μM; 0–100 μM; 0–200 μM; 0–300 μM; 0–800 μM; 25, 50 μM; 120 μM; 125 μM; 200 mg/kg; 300 mg/kg; 0.5%	Curcumin; luteolin	[318, 319, 329–331, 334, 342–345, 357, 358, 908–910]
Emodin	*Rheum palmatum*, *Polygonum cuspidatum*, *Polygonum multiflorum*, C*assia obtusifolia*	Bladder cancer; breast cancer; colorectal cancer; gallbladder cancer; gastric cancer; hepatocellular carcinoma; lung cancer; nasopharyngeal carcinoma; oral carcinogenesis; ovarian cancer; pancreatic cancer; prostate cancer	MBT2, T24, TSGH8301, 4T1, EO771, MCF-7, MDA-MB-231, MDA-MB-435, MDA-MB-453, HCT-116, LoVo, LS1034, SGC-996, MKN45, C3A, Hep3B, HepG2, PLC/PRF/5, SMMC-7721, A549, CNE-2Z, A2780, SKOV3, AsPC-1, BxPC-3, Panc-1, SW1990, SW1990/GZ, PC-3	4T1 xenograft mice; 7,12-dimethyl benz(a)anthracene-induced golden Syrian hamsters; EO771 xenograft mice; HCCLM3 tumor-bearing mice; LS1034 xenograft mice; MDA-MB-231 xenograft mice; SGC-996 xenograft mice; SKOV3 xenograft mice; SW1990 xenograft mice; T24 xenograft mice	Anti-metastasis; anti-proliferation; induces apoptosis, autophagy, cell cycle arrest; inhibits cell viability, epithelial-mesenchymal transition	Activates caspase-3, -9, PARP, chloride currents; Decreases mitochondrial membrane potential; Down-regulates Akt, Bcl-2, Bcl-xL, Bim-1, β-catenin, CDK1, CSF1, CSF2, CXCL12, CXCR4, cyclin D1, ERα, ERK, FABP4, bFGF, HBP17, HER2, ILK, Jagged1, Jak1, Jak2, Ki-67, Mcl-1, MCP-1, MMP-2, MMP-9, MRP1, NF-κB, p38, p62, u-PA, u-PAR, Slug, Snail, Src, STAT3, survivin, Thy-1, VEGF, vimentin, XIAP, ZEB1; Enhances Ca^2+^ levels, cytochrome c release, ROS production; Up-regulates AIF, Bax, Beclin-1, E-cadherin, GSK3β, microRNA-34, Notch1, SHP-1	0–10 μM; 0–40 μM; 0–50 μM; 0–60 μM; 0–80 μM; 0–100 μM; 0–250 μM; 0–320 μM; 0–1000 μM; 20 μM; 20–80 μM; 40 μM; 0.05 mM; 40 mg/ml; 20, 40 mg/kg; 20, 50 mg/kg; 25, 50 mg/kg; 40 mg/kg; 50 mg/kg	Cisplatin; curcumin; 5-fluorouracil; gemcitabine	[64, 367–378, 380, 382, 383, 385, 389, 394, 395, 402, 403, 405, 911]
Triptolide	*Tripterygium wilfordii* Hook. F.	Bladder cancer; breast cancer; colorectal cancer; endometrial carcinoma; liver cancer; lung cancer; lymphoma; melanoma; myeloma; nasopharyngeal carcinoma; neuroblastoma; osteosarcoma; ovarian cancer; oral cancer; pancreatic cancer; prostate cancer	UMUC3, MDA-MB-231, MCF-7, DLD-1, HCT-116, HEC-1B, MHCC-97H, HepaRG, HepG2, H460, H358, A549, A549/Taxol, HTB182, BEAS-2B, H1299, NCI-H2009, NCI-H460, Jurkat, Molt-3, Raji, NAMALWA, Daudi, B16F10, HS-sultan, IM9, RPMI 8226, U266, CNE, MG63, BE(2)-C, SH-SY5Y, SAOS2, U2OS, SKOV3, SKOV3/DDP, A2780, SAS, Panc-1, AsPC-1, SW1990, BxPC-3, LNCaP, PC-3, DU-145	3LL xenograft mice; A549 xenograft mice; AsPC-1 xenograft mice; BE(2)-C xenograft mice; CNE xenograft mice; Daudi xenograft mice; H358 xenograft mice; H460 xenograft mice; HEC-1B xenograft mice; Jurkat xenograft mice; MHCC-97H xenograft mice; SAS + U937 xenograft mice; SKOV3/DDP xenograft mice; SW1990 xenograft mice	Anti-angiogenesis; anti-metastasis; anti-proliferation; enhances radiosensitivity; induces autophagy, cell cycle arrest; inhibits cell viability; pro-apoptosis	Activates caspase-3, -7, -8, -9, GSK3β, PARP; Decreases mitochondrial membrane potential; Down-regulates Akt, AR, BCAR1, Bcl-2, β-catenin, Cav-1, CD147, CDK2, CHK1, COX IV, CXCR4, cyclin A1, ERK, ETS2, FAK, c-FLIP, GRB2, HIF-1α, HSF1, HSP70, IκBα, ITGβ1, ITGαVβ6, JMJD3, JMJD2B, NK, p38 MAPK, Mcl-1, MKP-1, MMP-2, MMP-3, MMP-7, MMP-9, MMP-14, MMP-19, c–Myc, NF-κB, iNOS, Nrf2, p65, PCNA, PI3K, PYK2, ROCK1, RhoA, Slug, Snail, SOS1, Src, survivin, mTOR, Twist, UTX, VEGF, vimentin, ZEB1; Enhances Ca^2+^ levels, cytochrome c release, ROS production; Inhibits Wnt/β-Catenin pathway; Up-regulates ATM, Bax, Beclin-1, E-cadherin, cathepsin B, Fas, DKK1, DR5, ENY2, FADD, FRZB, GSK3β, IL-2, γ-H2AX, LMP, LSD1, p53, PPARγ, PTEN, SFRP1, SIRT3, Smac, SUV39H1, TNF-α, Wnt3α	0–10 nM; 0–40 nM; 0–50 nM; 0–80 nM; 0–100 nM; 0–160 nM; 0–200 nM; 0–300 nM; 0–320 nM; 0–400 nM; 0–500 nM; 0–0.1 μM; 0–25 μM; 0–150 μM; 0–200 μM; 10 nM; 50, 72 nM; 100 nM; 0–8 ng/ml; 0–36 ng/ml; 0–50 ng/ml; 0–400 ng/ml; 5, 10 ng/ml; 5–160 ng/ml; 8 ng/ml; 250 μg/kg; 0–0.8 mg/kg; 0.04–0.36 mg/kg; 0.075 mg/kg; 0.15 mg/kg; 0.25 mg/kg; 0.4 mg/kg; 1 mg/kg; 1.5 mg/kg; 2–4 μg/mouse	Cisplatin; epirubicin; 5-fluorouracil; gemcitabine; hydroxycamptothecin	[408, 410, 411, 414, 415, 417, 419, 422, 423, 425–427, 429, 431–434, 438, 444, 446, 453, 454, 912–925]
Cucurbitacin B	*Bryonia, Cucumis, Cucurbita and Lepidium sativum*	Breast cancer; cervical cancer; hepatocellular carcinoma; lung cancer; neuroblastoma; prostate cancer	4T1, HCC1937, MCF-7, MCF-7/ADR, MDA-MB-231, MDA-MB-436, SKBR-3, HeLa, T47D, SK-Hep1, Hep3B, HepG2, Bel-7402, Bel-7402/5-Fu, A549, H1299, H23; SH-SY5Y; LNCaP, PC-3	4-(methylnitrosamino)-1-(3-pyridyl)-1-butanone-induced mice; 4T-1 xenograft mice; Bel-7402 xenograft mice; MDA-MB-231 xenograft mice; NNK-induced mice; PC-3 xenograft mice	Anti-angiogenesis; Anti-metastasis; Anti-proliferation; Inducing apoptosis, cell cycle arrest; Inhibits epithelial-mesenchymal transition	Activates caspase-3, -8, -9, PARP; Decreases mitochondrial membrane potential; Down-regulates Akt, ACLY, BCAR1, Bcl-2, β-catenin, CD31, CDK1, CIP2A, cyclin B1, cyclin D1, EGFR, ERK, FAK, galectin-3, GSK3β, HER2, HIF-1α, ILK1, ITGA6, ITGB4, Jak2, MMP-2, MMP-9, MRP1, c-Myc, nucleophosmin, P-gp, paxillin, RhoA, ROCK1, STAT3, Src, survivin, TACE, TCF1, mTOR, Twist, VEGF, VEGFR2, Wnt3; Enhances cytochrome c release, PP2A activity, ROS production; Inhibits Wnt/β-catenin pathway; Up-regulates ATM, Bax, Bim, E-cadherin, CDC25C, CHK1, γ-H2AX, JNK, p21, p53	0–100 nM; 0–200 nM; 0–1000 nM; 0.1–1000 nM; 0–0.1 μM; 0–1 μM; 0–1.6 μM; 0–30 μM; 0–100 μM; 0–128 μM; 0.02– 62.5 μM; 0–100 μg/ml; 0.1–100 μg/ml; 0.1, 0.2 mg/kg; 0.1, 0.25 mg/kg; 0.5, 1 mg/kg; 1, 5 mg/kg; 2 mg/kg; 10 mg/kg; 0.1 μmol/mouse	Curcumin; docetaxel; gefitinib; gemcitabine	[452, 460–462, 472–475, 485, 499, 926–931]
Tanshinone IIA	*Salvia miltiorrhiza* Bunge	Breast cancer; bladder cancer; cervical cancer; colorectal cancer; esophageal carcinoma; gastric cancer; NSCLC; osteosarcoma; oral squamous carcinoma	BT-20, 5637, BFTC 905, T24, HeLa, C33 A, HCT-116, COLO-205, LoVo, HT-29, SW-620, Eca109, SGC-7901, MKN45, A549, H596, H1299, Calu-1, H460, 143B, SCC090	HT-29 xenograft mice; MKN45 xenograft mice; SGC-7901 xenograft mice; 143B xenograft mice	Anti-angiogenesis; anti-metastasis; anti-proliferation; enhances chemosensitivity, radiosensitivity; induces autophagy, cell cycle arrest; inhibits cell viability, epithelial–mesenchymal transition; pro-apoptosis	Activates caspase-3, -8, -9, -12, PARP; Down-regulates ALDH1, Bcl-2, BIP, N-cadherin, β-catenin, CD31, COX-2, CTGF, FoxM1, HIF-1α, Ki-67, LEF1, MCP-1, Mfn-1, Mfn-2, MMP-2, MMP-9, c-Myc, NANOG, Opa-1, p65, PCNA, Slug, Snail, STAT3, survivin, TCF3, VEGF, vimentin, YAP; Enhances cytochrome c release, ROS accumulation; Reduces mitochondrial membrane potential; Up-regulates ATF-4, Bax, Bak, Bad, E-cadherin, CHOP, Drp-1, DR5, GRP78, p21	0–8 μM; 0–20 µM; 0–40 µM; 0–60 µM; 0–80 μM; 0–100 µM; 0–54.4 μM; 0–20 ng/ml; 0–4 µg/ml; 0–8 μg/ml; 0–18 µg/ml; 0–60 µg/ml; 1 mg/kg; 10, 30 mg/kg; 20 mg/kg	Adriamycin 5-fluorouracil; TRAIL	[514, 515, 517, 519, 523, 531, 539, 932–935]
Oridonin	*Rabdosia rubescens* (Hemsl.) Hara	Breast cancer; cervical cancer; colorectal cancer; esophageal cancer; gastric cancer; hepatocellular carcinoma; laryngeal; leukemia; liver cancer; lung cancer; melanoma; multiple myeloma; neuroblastoma; oral squamous carcinoma; osteosarcoma; ovarian cancer; pancreatic cancer; prostate cancer; uveal melanoma	4T1, MCF-7, MDA‑MB‑231, SW-48, SW-480, SW-620, SW-1116, HeLa, LoVo, HCT-116, HCT-15, COLO-205, RKO, EC9706, KYSE-30, KYSE-150, SGC-7901, AGS, HepG2, Huh6, MHCC97-H HCC, Hep-2, K562, K562/ADR, HL-60, HL-60/ADR, MV4-11/DDP, MOLM-13/DDP, A549, SHSY-5Y, SK-N-MC, LP-1, SCC-25, HSC-3, HSC-4, MG63, U2OS, HOS, Saos-2, 143B, WSU-HN4, WSU-HN6, CAL27, SKOV3, BxPC-3, PC-3, LNCaP, DU-145, RM-1, MUM2B, OCM-1	143B xenograft mice; 4T1 xenograft mice; HCT-116 xenograft mice; HepG2 xenograft mice and zebrafish; HL-60 xenograft mice; HOS xenograft mice; K562 xenograft mice; KYSE-150 xenograft mice; LoVo xenograft mice; MV4-11/DDP xenograft mice; RM-1 xenograft mice; SCC-25 xenograft mice; SHSY-5Y xenograft mice; SW-480 xenograft mice; WSU-HN6 xenograft mice	Anti-angiogenesis; anti-metastasis; anti-proliferation; induces apoptosis, autophagy, cell cycle arrest, epithelial–mesenchymal transition	Activates caspase-3, -8, -9, PARP; Decreases mitochondrial membrane potential; Down-regulates Akt, AMPK, AP-1, Bcl-2, Bcl-xL, N-cadherin, CD31, CD44, CDC25C, CDK1, CDK2, Claudin 1, Claudin 4, Claudin 7, α-CPI, cyclin B1, cyclin D1, cyclin E, DHFR, EGFR, ERK, GLUT-1, GSK3β, HO-1, ICAD, Mcl-1, MCT1, MDM2, MMP-2, MMP-9, c-Myc, NF-κB, Notch, Nrf2, NQO1, p38, p62, PCNA, PI3K, Rac2, Raf, Ras, SERTAD1, Slug, Smad, Snail, Stathmin, SREBP1, mTOR, vimentin; Enhances cytochrome c release, intracellular Ca^2+^ levels, ROS production; Inhibits TrxR activity; Up-regulates AIF, ASK1, ATM, Bad, Bax, Beclin-1, Bim, BMP7, E-cadherin, CHK2, CHOP, CKS2, eIF2α, FADD, GADD45AQ, GRP78, γ-H2AX, HERC5, HSP90, IRE1, JNK, p21, p53, PERK, PPARγ, RECQL4, SFN, PTEN	0–1000 nM; 0–1.5 μM; 0–4 μM; 0–9 μM; 0–12 μM; 0–15 μM; 0–20 μM; 0–25 μM; 0–30 μM; 0–32 μM; 0–40 μM; 0–50 μM; 0–60 μM; 0–64 μM; 0–80 μM; 0–100 μM; 0–160 μM; 36 μM; 0–10 mM; 0–64 μg/ml; 5–30 μg/ml; 1.875, 7.5 mg/ml; 1 mg/kg; 2–8 mg/kg; 2.5–10 mg/kg; 5, 10 mg/kg; 5–10 mg/kg; 5–15 mg/kg; 7.5–30 mg/kg; 10 mg/kg; 10, 20 mg/kg; 15 mg/kg; 30 mg/kg; 50, 100 mg/kg	Cisplatin; NVP-BEZ235; valproic acid	[544–556, 558–567, 573–576, 578, 579, 936–946]
Shikonin	*Lithospermum erythrorhizon*, *Arnebia euchroma*, *Arnebia guttata*	Breast cancer; cervical cancer; colorectal cancer; gallbladder cancer; gastric cancer; glioblastoma multiforme; glioma; hepatocellular carcinoma; leukemia; lung cancer; NSCLC; renal carcinoma; pancreatic cancer; thyroid cancer	MCF-7, MDA-MB-231, SKBR3, HeLa, HCT-116, HT-29, SNU-407, SW-1116, SW-680, SW-620, NOZ, BGC-823, SGC-7901, Primary glioblastoma stem cells, C6, SHG-44, U87, U251, SMMC-7721, NB4, Calu-6, H358, HCC-2279, NCI-H15, NCI-H460, NCI-H1229, NCI-H1437, NCI-H1703, A549, 789-O, Capan-1, Suit-2, 8305C, 8505C, BCPAP, C643, FTC133, IHH4, K1, TPC1	A549 xenograft mice; Glioblastoma stem cell xenograft mice; HCT-116 xenograft mice; NOZ xenograft mice; SGC-7901 xenograft mice	Anti-metastasis; anti-proliferation; enhances chemosensitivity; induces apoptosis, cell cycle arrest, necroptosis	Activates caspases-3, -8, -9, -12, PARP, JNK/c-Jun, p38 MAPK, PERK/elF2α/CHOP, pathways; Decreases mitochondrial membrane potential; Down-regulates Akt, Bcl-2, CDK4, cyclin D1, FoxO3a, ICBP90, ITGβ1, MDM2, MMP-9, c-Myc, RIPK1; Elevates intracellular Ca^2+^ and ROS levels; Enhances Ca^2+^ and K^+^ efflux; Inhibits ERK pathway, PKM2 activity; Promotes RIP1/RIP3 necrosome formation; Up-regulates Bax, Bim, Cbl-b, CHOP, cytochrome c, EGR1, eIF2α, GRP78, IRE1α, p16, p21, p53, p73, PERK, RIP1, RIP3	0–2 μM; 0–4 μM; 0–5 μM; 0–6 μM; 0–10 μM; 0–20 μM; 0–50 μM; 0.1–0.4 μM; 1 μM; 2 μM; 20 mg/kg; 2 mg/kg	Cisplatin; 5-fluorouracil; oxaliplatin	[593, 595, 600, 603, 607, 611, 614, 615, 617, 620, 636, 947–952]
Gambogic acid	*G. hanburyi*, *G. Morella*	Breast cancer; colorectal cancer; glioma; hepatocellular carcinoma; NSCLC; osteosarcoma; ovarian cancer; pancreatic cancer; prostate cancer; renal carcinoma	4T1, MCF-7, MDA-MB-231, HCT-15, HCT-15R, HCT-116, HT-29, SW-480, SW-620, LoVo/L-OHP, LoVo/L-OHP/GA, T98G, Hep3B, Huh7, A549, A549/DDP, SPC-A-1, MG63, SKOV3, BxPC-3, Capan-1, Capan-2, Colo-357, MIA PaCa-2, Panc-1, Suit-007, Suit-2, SW1990, B6WT, DU-145, LAPC-4, LNCaP, PC-3, PCAP-1, PTEN^−/−^/p53^−/−^, Caki	4T1 xenograft mice; A549 xenograft mice; B16F10 and MC38 xenograft mice; BxPC-3 xenograft mice; C26 xenograft mice; SKOV3 xenograft mice	Anti-angiogenesis; anti-metastasis; anti-proliferation; anti-tumor growth; enhances chemosensitivity; induces apoptosis, autophagy, cell cycle arrest; inhibits cell viability, survival	Activates caspase-3, -7, -8, -9, PARP, JNK pathway; Decreases mitochondrial membrane potential; Down-regulates Akt, ALDOA, ATG4B, Bcl-2, Bcl-xL, β-catenin, cFLIP_L_, cyclin D1, DLL1, DLL3, DLL4, ERK, Jagged1, Jagged2, LRP, p-53, P-gp, Mcl-1, MMP-2, MMP-9, MRP2, PI3K, RRM2, SIRT1, survivin, TOPIIα, VEGF, XIAP; Enhances ROS accumulation, cytochrome c release; Inhibits ERK/E2F1/RRM2, MAPK, PI3K/Akt pathways, NF-κB p65 binding activity, Trx activity; Up-regulates AIF, Atg-5, Bax, CHOP, DUSP1, DUSP5, FoxO3a, c-Jun, p27, p53	200–400 nM; 0–1 μM; 0–2 μM; 0–3 μM; 0–5 μM; 0–8 μM; 0–10 μM; 0–40 μM; 0–50 μM; 0–51.8 μM; 0.5 µM; 0–3 μg/ml; 2 mg/kg; 8 mg/kg	Chlorochalcone; Cisplatin; Doxorubicin; 5–Fluorouracil; Gemcitabine; Nal^131^; Oxaliplatin; Retinoic acid; TRAIL	[639, 644, 646, 647, 650, 656, 657, 663–665, 953–966]
Artesunate	*Artemisia annua* L.	B-cell lymphoma; bladder cancer; breast cancer; colorectal cancer; gastric cancer; head and neck cancer; hepatocellular carcinoma; myelodysplastic syndrome; ovarian cancer; pancreatic cancer; prostate cancer; rhabdomyosarcoma	BL-41, Raji, Ramos, Rec-1, RT4, T24, ACHN, BT-474, MCF-7, MDA-MB-231, BGC-823, HGC-27, MGC-803, SGC-7901, HN3, HN4, HN9, SKM-1, HO8910, SKOV3, AsPC-1, BxPC-3, Colo-357, Panc-1, DU-145, LNCaP, RD18, TE671	BL-41 xenograft mice; A2780 xenograft mice; HO8910 xenograft mice; TE671 xenograft mice; MCF-7 xenograft mice	Anti-angiogenesis; anti-metastasis; anti-proliferation; anti-tumor growth; induces apoptosis, cell cycle arrest, DNA damage, ferroptosis	Activates caspase-3, -9, p38 MAPK pathway; Decreases metabolic capacity, mitochondrial membrane potential, PGE_2_ production; Down-regulates Bcl-2, CDC25A, COX-2, cyclin B, cyclin D1, cyclin E2, γ-H2AX, IGF-1R, Keap1, c-Myc, PAX7, RAD51, STAT3, UCA1, xCT; Enhances ROS production; Up-regulates ATF-4, ATM, ATR, Bax, BRCA1, E-cadherin, CHK1, CHK2, CHOP, HO-1, microRNA-16, microRNA-133, microRNA-206, Nrf2, p53	0.1–10 μM; 0–50 μM; 0–100 μM; 0–120 μM; 0–200 μM; 50 μM; 0–50 μg/ml; 0–160 mg/L; 0–200 mg/kg; 50 mg/kg; 50, 150 mg/kg; 100 mg/kg; 200 mg/kg	Cisplatin; Connexin-43; Paclitaxel	[669, 673, 675, 681, 683, 688–690, 693, 695, 967–971]
Wogonin	*Scutellaria baicalensis*, *Scutellaria amoena*, *Scutellaria rivularis*, *Anodendron affine* Druce	Breast cancer; gastric cancer; head and neck cancer; hepatocellular carcinoma; leukemia; lymphoma; melanoma; multiple myeloma; neuroblastoma; osteosarcoma; ovarian cancer; pancreatic cancer; NSCLC	MDA-MB-231, BGC-823, MFC, MGC-803, MKN45, SGC-7901, AMC-HN2, AMC-HN3, AMC-HN4, AMC-HN5, AMC-HN9, AMC-HN4-cisR, AMC-HN9-cisR, SNU-1041, SNU-1066, SNU-1076, Bel-7402, Hep3B, HepG2, SMMC-7721, K562, K562/A02, K562R, Raji, B16F10, RPMI 8226, U266, IMR-32, SK-N-BE2, CD133^+^ CAL72, A549, A2780, Colo-357, Panc-1	MDA-MB-231 xenograft mice; Raji xenograft mice; AMC-HN4-cisR xenograft mice; AMC-HN9-cisR xenograft mice; B16F10 xenograft mice; BGC-823 xenograft mice and zebrafish; MFC xenograft mice; RPMI 8226 xenograft mice	Anti-angiogenesis; anti-metastasis; anti-proliferation; anti-tumor growth; induces apoptosis, autophagy, cell cycle arrest, ER stress, mitochondrial dysfunction; reverses drug resistance	Activates caspase-3, -4, -8, -9, -12, PARP, IRE1α-dependent pathway; Decreases mitochondrial membrane potential; Down-regulates Akt, B7H1, Bcl-2, CDK4, CDK6, cyclin D1, cyclin E, EGFR, ERK, HIF-1α, IL-8, IκB, IKKα, Ki-67, MMP-2, MMP-9, c-Myc, PDK1, PI3K, Rac1, RAE-1ε, SGK1, ULK1, VEGF; Enhances calreticulin, HMGB1, cytochrome c release, ROS accumulation; Inhibits 5-LO/BLT2/ERK/IL-8/MMP-9, NF-κB pathways; Up-regulates ASK, Bax, Bid, GRP78, GRP94, IRE1α, JNK, p21, p53, PU.1, PUMA	0–20 μM; 0–40 μM; 0–50 μM; 0–60 μM; 0–80 μM; 0–100 μM; 0–150 μM; 0–200 μM; 40 μM; 50 μM; 0–40 μg/ml; 0–60 mg/kg; 0–80 mg/kg; 8 mg/kg; 20 mg/kg; 60 mg/kg; 12.5 ng/zebrafish	Cisplatin; Paclitaxel; Oxaliplatin; Sorafenib	[704, 708, 709, 716, 717, 719, 721, 725, 730, 731, 741, 742, 972–976]
β-Elemene	*Curcuma wenyujin* Y. H. Chen *et* C. Ling, *Rhizoma zedoariae*, *Curcuma Zedoary*	Bladder cancer; bone neoplasms; breast cancer; cervical cancer; gastric cancer; melanoma; NSCLC; osteosarcoma; thyroid cancer	PBC, Bcap37, MBA-MD-231, MCF-7, MCF-7/ADR, MCF-7/DOC, 5637, SiHa, T-24, BGC-823, MKN45, SGC-7901, B16F10, A549, H358, H460, H1299, H1650, H1975, Lewis, PC9, MG63, U2OS, FTC-133	A549 xenograft mice; B16F10 xenograft mice; BGC-823 xenograft mice; Lewis tumor-bearing mice; MG63 xenograft mice; U2OS xenograft mice	Anti-angiogenesis; anti-metastasis; anti-proliferation; anti-tumor growth; enhances radiosensitivity; induces apoptosis, autophagy, cell cycle arrest; reverses chemoresistance	Activates caspase-3, -7, -8, -9, -10; Down-regulates Akt, Bcl-2, β-catenin, CDC25C, CDK1, cyclin B1, cyclin D1, endostatin, ERK, DNMT1, MMP-2, MMP-3, MMP-9, MTA3, c-Myc, STAT3, Sp1, survivin, TCF7, TIMP-1, TIMP-2, VEGF; Enhances ROS accumulation; Induces polarization from M2 to M1 macrophages; Inhibits Wnt/β-catenin pathway; Up-regulates ATF-4, ATF-6, Bad, Bax, BTF, CHK2, CHOP, FoxO3a, IGFBP1, IRE1α, p15, p21, p53, Pak1, PAK1IP1, PERK, TOPIIα	0–25 μM; 0–1000 μM; 67.5–1000 μM; 0–40 μg/ml; 0–50 μg/ml; 0–120 μg/ml; 0–160 μg/ml; 0–200 μg/ml; 0–320 μg/ml; 0–500 μg/ml; 0–800 μg/ml; 0–0.16 mg/ml; 15, 30 μg/ml; 100 mg/ml; 1 mg/kg; 20 mg/kg; 50 mg/kg; 75 mg/kg; 200 mg/kg	Cisplatin; Paclitaxel; Rapamycin	[746, 747, 749, 752, 754, 755, 762, 977–987]
Cepharanthine	*Stephania cepharantha* Hayata, *Stephania japonica*	Choroidal melanoma; colorectal cancer; breast cancer; gastric cancer; leukemia; nasopharyngeal carcinoma; NSCLC; ovarian cancer; renal carcinoma	MEL15-1, COLO-205, HCT-116 HT-29, SW-620, MCF-7, MDA-MB-231, Jurkat T-cells, A549, H1299, HCC827, NCI-H1299, NCI-H1650, NCI-H1975, CNE-1, CNE-2, A2780, A2780/Taxol, CaOV-3, OVCAR3, Caki	A549 xenograft mice; NCI-H1975 xenograft mice	Anti-angiogenesis; anti-metastasis; anti-proliferation; anti-tumor growth; induces apoptosis, autophagy, cell cycle arrest; Reverses multi-drug resistance	Activates caspase-3, -9, PARP; Decreases mitochondrial membrane potential; Down-regulates Akt, Bcl-2, Bcl-xL, CDK4, cyclin A, cyclin D, c-FLIP, mTOR, p50, p52, survivin; Enhances cytochrome c release, ROS accumulation; Inhibits lysosomal cathepsin B and cathepsin D maturation, Akt/mTOR, NF-κB, pathways; Up-regulates Atg-7, Bak, Bax, Beclin1, DR5, p38 MAPK, Mcl-1, p21^Waf1/Cip1^, p53	0–15 μM; 0–20 μM; 0–80 μM; 0–100 μM; 0–120 μM; 2–8 μM; 4, 5 μM; 5–80 mM; 25 mg/kg; 50 mg/kg	Cisplatin; Dacomitinib; Paclitaxel; TRAIL	[777, 782–790, 794, 988]

We believe that as the evidence for safety and efficacy continues to develop, this will improve the understanding about the mechanistic actions and clinical potential of these compounds. Chinese herbal medicine will also serve as a huge community from which many promising compounds will be developed for clinical use.

## Data Availability

Not applicable.

## Abbreviations

4-PBA: 4-phenylbutyrate
5-LO: 5-lipoxygenase
ABCG2: ATP-binding cassette super-family G member 2
ACC: acetyl-CoA carboxylase
ACLY: ATP-citrate lyase
AEG-1: astrocyte elevated gene-1
AIF: apoptosis inducing factor
ALDOA: aldolase A
ALDH1: aldehyde dehydrogenase 1
AMPK: 5′AMP-activated protein kinase
AP-1: activator protein 1
Apaf-1: apoptotic protease activating factor 1
AQP1: aquaporin 1
AR: androgen receptor
ARIE: acute radiation-induced esophagitis
ART: artemisinin
ASK: apoptosis signal-regulating kinase
ATF-4: activating transcription factor 4
ATF-6: activating transcription factor 6
ATG4B: autophagy related 4B cysteine peptidase
Atg-5: autophagy related 5 protein
ATM: ataxia-telangiectasia mutated protein kinase
ATP: adenosine triphosphate
ATR: ataxia telangiectasia and Rad3-related protein
Axin2: axis inhibition protein 2
B7-H1: B7 homolog 1
B7-H3: B7 homolog 3
Bad: Bcl-2 associated agonist of cell death
Bak: Bcl-2 homologous antagonist killer
Bax: Bcl-2-associated X protein
BCAR1: breast cancer anti-estrogen resistance protein 1
Bcl-2: B cell lymphoma 2
Bcl-xL: B-cell lymphoma-extra large
Bex: brain-expressed and X-linked
Bid: BH3 interacting-domain death agonist
Bim: Bcl-2-like protein 11
BIP: binding immunoglobulin protein
BLT2: leukotriene B_4_ receptor 2
BMP7: bone morphogenetic protein 7
BRCA1: breast cancer type 1 susceptibility protein
BTF: Bcl-2-associated transcription factor 1
Ca^2+^: calcium
CAMKKβ: Ca^2+^/calmodulin-dependent protein kinase kinase β
Cav-1: caveolin-1
Cbl: casitas B-lineage lymphoma
CD: cluster of differentiation
CDC25A: cell division cycle 25A
CDC25C: cell division cycle 25C
CDK: cyclin-dependent kinase
CEH: cepharanthine hydrochloride
CEP: cepharanthine
CHK: checkpoint kinase 1
CHOP: C/EBP homologous protein
CIP2A: cancerous inhibitor of protein phosphatase 2A
CK1α: casein kinase 1α
CKS2: cyclin-dependent kinases regulatory subunit 2
COX-2: cyclooxygenase-2
COX IV: cytochrome c oxidase subunit 4
α-CP1: poly(rC)-binding protein 1
CSF: colony stimulating factor
CTGF: connective tissue growth factor
CTR1: copper transporter 1
CTTN: cortactin
CXCL-12: C–X–C motif chemokine 12
CXCR4: C–X–C chemokine receptor type 4
CYP2E1: cytochrome P450 2E1
DC: dendritic cell
DHA: dihydroartemisinin
DHCR24: 24-dehydrocholesterol reductase
DHFR: dihydrofolate reductase
DLL: delta-like canonical Notch ligand
DKK1: Dickkopf-related protein 1
DNA: deoxyribonucleic acid
DNMT: DNA (cytosine-5)-methyltransferase
DR4: death receptor 4
DR5: death receptor 5
Drp-1: dynamin-related protein 1
DUSP: dual-specificity phosphatase
Dvl2: dishevelled segment polarity protein 2
E2F1: E2F transcription factor 1
EBNA1: Epstein–Barr nuclear antigen 1
EF-Tu: elongation factor thermo unstable
EGCG: epigallocatechin gallate
EGFR: epidermal growth factor receptor
EGFR-TKI: epidermal growth factor receptor-tyrosine kinase inhibitor
EGR1: early growth response protein 1
ENY2: enhancer of yellow 2 transcription factor homolog
eIF2α: eukaryotic translation-initiation factor 2α
EphA2: ephrin type-A receptor 2
ER: endoplasmic reticulum
ERα: estrogen receptor α
ERK: extracellular signal-regulated kinase
Ets2: ETS proto-oncogene 2
EZH2: enhancer of zeste homolog 2
FABP4: fatty acid binding protein 4
FADD: Fas-associated protein with death domain
FAK: focal adhesion kinase
FasL: Fas ligand
bFGF: basic fibroblast growth factor
c-FLIP: FLICE-like inhibitory protein
FN: fibronectin
FoxM1: forkhead box protein M1
FoxO: forkhead box O
Foxp3: forkhead box P3
FRZB: frizzled-related protein
FUT4: fucosyltransferase 4
GA: gambogic acid
GADD45AQ: growth arrest and DNA damage-inducible 45
GLI1: glioma-associated oncogene homolog 1
GLUT-1: glucose transporter 1
GRB2: growth factor receptor-bound protein 2
GRP78: 78-kDa glucose-regulated protein
GRP94: 94-kDa glucose-regulated protein
GSH: glutathione
GSK3β: glycogen synthase kinase 3β
γ-H2AX: phosphorylated H2A histone family member X
HBP17: human fibroblast growth factor binding protein 1
HO-1: heme oxygenase 1
H_2_O_2_: hydrogen peroxide
HDAC: histone deacetylases
HER2: human epidermal growth factor receptor 2
HERC5: HECT domain and RCC-1-like domain-containing protein 5
HIF-1α: hypoxia-inducible factor 1α
HK2: hexokinase 2
HMGB1: high mobility group box 1
HNF4α: hepatocyte nuclear factor 4α
HRK: activator of apoptosis harakiri
HSF1: heat shock factor 1
HSP: heat shock protein
HUVEC: human umbilical vein endothelial cell
IAP: inhibitor of apoptosis protein
ICAD: apoptosis protease activating factor-1
ICAM-1: intercellular adhesion molecule 1
ICBP90: inverted CCAAT box-binding protein of 90 kDa
IDO: indoleamine 2,3-dioxygenase
IFN-γ: interferon-γ
IGFBP1: insulin-like growth factor-binding protein 1
IGF-1R: insulin-like growth factor 1 receptor
IκB: nuclear factor of kappa light polypeptide gene enhancer in B-cells inhibitor
IKK: IκB kinase
IL: interleukin
ILK: integrin-linked kinase
iNOS: inducible nitric oxide synthase
IRE1α: inositol-requiring enzyme 1α
ITG: integrin
Jak1: Janus kinase 1
Jak2: Janus kinase 2
JMJD3: Jumonji domain-containing protein D3
JMJD2B: Jumonji domain-containing protein 2B
JNK: c-Jun N-terminal kinase
K^+^: potassium
Keap1: Kelch-like ECH-associated protein 1
LEF1: lymphoid enhancer-binding factor 1
LeY: Lewis Y
Lig4: DNA ligase 4
LLC: Lewis lung carcinoma
LMP: Epstein–Barr virus latent membrane protein
LRP: low density lipoprotein receptor-related protein
LPS: lipopolysaccharide
LSD1: lysine-specific histone demethylase 1
MAPK: mitogen-activated protein kinase
Mcl-1: myeloid cell leukemia 1
MCT1: monocarboxylate transporter 1
MCP-1: monocyte chemoattractant protein 1
MD2: myeloid differentiation factor 2
MDM2: mouse double minute 2 homolog
MDR: multi-drug resistance
MDSCs: myeloid-derived suppressor cells
MEK: MAPK kinase
MGMT: *O*-6-methylguanine-DNA methyltransferase
MHC: major histocompatibility complex
Mfn: mitofusin
MKP-1: MAPK phosphatase 1
MMP: matrix metalloproteinase
MRP1: multi-drug resistance-associated protein 1
MST1: macrophage-stimulating 1
MTA3: metastasis-associated 1 family member 3
mTOR: mammalian target of rapamycin
NADPH: nicotinamide adenine dinucleotide phosphate oxidase
NAG-1: non-steroidal anti-inflammatory drug-activated gene 1
NF-κB: nuclear factor kappa-light-chain-enhancer of activated B cells
NK: natural killing
NKD2: naked cuticle 2
NQO1: NADPH quinone oxidoreductase 1
Nrf2: nuclear factor erythroid 2-related factor 2
NSCLC: non-small-cell lung carcinoma
Oct-4: octamer-binding transcription factor 4
Opa-1: optic atrophy protein 1
p70S6K: p70S6 kinase
u-PA: urokinase-type plasminogen activator
u-PAR: urokinase-type plasminogen activator receptor
PAI-1: plasminogen activator inhibitor 1
PAK1: p21-activated protein kinase 1
PAK1IP1: p21-activated protein kinase-interacting protein 1
PARP: poly (ADP-ribose) polymerase
PAX7: paired box 7
PCNA: proliferating cell nuclear antigen
PERK: protein kinase R-like endoplasmic reticulum kinase
PD-L1: programmed death-ligand 1
PDK1: pyruvate dehydrogenase kinase 1
PGE_2_: prostaglandin E_2_
P-gp: P-glycoprotein
PHLPP2: pH domain and leucine Rich repeat protein phosphatase 2
PLA2: phospholipase A2
PI3K: phosphoinositide 3-kinase
PKC-α: protein kinase Cα
PKD1: polycystin 1
PKM2: pyruvate kinase isozyme M2
PP2A: pyrophosphatase (inorganic) 2
PPARγ: peroxisome proliferator-activated receptor γ
PSA: prostate-specific antigen
PTEN: phosphatase and tensin homolog
PTTG-1: pituitary tumor-transforming gene 1 protein
PU.1: spleen focus forming virus proviral integration oncogene
PUMA: p53 upregulated modulator of apoptosis
PYK2: proline-rich tyrosine kinase 2
Rac1: Ras-related C3 botulinum toxin substrate 1
Rac2: Ras-related C3 botulinum toxin substrate 2
RAE-1ε: ribonucleic acid export 1ε
Rb: retinoblastoma-associated protein
RECK: reversion-inducing-cysteine-rich protein with kazal motifs
RECQL4: ATP-dependent DNA helicase Q4
RhoA: Ras homolog family member A
RIP: receptor-interacting serine/threonine protein
RIPK1: receptor-interacting serine/threonine protein kinase 1
RRM2: ribonucleotide reductase regulatory subunit M2
ROCK1: Rho-associated protein kinase 1
ROS: reactive oxygen species
S6: ribosomal protein S6
S6K: ribosomal protein S6 kinase
SERTAD1: SERTA domain-containing protein 1
SFRP1: secreted frizzled related protein 1
SFN: stratifin
SGK1: serum and glucocorticoid-regulated kinase 1
SHH: sonic hedgehog
SHP-1: Src homology region 2 domain-containing phosphatase 1
SIRT: sirtuin
Smac: second mitochondria-derived activator of caspase
SMOX: spermine oxidase
SOS1: son of sevenless homolog 1
SOD: superoxide dismutase
SOX2: sex determining region Y-box 2
Sp1: specificity protein 1
SREBP1: sterol regulatory element-binding protein 1
SSAT: spermidine/spermine N1-acetyltransferase
STAT: signal transducer and activator of transcription
SUV39H1: suppressor of variegation 3-9 homolog 1
Suz12: suppressor of zeste 12 protein homolog
TACE: TNF-α-converting enzyme
TAZ: tafazzin
TFAP2A: transcription factor AP-2-alpha
TCF: T-cell factor
TGF-β: transforming growth factor-β
Th1: T helper type 1 cell
Th2: T helper type 2 cell
Thy-1: THYmocyte differentiation antigen 1
TIMP: TIMP metallopeptidase inhibitor
TLR: toll-like receptor
TNF: tumor necrosis factor
TOPK: T-LAK cell-originated protein kinase
TOPIIα: DNA topoisomerase IIα
TRAF6: TNF receptor-associated factor 6
TRAIL: TNF‐related apoptosis‐inducing ligand
TROP2: tumor-associated calcium signal transducer 2
T_reg_: regulatory T cells
Trx: thioredoxin
TrxR: thioredoxin reductase
Tyro3: tyrosine-protein kinase receptor
UA: ursolic acid
UAL: UA-lipsomes
UCA1: urothelial cancer-associated 1
ULK-1: UNC-51-like autophagy activating kinase 1
UQCRC1: ubiquinol-cytochrome c reductase core protein 1
UTX: ubiquitously transcribed tetratricopeptide repeat protein X-linked
VEGF: vascular endothelial growth factor
VEGFR2: vascular endothelial growth factor receptor 2
VHL: von Hippel–Lindau tumor suppressor
XBP-1: X-box binding protein 1
xCT: solute carrier family 7 member 11
XIAP: X-linked inhibitor of apoptosis protein
WT1: Wilms tumor 1
YAP: Yes-associated protein 1
ZEB1: zinc finger E-box binding homeobox 1
ZEBRA: BamHI Z Epstein–Barr virus replication activator

## References

[1] Bray F, Ferlay J, Soerjomataram I, Siegel RL, Torre LA, Jemal A (2018). Global cancer statistics 2018: GLOBOCAN estimates of incidence and mortality worldwide for 36 cancers in 185 countries. CA Cancer J Clin.

[2] Wang S, Wu X, Tan M, Gong J, Tan W, Bian B (2012). Fighting fire with fire: poisonous Chinese herbal medicine for cancer therapy. J Ethnopharmacol.

[3] Zhong Z, Yu H, Wang S, Wang Y, Cui L (2018). Anti-cancer effects of Rhizoma Curcumae against doxorubicin-resistant breast cancer cells. Chin Med.

[4] Sang W, Zhong Z, Linghu K, Xiong W, Tse AKW, Cheang WS (2018). Siegesbeckia pubescens Makino inhibits Pam3CSK4-induced inflammation in RAW 264.7 macrophages through suppressing TLR1/TLR2-mediated NF-kappaB activation. Chin Med.

[5] Zhong Z, Zhang Q, Tao H, Sang W, Cui L, Qiang W (2019). Anti-inflammatory activities of Sigesbeckia glabrescens Makino: combined in vitro and in silico investigations. Chin Med.

[6] Shukla R, Chanda N, Zambre A, Upendran A, Katti K, Kulkarni RR (2012). Laminin receptor specific therapeutic gold nanoparticles (198AuNP-EGCg) show efficacy in treating prostate cancer. Proc Natl Acad Sci USA.

[7] Zhang J, Zhou F, Wu X, Zhang X, Chen Y, Zha BS (2012). Cellular pharmacokinetic mechanisms of adriamycin resistance and its modulation by 20(S)-ginsenoside Rh2 in MCF-7/Adr cells. Br J Pharmacol.

[8] Hsiao YT, Kuo CL, Chueh FS, Liu KC, Bau DT, Chung JG (2018). Curcuminoids induce reactive oxygen species and autophagy to enhance apoptosis in human oral cancer cells. Am J Chin Med.

[9] Wang N, Tan HY, Li L, Yuen MF, Feng Y (2015). Berberine and Coptidis Rhizoma as potential anticancer agents: recent updates and future perspectives. J Ethnopharmacol.

[10] Wang Z, Yin J, Li M, Shen J, Xiao Z, Zhao Y (2019). Combination of shikonin with paclitaxel overcomes multidrug resistance in human ovarian carcinoma cells in a P-gp-independent manner through enhanced ROS generation. Chin Med..

[11] Kharat M, Du Z, Zhang G, McClements DJ (2017). Physical and chemical stability of curcumin in aqueous solutions and emulsions: impact of pH, temperature, and molecular environment. J Agric Food Chem.

[12] Liu Y, Wang X, Zeng S, Zhang X, Zhao J, Zhang X (2018). The natural polyphenol curcumin induces apoptosis by suppressing STAT3 signaling in esophageal squamous cell carcinoma. J Exp Clin Cancer Res.

[13] Liu D, You M, Xu Y, Li F, Zhang D, Li X (2016). Inhibition of curcumin on myeloid-derived suppressor cells is requisite for controlling lung cancer. Int Immunopharmacol.

[14] Samarghandian S, Azimi-Nezhad M, Farkhondeh T, Samini F (2017). Anti-oxidative effects of curcumin on immobilization-induced oxidative stress in rat brain, liver and kidney. Biomed Pharmacother.

[15] Cai YY, Lin WP, Li AP, Xu JY (2013). Combined effects of curcumin and triptolide on an ovarian cancer cell line. Asian Pac J Cancer Prev.

[16] Jose A, Labala S, Venuganti VVK (2017). Co-delivery of curcumin and STAT3 siRNA using deformable cationic liposomes to treat skin cancer. J Drug Target.

[17] Ravindranathan P, Pasham D, Balaji U, Cardenas J, Gu JH, Toden S (2018). A combination of curcumin and oligomeric proanthocyanidins offer superior anti-tumorigenic properties in colorectal cancer. Sci Rep.

[18] Siddiqui FA, Prakasam G, Chattopadhyay S, Rehman AU, Padder RA, Ansari MA (2018). Curcumin decreases Warburg effect in cancer cells by down-regulating pyruvate kinase M2 via mTOR-HIF1 alpha inhibition. Sci Rep.

[19] Sivanantham B, Sethuraman S, Krishnan UM (2016). Combinatorial effects of curcumin with an anti-neoplastic agent on head and neck squamous cell carcinoma through the regulation of EGFR-ERK1/2 and apoptotic signaling pathways. Acs Comb Sci.

[20] Zou P, Zhang JR, Xia YQ, Kanchana K, Guo GL, Chen WB (2015). ROS generation mediates the anti-cancer effects of WZ35 via activating JNK and ER stress apoptotic pathways in gastric cancer. Oncotarget..

[21] Guan F, Ding Y, Zhang Y, Zhou Y, Li M, Wang C (2016). Curcumin suppresses proliferation and migration of MDA-MB-231 breast cancer cells through autophagy-dependent Akt degradation. PLoS ONE.

[22] Khan MA, Zafaryab M, Mehdi SH, Ahmad I, Rizvi MM (2016). Characterization and anti-proliferative activity of curcumin loaded chitosan nanoparticles in cervical cancer. Int J Biol Macromol.

[23] Silva G, Teixeira Lima F, Seba V, Mendes Lourenco AL, Lucas TG, de Andrade BV (2018). Curcumin analog CH-5 suppresses the proliferation, migration, and invasion of the human gastric cancer cell line HGC-27. Molecules (Basel, Switzerland).

[24] Starok M, Preira P, Vayssade M, Haupt K, Salome L, Rossi C (2015). EGFR inhibition by curcumin in cancer cells: a dual mode of action. Biomacromol.

[25] Gaikwad D, Shewale R, Patil V, Mali D, Gaikwad U, Jadhav N (2017). Enhancement in in vitro anti-angiogenesis activity and cytotoxicity in lung cancer cell by pectin-PVP based curcumin particulates. Int J Biol Macromol.

[26] Liao W, Xiang W, Wang FF, Wang R, Ding Y (2017). Curcumin inhibited growth of human melanoma A375 cells via inciting oxidative stress. Biomed Pharmacother.

[27] Chen P, Huang HP, Wang Y, Jin J, Long WG, Chen K (2019). Curcumin overcome primary gefitinib resistance in non-small-cell lung cancer cells through inducing autophagy-related cell death. J Exp Clin Cancer Res: CR..

[28] Zhu J, Zhao B, Xiong P, Wang C, Zhang J, Tian X (2018). Curcumin induces autophagy via inhibition of yes-associated protein (YAP) in human colon cancer cells. Med Sci Monit.

[29] Hu P, Ke C, Guo X, Ren P, Tong Y, Luo S (2019). Both glypican-3/Wnt/beta-catenin signaling pathway and autophagy contributed to the inhibitory effect of curcumin on hepatocellular carcinoma. Digest Liver Dis.

[30] Liu F, Gao S, Yang Y, Zhao X, Fan Y, Ma W (2018). Antitumor activity of curcumin by modulation of apoptosis and autophagy in human lung cancer A549 cells through inhibiting PI3K/Akt/mTOR pathway. Oncol Rep.

[31] Tong WH, Wang Q, Sun DH, Suo J (2016). Curcumin suppresses colon cancer cell invasion via AMPK-induced inhibition of NF-B, uPA activator and MMP9. Oncol Lett.

[32] Chen QY, Zheng Y, Jiao DM, Chen FY, Hu HZ, Wu YQ (2014). Curcumin inhibits lung cancer cell migration and invasion through Rac1-dependent signaling pathway. J Nutr Biochem.

[33] Cheng TS, Chen WC, Lin YY, Tsai CH, Liao CI, Shyu HY (2013). Curcumin-targeting pericellular serine protease matriptase role in suppression of prostate cancer cell invasion, tumor growth, and metastasis. Cancer Prevent Res (Philadelphia, Pa)..

[34] Hu C, Li M, Guo T, Wang S, Huang W, Yang K (2019). Anti-metastasis activity of curcumin against breast cancer via the inhibition of stem cell-like properties and EMT. Phytomedicine.

[35] Shimada K, Ushijima K, Suzuki C, Horiguchi M, Ando H, Akita T (2018). Pulmonary administration of curcumin inhibits B16F10 melanoma lung metastasis and invasion in mice. Cancer Chemother Pharmacol.

[36] Liu GY, Sun YZ, Zhou N, Du XM, Yang J, Guo SJ (2016). 3,3′-OH curcumin causes apoptosis in HepG2 cells through ROS-mediated pathway. Eur J Med Chem.

[37] Wang L, Chen X, Du Z, Li G, Chen M, Chen X (2017). Curcumin suppresses gastric tumor cell growth via ROS-mediated DNA polymerase gamma depletion disrupting cellular bioenergetics. J Exp Clin Cancer Res: CR..

[38] Larasati YA, Yoneda-Kato N, Nakamae I, Yokoyama T, Meiyanto E, Kato JY (2018). Curcumin targets multiple enzymes involved in the ROS metabolic pathway to suppress tumor cell growth. Sci Rep..

[39] Zhu GH, Dai HP, Shen Q, Ji O, Zhang Q, Zhai YL (2016). Curcumin induces apoptosis and suppresses invasion through MAPK and MMP signaling in human monocytic leukemia SHI-1 cells. Pharm Biol.

[40] Liao H, Wang Z, Deng Z, Ren H, Li X (2015). Curcumin inhibits lung cancer invasion and metastasis by attenuating GLUT1/MT1–MMP/MMP2 pathway. Int J Clin Exp Med.

[41] Sun MX, Yu F, Gong ML, Fan GL, Liu CX (2018). Effects of curcumin on the role of MMP-2 in endometrial cancer cell proliferation and invasion. Eur Rev Med Pharmacol Sci.

[42] Chen J, Zhang L, Shu Y, Chen L, Zhu M, Yao S (2017). Curcumin analogue CA15 exhibits anticancer effects on HEp-2 cells via targeting NF-kappaB. Biomed Res Int.

[43] Li G, Wang Z, Chong T, Yang J, Li H, Chen H (2017). Curcumin enhances the radiosensitivity of renal cancer cells by suppressing NF-kappaB signaling pathway. Biomed Pharmacother.

[44] Schwertheim S, Wein F, Lennartz K, Worm K, Schmid KW, Sheu-Grabellus SY (2017). Curcumin induces G2/M arrest, apoptosis, NF-kappaB inhibition, and expression of differentiation genes in thyroid carcinoma cells. J Cancer Res Clin Oncol.

[45] Montazeri M, Pilehvar-Soltanahmadi Y, Mohaghegh M, Panahi A, Khodi S, Zarghami N (2017). Antiproliferative and apoptotic effect of dendrosomal curcumin nanoformulation in P53 mutant and wide-type cancer cell lines. Anticancer Agents Med Chem.

[46] Fu H, Wang C, Yang D, Wei Z, Xu J, Hu Z (2018). Curcumin regulates proliferation, autophagy, and apoptosis in gastric cancer cells by affecting PI3K and P53 signaling. J Cell Physiol.

[47] Ciolac OA, Filippi A, Maru N, Popa M, Chifiriuc MC, Ganea C (2017). Reduction of the clonogenic potential and collapse of the mitochondrial membrane potential in A-431 epidermoid carcinoma cell line induced by curcumin. Rom Biotechnol Lett.

[48] Liu F, Gao S, Yang Y, Zhao X, Fan Y, Ma W (2017). Curcumin induced autophagy anticancer effects on human lung adenocarcinoma cell line A549. Oncol Lett.

[49] Zhao G, Han X, Zheng S, Li Z, Sha Y, Ni J (2016). Curcumin induces autophagy, inhibits proliferation and invasion by downregulating AKT/mTOR signaling pathway in human melanoma cells. Oncol Rep.

[50] Seo SU, Woo SM, Lee HS, Kim SH, Min KJ, Kwon TK (2018). mTORC1/2 inhibitor and curcumin induce apoptosis through lysosomal membrane permeabilization-mediated autophagy. Oncogene.

[51] Milano F, Mari L, van de Luijtgaarden W, Parikh K, Calpe S, Krishnadath KK (2013). Nano-curcumin inhibits proliferation of esophageal adenocarcinoma cells and enhances the T cell mediated immune response. Front Oncol.

[52] Kang S, Oh SC, Min BW, Lee DH (2018). Transglutaminase 2 regulates self-renewal and stem cell marker of human colorectal cancer stem cells. Anticancer Res.

[53] Zhao GJ, Lu ZQ, Tang LM, Wu ZS, Wang DW, Zheng JY (2012). Curcumin inhibits suppressive capacity of naturally occurring CD4 + CD25 + regulatory T cells in mice in vitro. Int Immunopharmacol.

[54] Luo F, Song X, Zhang Y, Chu Y (2011). Low-dose curcumin leads to the inhibition of tumor growth via enhancing CTL-mediated antitumor immunity. Int Immunopharmacol.

[55] Liao F, Liu L, Luo E, Hu J (2018). Curcumin enhances anti-tumor immune response in tongue squamous cell carcinoma. Arch Oral Biol.

[56] Khan MN, Haggag YA, Lane ME, McCarron PA, Tambuwala MM (2018). Polymeric nano-encapsulation of curcumin enhances its anti-cancer activity in breast (MDA-MB231) and lung (A549) cancer cells through reduction in expression of HIF-1 alpha and nuclear p65 (Rel A). Curr Drug Deliv.

[57] Al-Ani LA, Yehye WA, Kadir FA, Hashim NM, AlSaadi MA, Julkapli NM (2019). Hybrid nanocomposite curcumin-capped gold nanoparticle-reduced graphene oxide: anti-oxidant potency and selective cancer cytotoxicity. PLoS ONE.

[58] Chen X, Chen X, Zhang X, Wang L, Cao P, Rajamanickam V (2019). Curcuminoid B63 induces ROS-mediated paraptosis-like cell death by targeting TrxR1 in gastric cells. Redox Biol.

[59] Hajigholami S, Malekshahi ZV, Bodaghabadi N, Najafi F, Shirzad H, Sadeghizadeh M (2018). Nano packaged tamoxifen and curcumin; effective formulation against sensitive and resistant MCF-7 cells. Iran J Pharm Res.

[60] Liu L, Xiong XY, Shen M, Ru D, Gao P, Zhang XY (2018). Co-delivery of triptolide and curcumin for ovarian cancer targeting therapy via mPEG-DPPE/CaP nanoparticle. J Biomed Nanotechnol.

[61] Anirudhan TS, Nair AS, Bino SJ (2017). Nanoparticle assisted solvent selective transdermal combination therapy of curcumin and 5-flurouracil for efficient cancer treatment. Carbohyd Polym.

[62] Choudhury D, Ganguli A, Dastidar DG, Acharya BR, Das A, Chakrabarti G (2013). Apigenin shows synergistic anticancer activity with curcumin by binding at different sites of tubulin. Biochimie.

[63] Gao X, Wang B, Wu Q, Wei X, Zheng F, Men K (2015). Combined delivery and anti-cancer activity of paclitaxel and curcumin using polymeric micelles. J Biomed Nanotechnol.

[64] Guo J, Li W, Shi H, Xie X, Li L, Tang H (2013). Synergistic effects of curcumin with emodin against the proliferation and invasion of breast cancer cells through upregulation of miR-34a. Mol Cell Biochem.

[65] Hu B, Sun D, Sun C, Sun YF, Sun HX, Zhu QF (2015). A polymeric nanoparticle formulation of curcumin in combination with sorafenib synergistically inhibits tumor growth and metastasis in an orthotopic model of human hepatocellular carcinoma. Biochem Biophys Res Commun.

[66] Huang YF, Zhu DJ, Chen XW, Chen QK, Luo ZT, Liu CC (2017). Curcumin enhances the effects of irinotecan on colorectal cancer cells through the generation of reactive oxygen species and activation of the endoplasmic reticulum stress pathway. Oncotarget..

[67] Ji J, Wang HS, Gao YY, Sang LM, Zhang L (2014). Synergistic anti-tumor effect of KLF4 and curcumin in human gastric carcinoma cell line. Asian Pac J Cancer Prevent.

[68] Klippstein R, Bansal SS, Al-Jamal KT (2016). Doxorubicin enhances curcumin’s cytotoxicity in human prostate cancer cells in vitro by enhancing its cellular uptake. Int J Pharm.

[69] Qian H, Yang Y, Wang X (2011). Curcumin enhanced adriamycin-induced human liver-derived Hepatoma G2 cell death through activation of mitochondria-mediated apoptosis and autophagy. Eur J Pharm Sci..

[70] Roberts JL, Poklepovic A, Booth L (2017). Curcumin interacts with sildenafil to kill GI tumor cells via endoplasmic reticulum stress and reactive oxygen/nitrogen species. Oncotarget..

[71] Siddiqui RA, Harvey KA, Walker C, Altenburg J, Xu Z, Terry C (2013). Characterization of synergistic anti-cancer effects of docosahexaenoic acid and curcumin on DMBA-induced mammary tumorigenesis in mice. BMC Cancer..

[72] Lee HM, Patel V, Shyur LF, Lee WL (2016). Copper supplementation amplifies the anti-tumor effect of curcumin in oral cancer cells. Phytomedicine.

[73] Kondo A, Takeda T, Li B, Tsuiji K, Kitamura M, Wong TF (2013). Epigallocatechin-3-gallate potentiates curcumin’s ability to suppress uterine leiomyosarcoma cell growth and induce apoptosis. Int J Clin Oncol.

[74] Parsons HA, Baracos VE, Hong DS, Abbruzzese J, Bruera E, Kurzrock R (2016). The effects of curcumin (diferuloylmethane) on body composition of patients with advanced pancreatic cancer. Oncotarget..

[75] Greil R, Greil-Ressler S, Weiss L, Schonlieb C, Magnes T, Radl B (2018). A phase 1 dose-escalation study on the safety, tolerability and activity of liposomal curcumin (Lipocurc()) in patients with locally advanced or metastatic cancer. Cancer Chemother Pharmacol.

[76] Kanai M, Otsuka Y, Otsuka K, Sato M, Nishimura T, Mori Y (2013). A phase I study investigating the safety and pharmacokinetics of highly bioavailable curcumin (Theracurmin) in cancer patients. Cancer Chemother Pharmacol.

[77] Mahammedi H, Planchat E, Pouget M, Durando X, Cure H, Guy L (2016). The new combination docetaxel, prednisone and curcumin in patients with castration-resistant prostate cancer: a pilot phase II study. Oncology..

[78] Pang J, Zhang Z, Zheng TZ, Bassig BA, Mao C, Liu X (2016). Green tea consumption and risk of cardiovascular and ischemic related diseases: a meta-analysis. Int J Cardiol.

[79] Shin CM, Lee DH, Seo AY, Lee HJ, Kim SB, Son WC (2018). Green tea extracts for the prevention of metachronous colorectal polyps among patients who underwent endoscopic removal of colorectal adenomas: a randomized clinical trial. Clin Nutr (Edinburgh, Scotland)..

[80] Zhan W, Liu Y, Li DP, Liu Y (2016). Advancing insights on the anti-obesity biochemical mechanism of (−)-epigallocatechin gallate (EGCG) by inhibiting alpha-amylase activity. Rsc Adv.

[81] Lee S, Al Razqan GS, Kwon DH (2017). Antibacterial activity of epigallocatechin-3-gallate (EGCG) and its synergism with beta-lactam antibiotics sensitizing carbapenem-associated multidrug resistant clinical isolates of *Acinetobacter baumannii*. Phytomedicine.

[82] Zhu C, Xu Y, Liu ZH, Wan XC, Li DX, Tai LL (2018). The anti-hyperuricemic effect of epigallocatechin-3-gallate (EGCG) on hyperuricemic mice. Biomed Pharmacother.

[83] Steinmann J, Buer J, Pietschmann T, Steinmann E (2013). Anti-infective properties of epigallocatechin-3-gallate (EGCG), a component of green tea. Br J Pharmacol.

[84] Irimie AI, Braicu C, Zanoaga O, Pileczki V, Gherman C, Berindan-Neagoe I (2015). Epigallocatechin-3-gallate suppresses cell proliferation and promotes apoptosis and autophagy in oral cancer SSC-4 cells. OncoTargets Ther.

[85] Zhang JL, Lei Z, Huang ZN, Zhang X, Zhou YY, Luo ZL (2016). Epigallocatechin-3-gallate(EGCG) suppresses melanoma cell growth and metastasis by targeting TRAF6 activity. Oncotarget..

[86] Tudoran O, Soritau O, Balacescu O, Balacescu L, Braicu C, Rus M (2012). Early transcriptional pattern of angiogenesis induced by EGCG treatment in cervical tumour cells. J Cell Mol Med.

[87] Manjegowda MC, Deb G, Kumar N, Limaye AM (2015). Expression profiling of genes modulated by estrogen, EGCG or both in MCF-7 breast cancer cells. Genomics Data..

[88] Borutinskaite V, Virksaite A, Gudelyte G, Navakauskiene R (2018). Green tea polyphenol EGCG causes anti-cancerous epigenetic modulations in acute promyelocytic leukemia cells. Leuk Lymphoma.

[89] Yang CG, Du WF, Yang DG (2016). Inhibition of green tea polyphenol EGCG((−)-epigallocatechin-3-gallate) on the proliferation of gastric cancer cells by suppressing canonical wnt/beta-catenin signalling pathway. Int J Food Sci Nutr.

[90] Dettlaff K, Stawny M, Ogrodowczyk M, Jelinska A, Bednarski W, Watrobska-Swietlikowska D (2017). Formulation and characterization of EGCG for the treatment of superficial bladder cancer. Int J Mol Med.

[91] Ni J, Guo X, Wang H, Zhou T, Wang X (2018). Differences in the effects of EGCG on chromosomal stability and cell growth between normal and colon cancer cells. Molecules..

[92] Flores-Perez A, Marchat LA, Sanchez LL, Romero-Zamora D, Arechaga-Ocampo E, Ramirez-Torres N (2016). Differential proteomic analysis reveals that EGCG inhibits HDGF and activates apoptosis to increase the sensitivity of non-small cells lung cancer to chemotherapy. Proteomics Clin Appl.

[93] Deng YT, Lin JK (2011). EGCG inhibits the invasion of highly invasive CL1-5 lung cancer cells through suppressing MMP-2 expression via JNK signaling and induces G2/M arrest. J Agric Food Chem.

[94] Luo KW, Wei C, Lung WY, Wei XY, Cheng BH, Cai ZM (2017). EGCG inhibited bladder cancer SW780 cell proliferation and migration both in vitro and in vivo via down-regulation of NF-kappaB and MMP-9. J Nutr Biochem.

[95] Van Aller GS, Carson JD, Tang W, Peng H, Zhao L, Copeland RA (2011). Epigallocatechin gallate (EGCG), a major component of green tea, is a dual phosphoinositide-3-kinase/mTOR inhibitor. Biochem Biophys Res Commun.

[96] Shin YS, Kang SU, Park JK, Kim YE, Kim YS, Baek SJ (2016). Anti-cancer effect of (−)-epigallocatechin-3-gallate (EGCG) in head and neck cancer through repression of transactivation and enhanced degradation of beta-catenin. Phytomedicine.

[97] Huang CY, Han Z, Li X, Xie HH, Zhu SS (2017). Mechanism of EGCG promoting apoptosis of MCF-7 cell line in human breast cancer. Oncol Lett..

[98] Satoh M, Takemura Y, Hamada H, Sekido Y, Kubota S (2013). EGCG induces human mesothelioma cell death by inducing reactive oxygen species and autophagy. Cancer Cell Int.

[99] Tsai CY, Chen CY, Chiou YH, Shyu HW, Lin KH, Chou MC (2017). Epigallocatechin-3-gallate suppresses human herpesvirus 8 replication and induces ROS leading to apoptosis and autophagy in primary effusion lymphoma cells. Int J Mol Sci.

[100] Liu LC, Tsao TC, Hsu SR, Wang HC, Tsai TC, Kao JY (2012). EGCG inhibits transforming growth factor-beta-mediated epithelial-to-mesenchymal transition via the inhibition of Smad2 and Erk1/2 signaling pathways in nonsmall cell lung cancer cells. J Agric Food Chem.

[101] Sakamoto Y, Terashita N, Muraguchi T, Fukusato T, Kubota S (2013). Effects of epigallocatechin-3-gallate (EGCG) on A549 lung cancer tumor growth and angiogenesis. Biosci Biotechnol Biochem.

[102] Li M, Li JJ, Gu QH, An J, Cao LM, Yang HP (2016). EGCG induces lung cancer A549 cell apoptosis by regulating Ku70 acetylation. Oncol Rep.

[103] Hu Q, Chang X, Yan R, Rong C, Yang C, Cheng S (2015). (−)-Epigallocatechin-3-gallate induces cancer cell apoptosis via acetylation of amyloid precursor protein. Med Oncol (Northwood, London, England)..

[104] Onoda C, Kuribayashi K, Nirasawa S, Tsuji N, Tanaka M, Kobayashi D (2011). (−)-Epigallocatechin-3-gallate induces apoptosis in gastric cancer cell lines by down-regulating survivin expression. Int J Oncol.

[105] Wang J, Zhu C, Song D, Xia R, Yu W, Dang Y (2017). Epigallocatechin-3-gallate enhances ER stress-induced cancer cell apoptosis by directly targeting PARP16 activity. Cell Death Discov.

[106] Jwa M, Chang P (2012). PARP16 is a tail-anchored endoplasmic reticulum protein required for the PERK- and IRE1alpha-mediated unfolded protein response. Nat Cell Biol.

[107] Martinotti S, Ranzato E, Burlando B (2018). (−)-Epigallocatechin-3-gallate induces GRP78 accumulation in the ER and shifts mesothelioma constitutive UPR into proapoptotic ER stress. J Cell Physiol.

[108] Ogawa K, Hara T, Shimizu M, Nagano J, Ohno T, Hoshi M (2012). (−)-Epigallocatechin gallate inhibits the expression of indoleamine 2,3-dioxygenase in human colorectal cancer cells. Oncol Lett..

[109] Rawangkan A, Wongsirisin P, Namiki K, Iida K, Kobayashi Y, Shimizu Y (2018). Green tea catechin is an alternative immune checkpoint inhibitor that inhibits PD-L1 expression and lung tumor growth. Molecules.

[110] Zeng L, Yan J, Luo L, Ma M, Zhu H (2017). Preparation and characterization of (-)-Epigallocatechin-3-gallate (EGCG)-loaded nanoparticles and their inhibitory effects on Human breast cancer MCF-7 cells. Sci Rep..

[111] Wang JZ, Man GCW, Chan TH, Kwong J, Wang CC (2018). A prodrug of green tea polyphenol (-)-epigallocatechin-3-gallate (Pro-EGCG) serves as a novel angiogenesis inhibitor in endometrial cancer. Cancer Lett.

[112] Zhong Y, Chiou YS, Pan MH, Ho CT, Shahidi F (2012). Protective effects of epigallocatechin gallate (EGCG) derivatives on azoxymethane-induced colonic carcinogenesis in mice. J Funct Foods..

[113] Zhong Y, Shahidi F (2011). Lipophilized epigallocatechin gal late (EGCG) derivatives as novel antioxidants. J Agric Food Chem.

[114] Wu M, Jin JC, Jin P, Xu YQ, Yin JF, Qin DK (2017). Epigallocatechin gallate-beta-lactoglobulin nanoparticles improve the antitumor activity of EGCG for inducing cancer cell apoptosis. J Funct Foods..

[115] Radhakrishnan R, Kulhari H, Pooja D, Gudem S, Bhargava S, Shukla R (2016). Encapsulation of biophenolic phytochemical EGCG within lipid nanoparticles enhances its stability and cytotoxicity against cancer. Chem Phys Lipids.

[116] Liao BW, Ying H, Yu CH, Fan ZY, Zhang WH, Shi J (2016). (-)-Epigallocatechin gallate (EGCG)-nanoethosomes as a transdermal delivery system for docetaxel to treat implanted human melanoma cell tumors in mice. Int J Pharm.

[117] Liang J, Cao L, Zhang L, Wan XC (2014). Preparation, characterization, and in vitro antitumor activity of folate conjugated chitosan coated EGCG nanoparticles. Food Sci Biotechnol..

[118] Khan N, Bharali DJ, Adhami VM, Siddiqui IA, Cui HD, Shabana SM (2014). Oral administration of naturally occurring chitosan-based nanoformulated green tea polyphenol EGCG effectively inhibits prostate cancer cell growth in a xenograft model. Carcinogenesis.

[119] Wu SS, Sun K, Wang X, Wang DX, Wan XC, Zhang JS (2013). Protonation of epigallocatechin-3-gallate (EGCG) results in massive aggregation and reduced oral bioavailability of EGCG-dispersed selenium nanoparticles. J Agric Food Chem.

[120] Onoue S, Ochi M, Yamada S (2011). Development of (-)-epigallocatechin-3-gallate (EGCG)-loaded enteric microparticles with intestinal mucoadhesive property. Int J Pharm.

[121] Yang R, Liu YQ, Gao YJ, Wang YJ, Blanchard C, Zhou ZK (2017). Ferritin glycosylated by chitosan as a novel EGCG nano-carrier: structure, stability, and absorption analysis. Int J Biol Macromol.

[122] Wang XM, Jiang P, Wang PQ, Yang CS, Wang XR, Feng Q (2015). EGCG enhances cisplatin sensitivity by regulating expression of the copper and cisplatin influx transporter CTR1 in ovary cancer. PLoS ONE..

[123] Zhou DH, Wang X, Yang M, Shi X, Huang W, Feng Q (2013). Combination of low concentration of (-)-epigallocatechin gallate (EGCG) and curcumin strongly suppresses the growth of non-small cell lung cancer in vitro and in vivo through causing cell cycle arrest. Int J Mol Sci..

[124] Zhang Y, Wang SX, Ma JW, Li HY, Ye JC, Xie SM (2015). EGCG inhibits properties of glioma stem-like cells and synergizes with temozolomide through downregulation of P-glycoprotein inhibition. J Neurooncol..

[125] Hu F, Wei F, Wang Y, Wu B, Fang Y, Xiong B (2015). EGCG synergizes the therapeutic effect of cisplatin and oxaliplatin through autophagic pathway in human colorectal cancer cells. J Pharmacol Sci.

[126] Shin DM, Amin ARM, Wang D, Rahman MA, Nannapaneni S, Khuri FR (2012). 1187 molecular mechanism of synergistic anti-tumor activity by the combination of natural compounds Green tea (-)epigallocathetin-3-gallate (EGCG) and resveratrol for potential chemoprevention in head and neck cancer (HNC). Eur J Cancer.

[127] Wang W, Chen D, Zhu K (2018). SOX2OT variant 7 contributes to the synergistic interaction between EGCG and Doxorubicin to kill osteosarcoma via autophagy and stemness inhibition. J Exp Clin Cancer Res: CR..

[128] Kumazoe M, Tsukamoto S, Lesnick C, Kay NE, Yamada K, Shanafelt TD (2015). Vardenafil, a clinically available phosphodiesterase inhibitor, potentiates the killing effect of EGCG on CLL cells. Br J Haematol.

[129] Haque A, Rahman MA, Chen ZG, Saba NF, Khuri FR, Shin DM (2015). Combination of erlotinib and EGCG induces apoptosis of head and neck cancers through posttranscriptional regulation of Bim and Bcl-2. Apoptosis.

[130] Zhao HX, Zhu WQ, Jia L, Sun XR, Chen GX, Zhao XG (2016). Phase I study of topical epigallocatechin-3-gallate (EGCG) in patients with breast cancer receiving adjuvant radiotherapy. Br J Radiol.

[131] Zhao HX, Xie P, Li XL, Zhu WQ, Sun XD, Sun XR (2015). A prospective phase II trial of EGCG in treatment of acute radiation-induced esophagitis for stage III lung cancer. Radiother Oncol.

[132] Wang K, Feng X, Chai L, Cao S, Qiu F (2017). The metabolism of berberine and its contribution to the pharmacological effects. Drug Metab Rev.

[133] Zhu XF, Yue HD, Guo XF, Yang JY, Liu JS, Liu JT (2017). The preconditioning of berberine suppresses hydrogen peroxide-induced premature senescence via regulation of sirtuin 1. Oxid Med Cell Longevity..

[134] Chuang TY, Wu HL, Min J, Diamond M, Azziz R, Chen YH (2017). Berberine regulates the protein expression of multiple tumorigenesis-related genes in hepatocellular carcinoma cell lines. Cancer Cell Int.

[135] Liu J, Zhang X, Liu A, Liu S, Zhang L, Wu B (2013). Berberine induces apoptosis in p53-null leukemia cells by down-regulating XIAP at the post-transcriptional level. Cell Physiol Biochem.

[136] Park KS, Kim JB, Lee SJ, Bae J (2012). Berberine-induced growth inhibition of epithelial ovarian carcinoma cell lines. J Obstetr Gynaecol Res.

[137] Wen CJ, Wu LX, Fu LJ, Yu J, Zhang YW, Zhang X (2013). Genomic screening for targets regulated by berberine in breast cancer cells. Asian Pac J Cancer Prev.

[138] Luo X, Gu J, Zhu R, Feng M, Zhu X, Li Y (2014). Integrative analysis of differential miRNA and functional study of miR-21 by seed-targeting inhibition in multiple myeloma cells in response to berberine. BMC Syst Biol.

[139] Wang J, Kang M, Wen Q, Qin YT, Wei ZX, Xiao JJ (2017). Berberine sensitizes nasopharyngeal carcinoma cells to radiation through inhibition of Sp1 and EMT. Oncol Rep.

[140] Jiang SX, Qi B, Yao WJ, Gu CW, Wei XF, Zhao Y (2017). Berberine displays antitumor activity in esophageal cancer cells in vitro. World J Gastroenterol.

[141] Naveen CR, Gaikwad S, Agrawal-Rajput R (2016). Berberine induces neuronal differentiation through inhibition of cancer stemness and epithelial–mesenchymal transition in neuroblastoma cells. Phytomedicine.

[142] Park KS, Kim JB, Bae J, Park SY, Jee HG, Lee KE (2012). Berberine inhibited the growth of thyroid cancer cell lines 8505C and TPC1. Yonsei Med J.

[143] Wang XB, Wang N, Li HL, Liu M, Cao FJ, Yu XJ (2016). Up-regulation of PAI-1 and down-regulation of uPA are involved in suppression of invasiveness and motility of hepatocellular carcinoma cells by a natural compound berberine. Int J Mol Sci.

[144] Wang Y, Liu Q, Liu Z, Li B, Sun Z, Zhou H (2012). Berberine, a genotoxic alkaloid, induces ATM-Chk1 mediated G2 arrest in prostate cancer cells. Mutat Res.

[145] Kuo HP, Chuang TC, Yeh MH, Hsu SC, Way TD, Chen PY (2011). Growth suppression of HER2-overexpressing breast cancer cells by berberine via modulation of the HER2/PI3K/Akt signaling pathway. J Agric Food Chem.

[146] Xu LN, Lu BN, Hu MM, Xu YW, Han X, Qi Y (2012). Mechanisms involved in the cytotoxic effects of berberine on human colon cancer HCT-8 cells. Biocell.

[147] Shukla S, Rizvi F, Raisuddin S, Kakkar P (2014). FoxO proteins’ nuclear retention and BH3-only protein Bim induction evoke mitochondrial dysfunction-mediated apoptosis in berberine-treated HepG2 cells. Free Radical Biol Med.

[148] La X, Zhang L, Li Z, Yang P, Wang Y (2017). Berberine-induced autophagic cell death by elevating GRP78 levels in cancer cells. Oncotarget..

[149] Wang J, Qi Q, Feng Z, Zhang X, Huang B, Chen A (2016). Berberine induces autophagy in glioblastoma by targeting the AMPK/mTOR/ULK1-pathway. Oncotarget..

[150] Yao Z, Wan Y, Li B, Zhai C, Yao F, Kang Y (2018). Berberine induces mitochondrialmediated apoptosis and protective autophagy in human malignant pleural mesothelioma NCIH2452 cells. Oncol Rep.

[151] Kou Y, Li L, Li H, Tan YH, Li B, Wang K (2016). Berberine suppressed epithelial mesenchymal transition through cross-talk regulation of PI3K/AKT and RAR alpha/RAR beta in melanoma cells. Biochem Biophys Res Commun.

[152] Jin F, Xie T, Huang X, Zhao X (2018). Berberine inhibits angiogenesis in glioblastoma xenografts by targeting the VEGFR2/ERK pathway. Pharm Biol..

[153] Chu SC, Yu CC, Hsu LS, Chen KS, Su MY, Chen PN (2014). Berberine reverses epithelial-to-mesenchymal transition and inhibits metastasis and tumor-induced angiogenesis in human cervical cancer cells. Mol Pharmacol.

[154] Li X-L, Hu Y-J, Wang H, Yu B-Q, Yue H-L (2012). Molecular spectroscopy evidence of berberine binding to DNA: comparative binding and thermodynamic profile of intercalation. Biomacromolecules.

[155] Li J, Liu F, Jiang S, Liu J, Chen X, Zhang S (2018). Berberine hydrochloride inhibits cell proliferation and promotes apoptosis of non-small cell lung cancer via the suppression of the MMP2 and Bcl-2/Bax signaling pathways. Oncol Lett.

[156] Lin YS, Chiu YC, Tsai YH, Tsai YF, Wang JY, Tseng LM (2019). Different mechanisms involved in the berberine-induced antiproliferation effects in triple-negative breast cancer cell lines. J Cell Biochem.

[157] Chidambara Murthy KN, Jayaprakasha GK, Patil BS (2012). The natural alkaloid berberine targets multiple pathways to induce cell death in cultured human colon cancer cells. Eur J Pharmacol.

[158] Xie J, Xu Y, Huang X, Chen Y, Fu J, Xi M (2015). Berberine-induced apoptosis in human breast cancer cells is mediated by reactive oxygen species generation and mitochondrial-related apoptotic pathway. Tumour Biol.

[159] Park GB, Park SH, Kim D, Kim YS, Yoon SH, Hur DY (2016). Berberine induces mitochondrial apoptosis of EBV-transformed B cells through p53-mediated regulation of XAF1 and GADD45alpha. Int J Oncol.

[160] Park SH, Sung JH, Kim EJ, Chung N (2015). Berberine induces apoptosis via ROS generation in PANC-1 and MIA-PaCa2 pancreatic cell lines. Braz J Med Biol Res.

[161] Wang L, Liu L, Shi Y, Cao H, Chaturvedi R, Calcutt MW (2012). Berberine induces caspase-independent cell death in colon tumor cells through activation of apoptosis-inducing factor. PLoS ONE.

[162] Takeda K, Akira S (2015). Toll-like receptors. Curr Protoc Immunol..

[163] Cheng WE, Ying Chang M, Wei JY, Chen YJ, Maa MC, Leu TH (2015). Berberine reduces Toll-like receptor-mediated macrophage migration by suppression of Src enhancement. Eur J Pharmacol.

[164] Liu M, Wang X, Wang L, Ma X, Gong Z, Zhang S (2018). Targeting the IDO1 pathway in cancer: from bench to bedside. J Hematol Oncol.

[165] Wang YX, Pang WQ, Zeng QX, Deng ZS, Fan TY, Jiang JD (2018). Synthesis and biological evaluation of new berberine derivatives as cancer immunotherapy agents through targeting IDO1. Eur J Med Chem.

[166] Kim S, You D, Jeong Y, Yu J, Kim SW, Nam SJ (2018). Berberine down-regulates IL-8 expression through inhibition of the EGFR/MEK/ERK pathway in triple-negative breast cancer cells. Phytomedicine.

[167] Chen W, Miao YQ, Fan DJ, Yang SS, Lin X, Meng LK (2011). Bioavailability study of berberine and the enhancing effects of TPGS on intestinal absorption in rats. AAPS PharmSciTech.

[168] Alamzeb M, Khan MR, Mamoon Ur R, Ali S, Khan AA (2015). A new isoquinoline alkaloid with anti-microbial properties from *Berberis jaeschkeana* Schneid. var. jaeschkeana. Nat Prod Res.

[169] Zhu JX, Tang D, Feng L, Zheng ZG, Wang RS, Wu AG (2013). Development of self-microemulsifying drug delivery system for oral bioavailability enhancement of berberine hydrochloride. Drug Dev Ind Pharm.

[170] Xiong YX, Su HF, Lv P, Ma Y, Wang SK, Miao H (2015). A newly identified berberine derivative induces cancer cell senescence by stabilizing endogenous G-quadruplexes and sparking a DNA damage response at the telomere region. Oncotarget..

[171] Hashemi-Niasari F, Rabbani-Chadegani A, Razmi M, Fallah S (2018). Synergy of theophylline reduces necrotic effect of berberine, induces cell cycle arrest and PARP, HMGB1, Bcl-2 family mediated apoptosis in MDA-MB-231 breast cancer cells. Biomed Pharmacother.

[172] Palmieri A, Iapichino A, Cura F, Scapoli L, Carinci F, Mandrone M (2018). Pre-treatment with berberine enhances effect of 5-fluorouracil and cisplatin in HEP2 laryngeal cancer cell line. J Biol Regul Homeost Agents.

[173] Liu L, Fan J, Ai G, Liu J, Luo N, Li C (2019). Berberine in combination with cisplatin induces necroptosis and apoptosis in ovarian cancer cells. Biol Res.

[174] Hou D, Xu G, Zhang C, Li B, Qin J, Hao X (2017). Berberine induces oxidative DNA damage and impairs homologous recombination repair in ovarian cancer cells to confer increased sensitivity to PARP inhibition. Cell Death Dis.

[175] Efferth T (2017). From ancient herb to modern drug: artemisia annua and artemisinin for cancer therapy. Semin Cancer Biol.

[176] Efferth T, Zacchino S, Georgiev MI, Liu L, Wagner H, Panossian AJ (2015). Nobel prize for artemisinin brings phytotherapy into the spotlight. Phytomedicine..

[177] Tilaoui M, Mouse HA, Jaafari A, Zyad A (2014). Differential effect of artemisinin against cancer cell lines. Nat Prod Bioprospect.

[178] Zhang CZ, Zhang H, Yun J, Chen GG, San Lai PB (2012). Dihydroartemisinin exhibits antitumor activity toward hepatocellular carcinoma in vitro and in vivo. Biochem Pharmacol..

[179] Jiang F, Zhou JY, Zhang D, Liu MH, Chen YG (2018). Artesunate induces apoptosis and autophagy in HCT116 colon cancer cells, and autophagy inhibition enhances the artesunateinduced apoptosis. Int J Mol Med.

[180] Zhao X, Guo X, Yue W, Wang J, Yang J, Chen J (2017). Artemether suppresses cell proliferation and induces apoptosis in diffuse large B cell lymphoma cells. Exp Ther Med.

[181] Azimi Mohamadabadi M, Hassan ZM, Zavaran Hosseini A, Gholamzad M, Noori S, Mahdavi M (2013). Arteether exerts antitumor activity and reduces CD4 + CD25 + FOXP3 + T-reg cells in vivo. Iran J Immunol.

[182] Li Y, Sui H, Jiang C, Li S, Han Y, Huang P (2018). Dihydroartemisinin increases the sensitivity of photodynamic therapy via NF-kappaB/HIF-1alpha/VEGF pathway in esophageal cancer cell in vitro and in vivo. Cell Physiol Biochem.

[183] Lin R, Zhang Z, Chen L, Zhou Y, Zou P, Feng C (2016). Dihydroartemisinin (DHA) induces ferroptosis and causes cell cycle arrest in head and neck carcinoma cells. Cancer Lett.

[184] Tong Y, Liu Y, Zheng H, Zheng L, Liu W, Wu J (2016). Artemisinin and its derivatives can significantly inhibit lung tumorigenesis and tumor metastasis through Wnt/beta-catenin signaling. Oncotarget..

[185] Im E, Yeo C, Lee HJ, Lee EO (2018). Dihydroartemisinin induced caspase-dependent apoptosis through inhibiting the specificity protein 1 pathway in hepatocellular carcinoma SK-Hep-1 cells. Life Sci.

[186] Noori S, Hassan ZM, Farsam V (2014). Artemisinin as a Chinese medicine, selectively induces apoptosis in pancreatic tumor cell line. Chin J Integr Med.

[187] Lu M, Sun L, Zhou J, Yang J (2014). Dihydroartemisinin induces apoptosis in colorectal cancer cells through the mitochondria-dependent pathway. Tumour Biol.

[188] Cao Y, Feng YH, Gao LW, Li XY, Jin QX, Wang YY (2019). Artemisinin enhances the anti-tumor immune response in 4T1 breast cancer cells in vitro and in vivo. Int Immunopharmacol.

[189] Li X, Ba Q, Liu Y, Yue Q, Chen P, Li J (2017). Dihydroartemisinin selectively inhibits PDGFRalpha-positive ovarian cancer growth and metastasis through inducing degradation of PDGFRalpha protein. Cell Discov.

[190] Wang L, Li J, Shi X, Li S, Tang PM, Li Z (2019). Antimalarial Dihydroartemisinin triggers autophagy within HeLa cells of human cervical cancer through Bcl-2 phosphorylation at Ser70. Phytomedicine.

[191] Paccez JD, Duncan K, Sekar D, Correa RG, Wang Y, Gu X (2019). Dihydroartemisinin inhibits prostate cancer via JARID2/miR-7/miR-34a-dependent downregulation of Axl. Oncogenesis..

[192] Li J, Feng W, Lu H, Wei Y, Ma S, Wei L (2019). Artemisinin inhibits breast cancer-induced osteolysis by inhibiting osteoclast formation and breast cancer cell proliferation. J Cell Physiol.

[193] Tin AS, Sundar SN, Tran KQ, Park AH, Poindexter KM, Firestone GL (2012). Antiproliferative effects of artemisinin on human breast cancer cells requires the downregulated expression of the E2F1 transcription factor and loss of E2F1-target cell cycle genes. Anticancer Drugs.

[194] Dong F, Tian H, Yan S, Li L, Dong X, Wang F (2015). Dihydroartemisinin inhibits endothelial cell proliferation through the suppression of the ERK signaling pathway. Int J Mol Med.

[195] Dong F, Zhou X, Li C, Yan S, Deng X, Cao Z (2014). Dihydroartemisinin targets VEGFR2 via the NF-kappaB pathway in endothelial cells to inhibit angiogenesis. Cancer Biol Ther.

[196] Kumari K, Keshari S, Sengupta D, Sabat SC, Mishra SK (2017). Transcriptome analysis of genes associated with breast cancer cell motility in response to artemisinin treatment. BMC Cancer..

[197] Wang Z, Hu W, Zhang JL, Wu XH, Zhou HJ (2012). Dihydroartemisinin induces autophagy and inhibits the growth of iron-loaded human myeloid leukemia K562 cells via ROS toxicity. FEBS open bio..

[198] Thongchot S, Vidoni C, Ferraresi A, Loilome W, Yongvanit P, Namwat N (2018). Dihydroartemisinin induces apoptosis and autophagy-dependent cell death in cholangiocarcinoma through a DAPK1-BECLIN1 pathway. Mol Carcinog.

[199] Shi X, Wang L, Li X, Bai J, Li J, Li S (2017). Dihydroartemisinin induces autophagy-dependent death in human tongue squamous cell carcinoma cells through DNA double-strand break-mediated oxidative stress. Oncotarget..

[200] Li B, Bu S, Sun J, Guo Y, Lai D (2018). Artemisinin derivatives inhibit epithelial ovarian cancer cells via autophagy-mediated cell cycle arrest. Acta Biochim Biophys Sin.

[201] Odaka Y, Xu B, Luo Y, Shen T, Shang C, Wu Y (2014). Dihydroartemisinin inhibits the mammalian target of rapamycin-mediated signaling pathways in tumor cells. Carcinogenesis.

[202] Hu W, Chen SS, Zhang JL, Lou XE, Zhou HJ (2014). Dihydroartemisinin induces autophagy by suppressing NF-kappaB activation. Cancer Lett.

[203] Jiang J, Geng G, Yu X, Liu H, Gao J, An H (2016). Repurposing the anti-malarial drug dihydroartemisinin suppresses metastasis of non-small-cell lung cancer via inhibiting NF-kappaB/GLUT1 axis. Oncotarget..

[204] Li Z, Ding X, Wu H, Liu C (2019). Artemisinin inhibits angiogenesis by regulating p38 MAPK/CREB/TSP-1 signaling pathway in osteosarcoma. J Cell Biochem.

[205] Hu CJ, Zhou L, Cai Y (2014). Dihydroartemisinin induces apoptosis of cervical cancer cells via upregulation of RKIP and downregulation of bcl-2. Cancer Biol Ther.

[206] Qin G, Zhao C, Zhang L, Liu H, Quan Y, Chai L (2015). Dihydroartemisinin induces apoptosis preferentially via a Bim-mediated intrinsic pathway in hepatocarcinoma cells. Apoptosis.

[207] Ooko E, Saeed ME, Kadioglu O, Sarvi S, Colak M, Elmasaoudi K (2015). Artemisinin derivatives induce iron-dependent cell death (ferroptosis) in tumor cells. Phytomedicine.

[208] Zhou ZH, Chen FX, Xu WR, Qian H, Sun LQ, Lu XT (2013). Enhancement effect of dihydroartemisinin on human gammadelta T cell proliferation and killing pancreatic cancer cells. Int Immunopharmacol.

[209] Houh YK, Kim KE, Park S, Hur DY, Kim S, Kim D (2017). The effects of artemisinin on the cytolytic activity of natural killer (NK) cells. Int J Mol Sci..

[210] Manjili HK, Malvandi H, Mousavi MS, Attari E, Danafar H (2018). In vitro and in vivo delivery of artemisinin loaded PCL-PEG-PCL micelles and its pharmacokinetic study. Artif Cells Nanomed Biotechnol.

[211] Zhang CJ, Wang J, Zhang J, Lee YM, Feng G, Lim TK (2016). Mechanism-guided design and synthesis of a mitochondria-targeting artemisinin analogue with enhanced anticancer activity. Angew Chem Int Ed Engl.

[212] Magoulas GE, Tsigkou T, Skondra L, Lamprou M, Tsoukala P, Kokkinogouli V (2017). Synthesis of nomicronvel artemisinin dimers with polyamine linkers and evaluation of their potential as anticancer agents. Bioorg Med Chem.

[213] Li P, Yang S, Dou M, Chen Y, Zhang J, Zhao X (2014). Synergic effects of artemisinin and resveratrol in cancer cells. J Cancer Res Clin Oncol.

[214] Zhao C, Gao W, Chen T (2014). Synergistic induction of apoptosis in A549 cells by dihydroartemisinin and gemcitabine. Apoptosis.

[215] Feng X, Li L, Jiang H, Jiang K, Jin Y, Zheng J (2014). Dihydroartemisinin potentiates the anticancer effect of cisplatin via mTOR inhibition in cisplatin-resistant ovarian cancer cells: involvement of apoptosis and autophagy. Biochem Biophys Res Commun.

[216] Li YJ, Zhou JH, Du XX, de Jia X, Wu CL, Huang P (2014). Dihydroartemisinin accentuates the anti-tumor effects of photodynamic therapy via inactivation of NF-kappaB in Eca109 and Ec9706 esophageal cancer cells. Cell Physiol Biochem.

[217] Ericsson T, Blank A, von Hagens C, Ashton M, Abelo A (2014). Population pharmacokinetics of artesunate and dihydroartemisinin during long-term oral administration of artesunate to patients with metastatic breast cancer. Eur J Clin Pharmacol.

[218] Jansen FH, Adoubi I, Cnodder T, Jansen N, Tschulakow A (2011). First study of oral Artenimol-R in advanced cervical cancer: clinical benefit, tolerability and tumor markers. Anticancer Res..

[219] He NW, Zhao Y, Guo L, Shang J, Yang XB (2012). Antioxidant, antiproliferative, and pro-apoptotic activities of a saponin extract derived from the roots of Panax notoginseng (Burk.) F.H. Chen. J Med Food..

[220] Lu M, Fei Z, Zhang G (2018). Synergistic anticancer activity of 20(S)-Ginsenoside Rg3 and Sorafenib in hepatocellular carcinoma by modulating PTEN/Akt signaling pathway. Biomed Pharmacother.

[221] Wang X, Sun YY, Zhao C, Qu FZ, Zhao YQ (2017). 12-Chloracetyl-PPD, a novel dammarane derivative, shows anti-cancer activity via delay the progression of cell cycle G2/M phase and reactive oxygen species-mediate cell apoptosis. Eur J Pharmacol.

[222] Lee IS, Uh I, Kim KS, Kim KH, Park J, Kim Y (2016). Anti-inflammatory effects of ginsenoside Rg3 via NF-kappaB pathway in A549 cells and human asthmatic lung tissue. J Immunol Res.

[223] Lu Z, Xu H, Yu X, Wang Y, Huang L, Jin X (2018). 20(S)-Protopanaxadiol induces apoptosis in human hepatoblastoma HepG2 cells by downregulating the protein kinase B signaling pathway. Exp Ther Med..

[224] Liu Y, Fan D (2018). Ginsenoside Rg5 induces apoptosis and autophagy via the inhibition of the PI3K/Akt pathway against breast cancer in a mouse model. Food Funct..

[225] Wu Q, Deng J, Fan D, Duan Z, Zhu C, Fu R (2018). Ginsenoside Rh4 induces apoptosis and autophagic cell death through activation of the ROS/JNK/p53 pathway in colorectal cancer cells. Biochem Pharmacol.

[226] Leem DG, Shin JS, Kim KT, Choi SY, Lee MH, Lee KT (2018). Dammarane-type triterpene ginsenoside-Rg18 inhibits human non-small cell lung cancer A549 cell proliferation via G1 phase arrest. Oncol Lett..

[227] Zeng D, Wang J, Kong P, Chang C, Li J, Li J (2014). Ginsenoside Rg3 inhibits HIF-1α and VEGF expression in patient with acute leukemia via inhibiting the activation of PI3K/Akt and ERK1/2 pathways. Int J Clin Exp Pathol..

[228] Wang L, Gao S, Jiang W, Luo C, Xu M, Bohlin L (2014). Antioxidative dietary compounds modulate gene expression associated with apoptosis, DNA repair, inhibition of cell proliferation and migration. Int J Mol Sci.

[229] Kim YJ, Choi WI, Jeon BN, Choi KC, Kim K, Kim TJ (2014). Stereospecific effects of ginsenoside 20-Rg3 inhibits TGF-beta1-induced epithelial-mesenchymal transition and suppresses lung cancer migration, invasion and anoikis resistance. Toxicology.

[230] Yang J, Yuan D, Xing T, Su H, Zhang S, Wen J (2016). Ginsenoside Rh2 inhibiting HCT116 colon cancer cell proliferation through blocking PDZ-binding kinase/T-LAK cell-originated protein kinase. J Ginseng Res..

[231] Wang J-H, Nao J-F, Zhang M, He P (2014). 20(s)-ginsenoside Rg3 promotes apoptosis in human ovarian cancer HO-8910 cells through PI3K/Akt and XIAP pathways. Tumor Biol.

[232] Zhang Y, Liu QZ, Xing SP, Zhang JL (2016). Inhibiting effect of Endostar combined with ginsenoside Rg3 on breast cancer tumor growth in tumor-bearing mice. Asian Pac J Trop Med.

[233] Yuan Z, Jiang H, Zhu X, Liu X, Li J (2017). Ginsenoside Rg3 promotes cytotoxicity of Paclitaxel through inhibiting NF-κB signaling and regulating Bax/Bcl-2 expression on triple-negative breast cancer. Biomed Pharmacother.

[234] Jiang JW, Chen XM, Chen XH, Zheng SS (2011). Ginsenoside Rg3 inhibit hepatocellular carcinoma growth via intrinsic apoptotic pathway. World J Gastroenterol.

[235] Zhang C, Liu L, Yu Y, Chen B, Tang C, Li X (2012). Antitumor effects of ginsenoside Rg3 on human hepatocellular carcinoma cells. Mol Med Rep..

[236] Xie Q, Wen H, Zhang Q, Zhou W, Lin X, Xie D (2017). Inhibiting PI3K-AKt signaling pathway is involved in antitumor effects of ginsenoside Rg3 in lung cancer cell. Biomed Pharmacother.

[237] Sun HY, Lee JH, Han YS, Yoon YM, Yun CW, Kim JH (2016). Pivotal roles of ginsenoside Rg3 in tumor apoptosis through regulation of reactive oxygen species. Anticancer Res.

[238] Kim BM, Kim DH, Park JH, Surh YJ, Na HK (2014). Ginsenoside Rg3 inhibits constitutive activation of NF-kappaB signaling in human breast cancer (MDA-MB-231) cells: ERK and Akt as potential upstream targets. J Cancer Prev.

[239] Shan X, Fu YS, Aziz F, Wang XQ, Yan Q, Liu JW (2014). Ginsenoside Rg3 inhibits melanoma cell proliferation through down-regulation of histone deacetylase 3 (HDAC3) and increase of p53 acetylation. PLoS ONE.

[240] Li Y, Yang T, Li J, Hao HL, Wang SY, Yang J (2016). Inhibition of multiple myeloma cell proliferation by ginsenoside Rg3 via reduction in the secretion of IGF-1. Mol Med Rep.

[241] Tang YC, Zhang Y, Zhou J, Zhi Q, Wu MY, Gong FR (2018). Ginsenoside Rg3 targets cancer stem cells and tumor angiogenesis to inhibit colorectal cancer progression in vivo. Int J Oncol.

[242] Ge G, Yan Y, Cai H (2017). Ginsenoside Rh2 inhibited proliferation by inducing ROS mediated ER stress dependent apoptosis in lung cancer cells. Biol Pharm Bull.

[243] Li Q, Li B, Dong C, Wang Y, Li Q (2017). 20(S)-Ginsenoside Rh2 suppresses proliferation and migration of hepatocellular carcinoma cells by targeting EZH2 to regulate CDKN2A-2B gene cluster transcription. Eur J Pharmacol.

[244] Zhang Z, Du GJ, Wang CZ, Wen XD, Calway T, Li Z (2013). Compound K, a ginsenoside metabolite, inhibits colon cancer growth via multiple pathways including p53-p21 interactions. Int J Mol Sci.

[245] Zheng ZZ, Ming YL, Chen LH, Zheng GH, Liu SS, Chen QX (2014). Compound K-induced apoptosis of human hepatocellular carcinoma MHCC97-H cells in vitro. Oncol Rep.

[246] Zheng X, Chen W, Hou H, Li J, Li H, Sun X (2017). Ginsenoside 20(S)-Rg3 induced autophagy to inhibit migration and invasion of ovarian cancer. Biomed Pharmacother.

[247] Wang XJ, Zhou RJ, Zhang N, Jing Z (2019). 20(S)-ginsenoside Rg3 sensitizes human non-small cell lung cancer cells to icotinib through inhibition of autophagy. Eur J Pharmacol.

[248] Kim DG, Jung KH, Lee DG, Yoon JH, Choi KS, Kwon SW (2014). 20(S)-Ginsenoside Rg3 is a novel inhibitor of autophagy and sensitizes hepatocellular carcinoma to doxorubicin. Oncotarget..

[249] Yang Z, Zhao T, Liu H, Zhang L (2016). Ginsenoside Rh2 inhibits hepatocellular carcinoma through beta-catenin and autophagy. Sci Rep.

[250] Li C, Dong Y, Wang L, Xu G, Yang Q, Tang X (2018). Ginsenoside metabolite compound K induces apoptosis and autophagy in non-small cell lung cancer cells via AMPK-mTOR and JNK pathways. Biochem Cell Biol..

[251] Kim AD, Kang KA, Kim HS, Kim DH, Choi YH, Lee SJ (2013). A ginseng metabolite, compound K, induces autophagy and apoptosis via generation of reactive oxygen species and activation of JNK in human colon cancer cells. Cell Death Dis..

[252] Jiang J, Yuan Z, Sun Y, Bu Y, Li W, Fei Z (2017). Ginsenoside Rg3 enhances the anti-proliferative activity of erlotinib in pancreatic cancer cell lines by downregulation of EGFR/PI3K/Akt signaling pathway. Biomed Pharmacother.

[253] Pan XY, Guo H, Han J, Hao F, An Y, Xu Y (2012). Ginsenoside Rg3 attenuates cell migration via inhibition of aquaporin 1 expression in PC-3M prostate cancer cells. Eur J Pharmacol.

[254] Chen XP, Qian LL, Jiang H, Chen JH (2011). Ginsenoside Rg3 inhibits CXCR255 expression and related migrations in a breast cancer cell line. Int J Clin Oncol..

[255] Liu T, Zhao L, Hou H, Ding L, Chen W, Li X (2017). Ginsenoside 20(S)-Rg3 suppresses ovarian cancer migration via hypoxia-inducible factor 1 alpha and nuclear factor-kappa B signals. Tumour Biol.

[256] Wang YS, Lin Y, Li H, Li Y, Song Z, Jin YH (2017). The identification of molecular target of (20S) ginsenoside Rh2 for its anti-cancer activity. Sci Rep.

[257] Li B, Zhao J, Wang CZ, Searle J, He TC, Yuan CS (2011). Ginsenoside Rh2 induces apoptosis and paraptosis-like cell death in colorectal cancer cells through activation of p53. Cancer Lett.

[258] Zhang F, Li M, Wu X, Hu Y, Cao Y, Wang X (2015). 20(S)-ginsenoside Rg3 promotes senescence and apoptosis in gallbladder cancer cells via the p53 pathway. Drug Design Develop Ther.

[259] Wu R, Ru Q, Chen L, Ma B, Li C (2014). Stereospecificity of ginsenoside Rg3 in the promotion of cellular immunity in hepatoma H22-bearing mice. J Food Sci.

[260] Jiang Z, Yang Y, Yang Y, Zhang Y, Yue Z, Pan Z (2017). Ginsenoside Rg3 attenuates cisplatin resistance in lung cancer by downregulating PD-L1 and resuming immune. Biomed Pharmacother.

[261] Wang M, Yan SJ, Zhang HT, Li N, Liu T, Zhang YL (2017). Ginsenoside Rh2 enhances the antitumor immunological response of a melanoma mice model. Oncol Lett.

[262] Sun C, Yu Y, Wang L, Wu B, Xia L, Feng F (2016). Additive antiangiogenesis effect of ginsenoside Rg3 with low-dose metronomic temozolomide on rat glioma cells both in vivo and in vitro. J Exp Clin Cancer Res..

[263] Li Y, Wang Y, Niu K, Chen X, Xia L, Lu D (2016). Clinical benefit from EGFR-TKI plus ginsenoside Rg3 in patients with advanced non-small cell lung cancer harboring EGFR active mutation. Oncotarget..

[264] Zhou B, Yan Z, Liu R, Shi P, Qian S, Qu X (2016). Prospective study of transcatheter arterial chemoembolization (TACE) with ginsenoside Rg3 versus TACE alone for the treatment of patients with advanced hepatocellular carcinoma. Radiology.

[265] Shin SW, Kim SY, Park JW (2012). Autophagy inhibition enhances ursolic acid-induced apoptosis in PC3 cells. Biochem Biophys Acta.

[266] Kashyap D, Sharma A, Tuli HS, Punia S, Sharma AK (2016). Ursolic acid and oleanolic acid: pentacyclic terpenoids with promising anti-inflammatory activities. Recent Pat Inflammation Allergy Drug Discov.

[267] Zhang Z, Zhang H, Chen R, Wang Z (2018). Oral supplementation with ursolic acid ameliorates sepsis-induced acute kidney injury in a mouse model by inhibiting oxidative stress and inflammatory responses. Mol Med Rep.

[268] Zhao J, Chen J, Liu T, Fang J, Wan J, Zhao J (2012). Anti-viral effects of urosolic acid on guinea pig cytomegalovirus in vitro. J Huazhong Univ Sci Technol Med Sci.

[269] Kaewthawee N, Brimson S (2013). The effects of ursolic acid on cytokine production via the MAPK pathways in leukemic T-cells. EXCLI J.

[270] Wang CM, Yeh KL, Tsai SJ, Jhan YL, Chou CH (2017). Anti-proliferative activity of triterpenoids and sterols isolated from alstonia scholaris against non-small-cell lung carcinoma cells. Molecules (Basel, Switzerland)..

[271] Li Q, Zhao W, Zeng X, Hao Z (2018). Ursolic acid attenuates atherosclerosis in ApoE(-/-) mice: role of LOX-1 Mediated by ROS/NF-kappaB pathway. Molecules (Basel, Switzerland)..

[272] Wang XT, Gong Y, Zhou B, Yang JJ, Cheng Y, Zhao JG (2018). Ursolic acid ameliorates oxidative stress, inflammation and fibrosis in diabetic cardiomyopathy rats. Biomed Pharmacother.

[273] Li J, Liang X, Yang X (2012). Ursolic acid inhibits growth and induces apoptosis in gemcitabine-resistant human pancreatic cancer via the JNK and PI3K/Akt/NF-kappaB pathways. Oncol Rep.

[274] Zheng QY, Li PP, Jin FS, Yao C, Zhang GH, Zang T (2013). Ursolic acid induces ER stress response to activate ASK1-JNK signaling and induce apoptosis in human bladder cancer T24 cells. Cell Signal.

[275] Liu T, Ma H, Shi W, Duan J, Wang Y, Zhang C (2017). Inhibition of STAT3 signaling pathway by ursolic acid suppresses growth of hepatocellular carcinoma. Int J Oncol.

[276] Huang CY, Lin CY, Tsai CW, Yin MC (2011). Inhibition of cell proliferation, invasion and migration by ursolic acid in human lung cancer cell lines. Toxicol In Vitro.

[277] Meng Y, Lin ZM, Ge N, Zhang DL, Huang J, Kong F (2015). Ursolic acid induces apoptosis of prostate cancer cells via the PI3K/Akt/mTOR pathway. Am J Chin Med.

[278] Zhu Z, Qian Z, Yan Z, Zhao C, Wang H, Ying G (2013). A phase I pharmacokinetic study of ursolic acid nanoliposomes in healthy volunteers and patients with advanced solid tumors. Int J Nanomed.

[279] Luo J, Hu YL, Wang H (2017). Ursolic acid inhibits breast cancer growth by inhibiting proliferation, inducing autophagy and apoptosis, and suppressing inflammatory responses via the PI3K/AKT and NF-kappaB signaling pathways in vitro. Exp Ther Med.

[280] Qian Z, Wang X, Song Z, Zhang H, Zhou S, Zhao J (2015). A phase I trial to evaluate the multiple-dose safety and antitumor activity of ursolic acid liposomes in subjects with advanced solid tumors. Biomed Res Int.

[281] Shan JZ, Xuan YY, Ruan SQ, Sun M (2011). Proliferation-inhibiting and apoptosis-inducing effects of ursolic acid and oleanolic acid on multi-drug resistance cancer cells in vitro. Chin J Integr Med.

[282] Wang JS, Ren TN, Xi T (2012). Ursolic acid induces apoptosis by suppressing the expression of FoxM1 in MCF-7 human breast cancer cells. Med Oncol (Northwood, London, England)..

[283] Shanmugam MK, Rajendran P, Li F, Nema T, Vali S, Abbasi T (2011). Ursolic acid inhibits multiple cell survival pathways leading to suppression of growth of prostate cancer xenograft in nude mice. J Mol Med.

[284] Park JH, Kwon HY, Sohn EJ, Kim KA, Kim B, Jeong SJ (2013). Inhibition of Wnt/beta-catenin signaling mediates ursolic acid-induced apoptosis in PC-3 prostate cancer cells. Pharmacol Rep.

[285] Shin SW, Park JW (2013). Ursolic acid sensitizes prostate cancer cells to TRAIL-mediated apoptosis. Biochem Biophys Acta.

[286] Xavier CP, Lima CF, Pedro DF, Wilson JM, Kristiansen K, Pereira-Wilson C (2013). Ursolic acid induces cell death and modulates autophagy through JNK pathway in apoptosis-resistant colorectal cancer cells. J Nutr Biochem..

[287] Prasad S, Yadav VR, Sung B, Reuter S, Kannappan R, Deorukhkar A (2012). Ursolic acid inhibits growth and metastasis of human colorectal cancer in an orthotopic nude mouse model by targeting multiple cell signaling pathways: chemosensitization with capecitabine. Clin Cancer Res.

[288] Hassan L, Pinon A, Limami Y, Seeman J, Fidanzi-Dugas C, Martin F (2016). Resistance to ursolic acid-induced apoptosis through involvement of melanogenesis and COX-2/PGE2 pathways in human M4Beu melanoma cancer cells. Exp Cell Res.

[289] Kim KH, Seo HS, Choi HS, Choi I, Shin YC, Ko SG (2011). Induction of apoptotic cell death by ursolic acid through mitochondrial death pathway and extrinsic death receptor pathway in MDA-MB-231 cells. Arch Pharmacal Res.

[290] Leng S, Hao Y, Du D, Xie S, Hong L, Gu H (2013). Ursolic acid promotes cancer cell death by inducing Atg5-dependent autophagy. Int J Cancer.

[291] Lewinska A, Adamczyk-Grochala J, Kwasniewicz E, Deregowska A, Wnuk M (2017). Ursolic acid-mediated changes in glycolytic pathway promote cytotoxic autophagy and apoptosis in phenotypically different breast cancer cells. Apoptosis.

[292] Lin CW, Chin HK, Lee SL, Chiu CF, Chung JG, Lin ZY (2019). Ursolic acid induces apoptosis and autophagy in oral cancer cells. Environ Toxicol..

[293] Saraswati S, Agrawal SS, Alhaider AA (2013). Ursolic acid inhibits tumor angiogenesis and induces apoptosis through mitochondrial-dependent pathway in Ehrlich ascites carcinoma tumor. Chem Biol Interact.

[294] Guo JL, Han T, Bao L, Li XM, Ma JQ, Tang LP (2019). Ursolic acid promotes the apoptosis of cervical cancer cells by regulating endoplasmic reticulum stress. J Obstetr Gynaecol Res.

[295] Wu CC, Cheng CH, Lee YH, Chang IL, Chen HY, Hsieh CP (2016). Ursolic acid triggers apoptosis in human osteosarcoma cells via caspase activation and the ERK1/2 MAPK pathway. J Agric Food Chem.

[296] Nam H, Kim MM (2013). Ursolic acid induces apoptosis of SW480 cells via p53 activation. Food Chem Toxicol.

[297] Zhang X, Song X, Yin S, Zhao C, Fan L, Hu H (2016). p21 induction plays a dual role in anti-cancer activity of ursolic acid. Exp Biol Med.

[298] Biswas S, Mukherjee PK, Harwansh RK, Bannerjee S, Bhattacharjee P (2019). Enhanced bioavailability and hepatoprotectivity of optimized ursolic acid-phospholipid complex. Drug Dev Ind Pharm.

[299] Rocha TG, Lopes SC, Cassali GD, Ferreira E, Veloso ES, Leite EA (2016). Evaluation of antitumor activity of long-circulating and pH-sensitive liposomes containing ursolic acid in animal models of breast tumor and gliosarcoma. Integr Cancer Ther.

[300] Zhao T, Liu Y, Gao Z, Gao D, Li N, Bian Y (2015). Self-assembly and cytotoxicity study of PEG-modified ursolic acid liposomes. Mater Sci Eng C Mater Biol Appl.

[301] Lopes S, Novais M, Teixeira C, Honorato-Sampaio K, Pereira M, Ferreira L (2013). Preparation, physicochemical characterization, and cell viability evaluation of long-circulating and pH-sensitive liposomes containing ursolic acid. Biomed Res Int.

[302] Wang M, Zhao T, Liu Y, Wang Q, Xing S, Li L (2017). Ursolic acid liposomes with chitosan modification: promising antitumor drug delivery and efficacy. Mater Sci Eng C Mater Biol Appl..

[303] de Oliveira Eloy J, Saraiva J, de Albuquerque S, Marchetti JM (2012). Solid dispersion of ursolic acid in Gelucire 50/13: a strategy to enhance drug release and trypanocidal activity. AAPS PharmSciTech.

[304] Jamal M, Imam SS, Aqil M, Amir M, Mir SR, Mujeeb M (2015). Transdermal potential and anti-arthritic efficacy of ursolic acid from niosomal gel systems. Int Immunopharmacol.

[305] Li T, Chen X, Liu Y, Fan L, Lin L, Xu Y (2017). pH-Sensitive mesoporous silica nanoparticles anticancer prodrugs for sustained release of ursolic acid and the enhanced anti-cancer efficacy for hepatocellular carcinoma cancer. Eur J Pharm Sci..

[306] Wu CC, Huang YF, Hsieh CP, Chueh PJ, Chen YL (2016). Combined use of zoledronic acid augments ursolic acid-induced apoptosis in human osteosarcoma cells through enhanced oxidative stress and autophagy. Molecules (Basel, Switzerland)..

[307] Tremmel L, Rho O, Slaga TJ, DiGiovanni J (2019). Inhibition of skin tumor promotion by TPA using a combination of topically applied ursolic acid and curcumin. Mol Carcinog.

[308] Zong L, Cheng G, Liu S, Pi Z, Liu Z, Song F (2019). Reversal of multidrug resistance in breast cancer cells by a combination of ursolic acid with doxorubicin. J Pharm Biomed Anal.

[309] Wang XH, Zhou SY, Qian ZZ, Zhang HL, Qiu LH, Song Z (2013). Evaluation of toxicity and single-dose pharmacokinetics of intravenous ursolic acid liposomes in healthy adult volunteers and patients with advanced solid tumors. Expert Opin Drug Metab Toxicol.

[310] Kim BR, Seo HS, Ku JM, Kim GJ, Jeon CY, Park JH (2013). Silibinin inhibits the production of pro-inflammatory cytokines through inhibition of NF-kappaB signaling pathway in HMC-1 human mast cells. Inflammation Res.

[311] Bai D, Jin G, Yin S, Zou D, Zhu Q, Yang Z (2017). Antioxidative and anti-apoptotic roles of silibinin in reversing learning and memory deficits in APP/PS1 mice. Neurochem Res.

[312] Harati K, Behr B, Wallner C, Daigeler A, Hirsch T, Jacobsen F (2017). Antiproliferative activity of epigallocatechin3gallate and silibinin on soft tissue sarcoma cells. Mol Med Rep..

[313] Yun DG, Lee DG (2017). Assessment of silibinin as a potential antifungal agent and investigation of its mechanism of action. IUBMB Life.

[314] Vimalraj S, Rajalakshmi S, Saravanan S, Raj-Preeth D, Lav R, Shairam M (2018). Synthesis and characterization of zinc–silibinin complexes: a potential bioactive compound with angiogenic, and antibacterial activity for bone tissue engineering. Coll Surf B Biointerfaces..

[315] Fernandes V, Sharma D, Kalia K, Tiwari V (2018). Neuroprotective effects of silibinin: an in silico and in vitro study. Int J Neurosci.

[316] Faridnia R, Kalani H, Fakhar M, Akhtari J (2018). Investigating in vitro anti-leishmanial effects of silibinin and silymarin on *Leishmania major*. Ann Parasitol.

[317] Jiang C, Jin S, Jiang Z, Wang J (2016). Inhibitory effects of silibinin on proliferation and lung metastasis of human high metastasis cell line of salivary gland adenoid cystic carcinoma via autophagy induction. OncoTargets Ther.

[318] Ma Z, Liu W, Zeng J, Zhou J, Guo P, Xie H (2015). Silibinin induces apoptosis through inhibition of the mTOR-GLI1-BCL2 pathway in renal cell carcinoma. Oncol Rep.

[319] Brandon-Warner E, Eheim AL, Foureau DM, Walling TL, Schrum LW, McKillop IH (2012). Silibinin (Milk Thistle) potentiates ethanol-dependent hepatocellular carcinoma progression in male mice. Cancer Lett.

[320] Gandara L, Sandes E, Di Venosa G, Prack Mc Cormick B, Rodriguez L, Mamone L (2014). The natural flavonoid silybin improves the response to photodynamic therapy of bladder cancer cells. J Photochem Photobiol B..

[321] Sun HP, Su JH, Meng QS, Yin Q, Zhang ZW, Yu HJ (2016). Silibinin and indocyanine green-loaded nanoparticles inhibit the growth and metastasis of mammalian breast cancer cells in vitro. Acta Pharmacol Sin.

[322] Nambiar D, Prajapati V, Agarwal R, Singh RP (2013). In vitro and in vivo anticancer efficacy of silibinin against human pancreatic cancer BxPC-3 and PANC-1 cells. Cancer Lett.

[323] Akhtar R, Ali M, Mahmood S, Sanyal SN (2014). Anti-proliferative action of silibinin on human colon adenomatous cancer HT-29 cells. Nutr Hosp.

[324] Pashaei-Asl F, Pashaei-Asl R, Khodadadi K, Akbarzadeh A, Ebrahimie E, Pashaiasl M (2018). Enhancement of anticancer activity by silibinin and paclitaxel combination on the ovarian cancer. Artif Cells Nanomed Biotechnol.

[325] Amirsaadat S, Pilehvar-Soltanahmadi Y, Zarghami F, Alipour S, Ebrahimnezhad Z, Zarghami N (2017). Silibinin-loaded magnetic nanoparticles inhibit hTERT gene expression and proliferation of lung cancer cells. Artif Cells Nanomed Biotechnol.

[326] Choi ES, Oh S, Jang B, Yu HJ, Shin JA, Cho NP (2017). Silymarin and its active component silibinin act as novel therapeutic alternatives for salivary gland cancer by targeting the ERK1/2-Bim signaling cascade. Cell Oncol (Dordrecht)..

[327] Deep G, Kumar R, Nambiar DK, Jain AK, Ramteke AM, Serkova NJ (2017). Silibinin inhibits hypoxia-induced HIF-1alpha-mediated signaling, angiogenesis and lipogenesis in prostate cancer cells: in vitro evidence and in vivo functional imaging and metabolomics. Mol Carcinog.

[328] Lu S, Zhang Z, Chen M, Li C, Liu L, Li Y (2017). Silibinin inhibits the migration and invasion of human gastric cancer SGC7901 cells by downregulating MMP-2 and MMP-9 expression via the p38MAPK signaling pathway. Oncol Lett.

[329] Kim S, Jeon M, Lee J, Han J, Oh SJ, Jung T (2014). Induction of fibronectin in response to epidermal growth factor is suppressed by silibinin through the inhibition of STAT3 in triple negative breast cancer cells. Oncol Rep.

[330] Ge Y, Zhang Y, Chen Y, Li Q, Chen J, Dong Y (2011). Silibinin causes apoptosis and cell cycle arrest in some human pancreatic cancer cells. Int J Mol Sci.

[331] Woo SM, Min KJ, Kim S, Park JW, Kim DE, Chun KS (2014). Silibinin induces apoptosis of HT29 colon carcinoma cells through early growth response-1 (EGR-1)-mediated non-steroidal anti-inflammatory drug-activated gene-1 (NAG-1) up-regulation. Chem Biol Interact.

[332] Si L, Liu W, Hayashi T, Ji Y, Fu J, Nie Y (2019). Silibinin-induced apoptosis of breast cancer cells involves mitochondrial impairment. Arch Biochem Biophys.

[333] Jiang K, Wang W, Jin X, Wang Z, Ji Z, Meng G (2015). Silibinin, a natural flavonoid, induces autophagy via ROS-dependent mitochondrial dysfunction and loss of ATP involving BNIP3 in human MCF7 breast cancer cells. Oncol Rep.

[334] Liu W, Otkur W, Li L, Wang Q, He H, Ye Y (2013). Autophagy induced by silibinin protects human epidermoid carcinoma A431 cells from UVB-induced apoptosis. J Photochem Photobiol, B.

[335] Bai ZL, Tay V, Guo SZ, Ren J, Shu MG (2018). Silibinin induced human glioblastoma cell apoptosis concomitant with autophagy through simultaneous inhibition of mTOR and YAP. Biomed Res Int.

[336] Zheng N, Liu L, Liu WW, Li F, Hayashi T, Tashiro SI (2017). Crosstalk of ROS/RNS and autophagy in silibinin-induced apoptosis of MCF-7 human breast cancer cells in vitro. Acta Pharmacol Sin.

[337] Kim SH, Kim KY, Yu SN, Park SK, Choi HD, Ji JH (2015). Autophagy inhibition enhances silibinin-induced apoptosis by regulating reactive oxygen species production in human prostate cancer PC-3 cells. Biochem Biophys Res Commun.

[338] Zhang Y, Ge Y, Chen Y, Li Q, Chen J, Dong Y (2012). Cellular and molecular mechanisms of silibinin induces cell-cycle arrest and apoptosis on HeLa cells. Cell Biochem Funct.

[339] Zhang X, Liu J, Zhang P, Dai L, Wu Z, Wang L (2018). Silibinin induces G1 arrest, apoptosis and JNK/SAPK upregulation in SW1990 human pancreatic cancer cells. Oncol Lett.

[340] Jahanafrooz Z, Motameh N, Bakhshandeh B (2016). Comparative evaluation of silibinin effects on cell cycling and apoptosis in human breast cancer MCF-7 and T47D cell lines. Asian Pac J Cancer Prevent.

[341] Zhang Y, Li Q, Ge Y, Chen Y, Chen J, Dong Y (2013). Silibinin triggers apoptosis and cell-cycle arrest of SGC7901 cells. Phytother Res.

[342] Liang L, Li L, Zeng J, Gao Y, Chen YL, Wang ZQ (2012). Inhibitory effect of silibinin on EGFR signal-induced renal cell carcinoma progression via suppression of the EGFR/MMP-9 signaling pathway. Oncol Rep.

[343] Liu W, Otkur W, Li L, Wang Q, He H, Zang L (2013). Interference of silibinin with IGF-1R signalling pathways protects human epidermoid carcinoma A431 cells from UVB-induced apoptosis. Biochem Biophys Res Commun.

[344] Oh SJ, Jung SP, Han J, Kim S, Kim JS, Nam SJ (2013). Silibinin inhibits TPA-induced cell migration and MMP-9 expression in thyroid and breast cancer cells. Oncol Rep.

[345] Yousefi M, Ghaffari SH, Zekri A, Hassani S, Alimoghaddam K, Ghavamzadeh A (2014). Silibinin induces apoptosis and inhibits proliferation of estrogen receptor (ER)-negative breast carcinoma cells through suppression of nuclear factor kappa B activation. Arch Iran Med.

[346] Yousefi M, Ghaffari SH, Soltani BM, Nafissi S, Momeny M, Zekri A (2012). Therapeutic efficacy of silibinin on human neuroblastoma cells: akt and NF-kappaB expressions may play an important role in silibinin-induced response. Neurochem Res.

[347] Raina K, Agarwal C, Agarwal R (2013). Effect of silibinin in human colorectal cancer cells: targeting the activation of NF-kappaB signaling. Mol Carcinog.

[348] Duan WJ, Li QS, Xia MY, Tashiro S, Onodera S, Ikejima T (2011). Silibinin activated ROS-p38-NF-kappaB positive feedback and induced autophagic death in human fibrosarcoma HT1080 cells. J Asian Nat Prod Res.

[349] Zeng J, Sun Y, Wu K, Li L, Zhang G, Yang Z (2011). Chemopreventive and chemotherapeutic effects of intravesical silibinin against bladder cancer by acting on mitochondria. Mol Cancer Ther.

[350] Tiwari P, Kumar A, Balakrishnan S, Kushwaha HS, Mishra KP (2011). Silibinin-induced apoptosis in MCF7 and T47D human breast carcinoma cells involves caspase-8 activation and mitochondrial pathway. Cancer Invest.

[351] Forghani P, Khorramizadeh MR, Waller EK (2014). Silibinin inhibits accumulation of myeloid-derived suppressor cells and tumor growth of murine breast cancer. Cancer Med.

[352] Ting H, Deep G, Kumar S, Jain AK, Agarwal C, Agarwal R (2016). Beneficial effects of the naturally occurring flavonoid silibinin on the prostate cancer microenvironment: role of monocyte chemotactic protein-1 and immune cell recruitment. Carcinogenesis.

[353] Gohulkumar M, Gurushankar K, Rajendra Prasad N, Krishnakumar N (2014). Enhanced cytotoxicity and apoptosis-induced anticancer effect of silibinin-loaded nanoparticles in oral carcinoma (KB) cells. Mater Sci Eng C Mater Biol Appl..

[354] Yazdi Rouholamini SE, Moghassemi S, Maharat Z, Hakamivala A, Kashanian S, Omidfar K (2018). Effect of silibinin-loaded nano-niosomal coated with trimethyl chitosan on miRNAs expression in 2D and 3D models of T47D breast cancer cell line. Artif Cells Nanomed Biotechnol.

[355] Vue B, Zhang S, Zhang X, Parisis K, Zhang Q, Zheng S (2016). Silibinin derivatives as anti-prostate cancer agents: synthesis and cell-based evaluations. Eur J Med Chem.

[356] Pooja D, Babu Bikkina DJ, Kulhari H, Nikhila N, Chinde S, Raghavendra YM (2014). Fabrication, characterization and bioevaluation of silibinin loaded chitosan nanoparticles. Int J Biol Macromol.

[357] Nasiri M, Zarghami N, Koshki KN, Mollazadeh M, Moghaddam MP, Yamchi MR (2013). Curcumin and silibinin inhibit telomerase expression in T47D human breast cancer cells. Asian Pac J Cancer Prevent.

[358] Chakrabarti M, Ray SK (2015). Synergistic anti-tumor actions of luteolin and silibinin prevented cell migration and invasion and induced apoptosis in glioblastoma SNB19 cells and glioblastoma stem cells. Brain Res.

[359] Nafees S, Mehdi SH, Zafaryab M, Zeya B, Sarwar T, Rizvi MA (2018). Synergistic interaction of rutin and silibinin on human colon cancer cell line. Arch Med Res.

[360] Chen G, Zhang J, Zhang H, Xiao Y, Kao X, Liu Y (2015). Anti-inflammatory effect of emodin on lipopolysaccharide-induced keratitis in Wistar rats. Int J Clin Exp Med..

[361] Li Y, Xiong W, Yang J, Zhong J, Zhang L, Zheng J (2015). Attenuation of inflammation by emodin in lipopolysaccharide-induced acute kidney injury via inhibition of toll-like receptor 2 signal pathway. Iran J Kidney Dis..

[362] Chen GL, Zhang JJ, Kao X, Wei LW, Liu ZY (2015). Emodin ameliorates lipopolysaccharides-induced corneal inflammation in rats. Int J Ophthalmol..

[363] Park SY, Jin ML, Ko MJ, Park G, Choi YW (2016). Anti-neuroinflammatory effect of emodin in LPS-stimulated microglia: involvement of AMPK/Nrf2 activation. Neurochem Res.

[364] Yao WY, Zhou YF, Qian AH, Zhang YP, Qiao MM, Zhai ZK (2015). Emodin has a protective effect in cases of severe acute pancreatitis via inhibition of nuclear factor-kappa B activation resulting in antioxidation. Mol Med Rep..

[365] Alisi A, Pastore A, Ceccarelli S, Panera N, Gnani D, Bruscalupi G (2012). Emodin prevents intrahepatic fat accumulation, inflammation and redox status imbalance during diet-induced hepatosteatosis in rats. Int J Mol Sci.

[366] Sharma R, Tiku AB (2014). Emodin, an anthraquinone derivative, protects against gamma radiation-induced toxicity by inhibiting DNA damage and oxidative stress. Int J Radiat Biol.

[367] Ma L, Yang Y, Yin Z, Liu M, Wang L, Chen L (2017). Emodin suppresses the nasopharyngeal carcinoma cells by targeting the chloride channels. Biomed Pharmacother.

[368] Li XX, Dong Y, Wang W, Wang HL, Chen YY, Shi GY (2013). Emodin as an effective agent in targeting cancer stem-like side population cells of gallbladder carcinoma. Stem Cells Develop..

[369] Haque E, Kamil M, Irfan S, Sheikh S, Hasan A, Nazir A (2018). Blocking mutation independent p53 aggregation by emodin modulates autophagic cell death pathway in lung cancer. Int J Biochem Cell Biol.

[370] Zhang X, Chen Y, Zhang T, Zhang Y (2015). Inhibitory effect of emodin on human hepatoma cell line SMMC-7721 and its mechanism. Afr Health Sci.

[371] Ma YS, Weng SW, Lin MW, Lu CC, Chiang JH, Yang JS (2012). Antitumor effects of emodin on LS1034 human colon cancer cells in vitro and in vivo: roles of apoptotic cell death and LS1034 tumor xenografts model. Food Chem Toxicol.

[372] Manimaran A, Buddhan R, Manoharan S (2017). Emodin downregulates cell proliferation markers during DMBA induced oral carcinogenesis in Golden Syrian Hamsters. Afr J Tradit Complement Altern Med.

[373] Lu J, Xu Y, Zhao Z, Ke X, Wei X, Kang J (2017). Emodin suppresses proliferation, migration and invasion in ovarian cancer cells by down regulating ILK in vitro and in vivo. OncoTargets Ther.

[374] Cha TL, Chuang MJ, Tang SH, Wu ST, Sun KH, Chen TT (2015). Emodin modulates epigenetic modifications and suppresses bladder carcinoma cell growth. Mol Carcinog.

[375] Deng G, Ju X, Meng Q, Yu ZJ, Ma LB (2015). Emodin inhibits the proliferation of PC3 prostate cancer cells in vitro via the Notch signaling pathway. Mol Med Rep..

[376] Sun Y, Wang X, Zhou Q, Lu Y, Zhang H, Chen Q (2015). Inhibitory effect of emodin on migration, invasion and metastasis of human breast cancer MDA-MB-231 cells in vitro and in vivo. Oncol Rep.

[377] Chihara T, Shimpo K, Beppu H, Yamamoto N, Kaneko T, Wakamatsu K (2015). Effects of aloe-emodin and emodin on proliferation of the MKN45 human gastric cancer cell line. Asian Pac J Cancer Prev.

[378] Liu A, Chen H, Wei W, Ye S, Liao W, Gong J (2011). Antiproliferative and antimetastatic effects of emodin on human pancreatic cancer. Oncol Rep.

[379] Qu W, Wang Y, Wu Q, Liu J, Hao D (2015). Emodin inhibits HMGB1-induced tumor angiogenesis in human osteosarcoma by regulating SIRT1. Int J Clin Exp Med..

[380] Wang X, Li L, Guan R, Zhu D, Song N, Shen L (2017). Emodin inhibits ATP-induced proliferation and migration by suppressing P2Y receptors in human lung adenocarcinoma cells. Cell Physiol Biochem.

[381] Dong X, Ni B, Fu J, Yin X, You L, Leng X (2018). Emodin induces apoptosis in human hepatocellular carcinoma HepaRG cells via the mitochondrial caspasedependent pathway. Oncol Rep.

[382] Wang Y, Luo Q, He X, Wei H, Wang T, Shao J (2017). Emodin induces apoptosis of colon cancer cells via induction of autophagy in a ROS-dependent manner. Oncol Res..

[383] Song X, Zhou X, Qin Y, Yang J, Wang Y, Sun Z (2018). Emodin inhibits epithelialmesenchymal transition and metastasis of triple negative breast cancer via antagonism of CCchemokine ligand 5 secreted from adipocytes. Int J Mol Med.

[384] Dai G, Ding K, Cao Q, Xu T, He F, Liu S (2019). Emodin suppresses growth and invasion of colorectal cancer cells by inhibiting VEGFR2. Eur J Pharmacol.

[385] Li N, Wang C, Zhang P, You S (2018). Emodin inhibits pancreatic cancer EMT and invasion by upregulating microRNA1271. Mol Med Rep..

[386] Lin SZ, Xu JB, Ji X, Chen H, Xu HT, Hu P (2015). Emodin inhibits angiogenesis in pancreatic cancer by regulating the transforming growth factor-beta/drosophila mothers against decapentaplegic pathway and angiogenesis-associated microRNAs. Mol Med Rep..

[387] Lin SZ, Wei WT, Chen H, Chen KJ, Tong HF, Wang ZH (2012). Antitumor activity of emodin against pancreatic cancer depends on its dual role: promotion of apoptosis and suppression of angiogenesis. PLoS ONE.

[388] Shi GH, Zhou L (2018). Emodin suppresses angiogenesis and metastasis in anaplastic thyroid cancer by affecting TRAF6mediated pathways in vivo and in vitro. Mol Med Rep..

[389] Sui JQ, Xie KP, Zou W, Xie MJ (2014). Emodin inhibits breast cancer cell proliferation through the ERalpha-MAPK/Akt-cyclin D1/Bcl-2 signaling pathway. Asian Pac J Cancer Prev.

[390] Saunders IT, Mir H, Kapur N, Singh S (2019). Emodin inhibits colon cancer by altering BCL-2 family proteins and cell survival pathways. Cancer Cell Int.

[391] Ying J, Xu H, Wu D, Wu X (2015). Emodin induces apoptosis of human osteosarcoma cells via mitochondria- and endoplasmic reticulum stress-related pathways. Int J Clin Exp Pathol.

[392] Zhang L, He D, Li K, Liu H, Wang B, Zheng L (2017). Emodin targets mitochondrial cyclophilin D to induce apoptosis in HepG2 cells. Biomed Pharmacother.

[393] Su J, Yan Y, Qu J, Xue X, Liu Z, Cai H (2017). Emodin induces apoptosis of lung cancer cells through ER stress and the TRIB3/NF-kappaB pathway. Oncol Rep.

[394] Iwanowycz S, Wang J, Hodge J, Wang Y, Yu F, Fan D (2016). Emodin inhibits breast cancer growth by blocking the tumor-promoting feedforward loop between cancer cells and macrophages. Mol Cancer Ther.

[395] Manu KA, Shanmugam MK, Ong TH, Subramaniam A, Siveen KS, Perumal E (2013). Emodin suppresses migration and invasion through the modulation of CXCR398 expression in an orthotopic model of human hepatocellular carcinoma. PLoS ONE.

[396] Zhang W, Li H, Bu H, Chen H, Tong H, Liu D (2012). Emodin inhibits the differentiation and maturation of dendritic cells and increases the production of regulatory T cells. Int J Mol Med.

[397] Iwanowycz S, Wang J, Altomare D, Hui Y, Fan D (2016). Emodin bidirectionally modulates macrophage polarization and epigenetically regulates macrophage memory. J Biol Chem.

[398] Li Q, Wen J, Yu K, Shu Y, He W, Chu H (2018). Aloe-emodin induces apoptosis in human oral squamous cell carcinoma SCC15 cells. BMC Complement Altern Med.

[399] Gao R, Wu X, Huang Z, Wang B, Li F, Xu H (2019). Anti-tumor effect of aloe-emodin on cervical cancer cells was associated with human papillomavirus E6/E7 and glucose metabolism. OncoTargets Ther..

[400] Yang L, Lin S, Kang Y, Xiang Y, Xu L, Li J (2019). Rhein sensitizes human pancreatic cancer cells to EGFR inhibitors by inhibiting STAT3 pathway. J Exp Clin Cancer Res: CR..

[401] Zhou G, Peng F, Zhong Y, Chen Y, Tang M, Li D (2017). Rhein suppresses matrix metalloproteinase production by regulating the Rac1/ROS/MAPK/AP-1 pathway in human ovarian carcinoma cells. Int J Oncol.

[402] Wang ZH, Chen H, Guo HC, Tong HF, Liu JX, Wei WT (2011). Enhanced antitumor efficacy by the combination of emodin and gemcitabine against human pancreatic cancer cells via downregulation of the expression of XIAP in vitro and in vivo. Int J Oncol.

[403] Liu A, Chen H, Tong H, Ye S, Qiu M, Wang Z (2011). Emodin potentiates the antitumor effects of gemcitabine in pancreatic cancer cells via inhibition of nuclear factor-kappaB. Mol Med Rep..

[404] Chen H, Wei W, Guo Y, Liu A, Tong H, Wang Z (2011). Enhanced effect of gemcitabine by emodin against pancreatic cancer in vivo via cytochrome C-regulated apoptosis. Oncol Rep.

[405] Li X, Wang H, Wang J, Chen Y, Yin X, Shi G (2016). Emodin enhances cisplatin-induced cytotoxicity in human bladder cancer cells through ROS elevation and MRP1 downregulation. BMC Cancer..

[406] Li H, Pan GF, Jiang ZZ, Yang J, Sun LX, Zhang LY (2015). Triptolide inhibits human breast cancer MCF-7 cell growth via downregulation of the ERalpha-mediated signaling pathway. Acta Pharmacol Sin.

[407] Bai S, Hu Z, Yang Y, Yin Y, Li W, Wu L (2016). Anti-inflammatory and neuroprotective effects of triptolide via the NF-kappaB signaling pathway in a Rat MCAO Model. Anatomical Record (Hoboken, NJ: 2007)..

[408] Reno TA, Kim JY, Raz DJ (2015). Triptolide inhibits lung cancer cell migration, invasion, and metastasis. Ann Thor Surg.

[409] Ho JN, Byun SS, Lee S, Oh JJ, Hong SK, Lee SE (2015). Synergistic antitumor effect of triptolide and cisplatin in cisplatin resistant human bladder cancer cells. J Urol.

[410] Wang H, Ma D, Wang C, Zhao S, Liu C (2016). Triptolide inhibits invasion and tumorigenesis of hepatocellular carcinoma MHCC-97H cells through NF-kappaB signaling. Med Sci Monit.

[411] Nakazato T, Sagawa M, Kizaki M (2014). Triptolide induces apoptotic cell death of multiple myeloma cells via transcriptional repression of Mcl-1. Int J Oncol.

[412] Chen J, Qiao Y, Tang B, Chen G, Liu X, Yang B (2017). Modulation of salmonella tumor-colonization and intratumoral anti-angiogenesis by triptolide and its mechanism. Theranostics..

[413] Liu Y, Xiao E, Yuan L, Li G (2014). Triptolide synergistically enhances antitumor activity of oxaliplatin in colon carcinoma in vitro and in vivo. DNA Cell Biol.

[414] Ding X, Zhou X, Jiang B, Zhao Q, Zhou G (2015). Triptolide suppresses proliferation, hypoxia-inducible factor-1alpha and c-Myc expression in pancreatic cancer cells. Mol Med Rep..

[415] Zhao H, Yang Z, Wang X, Zhang X, Wang M, Wang Y (2012). Triptolide inhibits ovarian cancer cell invasion by repression of matrix metalloproteinase 7 and 19 and upregulation of E-cadherin. Exp Mol Med.

[416] Liu S, Zhang J, Zhang X-Z, Zhang H-H, Li X-W, Zhang S-J (2016). Triptolide induces cell apoptosis in human stomach cancer cell via caspase 3-dependent cascade pathway. Trop J Pharm Res..

[417] Yuan S, Wang L, Chen X, Fan B, Yuan Q, Zhang H (2016). Triptolide inhibits the migration and invasion of human prostate cancer cells via Caveolin-1/CD147/MMPs pathway. Biomed Pharmacother.

[418] Chan SF, Chen YY, Lin JJ, Liao CL, Ko YC, Tang NY (2017). Triptolide induced cell death through apoptosis and autophagy in murine leukemia WEHI-3 cells in vitro and promoting immune responses in WEHI-3 generated leukemia mice in vivo. Environ Toxicol.

[419] Yang CY, Lin CK, Lin GJ, Hsieh CC, Huang SH, Ma KH (2017). Triptolide represses oral cancer cell proliferation, invasion, migration, and angiogenesis in co-inoculation with U937 cells. Clin Oral Invest.

[420] Qin G, Li P, Xue Z (2017). Triptolide induces protective autophagy and apoptosis in human cervical cancer cells by downregulating Akt/mTOR activation. Oncol Lett..

[421] Krosch TC, Sangwan V, Banerjee S, Mujumdar N, Dudeja V, Saluja AK (2013). Triptolide-mediated cell death in neuroblastoma occurs by both apoptosis and autophagy pathways and results in inhibition of nuclear factor-kappa B activity. Am J Surg.

[422] Zhao L, Jiang BO, Wang D, Liu W, Zhang H, Liu W (2016). Triptolide reduces the viability of osteosarcoma cells by reducing MKP-1 and Hsp70 expression. Exp Ther Med.

[423] Huang Y, Wu S, Zhang Y, Wang L, Guo Y (2018). Antitumor effect of triptolide in T-cell lymphoblastic lymphoma by inhibiting cell viability, invasion, and epithelial-mesenchymal transition via regulating the PI3K/AKT/mTOR pathway. OncoTargets Ther.

[424] Brincks EL, Kucaba TA, James BR, Murphy KA, Schwertfeger KL, Sangwan V (2015). Triptolide enhances the tumoricidal activity of TRAIL against renal cell carcinoma. FEBS J.

[425] Zhang W, Kang M, Zhang T, Li B, Liao X, Wang R (2016). Triptolide combined with radiotherapy for the treatment of nasopharyngeal carcinoma via NF-kappaB-related mechanism. Int J Mol Sci..

[426] Ni J, Wu Q, Sun ZH, Zhong J, Cai Y, Huang XE (2015). The inhibition effect of triptolide on human endometrial carcinoma cell line HEC-1B: a in vitro and in vivo studies. Asian Pac J Cancer Prevent.

[427] You L, Dong X, Ni B, Fu J, Yang C, Yin X (2018). Triptolide induces apoptosis through fas death and mitochondrial pathways in HepaRG cell line. Front Pharmacol.

[428] Wang Y, Guo SH, Shang XJ, Yu LS, Zhu JW, Zhao A (2018). Triptolide induces Sertoli cell apoptosis in mice via ROS/JNK-dependent activation of the mitochondrial pathway and inhibition of Nrf2-mediated antioxidant response. Acta Pharmacol Sin.

[429] Nardi I, Reno T, Yun X, Sztain T, Wang J, Dai H (2018). Triptolide inhibits Wnt signaling in NSCLC through upregulation of multiple Wnt inhibitory factors via epigenetic modifications to Histone H3. Int J Cancer.

[430] Mao X, Tong J, Wang Y, Zhu Z, Yin Y, Wang Y (2018). Triptolide exhibits antitumor effects by reversing hypermethylation of WIF1 in lung cancer cells. Mol Med Rep..

[431] Li X, Lu Q, Xie W, Wang Y, Wang G (2018). Anti-tumor effects of triptolide on angiogenesis and cell apoptosis in osteosarcoma cells by inducing autophagy via repressing Wnt/beta-Catenin signaling. Biochem Biophys Res Commun.

[432] Kong J, Wang L, Ren L, Yan Y, Cheng Y, Huang Z (2018). Triptolide induces mitochondria-mediated apoptosis of Burkitt’s lymphoma cell via deacetylation of GSK-3beta by increased SIRT3 expression. Toxicol Appl Pharmacol.

[433] Jiang J, Song X, Yang J, Lei K, Ni Y, Zhou F (2018). Triptolide inhibits proliferation and migration of human neuroblastoma SH-SY5Y cells by upregulating MicroRNA-181a. Oncol Res.

[434] Gao H, Zhang Y, Dong L, Qu XY, Tao LN, Zhang YM (2018). Triptolide induces autophagy and apoptosis through ERK activation in human breast cancer MCF-7 cells. Exp Ther Med.

[435] Zhao F, Huang W, Zhang Z, Mao L, Han Y, Yan J (2016). Triptolide induces protective autophagy through activation of the CaMKKbeta-AMPK signaling pathway in prostate cancer cells. Oncotarget..

[436] Liu H, Tang L, Li X, Li H (2018). Triptolide inhibits vascular endothelial growth factor-mediated angiogenesis in human breast cancer cells. Exp Ther Med..

[437] Ma JX, Sun YL, Wang YQ, Wu HY, Jin J, Yu XF (2013). Triptolide induces apoptosis and inhibits the growth and angiogenesis of human pancreatic cancer cells by downregulating COX-2 and VEGF. Oncol Res.

[438] Kumar A, Corey C, Scott I, Shiva S, D’Cunha J (2016). Minnelide/triptolide impairs mitochondrial function by regulating SIRT3 in P53-dependent manner in non-small cell lung cancer. PLoS ONE.

[439] Kwon HY, Kim KS, An HK, Moon HI, Kim HJ, Lee YC (2013). Triptolide induces apoptosis through extrinsic and intrinsic pathways in human osteosarcoma U2OS cells. Indian J Biochem Biophys.

[440] Kwon HY, Kim KS, Baik JS, Moon HI, Lee JW, Kim CH (2013). Triptolide-mediated apoptosis by suppression of focal adhesion kinase through extrinsic and intrinsic pathways in human melanoma cells. Evid Based Complement Altern Med..

[441] Wu PP, Liu KC, Huang WW, Ma CY, Lin H, Yang JS (2011). Triptolide induces apoptosis in human adrenal cancer NCI-H295 cells through a mitochondrial-dependent pathway. Oncol Rep.

[442] Tan BJ, Chiu GN (2013). Role of oxidative stress, endoplasmic reticulum stress and ERK activation in triptolide-induced apoptosis. Int J Oncol.

[443] Yanchun M, Yi W, Lu W, Yu Q, Jian Y, Pengzhou K (2019). Triptolide prevents proliferation and migration of Esophageal Squamous Cell Cancer via MAPK/ERK signaling pathway. Eur J Pharmacol.

[444] Jao HY, Yu FS, Yu CS, Chang SJ, Liu KC, Liao CL (2016). Suppression of the migration and invasion is mediated by triptolide in B16F10 mouse melanoma cells through the NF-kappaB-dependent pathway. Environ Toxicol.

[445] Liu X, Wang K, Duan N, Lan Y, Ma P, Zheng H (2015). Computational prediction and experimental validation of low-affinity target of triptolide and its analogues. RSC Adv..

[446] Hu H, Huang G, Wang H, Li X, Wang X, Feng Y (2018). Inhibition effect of triptolide on human epithelial ovarian cancer via adjusting cellular immunity and angiogenesis. Oncol Rep.

[447] Reno TA, Tong SW, Wu J, Fidler JM, Nelson R, Kim JY (2016). The triptolide derivative MRx102 inhibits Wnt pathway activation and has potent anti-tumor effects in lung cancer. BMC Cancer..

[448] Modi S, Kir D, Giri B, Majumder K, Arora N, Dudeja V (2016). Minnelide overcomes oxaliplatin resistance by downregulating the DNA repair pathway in pancreatic cancer. J Gastrointest Surg..

[449] Yuan ZX, Wu XJ, Mo J, Wang YL, Xu CQ, Lim LY (2015). Renal targeted delivery of triptolide by conjugation to the fragment peptide of human serum albumin. Eur J Pharm Biopharm.

[450] Lin C, Zhang X, Chen H, Bian Z, Zhang G, Riaz MK (2018). Dual-ligand modified liposomes provide effective local targeted delivery of lung-cancer drug by antibody and tumor lineage-homing cell-penetrating peptide. Drug Deliv..

[451] Liu H, Shen M, Zhao X, Ru D, Duan Y, Ding C (2019). The effect of triptolide-loaded exosomes on the proliferation and apoptosis of human ovarian cancer SKOV3 cells. BioMed Res Int..

[452] Aribi A, Gery S, Lee DH, Thoennissen NH, Thoennissen GB, Alvarez R (2013). The triterpenoid cucurbitacin B augments the antiproliferative activity of chemotherapy in human breast cancer. Int J Cancer.

[453] Wang W, Wang Q, Wang L, Li X, Liu D (2018). Enhanced antitumor effect via combination of triptolide with 5-fluorouracil in pancreatic cancer. Translat Cancer Res.

[454] Wang G, Wang X, Xu X (2015). Triptolide potentiates lung cancer cells to cisplatin-induced apoptosis by selectively inhibiting the NER activity. Biomarker Res.

[455] Qi X, Li C, Wu C, Yu C, Liu M, Gao M (2016). Dephosphorylation of Tak1 at Ser412 greatly contributes to the spermatocyte-specific testis toxicity induced by (5R)-5-hydroxytriptolide in C57BL/6 mice. Toxicol Res (Camb)..

[456] Wang L, Xu Y, Fu L, Li Y, Lou L (2012). (5R)-5-hydroxytriptolide (LLDT-8), a novel immunosuppressant in clinical trials, exhibits potent antitumor activity via transcription inhibition. Cancer Lett.

[457] Greeno E, Borazanci E, Gockerman J, Korn R, Saluja A, Von Hoff D (2015). Abstract CT207: phase I dose escalation and pharmokinetic study of 14-O-phosphonooxymethyltriptolide. Cancer Res..

[458] Noel P, Von Hoff DD, Saluja AK, Velagapudi M, Borazanci E, Han H (2019). Triptolide and its derivatives as cancer therapies. Trends Pharmacol Sci.

[459] Wang Y, Zhao G-X, Xu L-H, Liu K-P, Pan H, He J (2014). Cucurbitacin IIb exhibits anti-inflammatory activity through modulating multiple cellular behaviors of mouse lymphocytes. PLoS ONE..

[460] Ma J, Zi Jiang Y, Shi H, Mi C, Li J, Xing Nan J (2014). Cucurbitacin B inhibits the translational expression of hypoxia-inducible factor-1alpha. Eur J Pharmacol.

[461] Piao XM, Gao F, Zhu JX, Wang LJ, Zhao X, Li X (2018). Cucurbitacin B inhibits tumor angiogenesis by triggering the mitochondrial signaling pathway in endothelial cells. Int J Mol Med.

[462] Zhang M, Bian ZG, Zhang Y, Wang JH, Kan L, Wang X (2014). Cucurbitacin B inhibits proliferation and induces apoptosis via STAT3 pathway inhibition in A549 lung cancer cells. Mol Med Rep..

[463] Tseng L-M, Huang P-I, Chen Y-R, Chen Y-C, Chou Y-C, Chen Y-W (2012). Targeting signal transducer and activator of transcription 3 pathway by cucurbitacin I diminishes self-renewing and radiochemoresistant abilities in thyroid cancer-derived CD133 + cells. J Pharmacol Exp Ther.

[464] Huang W-W, Yang J-S, Lin M-W, Chen P-Y, Chiou S-M, Chueh F-S (2012). Cucurbitacin E induces G(2)/M phase arrest through STAT3/p53/p21 signaling and provokes apoptosis via Fas/CD95 and mitochondria-dependent pathways in human bladder cancer T24 cells. Evid Based Complement Alternat Med..

[465] Deng C, Zhang B, Zhang S, Duan C, Cao Y, Kang W (2016). Low nanomolar concentrations of Cucurbitacin-I induces G2/M phase arrest and apoptosis by perturbing redox homeostasis in gastric cancer cells in vitro and in vivo. Cell Death Dis..

[466] Liu J, Liu X, Ma W, Kou W, Li C, Zhao J (2018). Anticancer activity of cucurbitacin-A in ovarian cancer cell line SKOV3 involves cell cycle arrest, apoptosis and inhibition of mTOR/PI3K/Akt signaling pathway. J BUON..

[467] El-Senduny FF, Badria FA, El-Waseef AM, Chauhan SC, Halaweish F (2016). Approach for chemosensitization of cisplatin-resistant ovarian cancer by cucurbitacin B. Tumor Biol..

[468] Lan T, Wang L, Xu Q, Liu W, Jin H, Mao W (2013). Growth inhibitory effect of Cucurbitacin E on breast cancer cells. Int J Clin Exp Pathol..

[469] Lopez-Haber C, Kazanietz MG (2013). Cucurbitacin I inhibits Rac1 activation in breast cancer cells by a reactive oxygen species-mediated mechanism and independently of Janus tyrosine kinase 2 and P-Rex1. Mol Pharmacol.

[470] Hung C-M, Chang C-C, Lin C-W, Ko S-Y, Hsu Y-C (2013). Cucurbitacin E as inducer of cell death and apoptosis in human oral squamous cell carcinoma cell line SAS. Int J Mol Sci..

[471] Wang Y, Xu S, Wu Y, Zhang J (2016). Cucurbitacin E inhibits osteosarcoma cells proliferation and invasion through attenuation of PI3K/AKT/mTOR signalling pathway. Biosci Rep.

[472] Gao Y, Islam MS, Tian J, Lui VW, Xiao D (2014). Inactivation of ATP citrate lyase by Cucurbitacin B: a bioactive compound from cucumber, inhibits prostate cancer growth. Cancer Lett.

[473] Shukla S, Sinha S, Khan S, Kumar S, Singh K, Mitra K (2016). Cucurbitacin B inhibits the stemness and metastatic abilities of NSCLC via downregulation of canonical Wnt/beta-catenin signaling axis. Sci Rep..

[474] Zheng Q, Liu Y, Liu W, Ma F, Zhou Y, Chen M (2014). Cucurbitacin B inhibits growth and induces apoptosis through the JAK2/STAT3 and MAPK pathways in SHSY5Y human neuroblastoma cells. Mol Med Rep..

[475] Zhou X, Yang J, Wang Y, Li W, Li-Ling J, Deng Y (2012). Cucurbitacin B inhibits 12-O-tetradecanoylphorbol 13-acetate-induced invasion and migration of human hepatoma cells through inactivating mitogen-activated protein kinase and PI3K/Akt signal transduction pathways. Hepatol Res.

[476] Zha Q-B, Zhang X-Y, Lin Q-R, Xu L-H, Zhao G-X, Pan H (2015). Cucurbitacin E induces autophagy via downregulating mTORC1 signaling and upregulating AMPK activity. PLoS ONE..

[477] Mao D, Liu AH, Wang ZP, Zhang XW, Lu H (2019). Cucurbitacin B inhibits cell proliferation and induces cell apoptosis in colorectal cancer by modulating methylation status of BTG3. Neoplasma..

[478] Xie YL, Tao WH, Yang TX, Qiao JG (2016). Anticancer effect of cucurbitacin B on MKN-45 cells via inhibition of the JAK2/STAT3 signaling pathway. Exp Ther Med.

[479] Zhang YZ, Wang CF, Zhang LF (2018). Cucurbitacin D impedes gastric cancer cell survival via activation of the iNOS/NO and inhibition of the Akt signalling pathway. Oncol Rep.

[480] Kong Y, Chen J, Zhou Z, Xia H, Qiu MH, Chen C (2014). Cucurbitacin E induces cell cycle G2/M phase arrest and apoptosis in triple negative breast cancer. PLoS ONE.

[481] Zhang T, Li Y, Park KA, Byun HS, Won M, Jeon J (2012). Cucurbitacin induces autophagy through mitochondrial ROS production which counteracts to limit caspase-dependent apoptosis. Autophagy..

[482] Ma G, Luo W, Lu J, Ma DL, Leung CH, Wang Y (2016). Cucurbitacin E induces caspase-dependent apoptosis and protective autophagy mediated by ROS in lung cancer cells. Chem Biol Interact.

[483] Yuan G, Yan SF, Xue H, Zhang P, Sun JT, Li G (2014). Cucurbitacin I induces protective autophagy in glioblastoma in vitro and in vivo. J Biol Chem.

[484] Liang J, Zhang XL, Yuan JW, Zhang HR, Liu D, Hao J (2019). Cucurbitacin B inhibits the migration and invasion of breast cancer cells by altering the biomechanical properties of cells. Phytother Res.

[485] Sinha S, Khan S, Shukla S, Lakra AD, Kumar S, Das G (2016). Cucurbitacin B inhibits breast cancer metastasis and angiogenesis through VEGF-mediated suppression of FAK/MMP-9 signaling axis. Int J Biochem Cell Biol.

[486] Hsu PC, Tian B, Yang YL, Wang YC, Liu S, Urisman A (2019). Cucurbitacin E inhibits the Yesassociated protein signaling pathway and suppresses brain metastasis of human nonsmall cell lung cancer in a murine model. Oncol Rep.

[487] Song J, Liu H, Li Z, Yang C, Wang C (2015). Cucurbitacin I inhibits cell migration and invasion and enhances chemosensitivity in colon cancer. Oncol Rep.

[488] Kim HJ, Kim J-K (2015). Antiangiogenic effects of cucurbitacin-I. Arch Pharmacal Res.

[489] Ku JM, Kim SR, Hong SH, Choi H-S, Seo HS, Shin YC (2015). Cucurbitacin D induces cell cycle arrest and apoptosis by inhibiting STAT3 and NF-κB signaling in doxorubicin-resistant human breast carcinoma (MCF7/ADR) cells. Mol Cell Biochem.

[490] Hsu YC, Huang TY, Chen MJ (2014). Therapeutic ROS targeting of GADD45gamma in the induction of G2/M arrest in primary human colorectal cancer cell lines by cucurbitacin E. Cell Death Dis.

[491] Spear SA, Burns SS, Oblinger JL, Ren Y, Pan L, Kinghorn AD (2013). Natural compounds as potential treatments of NF2-deficient schwannoma and meningioma: cucurbitacin D and goyazensolide. Otol Neurotol..

[492] Kausar H, Munagala R, Bansal SS, Aqil F, Vadhanam MV, Gupta RC (2013). Cucurbitacin B potently suppresses non-small-cell lung cancer growth: identification of intracellular thiols as critical targets. Cancer Lett.

[493] Li H, Chen H, Li R, Xin J, Wu S, Lan J (2018). Cucurbitacin I induces cancer cell death through the endoplasmic reticulum stress pathway. J Cell Biochem.

[494] Lu P, Yu B, Xu J (2012). Cucurbitacin B regulates immature myeloid cell differentiation and enhances antitumor immunity in patients with lung cancer. Cancer Biother Radiopharm.

[495] Hira SK, Mondal I, Manna PP (2015). Combined immunotherapy with whole tumor lysate-pulsed interleukin-15-activated dendritic cells and cucurbitacin I promotes strong CD8(+) T-cell responses and cures highly aggressive lymphoma. Cytotherapy.

[496] Ge W, Chen X, Han F, Liu Z, Wang T, Wang M (2018). Synthesis of Cucurbitacin B derivatives as potential anti-hepatocellular carcinoma agents. Molecules (Basel, Switzerland)..

[497] Tang L, Fu L, Zhu Z, Yang Y, Sun B, Shan W (2018). Modified mixed nanomicelles with collagen peptides enhanced oral absorption of Cucurbitacin B: preparation and evaluation. Drug Delivery.

[498] Lee DH, Thoennissen NH, Goff C, Iwanski GB, Forscher C, Doan NB (2011). Synergistic effect of low-dose cucurbitacin B and low-dose methotrexate for treatment of human osteosarcoma. Cancer Lett (N Y, NY, U S)..

[499] Sun Y, Zhang J, Zhou J, Huang Z, Hu H, Qiao M (2015). Synergistic effect of cucurbitacin B in combination with curcumin via enhancing apoptosis induction and reversing multidrug resistance in human hepatoma cells. Eur J Pharmacol.

[500] Garg S, Huifu H, Kaul SC, Wadhwa R, Garg S, Wadhwa R (2019). Induction of senescence in cancer cells by a novel combination of Cucurbitacin B and withanone: molecular mechanism and therapeutic potential. J Gerontol A Biol Sci Med Sci..

[501] Eyol E, Tanriverdi Z, Karakus F, Yilmaz K, Unuvar S (2016). Synergistic anti-proliferative effects of cucurbitacin i and irinotecan onhuman colorectal cancer cell lines. Clin Exp Pharmacol..

[502] Lee J, Sohn EJ, Yoon S, Won G, Kim CG, Jung JH (2017). Activation of JNK and IRE1 is critically involved in tanshinone I-induced p62 dependent autophagy in malignant pleural mesothelioma cells: implication of p62 UBA domain. Oncotarget..

[503] Xu SW, Little PJ, Lan T, Huang Y, Le K, Wu XQ (2011). Tanshinone II-A attenuates and stabilizes atherosclerotic plaques in Apolipoprotein-E knockout mice fed a high cholesterol diet. Arch Biochem Biophys.

[504] Gong Y, Li YL, Lu Y, Li LL, Abdolmaleky H, Blackburn GL (2011). Bioactive tanshinones in Salvia Miltiorrhiza inhibit the growth of prostate cancer cells in vitro and in mice. Int J Cancer.

[505] Yin X, Yin Y, Cao FL, Chen YF, Peng Y, Hou WG (2012). Tanshinone IIA attenuates the inflammatory response and apoptosis after traumatic injury of the spinal cord in adult rats. Plos ONE..

[506] Li ZM, Xu SW, Liu PQ (2018). Salvia miltiorrhizaBurge (Danshen): a golden herbal medicine in cardiovascular therapeutics. Acta Pharmacologica Sinica..

[507] Zhang RW, Liu ZG, Xie Y, Wang LX, Li MC, Sun X (2016). In vitro inhibition of invasion and metastasis in colon cancer cells by TanIIA. Genetics Mol Res Gmr..

[508] Nicolin V, Fancellu G, Valentini R (2014). Effect of tanshinone II on cell growth of breast cancer cell line type MCF-7 and MD-MB-231. Italian J Anat Embryol.

[509] Shin EA, Sohn EJ, Won G, Choi JU, Jeong M, Kim B (2014). Upregulation of microRNA135a-3p and death receptor 5 plays a critical role in Tanshinone I sensitized prostate cancer cells to TRAIL induced apoptosis. Oncotarget..

[510] Jing X, Xu Y, Cheng W, Guo S, Zou Y, He L (2016). Tanshinone I induces apoptosis and pro-survival autophagy in gastric cancers. Cancer Chemother Pharmacol.

[511] Yun SM, Jung JH, Jeong SJ, Sohn EJ, Kim B, Kim SH (2014). Tanshinone IIA induces autophagic cell death via activation of AMPK and ERK and inhibition of mTOR and p70 S6K in KBM-5 leukemia cells. Phytother Res.

[512] Zhang K, Li J, Meng W, Xing H, Yang Y (2016). Tanshinone IIA inhibits acute promyelocytic leukemia cell proliferation and induces their apoptosis in vivo. Blood Cells Mol Dis.

[513] Wang L, Wu J, Lu J, Ma R, Sun D, Tang J (2015). Regulation of the cell cycle and PI3K/Akt/mTOR signaling pathway by tanshinone I in human breast cancer cell lines. Mol Med Rep..

[514] Jiao Y, Wang X, Li Y, Tang B (2017). Tanshinone IIA suppresses gastric cancer cell proliferation and migration by downregulation of FOXM1. Oncol Rep.

[515] Kim EO, Kang SE, Im CR, Lee JH, Ahn KS, Yang WM (2016). Tanshinone IIA induces TRAIL sensitization of human lung cancer cells through selective ER stress induction. Int J Oncol.

[516] Guerram M, Jiang ZZ, Yousef BA, Hamdi AM, Hassan HM, Yuan ZQ (2015). The potential utility of acetyltanshinone IIA in the treatment of HER2-overexpressed breast cancer: induction of cancer cell death by targeting apoptotic and metabolic signaling pathways. Oncotarget..

[517] Yan MY, Chien SY, Kuo SJ, Chen DR, Su CC (2012). Tanshinone IIA inhibits BT-20 human breast cancer cell proliferation through increasing caspase 12, GADD153 and phospho-p38 protein expression. Int J Mol Med.

[518] Jian C, Dong-Yun S, Shan-Lin L, Liang Z (2012). Tanshinone IIA induces growth inhibition and apoptosis in gastric cancer in vitro and in vivo. Oncol Rep.

[519] Qin J, Shi H, Xu Y, Zhao F, Wang Q (2018). Tanshinone IIA inhibits cervix carcinoma stem cells migration and invasion via inhibiting YAP transcriptional activity. Biomed Pharmacother.

[520] Chen L, Zheng SZ, Sun ZG, Wang AY, Huang CH, Punchard NA (2011). Cryptotanshinone has diverse effects on cell cycle events in melanoma cell lines with different metastatic capacity. Cancer Chemother Pharmacol.

[521] Wang Y, Li JX, Wang YQ, Miao ZH (2015). Tanshinone I inhibits tumor angiogenesis by reducing Stat3 phosphorylation at Tyr705 and hypoxia-induced HIF-1alpha accumulation in both endothelial and tumor cells. Oncotarget..

[522] Lee HP, Liu YC, Chen PC, Tai HC, Li TM, Fong YC (2017). Tanshinone IIA inhibits angiogenesis in human endothelial progenitor cells in vitro and in vivo. Oncotarget..

[523] Sui H, Zhao J, Zhou L, Wen H, Deng W, Li C (2017). Tanshinone IIA inhibits beta-catenin/VEGF-mediated angiogenesis by targeting TGF-beta1 in normoxic and HIF-1alpha in hypoxic microenvironments in human colorectal cancer. Cancer Lett.

[524] Zhang L, Chen C, Duanmu J, Wu Y, Tao J, Yang A (2018). Cryptotanshinone inhibits the growth and invasion of colon cancer by suppressing inflammation and tumor angiogenesis through modulating MMP/TIMP system, PI3K/Akt/mTOR signaling and HIF-1alpha nuclear translocation. Int Immunopharmacol.

[525] Xu X, Wu L, Zhou X, Zhou N, Zhuang Q, Yang J (2017). Cryptotanshinone inhibits VEGF-induced angiogenesis by targeting the VEGFR2 signaling pathway. Microvasc Res.

[526] Yen JH, Huang ST, Huang HS, Fong YC, Wu YY, Chiang JH (2018). HGK-sestrin 2 signaling-mediated autophagy contributes to antitumor efficacy of Tanshinone IIA in human osteosarcoma cells. Cell Death Dis.

[527] Qiu Y, Li C, Wang Q, Zeng X, Ji P (2018). Tanshinone IIA induces cell death via Beclin-1-dependent autophagy in oral squamous cell carcinoma SCC-9 cell line. Cancer Med.

[528] Li X, Li Z, Li X, Liu B, Liu Z (2017). Mechanisms of Tanshinone II a inhibits malignant melanoma development through blocking autophagy signal transduction in A375 cell. BMC Cancer..

[529] Ding L, Wang S, Wang W, Lv P, Zhao D, Chen F (2017). Tanshinone IIA affects autophagy and apoptosis of glioma cells by inhibiting phosphatidylinositol 3-Kinase/Akt/mammalian target of rapamycin signaling pathway. Pharmacology.

[530] Li C, Han X, Hong Z, Wu J, Bao L (2016). The interplay between autophagy and apoptosis induced by tanshinone IIA in prostate cancer cells. Tumor Biol.

[531] Huang SY, Chang SF, Liao KF, Chiu SC (2017). Tanshinone IIA inhibits epithelial-mesenchymal transition in bladder cancer cells via modulation of STAT3-CCL2 signaling. Int J Mol Sci..

[532] Kim SA, Kang OH, Kwon DY (2018). Cryptotanshinone induces cell cycle arrest and apoptosis of NSCLC cells through the PI3K/Akt/GSK-3beta pathway. Int J Mol Sci..

[533] Park IJ, Kim MJ, Park OJ, Choe W, Kang I, Kim SS (2012). Cryptotanshinone induces ER stress-mediated apoptosis in HepG2 and MCF7 cells. Apoptosis.

[534] Li Q, He K, Tang S, Xu LF, Luo YC (2016). Anti-tumor activity of tanshinone IIA in combined with cyclophosphamide against Lewis mice with lung cancer. Asian Pac J Trop Med.

[535] Liu S, Han Z, Trivett AL, Lin H, Hannifin S, Yang D (2019). Cryptotanshinone has curative dual anti-proliferative and immunotherapeutic effects on mouse Lewis lung carcinoma. Cancer Immunol Immunother.

[536] Zhang J, Li Y, Fang X, Zhou D, Wang Y, Chen M (2014). TPGS-g-PLGA/Pluronic F68 mixed micelles for tanshinone IIA delivery in cancer therapy. Int J Pharm.

[537] Li Z, Zhang Y, Zhang K, Wu Z, Feng N (2018). Biotinylated-lipid bilayer coated mesoporous silica nanoparticles for improving the bioavailability and anti-leukaemia activity of Tanshinone IIA. Artif Cells Nanomed Biotechnol.

[538] Qiu S, Granet R, Mbakidi JP, Bregier F, Pouget C, Micallef L (2016). Delivery of tanshinone IIA and alpha-mangostin from gold/PEI/cyclodextrin nanoparticle platform designed for prostate cancer chemotherapy. Bioorg Med Chem Lett.

[539] Bai Y, Zhang L, Fang X, Yang Y (2016). Tanshinone IIA enhances chemosensitivity of colon cancer cells by suppressing nuclear factor-kappaB. Exp Ther Med.

[540] Li K, Liu W, Zhao Q, Wu C, Fan C, Lai H (2019). Combination of tanshinone IIA and doxorubicin possesses synergism and attenuation effects on doxorubicin in the treatment of breast cancer. Phytother Res.

[541] Jung JH, Kwon TR, Jeong SJ, Kim EO, Sohn EJ, Yun M (2013). Apoptosis induced by tanshinone IIA and cryptotanshinone is mediated by distinct JAK/STAT3/5 and SHP1/2 signaling in chronic myeloid leukemia K562 cells. Evid Based Complement Altern Med..

[542] Xu P, Ji L, Tian S, Li F (2018). Clinical effects of tanshinone IIA sodium sulfonate combined with trimetazidine and levocarnitine in the treatment of AVMC and its effects on serum TNF-alpha, IL-18 and IL-35. Exp Ther Med.

[543] Wang YY, Lv YF, Lu L, Cai L (2014). Oridonin inhibits mTOR signaling and the growth of lung cancer tumors. Anticancer Drugs.

[544] Zhang Y, Wang L, Zi Y, Zhang L, Guo Y, Huang Y (2017). Oridonin effectively reverses the drug resistance of cisplatin involving induction of cell apoptosis and inhibition of MMP expression in human acute myeloid leukemia cells. Saudi J Biol Sci..

[545] Huang HL, Weng HY, Wang LQ, Yu CH, Huang QJ, Zhao PP (2012). Triggering Fbw7-mediated proteasomal degradation of c-Myc by oridonin induces cell growth inhibition and apoptosis. Mol Cancer Ther.

[546] Wang H, Ye Y, Chu JH, Zhu GY, Fong WF, Yu ZL (2011). Proteomic and functional analyses reveal the potential involvement of endoplasmic reticulum stress and alpha-CP1 in the anticancer activities of oridonin in HepG2 cells. Integr Cancer Ther.

[547] Lu Y, Sun Y, Zhu J, Yu L, Jiang X, Zhang J (2018). Oridonin exerts anticancer effect on osteosarcoma by activating PPAR-gamma and inhibiting Nrf2 pathway. Cell Death Dis..

[548] Zhao J, Zhang M, He P, Zhao J, Chen Y, Qi J (2017). Proteomic analysis of oridonin-induced apoptosis in multiple myeloma cells. Mol Med Rep..

[549] Gu Z, Wang X, Qi R, Wei L, Huo Y, Ma Y (2015). Oridonin induces apoptosis in uveal melanoma cells by upregulation of Bim and downregulation of Fatty Acid Synthase. Biochem Biophys Res Commun.

[550] Kang N, Cao SJ, Zhou Y, He H, Tashiro S, Onodera S (2015). Inhibition of caspase-9 by oridonin, a diterpenoid isolated from Rabdosia rubescens, augments apoptosis in human laryngeal cancer cells. Int J Oncol.

[551] Jiang JH, Pi J, Jin H, Cai JY (2019). Oridonin-induced mitochondria-dependent apoptosis in esophageal cancer cells by inhibiting PI3K/AKT/mTOR and Ras/Raf pathways. J Cell Biochem.

[552] Yang J, Ren X, Zhang L, Li Y, Cheng B, Xia J (2018). Oridonin inhibits oral cancer growth and PI3K/Akt signaling pathway. Biomed Pharmacother.

[553] Ren CM, Li Y, Chen QZ, Zeng YH, Shao Y, Wu QX (2016). Oridonin inhibits the proliferation of human colon cancer cells by upregulating BMP7 to activate p38 MAPK. Oncol Rep.

[554] Xia S, Zhang X, Li C, Guan H (2017). Oridonin inhibits breast cancer growth and metastasis through blocking the Notch signaling. Saudi Pharm J..

[555] Gao SY, Li J, Qu XY, Zhu N, Ji YB (2014). Downregulation of Cdk1 and cyclinB1 expression contributes to oridonin-induced cell cycle arrest at G2/M phase and growth inhibition in SGC-7901 gastric cancer cells. Asian Pac J Cancer Prevent.

[556] Xu B, Shen W, Liu X, Zhang T, Ren J, Fan Y (2015). Oridonin inhibits BxPC-3 cell growth through cell apoptosis. Acta Biochim Biophys Sin (Shanghai).

[557] Li J, Yang L, Wu H (2011). Oridonin induced the apoptosis of PC-3 cells and its mechanism. J Central South Univ Med Sci..

[558] Zhang LD, Liu Z, Liu H, Ran DM, Guo JH, Jiang B (2016). Oridonin enhances the anticancer activity of NVP-BEZ235 against neuroblastoma cells in vitro and in vivo through autophagy. Int J Oncol.

[559] Cai DT, Jin H, Xiong QX, Liu WG, Gao ZG, Gu GX (2013). ER stress and ASK1-JNK activation contribute to oridonin-induced apoptosis and growth inhibition in cultured human hepatoblastoma HuH-6 cells. Mol Cell Biochem.

[560] Yao Z, Xie F, Li M, Liang Z, Xu W, Yang J (2017). Oridonin induces autophagy via inhibition of glucose metabolism in p53-mutated colorectal cancer cells. Cell Death Dis.

[561] Wang H, Zhu L, Feng X, Zhang H, Luo Q, Chen F (2017). Oridonin induces G2/M cell cycle arrest and apoptosis in human oral squamous cell carcinoma. Eur J Pharmacol.

[562] Tian L, Xie K, Sheng D, Wan X, Zhu G (2017). Antiangiogenic effects of oridonin. BMC Complement Altern Med.

[563] Li CY, Wang Q, Shen S, Wei XL, Li GX (2018). Oridonin inhibits migration, invasion, adhesion and TGF-beta1-induced epithelial-mesenchymal transition of melanoma cells by inhibiting the activity of PI3K/Akt/GSK-3beta signaling pathway. Oncol Lett.

[564] Li C, Wang Q, Shen S, Wei X, Li G (2018). Oridonin inhibits VEGF-A-associated angiogenesis and epithelial-mesenchymal transition of breast cancer in vitro and in vivo. Oncol Lett.

[565] Liu X, Kang J, Wang H, Huang T (2018). Mitochondrial ROS contribute to oridonin-induced HepG2 apoptosis through PARP activation. Oncol Lett..

[566] Lu J, Chen X, Qu S, Yao B, Xu Y, Wu J (2017). Oridonin induces G2/M cell cycle arrest and apoptosis via the PI3K/Akt signaling pathway in hormone-independent prostate cancer cells. Oncol Lett.

[567] Zhang T, Tan Y, Zhao R, Liu Z (2013). DNA damage induced by oridonin involves cell cycle arrest at G2/M phase in human MCF-7 cells. Contemporary Oncol (Poznan, Poland)..

[568] Liu Y, Liu JH, Chai K, Tashiro S, Onodera S, Ikejima T (2013). Inhibition of c-Met promoted apoptosis, autophagy and loss of the mitochondrial transmembrane potential in oridonin-induced A549 lung cancer cells. J Pharm Pharmacol.

[569] Zhang YH, Wu YL, Tashiro S, Onodera S, Ikejima T (2011). Reactive oxygen species contribute to oridonin-induced apoptosis and autophagy in human cervical carcinoma HeLa cells. Acta Pharmacol Sin.

[570] Zeng R, Chen Y, Zhao S, Cui GH (2012). Autophagy counteracts apoptosis in human multiple myeloma cells exposed to oridonin in vitro via regulating intracellular ROS and SIRT1. Acta Pharmacol Sin.

[571] Yu Y, Fan SM, Song JK, Tashiro S, Onodera S, Ikejima T (2012). Hydroxyl radical (·OH) played a pivotal role in oridonin-induced apoptosis and autophagy in human epidermoid carcinoma A431 cells. Biol Pharm Bull.

[572] Cao S, Huang Y, Zhang Q, Lu F, Donkor PO, Zhu Y (2019). Molecular mechanisms of apoptosis and autophagy elicited by combined treatment with oridonin and cetuximab in laryngeal squamous cell carcinoma. Apoptosis.

[573] Sun Y, Jiang X, Lu Y, Zhu J, Yu L, Ma B (2018). Oridonin prevents epithelial–mesenchymal transition and TGF-beta1-induced epithelial–mesenchymal transition by inhibiting TGF-beta1/Smad2/3 in osteosarcoma. Chem Biol Interact.

[574] Zhu MIN, Hong DUN, Bao Y, Wang C, Pan W (2013). Oridonin induces the apoptosis of metastatic hepatocellular carcinoma cells via a mitochondrial pathway. Oncol Lett.

[575] Gao S, Tan H, Zhu N, Gao H, Lv C, Gang J (2016). Oridonin induces apoptosis through the mitochondrial pathway in human gastric cancer SGC-7901 cells. Int J Oncol.

[576] Wu QX, Yuan SX, Ren CM, Yu Y, Sun WJ, He BC (2016). Oridonin upregulates PTEN through activating p38 MAPK and inhibits proliferation in human colon cancer cells. Oncol Rep.

[577] Liu RX, Ma Y, Hu XL, Ren WY, Liao YP, Wang H (2018). Anticancer effects of oridonin on colon cancer are mediated via BMP7/p38 MAPK/p53 signaling. Int J Oncol.

[578] Gao F-H, Liu F, Wei WEI, Liu L-B, Xu M-H, Guo Z-Y (2012). Oridonin induces apoptosis and senescence by increasing hydrogen peroxide and glutathione depletion in colorectal cancer cells. Int J Mol Med.

[579] Jin H, Tan X, Liu X, Ding Y (2011). Downregulation of AP-1 gene expression is an initial event in the oridonin-mediated inhibition of colorectal cancer: studies in vitro and in vivo. J Gastroenterol Hepatol.

[580] Wu QJ, Zheng XC, Wang T, Zhang TY (2018). Effects of oridonin on immune cells, Th1/Th2 balance and the expression of BLys in the spleens of broiler chickens challenged with Salmonella pullorum. Res Vet Sci.

[581] Chen RY, Xu B, Chen SF, Chen SS, Zhang T, Ren J (2014). Effect of oridonin-mediated hallmark changes on inflammatory pathways in human pancreatic cancer (BxPC-3) cells. World J Gastroenterol.

[582] Wang SQ, Wang C, Wang JW, Yang DX, Wang R, Wang CJ (2017). Geridonin, a novel derivative of oridonin, inhibits proliferation of MGC 803 cells both in vitro and in vivo through elevating the intracellular ROS. J Pharm Pharmacol..

[583] Li Y, Wang Y, Wang S, Gao Y, Zhang X, Lu C (2015). Oridonin phosphate-induced autophagy effectively enhances cell apoptosis of human breast cancer cells. Med Oncol.

[584] Chen W, Zhou J, Wu K, Huang J, Ding Y, Yun EJ (2016). Targeting XBP1-mediated beta-catenin expression associated with bladder cancer with newly synthetic Oridonin analogues. Oncotarget..

[585] Zhou J, Yun EJ, Chen W, Ding Y, Wu K, Wang B (2017). Targeting 3-phosphoinositide-dependent protein kinase 1 associated with drug-resistant renal cell carcinoma using new oridonin analogs. Cell Death Dis.

[586] Zhu L, Li M, Liu X, Du L, Jin Y (2017). Inhalable oridonin-loaded poly(lactic-co-glycolic)acid large porous microparticles for in situ treatment of primary non-small cell lung cancer. Acta Pharm Sinica B..

[587] Wang Y, Liu X, Liu G, Guo H, Li C, Zhang Y (2016). Novel galactosylated biodegradable nanoparticles for hepatocyte-delivery of oridonin. Int J Pharm.

[588] Yu Y, Fan SM, Ye YC, Tashiro S, Onodera S, Ikejima T (2012). The tyrphostin AG1478 augments oridonin-induced A431 cell apoptosis by blockage of JNK MAPK and enhancement of oxidative stress. Free Radic Res..

[589] Tiwari RV, Parajuli P, Sylvester PW (2015). Synergistic anticancer effects of combined gamma-tocotrienol and oridonin treatment is associated with the induction of autophagy. Mol Cell Biochem.

[590] Ding Y, Ding C, Ye N, Liu Z, Wold EA, Chen H (2016). Discovery and development of natural product oridonin-inspired anticancer agents. Eur J Med Chem.

[591] Chen CH, Lin ML, Ong PL, Yang JT (2012). Novel multiple apoptotic mechanism of shikonin in human glioma cells. Ann Surg Oncol.

[592] Lu L, Qin A, Huang H, Zhou P, Zhang C, Liu N (2011). Shikonin extracted from medicinal Chinese herbs exerts anti-inflammatory effect via proteasome inhibition. Eur J Pharmacol.

[593] Kim HJ, Hwang KE, Park DS, Oh SH, Jun HY, Yoon KH (2017). Shikonin-induced necroptosis is enhanced by the inhibition of autophagy in non-small cell lung cancer cells. J Transl Med.

[594] Zuo AR, Dong HH, Yu YY, Shu QL, Zheng LX, Yu XY (2018). The antityrosinase and antioxidant activities of flavonoids dominated by the number and location of phenolic hydroxyl groups. Chin Med.

[595] Jang SY, Lee JK, Jang EH, Jeong SY, Kim JH (2014). Shikonin blocks migration and invasion of human breast cancer cells through inhibition of matrix metalloproteinase-9 activation. Oncol Rep.

[596] Chen CM, Shanmugasundaram K, Rigby AC, Kung AL (2013). Shikonin, a natural product from the root of Lithospermum erythrorhizon, is a cytotoxic DNA-binding agent. Eur J Pharm Sci.

[597] Chen Y, Zheng L, Liu J, Zhou Z, Cao X, Lv X (2014). Shikonin inhibits prostate cancer cells metastasis by reducing matrix metalloproteinase-2/-9 expression via AKT/mTOR and ROS/ERK1/2 pathways. Int Immunopharmacol.

[598] Hao ZF, Qian J, Yang JS (2015). Shikonin induces apoptosis and inhibits migration of ovarian carcinoma cells by inhibiting the phosphorylation of Src and FAK. Oncol Lett.

[599] Ni F, Huang X, Chen Z, Qian W, Tong X (2018). Shikonin exerts antitumor activity in Burkitt’s lymphoma by inhibiting C-MYC and PI3K/AKT/mTOR pathway and acts synergistically with doxorubicin. Sci Rep..

[600] Yang Q, Ji M, Guan H, Shi B, Hou P (2013). Shikonin inhibits thyroid cancer cell growth and invasiveness through targeting major signaling pathways. J Clin Endocrinol Metab.

[601] Lan WJ, Wan SB, Gu WQ, Wang HY, Zhou SW (2014). Mechanisms behind the inhibition of lung adenocarcinoma cell by shikonin. Cell Biochem Biophys.

[602] Hsieh YS, Liao CH, Chen WS, Pai JT, Weng MS (2017). Shikonin inhibited migration and invasion of human lung cancer cells via suppression of c-Met-mediated epithelial-to-mesenchymal transition. J Cell Biochem.

[603] Zhai T, Hei Z, Ma Q, Liang H, Xu Y, Zhang Y (2017). Shikonin induces apoptosis and G0/G1 phase arrest of gallbladder cancer cells via the JNK signaling pathway. Oncol Rep.

[604] Tang JC, Zhao J, Long F, Chen JY, Mu B, Jiang Z (2018). Efficacy of Shikonin against esophageal cancer cells and its possible mechanisms in vitro and in vivo. J Cancer..

[605] Tian R, Li Y, Gao M (2015). Shikonin causes cell-cycle arrest and induces apoptosis by regulating the EGFR–NF-κB signalling pathway in human epidermoid carcinoma A431 cells. Biosci Rep.

[606] Liu T, Sun X, Cao Z (2019). Shikonin-induced necroptosis in nasopharyngeal carcinoma cells via ROS overproduction and upregulation of RIPK1/RIPK3/MLKL expression. OncoTargets Ther..

[607] Lu B, Gong X, Wang ZQ, Ding Y, Wang C, Luo TF (2017). Shikonin induces glioma cell necroptosis in vitro by ROS overproduction and promoting RIP1/RIP3 necrosome formation. Acta Pharmacol Sin.

[608] Liu Y, Kang X, Niu G, He S, Zhang T, Bai Y (2019). Shikonin induces apoptosis and prosurvival autophagy in human melanoma A375 cells via ROS-mediated ER stress and p38 pathways. Artif Cells Nanomed Biotechnol.

[609] Shi S, Cao H (2014). Shikonin promotes autophagy in BXPC-3 human pancreatic cancer cells through the PI3K/Akt signaling pathway. Oncol Lett.

[610] Gong K, Zhang Z, Chen Y, Shu HB, Li W (2014). Extracellular signal-regulated kinase, receptor interacting protein, and reactive oxygen species regulate shikonin-induced autophagy in human hepatocellular carcinoma. Eur J Pharmacol.

[611] Wang H, Wu C, Wan S, Zhang H, Zhou S, Liu G (2013). Shikonin attenuates lung cancer cell adhesion to extracellular matrix and metastasis by inhibiting integrin beta1 expression and the ERK1/2 signaling pathway. Toxicology.

[612] Huang CJ, Luo YA, Zhao JW, Yang FW, Zhao HW, Fan WH (2013). Shikonin kills glioma cells through necroptosis mediated by RIP-1. PloS ONE..

[613] Yang JT, Li ZL, Wu JY, Lu FJ, Chen CH (2014). An oxidative stress mechanism of shikonin in human glioma cells. PloS ONE..

[614] Liu J, Wang P, Xue Y-X, Li Z, Qu C-B, Liu Y-H (2015). Enhanced antitumor effect of shikonin by inhibiting endoplasmic reticulum stress via JNK/c-Jun pathway in human glioblastoma stem cells. Biochem Biophys Res Commun..

[615] Wang H, Liu Z, Li X, Zhao R, Pu Y, Wu H (2018). Shikonin causes apoptosis by disrupting intracellular calcium homeostasis and mitochondrial function in human hepatoma cells. Exp Ther Med.

[616] Liang W, Cui J, Zhang K, Xi H, Cai A, Li J (2017). Shikonin induces ROS-based mitochondria-mediated apoptosis in colon cancer. Oncotarget..

[617] Qu D, Chen YU, Xu XM, Zhang M, Zhang YI, Li SQ (2015). Cbl-b-regulated extracellular signal-regulated kinase signaling is involved in the shikonin-induced apoptosis of lung cancer cells in vitro. Exp Ther Med.

[618] Zhao QL, Assimopoulou AN, Klauck SM, Damianakos H, Chinou I, Kretschmer N (2015). Inhibition of c-MYC with involvement of ERK/JNK/MAPK and AKT pathways as a novel mechanism for shikonin and its derivatives in killing leukemia cells. Oncotarget..

[619] Chen Y, Wang TT, Du J, Li YC, Wang X, Zhou Y (2018). The critical role of PTEN/PI3K/AKT signaling pathway in shikonin-induced apoptosis and proliferation inhibition of chronic myeloid leukemia. Cell Physiol Biochem.

[620] Shan ZL, Zhong L, Xiao CL, Gan LG, Xu T, Song H (2017). Shikonin suppresses proliferation and induces apoptosis in human leukemia NB4 cells through modulation of MAPKs and cMyc. Mol Med Rep..

[621] Zhou ZJ, Lu B, Wang C, Wang ZQ, Luo TF, Piao MH (2017). RIP1 and RIP3 contribute to shikonin-induced DNA double-strand breaks in glioma cells via increase of intracellular reactive oxygen species. Cancer Lett.

[622] Lu B, Wang ZQ, Ding Y, Wang XZ, Lu S, Wang CC (2018). RIP1 and RIP3 contribute to shikonin-induced glycolysis suppression in glioma cells via increase of intracellular hydrogen peroxide. Cancer Lett.

[623] Li Y, Lu H, Gu Y, Ning Z, Cao T, Chen C (2017). Enhancement of NK cells proliferation and function by Shikonin. Immunopharmacol Immunotoxicol.

[624] Yin SY, Efferth T, Jian FY, Chen YH, Liu CI, Wang AH (2016). Immunogenicity of mammary tumor cells can be induced by shikonin via direct binding-interference with hnRNPA1. Oncotarget..

[625] Chen HM, Wang PH, Aravindaram K, Chen YH, Yu HH, Yang WC (2012). Shikonin enhances efficacy of a gene-based cancer vaccine via induction of RANTES. J Biomed Sci.

[626] Yang YY, He HQ, Cui JH, Nie YJ, Wu YX, Wang R (2016). Shikonin derivative DMAKO-05 inhibits Akt signal activation and melanoma proliferation. Chem Biol Drug Des.

[627] Zhang X, Wang RB, Zhou W, Xiao S, Meng QQ, Li SS (2015). Antitumor activity of DMAKO-05, a novel shikonin derivative, and its metabolism in rat liver microsome. AAPS PharmSciTech.

[628] Durchschein C, Hufner A, Rinner B, Stallinger A, Deutsch A, Lohberger B (2018). Synthesis of novel shikonin derivatives and pharmacological effects of cyclopropylacetylshikonin on melanoma cells. Molecules (Basel, Switzerland)..

[629] Li S, Zhang T, Xu W, Ding J, Yin F, Xu J (2018). Sarcoma-targeting peptide-decorated polypeptide nanogel intracellularly delivers shikonin for upregulated osteosarcoma necroptosis and diminished pulmonary metastasis. Theranostics..

[630] Wen X, Li J, Cai D, Yue L, Wang Q, Zhou L (2018). Anticancer efficacy of targeted shikonin liposomes modified with RGD in breast cancer cells. Molecules (Basel, Switzerland)..

[631] Li W, Liu J, Jackson K, Shi R, Zhao Y (2014). Sensitizing the therapeutic efficacy of taxol with shikonin in human breast cancer cells. PLoS ONE.

[632] Wang Y, Zhou Y, Jia G, Han B, Liu J, Teng Y (2014). Shikonin suppresses tumor growth and synergizes with gemcitabine in a pancreatic cancer xenograft model: involvement of NF-kappaB signaling pathway. Biochem Pharmacol.

[633] Chen C, Xiao W, Huang L, Yu G, Ni J, Yang L (2017). Shikonin induces apoptosis and necroptosis in pancreatic cancer via regulating the expression of RIP1/RIP3 and synergizes the activity of gemcitabine. Am J Transl Res..

[634] Liu X, Sun G (2017). Shikonin enhances Adriamycin antitumor effects by inhibiting efflux pumps in A549 cells. Oncol Lett..

[635] Yang Q, Li S, Fu Z, Lin B, Zhou Z, Wang Z (2017). Shikonin promotes adriamycininduced apoptosis by upregulating caspase3 and caspase8 in osteosarcoma. Mol Med Rep..

[636] He G, He G, Zhou R, Pi Z, Zhu T, Jiang L (2016). Enhancement of cisplatin-induced colon cancer cells apoptosis by shikonin, a natural inducer of ROS in vitro and in vivo. Biochem Biophys Res Commun.

[637] Wang X, Chen W (2012). Gambogic acid is a novel anti-cancer agent that inhibits cell proliferation, angiogenesis and metastasis. Anticancer Agents Med Chem.

[638] Cascao R, Vidal B, Raquel H, Neves-Costa A, Figueiredo N, Gupta V (2014). Potent anti-inflammatory and antiproliferative effects of gambogic acid in a rat model of antigen-induced arthritis. Mediators Inflamm.

[639] Liu L, Qi XJ, Zhong ZK, Zhang EN (2016). Nanomedicine-based combination of gambogic acid and retinoic acid chlorochalcone for enhanced anticancer efficacy in osteosarcoma. Biomed Pharmacother.

[640] Zou Z, Xie L, Wei J, Yu L, Qian X, Chen J (2012). Synergistic anti-proliferative effects of gambogic acid with docetaxel in gastrointestinal cancer cell lines. BMC Complement Altern Med.

[641] Shi X, Chen X, Li X, Lan X, Zhao C, Liu S (2014). Gambogic acid induces apoptosis in imatinib-resistant chronic myeloid leukemia cells via inducing proteasome inhibition and caspase-dependent Bcr-Abl downregulation. Clin Cancer Res.

[642] Liang L, Zhang Z (2016). Gambogic acid inhibits malignant melanoma cell proliferation through mitochondrial p66shc/ROS-p53/Bax-mediated apoptosis. Cell Physiol Biochem.

[643] Pandey MK, Kale VP, Song C, Sung SS, Sharma AK, Talamo G (2014). Gambogic acid inhibits multiple myeloma mediated osteoclastogenesis through suppression of chemokine receptor CXCR651 signaling pathways. Exp Hematol.

[644] Jang JH, Kim JY, Sung EG, Kim EA, Lee TJ (2016). Gambogic acid induces apoptosis and sensitizes TRAIL-mediated apoptosis through downregulation of cFLIPL in renal carcinoma Caki cells. Int J Oncol.

[645] Lu N, Hui H, Yang H, Zhao K, Chen Y, You QD (2013). Gambogic acid inhibits angiogenesis through inhibiting PHD2-VHL-HIF-1alpha pathway. Eur J Pharm Sci..

[646] Pan H, Jansson KH, Beshiri ML, Yin J, Fang L, Agarwal S (2017). Gambogic acid inhibits thioredoxin activity and induces ROS-mediated cell death in castration-resistant prostate cancer. Oncotarget..

[647] Zhen YZ, Lin YJ, Li KJ, Yang XS, Zhao YF, Wei J (2015). Gambogic acid lysinate induces apoptosis in breast cancer mcf-7 cells by increasing reactive oxygen species. Evid Based Complement Altern Med..

[648] Li D, Song XY, Yue QX, Cui YJ, Liu M, Feng LX (2015). Proteomic and bioinformatic analyses of possible target-related proteins of gambogic acid in human breast carcinoma MDA-MB-231 cells. Chin J Nat Med.

[649] Wang S, Wang L, Chen M, Wang Y (2015). Gambogic acid sensitizes resistant breast cancer cells to doxorubicin through inhibiting P-glycoprotein and suppressing survivin expression. Chem Biol Interact.

[650] Wang S, Xu Y, Li C, Tao H, Wang A, Sun C (2018). Gambogic acid sensitizes breast cancer cells to TRAIL-induced apoptosis by promoting the crosstalk of extrinsic and intrinsic apoptotic signalings. Food Chem Toxicol.

[651] Li C, Qi Q, Lu N, Dai Q, Li F, Wang X (2012). Gambogic acid promotes apoptosis and resistance to metastatic potential in MDA-MB-231 human breast carcinoma cells. Biochem Cell Biol.

[652] Ishaq M, Khan MA, Sharma K, Sharma G, Dutta RK, Majumdar S (1840). Gambogic acid induced oxidative stress dependent caspase activation regulates both apoptosis and autophagy by targeting various key molecules (NF-kappaB, Beclin-1, p62 and NBR1) in human bladder cancer cells. Biochim Biophys Acta.

[653] Wang H, Zhao Z, Lei S, Li S, Xiang Z, Wang X (2019). Gambogic acid induces autophagy and combines synergistically with chloroquine to suppress pancreatic cancer by increasing the accumulation of reactive oxygen species. Cancer Cell Int.

[654] Ye L, Zhou J, Zhao W, Jiao P, Ren G, Wang S (2018). Gambogic acid-induced autophagy in nonsmall cell lung cancer NCI-H441 cells through a reactive oxygen species pathway. J Cancer Res Ther.

[655] Qi Q, Lu N, Li C, Zhao J, Liu W, You Q (2015). Involvement of RECK in gambogic acid induced anti-invasive effect in A549 human lung carcinoma cells. Mol Carcinog.

[656] Lu L, Tang D, Wang L, Huang LQ, Jiang GS, Xiao XY (2012). Gambogic acid inhibits TNF-alpha-induced invasion of human prostate cancer PC3 cells in vitro through PI3K/Akt and NF-kappaB signaling pathways. Acta Pharmacol Sin.

[657] Wan L, Zhang Q, Wang S, Gao Y, Chen X, Zhao Y (2019). Gambogic acid impairs tumor angiogenesis by targeting YAP/STAT3 signaling axis. Phytother Res..

[658] Lyu L, Huang LQ, Huang T, Xiang W, Yuan JD, Zhang CH (2018). Cell-penetrating peptide conjugates of gambogic acid enhance the antitumor effect on human bladder cancer EJ cells through ROS-mediated apoptosis. Drug Design Develop Ther.

[659] Zhu M, Jiang Y, Wu H, Shi W, Lu G, Cong D (2019). Gambogic acid shows anti-proliferative effects on non-small cell lung cancer (NSCLC) cells by activating reactive oxygen species (ROS)-induced endoplasmic reticulum (ER) stress-mediated apoptosis. Med Sci Moni.

[660] Lee JY, Lee BH, Lee JY (2015). Gambogic acid disrupts toll-like receptor4 activation by blocking lipopolysaccharides binding to myeloid differentiation factor 2. Toxicol Res.

[661] Ma J, Huang K, Ma Y, Chen S, Liu C, Shan Z (2018). Gambogic acid inhibits LPS-induced macrophage pro-inflammatory cytokine production mainly through suppression of the p38 pathway. Iran J Basic Med Sci.

[662] Liu F, Huang X, Han L, Sang M, Hu L, Liu B (2019). Improved druggability of gambogic acid using core-shell nanoparticles. Biomater Sci.

[663] Zhang W, Zhou H, Yu Y, Li J, Li H, Jiang D (2016). Combination of gambogic acid with cisplatin enhances the antitumor effects on cisplatin-resistant lung cancer cells by downregulating MRP2 and LRP expression. OncoTargets Ther..

[664] Xia G, Wang H, Song Z, Meng Q, Huang X, Huang X (2017). Gambogic acid sensitizes gemcitabine efficacy in pancreatic cancer by reducing the expression of ribonucleotide reductase subunit-M2 (RRM2). J Exp Clin Cancer Res.

[665] Wang J, Yuan Z (2013). Gambogic acid sensitizes ovarian cancer cells to doxorubicin through ROS-mediated apoptosis. Cell Biochem Biophys.

[666] Chi Y, Zhan XK, Yu H, Xie GR, Wang ZZ, Xiao W (2013). An open-labeled, randomized, multicenter phase IIa study of gambogic acid injection for advanced malignant tumors. Chin Med J.

[667] Augustin Y, Krishna S, Kumar D, Pantziarka PJE (2015). The wisdom of crowds and the repurposing of artesunate as an anticancer drug. Ecancermedicalscience..

[668] Ho WE, Peh HY, Chan TK, Wong WS (2014). Artemisinins: pharmacological actions beyond anti-malarial. Pharmacol Ther.

[669] Zuo W, Wang ZZ, Xue J (2014). Artesunate induces apoptosis of bladder cancer cells by miR-16 regulation of COX-2 expression. Int J Mol Sci.

[670] Greenshields AL, Fernando W, Hoskin DW (2019). The anti-malarial drug artesunate causes cell cycle arrest and apoptosis of triple-negative MDA-MB-468 and HER2-enriched SK-BR-3 breast cancer cells. Exp Mol Pathol.

[671] Zhang LX, Liu ZN, Ye J, Sha M, Qian H, Bu XH (2014). Artesunate exerts an anti-immunosuppressive effect on cervical cancer by inhibiting PGE2 production and Foxp3 expression. Cell Biol Int.

[672] Liu L, Zuo LF, Zuo J, Wang J (2015). Artesunate induces apoptosis and inhibits growth of Eca109 and Ec9706 human esophageal cancer cell lines in vitro and in vivo. Mol Med Rep..

[673] Wang L, Liu L, Wang J, Chen Y (2017). Inhibitory effect of artesunate on growth and apoptosis of gastric cancer cells. Arch Med Res.

[674] Chen X, Zhang XL, Zhang GH, Gao YF (2019). Artesunate promotes Th1 differentiation from CD4 + T cells to enhance cell apoptosis in ovarian cancer via miR-142. Braz J Med Biol Res.

[675] Zhou Y, Wang X, Zhang J, He A, Wang YL, Han K (2017). Artesunate suppresses the viability and mobility of prostate cancer cells through UCA1, the sponge of miR-184. Oncotarget..

[676] Jeong DE, Song HJ, Lim S, Lee SJ, Lim JE, Nam DH (2015). Repurposing the anti-malarial drug artesunate as a novel therapeutic agent for metastatic renal cell carcinoma due to its attenuation of tumor growth, metastasis, and angiogenesis. Oncotarget..

[677] Drenberg CD, Buaboonnam J, Orwick SJ, Hu S, Li L, Fan Y (2016). Evaluation of artemisinins for the treatment of acute myeloid leukemia. Cancer Chemother Pharmacol.

[678] Zheng L, Pan J (2018). The anti-malarial drug artesunate blocks Wnt/beta-catenin pathway and inhibits growth, migration and invasion of uveal melanoma cells. Curr Cancer Drug Targets.

[679] Chen H, Shi L, Yang X, Li S, Guo X, Pan L (2010). Artesunate inhibiting angiogenesis induced by human myeloma RPMI8226 cells. Int J Hematol.

[680] Krusche B, Arend J, Efferth T (2013). Synergistic inhibition of angiogenesis by artesunate and captopril in vitro and in vivo. Evid Based Complement Altern Med..

[681] Dong HY, Wang ZF (2014). Antitumor effects of artesunate on human breast carcinoma MCF-7 cells and IGF-IR expression in nude mice xenografts. Chin J Cancer Res.

[682] Chen X, Wong YK, Lim TK, Lim WH, Lin Q, Wang J (2017). Artesunate activates the intrinsic apoptosis of HCT116 cells through the suppression of fatty acid synthesis and the NF-kappaB pathway. Molecules (Basel, Switzerland)..

[683] Zhang P, Luo HS, Li M, Tan SY (2015). Artesunate inhibits the growth and induces apoptosis of human gastric cancer cells by downregulating COX-2. OncoTargets Ther..

[684] Greenshields AL, Shepherd TG, Hoskin DW (2017). Contribution of reactive oxygen species to ovarian cancer cell growth arrest and killing by the anti-malarial drug artesunate. Mol Carcinog.

[685] Chen K, Shou LM, Lin F, Duan WM, Wu MY, Xie X (2014). Artesunate induces G2/M cell cycle arrest through autophagy induction in breast cancer cells. Anticancer Drugs.

[686] Zhang J, Sun X, Wang L, Wong YK, Lee YM, Zhou C (2018). Artesunate-induced mitophagy alters cellular redox status. Redox Biol..

[687] Zhang L, Qian H, Sha M, Luan Z, Lin M, Yuan D (2016). Downregulation of HOTAIR expression mediated anti-metastatic effect of artesunate on cervical cancer by inhibiting COX-2 expression. PLoS ONE.

[688] Beccafico S, Morozzi G, Marchetti MC, Riccardi C, Sidoni A, Donato R (2015). Artesunate induces ROS- and p38 MAPK-mediated apoptosis and counteracts tumor growth in vivo in embryonal rhabdomyosarcoma cells. Carcinogenesis.

[689] Vatsveen TK, Myhre MR, Steen CB, Walchli S, Lingjaerde OC, Bai B (2018). Artesunate shows potent anti-tumor activity in B-cell lymphoma. J Hematol Oncol.

[690] Roh JL, Kim EH, Jang H, Shin D (2017). Nrf2 inhibition reverses the resistance of cisplatin-resistant head and neck cancer cells to artesunate-induced ferroptosis. Redox Biol.

[691] Cui C, Feng H, Shi X, Wang Y, Feng Z, Liu J (2015). Artesunate down-regulates immunosuppression from colorectal cancer Colon26 and RKO cells in vitro by decreasing transforming growth factor beta1 and interleukin-10. Int Immunopharmacol.

[692] Qian P, Zhang YW, Zhou ZH, Liu JQ, Yue SY, Guo XL (2018). Artesunate enhances gammadelta T-cell-mediated antitumor activity through augmenting gammadelta T-cell function and reversing immune escape of HepG2 cells. Immunopharmacol Immunotoxicol.

[693] Wang B, Hou D, Liu Q, Wu T, Guo H, Zhang X (2015). Artesunate sensitizes ovarian cancer cells to cisplatin by downregulating RAD51. Cancer Biol Ther.

[694] Efferth T, Ramirez T, Gebhart E, Halatsch ME (2004). Combination treatment of glioblastoma multiforme cell lines with the anti-malarial artesunate and the epidermal growth factor receptor tyrosine kinase inhibitor OSI-774. Biochem Pharmacol.

[695] Tran BN, Nguyen HT, Kim JO, Yong CS, Nguyen CN (2017). Developing combination of artesunate with paclitaxel loaded into poly-d, l-lactic-co-glycolic acid nanoparticle for systemic delivery to exhibit synergic chemotherapeutic response. Drug Dev Ind Pharm.

[696] Wang X, Du Q, Mao Z, Fan X, Hu B, Wang Z (2017). Combined treatment with artesunate and bromocriptine has synergistic anticancer effects in pituitary adenoma cell lines. Oncotarget..

[697] Osaki T, Uto Y, Ishizuka M, Tanaka T, Yamanaka N, Kurahashi T (2017). Artesunate enhances the cytotoxicity of 5-aminolevulinic acid-based sonodynamic therapy against mouse mammary tumor cells in vitro. Molecules (Basel, Switzerland)..

[698] Deeken JF, Wang H, Hartley M, Cheema AK, Smaglo B, Hwang JJ (2018). A phase I study of intravenous artesunate in patients with advanced solid tumor malignancies. Cancer Chemother Pharmacol.

[699] Krishna S, Ganapathi S, Ster IC, Saeed ME, Cowan M, Finlayson C (2015). A randomised, double blind, placebo-controlled pilot study of oral artesunate therapy for colorectal cancer. EBioMedicine..

[700] von Hagens C, Walter-Sack I, Goeckenjan M, Storch-Hagenlocher B, Sertel S, Elsasser M (2019). Long-term add-on therapy (compassionate use) with oral artesunate in patients with metastatic breast cancer after participating in a phase I study (ARTIC M33/2). Phytomedicine.

[701] von Hagens C, Walter-Sack I, Goeckenjan M, Osburg J, Storch-Hagenlocher B, Sertel S (2017). Prospective open uncontrolled phase I study to define a well-tolerated dose of oral artesunate as add-on therapy in patients with metastatic breast cancer (ARTIC M33/2). Breast Cancer Res Treat.

[702] Seong RK, Kim JA, Shin OS (2018). Wogonin, a flavonoid isolated from *Scutellaria baicalensis*, has anti-viral activities against influenza infection via modulation of AMPK pathways. Acta Virol.

[703] Khan S, Zhang D, Zhang Y, Li M, Wang C (2016). Wogonin attenuates diabetic cardiomyopathy through its anti-inflammatory and anti-oxidative properties. Mol Cell Endocrinol.

[704] Ge W, Yin Q, Xian H (2015). Wogonin induced mitochondrial dysfunction and endoplasmic reticulum stress in human malignant neuroblastoma cells via ire1alpha-dependent pathway. J Mol Neurosci.

[705] Xu Y, Yang B, Hu Y, Lu L, Lu X, Wang J (2016). Wogonin prevents TLR4-NF-kappaB-medicated neuro-inflammation and improves retinal ganglion cells survival in retina after optic nerve crush. Oncotarget..

[706] Chen XM, Bai Y, Zhong YJ, Xie XL, Long HW, Yang YY (2013). Wogonin has multiple anti-cancer effects by regulating c-Myc/SKP2/Fbw7alpha and HDAC1/HDAC2 pathways and inducing apoptosis in human lung adenocarcinoma cell line A549. PLoS ONE.

[707] Yao Y, Zhao K, Yu Z, Ren H, Zhao L, Li Z (2017). Wogonoside inhibits invasion and migration through suppressing TRAF2/4 expression in breast cancer. J Exp Clin Cancer Res: CR..

[708] Kim EH, Jang H, Shin D, Baek SH, Roh JL (2016). Targeting Nrf2 with wogonin overcomes cisplatin resistance in head and neck cancer. Apoptosis.

[709] Wang T, Gao J, Yu J, Shen L (2013). Synergistic inhibitory effect of wogonin and low-dose paclitaxel on gastric cancer cells and tumor xenografts. Chin J Cancer Res.

[710] Feng Q, Wang H, Pang J, Ji L, Han J, Wang Y (2018). Prevention of wogonin on colorectal cancer tumorigenesis by regulating p53 nuclear translocation. Front Pharmacol.

[711] Tsai CF, Yeh WL, Huang SM, Tan TW, Lu DY (2012). Wogonin induces reactive oxygen species production and cell apoptosis in human glioma cancer cells. Int J Mol Sci.

[712] Durr C, Hanna BS, Schulz A, Lucas F, Zucknick M, Benner A (2018). Tumor necrosis factor receptor signaling is a driver of chronic lymphocytic leukemia that can be therapeutically targeted by the flavonoid wogonin. Haematologica.

[713] Xu PP, Zuo HQ, Zhou RF, Chen B, Ouyang J (2018). Wogonin inhibits growth of mantle cell lymphoma cells through nuclear factor-kappab signaling pathway. Chin Med J.

[714] Lin CC, Kuo CL, Lee MH, Lai KC, Lin JP, Yang JS (2011). Wogonin triggers apoptosis in human osteosarcoma U-2 OS cells through the endoplasmic reticulum stress, mitochondrial dysfunction and caspase-3-dependent signaling pathways. Int J Oncol.

[715] Song X, Yao J, Wang F, Zhou M, Zhou Y, Wang H (2013). Wogonin inhibits tumor angiogenesis via degradation of HIF-1alpha protein. Toxicol Appl Pharmacol.

[716] Ruibin J, Bo J, Danying W, Chihong Z, Jianguo F, Linhui G (2017). Therapy effects of wogonin on ovarian cancer cells. Biomed Res Int.

[717] Liu X, Tian S, Liu M, Jian L, Zhao L (2016). Wogonin inhibits the proliferation and invasion, and induces the apoptosis of HepG2 and Bel7402 HCC cells through NFkappaB/Bcl-2, EGFR and EGFR downstream ERK/AKT signaling. Int J Mol Med.

[718] He L, Lu N, Dai Q, Zhao Y, Zhao L, Wang H (2013). Wogonin induced G1 cell cycle arrest by regulating Wnt/beta-catenin signaling pathway and inactivating CDK8 in human colorectal cancer carcinoma cells. Toxicology.

[719] Shi G, Wang Q, Zhou X, Li J, Liu H, Gu J (2017). Response of human non-small-cell lung cancer cells to the influence of Wogonin with SGK1 dynamics. Acta Biochim Biophys Sin.

[720] Yang H, Hui H, Wang Q, Li H, Zhao K, Zhou Y (2014). Wogonin induces cell cycle arrest and erythroid differentiation in imatinib-resistant K562 cells and primary CML cells. Oncotarget..

[721] Li SJ, Sun SJ, Gao J, Sun FB (2016). Wogonin induces Beclin-1/PI3K and reactive oxygen species-mediated autophagy in human pancreatic cancer cells. Oncol Lett.

[722] Chow SE, Chen YW, Liang CA, Huang YK, Wang JS (2012). Wogonin induces cross-regulation between autophagy and apoptosis via a variety of Akt pathway in human nasopharyngeal carcinoma cells. J Cell Biochem.

[723] Zhao Y, Yao J, Wu XP, Zhao L, Zhou YX, Zhang Y (2015). Wogonin suppresses human alveolar adenocarcinoma cell A549 migration in inflammatory microenvironment by modulating the IL-6/STAT3 signaling pathway. Mol Carcinog.

[724] Hong M, Cheng H, Song L, Wang W, Wang Q, Xu D (2018). Wogonin suppresses the activity of matrix metalloproteinase-9 and inhibits migration and invasion in human hepatocellular carcinoma. Molecules..

[725] Fu R, Chen Y, Wang XP, An T, Tao L, Zhou YX (2016). Wogonin inhibits multiple myeloma-stimulated angiogenesis via c-Myc/VHL/HIF-1alpha signaling axis. Oncotarget..

[726] Zhao K, Song X, Huang Y, Yao J, Zhou M, Li Z (2014). Wogonin inhibits LPS-induced tumor angiogenesis via suppressing PI3K/Akt/NF-kappaB signaling. Eur J Pharmacol.

[727] Zhou M, Song X, Huang Y, Wei L, Li Z, You Q (2014). Wogonin inhibits H2O2-induced angiogenesis via suppressing PI3K/Akt/NF-kappaB signaling pathway. Vascul Pharmacol.

[728] Hu C, Xu M, Qin R, Chen W, Xu X (2015). Wogonin induces apoptosis and endoplasmic reticulum stress in HL-60 leukemia cells through inhibition of the PI3K-AKT signaling pathway. Oncol Rep.

[729] Yang L, Wang Q, Li D, Zhou Y, Zheng X, Sun H (2013). Wogonin enhances antitumor activity of tumor necrosis factor-related apoptosis-inducing ligand in vivo through ROS-mediated downregulation of cFLIPL and IAP proteins. Apoptosis.

[730] Wu X, Liu P, Zhang H, Li Y, Salmani JM, Wang F (2017). Wogonin as a targeted therapeutic agent for EBV (+) lymphoma cells involved in LMP1/NF-kappaB/miR-155/PU1 pathway. BMC Cancer..

[731] Xiao W, Wu K, Yin M, Han S, Ding Y, Qiao A (2015). Wogonin inhibits tumor-derived regulatory molecules by suppressing STAT3 signaling to promote tumor immunity. J Immunother (Hagerstown, Md: 1997)..

[732] Yang Y, Li XJ, Chen Z, Zhu XX, Wang J, Zhang LB (2012). Wogonin induced calreticulin/annexin A1 exposure dictates the immunogenicity of cancer cells in a PERK/AKT dependent manner. PLoS ONE.

[733] Chen F, Qin X, Xu G, Gou S, Jin X (2017). Reversal of cisplatin resistance in human gastric cancer cells by a wogonin-conjugated Pt(IV) prodrug via attenuating Casein Kinase 2-mediated Nuclear Factor-kappaB pathways. Biochem Pharmacol.

[734] Qin X, Xu G, Chen F, Fang L, Gou S (2017). Novel platinum(IV) complexes conjugated with a wogonin derivative as multi-targeted anticancer agents. Bioorg Med Chem.

[735] Zhao L, Miao HC, Li WJ, Sun Y, Huang SL, Li ZY (2016). LW-213 induces G2/M cell cycle arrest through AKT/GSK3beta/beta-catenin signaling pathway in human breast cancer cells. Mol Carcinog.

[736] Li L, Chen P, Ling Y, Song X, Lu Z, He Q (2011). Inhibitory effects of GL-V9 on the invasion of human breast carcinoma cells by downregulating the expression and activity of matrix metalloproteinase-2/9. Eur J Pharm Sci..

[737] Li L, Lu N, Dai Q, Wei L, Zhao Q, Li Z (2011). GL-V9, a newly synthetic flavonoid derivative, induces mitochondrial-mediated apoptosis and G2/M cell cycle arrest in human hepatocellular carcinoma HepG2 cells. Eur J Pharmacol.

[738] Zhao K, Li G, Yao Y, Zhou Y, Li Z, Guo Q (2017). Activation of phospholipase C-gamma1 and translocation of phosphatidylinositol-3,4,5-trisphosphate 3-phosphatase contribute to GL-V9-induced apoptosis in human gastric cancer cells. Exp Cell Res.

[739] Zhang X, Kang Y, Huo T, Tao R, Wang X, Li Z (2017). GL-V9 induced upregulation and mitochondrial localization of NAG-1 associates with ROS generation and cell death in hepatocellular carcinoma cells. Free Radical Biol Med.

[740] Tian J, Wang L, Wang L, Ke X (2014). A wogonin-loaded glycyrrhetinic acid-modified liposome for hepatic targeting with anti-tumor effects. Drug Delivery.

[741] Hong ZP, Wang LG, Wang HJ, Ye WF, Wang XZ (2018). Wogonin exacerbates the cytotoxic effect of oxaliplatin by inducing nitrosative stress and autophagy in human gastric cancer cells. Phytomedicine.

[742] Rong LW, Wang RX, Zheng XL, Feng XQ, Zhang L, Zhang L (2017). Combination of wogonin and sorafenib effectively kills human hepatocellular carcinoma cells through apoptosis potentiation and autophagy inhibition. Oncol Lett.

[743] He F, Wang Q, Zheng XL, Yan JQ, Yang L, Sun H (2012). Wogonin potentiates cisplatin-induced cancer cell apoptosis through accumulation of intracellular reactive oxygen species. Oncol Rep.

[744] Liu M, Chen X, Ma J, Hassan W, Wu H, Ling J (2017). beta-Elemene attenuates atherosclerosis in apolipoprotein E-deficient mice via restoring NO levels and alleviating oxidative stress. Biomed Pharmacother.

[745] Fang Y, Kang Y, Zou H, Cheng X, Xie T, Shi L (2018). beta-elemene attenuates macrophage activation and proinflammatory factor production via crosstalk with Wnt/beta-catenin signaling pathway. Fitoterapia.

[746] Cai B, Ma L, Nong S, Wu Y, Guo X, Pu J (2018). beta-elemene induced anticancer effect in bladder cancer through upregulation of PTEN and suppression of AKT phosphorylation. Oncol Lett.

[747] Liu Y, Jiang ZY, Zhou YL, Qiu HH, Wang G, Luo Y (2017). beta-elemene regulates endoplasmic reticulum stress to induce the apoptosis of NSCLC cells through PERK/IRE1alpha/ATF6 pathway. Biomed Pharmacother.

[748] Liu J, Zhang Y, Qu J, Xu L, Hou K, Zhang J (2011). beta-Elemene-induced autophagy protects human gastric cancer cells from undergoing apoptosis. BMC Cancer..

[749] Wang L, Zhao Y, Wu Q, Guan Y, Wu X (2018). Therapeutic effects of beta-elemene via attenuation of the Wnt/beta-catenin signaling pathway in cervical cancer cells. Mol Med Rep..

[750] Zhang X, Zhang Y, Li Y (2013). beta-elemene decreases cell invasion by upregulating E-cadherin expression in MCF-7 human breast cancer cells. Oncol Rep.

[751] Liang D, Yang M, Guo B, Yang L, Cao J, Zhang X (2012). HIF-1alpha induced by beta-elemene protects human osteosarcoma cells from undergoing apoptosis. J Cancer Res Clin Oncol.

[752] Chen W, Lu Y, Wu J, Gao M, Wang A, Xu B (2011). Beta-elemene inhibits melanoma growth and metastasis via suppressing vascular endothelial growth factor-mediated angiogenesis. Cancer Chemother Pharmacol.

[753] Hu T, Gao Y (2018). beta-elemene against Burkitt’s lymphoma via activation of PUMA mediated apoptotic pathway. Biomed Pharmacother.

[754] Wu Z, Wang T, Zhang Y, Zheng Z, Yu S, Jing S (2017). Anticancer effects of beta-elemene with hyperthermia in lung cancer cells. Exp Ther Med.

[755] Zheng F, Tang Q, Zheng XH, Wu J, Huang H, Zhang H (2018). Inactivation of Stat3 and crosstalk of miRNA155-5p and FOXO3a contribute to the induction of IGFBP1 expression by beta-elemene in human lung cancer. Exp Mol Med.

[756] Zhu T, Zhao Y, Zhang J, Li L, Zou L, Yao Y (2011). ss-Elemene inhibits proliferation of human glioblastoma cells and causes cell-cycle G0/G1 arrest via mutually compensatory activation of MKK3 and MKK6. Int J Oncol.

[757] Liu J, Hu XJ, Jin B, Qu XJ, Hou KZ, Liu YP (2012). beta-Elemene induces apoptosis as well as protective autophagy in human non-small-cell lung cancer A549 cells. J Pharm Pharmacol..

[758] Zhou K, Wang L, Cheng R, Liu X, Mao S, Yan Y (2017). Elemene increases autophagic apoptosis and drug sensitivity in human cisplatin (DDP)-resistant lung cancer cell line SPC-A-1/DDP by inducing beclin-1 expression. Oncol Res..

[759] Zhang Y, Sun X, Nan N, Cao KX, Ma C, Yang GW (2017). Elemene inhibits the migration and invasion of 4T1 murine breast cancer cells via heparanase. Mol Med Rep..

[760] Lin L, Li L, Chen X, Zeng B, Lin T (2018). Preliminary evaluation of the potential role of beta-elemene in reversing erlotinib-resistant human NSCLC A549/ER cells. Oncol Lett..

[761] Yao CC, Tu YR, Jiang J, Ye SF, Du HX, Zhang Y (2014). beta-elemene reverses the drug resistance of lung cancer A549/DDP cells via the mitochondrial apoptosis pathway. Oncol Rep.

[762] Yu X, Xu M, Li N, Li Z, Li H, Shao S (2017). beta-elemene inhibits tumor-promoting effect of M2 macrophages in lung cancer. Biochem Biophys Res Commun.

[763] Shi F, Yang G, Ren J, Guo T, Du Y, Feng N (2013). Formulation design, preparation, and in vitro and in vivo characterizations of beta-Elemene-loaded nanostructured lipid carriers. Int J Nanomed.

[764] Zeng Y-Y, Zeng Y-J, Zhang N-N, Li C-X, Xie T, Zeng Z-W (2019). The preparation, determination of a flexible complex liposome co-loaded with cabazitaxel and β-elemene, and animal pharmacodynamics on paclitaxel-resistant lung adenocarcinoma. Molecules (Basel, Switzerland)..

[765] Hu CJ, Zhao XL, Li JZ, Kang SM, Yang CR, Jin YH (2011). Preparation and characterization of beta-elemene-loaded microemulsion. Drug Dev Ind Pharm.

[766] Yu Z, Wu F, Chen L, Li Q, Wang C, Dong J (2014). ETME, a novel beta-elemene derivative, synergizes with arsenic trioxide in inducing apoptosis and cell cycle arrest in hepatocarcinoma cells via a p53-dependent pathway. Acta Pharm Sinica B..

[767] Ding XF, Shen M, Xu LY, Dong JH, Chen G (2013). 13,14-bis(cis-3,5-dimethyl-1-piperazinyl)-beta-elemene, a novel beta-elemene derivative, shows potent antitumor activities via inhibition of mTOR in human breast cancer cells. Oncol Lett.

[768] Huang C, Yu Y (2017). Synergistic cytotoxicity of beta-elemene and cisplatin in gingival squamous cell carcinoma by inhibition of STAT3 signaling pathway. Med Sci Monit.

[769] Mu L, Wang T, Chen Y, Tang X, Yuan Y, Zhao Y (2016). beta-Elemene enhances the efficacy of gefitinib on glioblastoma multiforme cells through the inhibition of the EGFR signaling pathway. Int J Oncol.

[770] Zhang J, Zhang HD, Yao YF, Zhong SL, Zhao JH, Tang JH (2015). beta-elemene reverses chemoresistance of breast cancer cells by reducing resistance transmission via exosomes. Cell Physiol Biochem.

[771] Xu L, Guo T, Qu X, Hu X, Zhang Y, Che X (2018). beta-elemene increases the sensitivity of gastric cancer cells to TRAIL by promoting the formation of DISC in lipid rafts. Cell Biol Int.

[772] Matsuda K, Hattori S, Komizu Y, Kariya R, Ueoka R, Okada S (2014). Cepharanthine inhibited HIV-1 cell–cell transmission and cell-free infection via modification of cell membrane fluidity. Bioorg Med Chem Lett.

[773] Rogosnitzky M, Danks R (2011). Therapeutic potential of the biscoclaurine alkaloid, cepharanthine, for a range of clinical conditions. Pharmacol Rep.

[774] Desgrouas C, Chapus C, Desplans J, Travaille C, Pascual A, Baghdikian B (2014). In vitro antiplasmodial activity of cepharanthine. Malaria J.

[775] Paudel KR, Karki R, Kim DW (2016). Cepharanthine inhibits in vitro VSMC proliferation and migration and vascular inflammatory responses mediated by RAW264.7. Toxicol Vitro..

[776] Uthaisar K, Seubwai W, Srikoon P, Vaeteewoottacharn K, Sawanyawisuth K, Okada S (2012). Cepharanthine suppresses metastatic potential of human cholangiocarcinoma cell lines. Asian Pac J Cancer Prevent.

[777] Lyu J, Yang EJ, Head SA, Ai N, Zhang B, Wu C (2017). Pharmacological blockade of cholesterol trafficking by cepharanthine in endothelial cells suppresses angiogenesis and tumor growth. Cancer Lett.

[778] Harada T, Harada K, Ueyama Y (2012). The enhancement of tumor radioresponse by combined treatment with cepharanthine is accompanied by the inhibition of DNA damage repair and the induction of apoptosis in oral squamous cell carcinoma. Int J Oncol.

[779] Li H, Yan Z, Ning W, Xiao-Juan G, Cai-Hong Z, Jin-Hua J (2011). Using rhodamine 123 accumulation in CD8 + cells as a surrogate indicator to study the P-glycoprotein modulating effect of cepharanthine hydrochloride in vivo. J Biomed Biotechnol.

[780] Zhou P, Zhang R, Wang Y, Xu D, Zhang L, Qin J (2017). Cepharanthine hydrochloride reverses the mdr1 (P-glycoprotein)-mediated esophageal squamous cell carcinoma cell cisplatin resistance through JNK and p53 signals. Oncotarget..

[781] Han L, Wang Y, Guo X, Zhou Y, Zhang J, Wang N (2014). Downregulation of MDR1 gene by cepharanthine hydrochloride is related to the activation of c-Jun/JNK in K562/ADR cells. Biomed Res Int.

[782] Tang ZH, Cao WX, Guo X, Dai XY, Lu JH, Chen X (2018). Identification of a novel autophagic inhibitor cepharanthine to enhance the anti-cancer property of dacomitinib in non-small cell lung cancer. Cancer Lett.

[783] Rattanawong A, Payon V, Limpanasittikul W, Boonkrai C, Mutirangura A, Wonganan P (2018). Cepharanthine exhibits a potent anticancer activity in p53-mutated colorectal cancer cells through upregulation of p21Waf1/Cip1. Oncol Rep.

[784] Zhu Q, Guo B, Chen L, Ji Q, Liang H, Wen N (2017). Cepharanthine exerts antitumor activity on choroidal melanoma by reactive oxygen species production and c-Jun N-terminal kinase activation. Oncol Lett.

[785] Gao S, Li X, Ding X, Qi W, Yang Q (2017). Cepharanthine induces autophagy, apoptosis and cell cycle arrest in breast cancer cells. Cell Physiol Biochem.

[786] Hua P, Sun M, Zhang G, Zhang Y, Tian X, Li X (2015). Cepharanthine induces apoptosis through reactive oxygen species and mitochondrial dysfunction in human non-small-cell lung cancer cells. Biochem Biophys Res Commun.

[787] Payon V, Kongsaden C, Ketchart W, Mutirangura A, Wonganan P (2019). Mechanism of cepharanthine cytotoxicity in human ovarian cancer cells. Planta Med.

[788] Huang CZ, Wang YF, Zhang Y, Peng YM, Liu YX, Ma F (2017). Cepharanthine hydrochloride reverses Pglycoprotein-mediated multidrug resistance in human ovarian carcinoma A2780/Taxol cells by inhibiting the PI3K/Akt signaling pathway. Oncol Rep.

[789] Liu G, Wu D, Liang X, Yue H, Cui Y (2015). Mechanisms and in vitro effects of cepharanthine hydrochloride: classification analysis of the drug-induced differentially-expressed genes of human nasopharyngeal carcinoma cells. Oncol Rep.

[790] Xu W, Wang X, Tu Y, Masaki H, Tanaka S, Onda K (2019). Tetrandrine and cepharanthine induce apoptosis through caspase cascade regulation, cell cycle arrest, MAPK activation and PI3K/Akt/mTOR signal modification in glucocorticoid resistant human leukemia Jurkat T cells. Chem-Biol Interactions..

[791] Law BY, Chan WK, Xu SW, Wang JR, Bai LP, Liu L (2014). Natural small-molecule enhancers of autophagy induce autophagic cell death in apoptosis-defective cells. Sci Rep..

[792] Villeneuve NF, Sun Z, Chen W, Zhang DD (2009). Nrf2 and p21 regulate the fine balance between life and death by controlling ROS levels. Cell Cycle (Georgetown, Tex)..

[793] Uto T, Nishi Y, Toyama M, Yoshinaga K, Baba M (2011). Inhibitory effect of cepharanthine on dendritic cell activation and function. Int Immunopharmacol.

[794] Yu HH, Mi WN, Liu B, Zhao HP (2016). In vitro and in vivo effect of paclitaxel and cepharanthine co-loaded polymeric nanoparticles in gastric cancer. J BUON.

[795] Zhang LH, Yang GL, Zhang RY, Dong L, Chen HG, Bo JJ (2018). Curcumin inhibits cell proliferation and motility via suppression of TROP2 in bladder cancer cells. Int J Oncol.

[796] Yoshida K, Toden S, Ravindranathan P, Han H, Goel A (2017). Curcumin sensitizes pancreatic cancer cells to gemcitabine by attenuating PRC2 subunit EZH2, and the lncRNA PVT1 expression. Carcinogenesis.

[797] Thacker PC, Karunagaran D (2015). Curcumin and emodin down-regulate TGF-β signaling pathway in human cervical cancer cells. PLoS ONE..

[798] Link A, Balaguer F, Shen Y, Lozano JJ, Leung H-CE, Boland CR (2013). Curcumin modulates DNA methylation in colorectal cancer cells. PLoS ONE..

[799] Lim T-G, Lee S-Y, Huang Z, Lim DY, Chen H, Jung SK (2014). Curcumin suppresses proliferation of colon cancer cells by targeting CDK2. Cancer Prevent Res (Philadelphia, Pa)..

[800] Murray-Stewart T, Dunworth M, Lui Y, Giardiello FM, Woster PM, Casero RA (2018). Curcumin mediates polyamine metabolism and sensitizes gastrointestinal cancer cells to antitumor polyamine-targeted therapies. PLoS ONE..

[801] Shao S, Duan W, Xu Q, Li X, Han L, Li W (2019). Curcumin suppresses hepatic stellate cell-induced hepatocarcinoma angiogenesis and invasion through downregulating CTGF. Oxid Med Cell Longevity..

[802] Du WZ, Feng Y, Wang XF, Piao XY, Cui YQ, Chen LC (2013). Curcumin suppresses malignant glioma cells growth and induces apoptosis by inhibition of SHH/GLI1 signaling pathway in vitro and vivo. CNS Neurosci Ther.

[803] Gao SM, Yang JJ, Chen CQ, Chen JJ, Ye LP, Wang LY (2012). Pure curcumin decreases the expression of WT1 by upregulation of miR-15a and miR-16-1 in leukemic cells. J Exp Clin Cancer Res: CR..

[804] Marquardt JU, Gomez-Quiroz L, Camacho LOA, Pinna F, Hepatology SS (2015). Curcumin effectively inhibits oncogenic NF-κB signaling and restrains stemness features in liver cancer. J Hepatol..

[805] Wu G-Q, Chai K-Q, Zhu X-M, Jiang H, Wang X, Xue Q (2016). Anti-cancer effects of curcumin on lung cancer through the inhibition of EZH2 and NOTCH1. Oncotarget..

[806] Zhang C, Hao Y, Wu L, Dong X, Jiang N, Cong B (2018). Curcumin induces apoptosis and inhibits angiogenesis in murine malignant mesothelioma. Int J Oncol.

[807] Sidhar H, Giri RK (2017). Induction of Bex genes by curcumin is associated with apoptosis and activation of p53 in N2a neuroblastoma cells. Sci Rep..

[808] Dudás J, Fullár A, Romani A, Pritz C, Kovalszky I, Hans Schartinger V (2013). Curcumin targets fibroblast-tumor cell interactions in oral squamous cell carcinoma. Exp Cell Res.

[809] Zhou X, Su J, Feng S, Wang L, Yin X, Yan J (2016). Antitumor activity of curcumin is involved in down-regulation of YAP/TAZ expression in pancreatic cancer cells. Oncotarget..

[810] Huang H, Chen X, Li D, He Y, Li Y, Du Z (2015). Combination of α-tomatine and curcumin inhibits growth and induces apoptosis in human prostate cancer cells. PLoS ONE..

[811] Sundram V, Chauhan SC, Ebeling M, Jaggi M (2012). Curcumin attenuates β-catenin signaling in prostate cancer cells through activation of protein kinase D1. PLoS ONE..

[812] Seo BR, Min K-J, Cho IJ, Kim SC, Kwon TK (2014). Curcumin significantly enhances dual PI3K/Akt and mTOR inhibitor NVP-BEZ235-induced apoptosis in human renal carcinoma Caki cells through down-regulation of p53-dependent Bcl-2 expression and inhibition of Mcl-1 protein stability. PLoS ONE..

[813] Li Y, Sun W, Han N, Zou Y, Yin D (2018). Curcumin inhibits proliferation, migration, invasion and promotes apoptosis of retinoblastoma cell lines through modulation of miR-99a and JAK/STAT pathway. BMC Cancer..

[814] Amin ARMR, Haque A, Rahman MA, Chen ZG, Khuri FR, Shin DM (2015). Curcumin induces apoptosis of upper aerodigestive tract cancer cells by targeting multiple pathways. PLoS One..

[815] Tang H, Zeng L, Wang J, Zhang X, Ruan Q, Wang J (2017). Reversal of 5-fluorouracil resistance by EGCG is mediate by inactivation of TFAP2A/VEGF signaling pathway and down-regulation of MDR-1 and P-gp expression in gastric cancer. Oncotarget..

[816] Zhu BH, Chen HY, Zhan WH, Wang CY, Cai SR, Wang Z (2011). (−)-Epigallocatechin-3-gallate inhibits VEGF expression induced by IL-6 via Stat3 in gastric cancer. World J Gastroenterol.

[817] Chen PN, Chu SC, Kuo WH, Chou MY, Lin JK, Hsieh YS (2011). Epigallocatechin-3 gallate inhibits invasion, epithelial-mesenchymal transition, and tumor growth in oral cancer cells. J Agric Food Chem.

[818] Sharma C, Nusri Qel A, Begum S, Javed E, Rizvi TA, Hussain A (2012). (-)-Epigallocatechin-3-gallate induces apoptosis and inhibits invasion and migration of human cervical cancer cells. Asian Pac J Cancer Prevent.

[819] Zhang X, Min KW, Wimalasena J, Baek SJ (2012). Cyclin D1 degradation and p21 induction contribute to growth inhibition of colorectal cancer cells induced by epigallocatechin-3-gallate. J Cancer Res Clin Oncol.

[820] Kostin SF, McDonald DE, McFadden DW (2012). Inhibitory effects of (-)-epigallocatechin-3-gallate and pterostilbene on pancreatic cancer growth in vitro. J Surg Res.

[821] Min NY, Kim JH, Choi JH, Liang W, Ko YJ, Rhee S (2012). Selective death of cancer cells by preferential induction of reactive oxygen species in response to (-)-epigallocatechin-3-gallate. Biochem Biophys Res Commun.

[822] Mineva ND, Paulson KE, Naber SP, Yee AS, Sonenshein GE (2013). Epigallocatechin-3-gallate inhibits stem-like inflammatory breast cancer cells. PLoS ONE.

[823] Jang JY, Lee JK, Jeon YK, Kim CW (2013). Exosome derived from epigallocatechin gallate treated breast cancer cells suppresses tumor growth by inhibiting tumor-associated macrophage infiltration and M2 polarization. BMC Cancer..

[824] Lee SH, Nam HJ, Kang HJ, Kwon HW, Lim YC (2013). Epigallocatechin-3-gallate attenuates head and neck cancer stem cell traits through suppression of Notch pathway. Eur J Cancer (Oxford, England: 1990)..

[825] Singh T, Katiyar SK (2013). Green tea polyphenol, (-)-epigallocatechin-3-gallate, induces toxicity in human skin cancer cells by targeting beta-catenin signaling. Toxicol Appl Pharmacol.

[826] Hwang YS, Park KK, Chung WY (2013). Epigallocatechin-3 gallate inhibits cancer invasion by repressing functional invadopodia formation in oral squamous cell carcinoma. Eur J Pharmacol.

[827] Wang S, Chen R, Zhong Z, Shi Z, Chen M, Wang Y (2014). Epigallocatechin-3-gallate potentiates the effect of curcumin in inducing growth inhibition and apoptosis of resistant breast cancer cells. Am J Chin Med.

[828] Kim KC, Lee C (2014). Reversal of Cisplatin resistance by epigallocatechin gallate is mediated by downregulation of axl and tyro 3 expression in human lung cancer cells. Korean J Physiol Pharmacol.

[829] Lin CH, Chao LK, Hung PH, Chen YJ (2014). EGCG inhibits the growth and tumorigenicity of nasopharyngeal tumor-initiating cells through attenuation of STAT3 activation. Int J Clin Exp Pathol..

[830] Bimonte S, Leongito M, Barbieri A, Del Vecchio V, Barbieri M, Albino V (2015). Inhibitory effect of (-)-epigallocatechin-3-gallate and bleomycin on human pancreatic cancer MiaPaca-2 cell growth. Infect Agents Cancer..

[831] Mayr C, Wagner A, Neureiter D, Pichler M, Jakab M, Illig R (2015). The green tea catechin epigallocatechin gallate induces cell cycle arrest and shows potential synergism with cisplatin in biliary tract cancer cells. BMC Complement Altern Med.

[832] Moses MA, Henry EC, Ricke WA, Gasiewicz TA (2015). The heat shock protein 90 inhibitor, (-)-epigallocatechin gallate, has anticancer activity in a novel human prostate cancer progression model. Cancer Prevent Res (Philadelphia, Pa)..

[833] De Amicis F, Russo A, Avena P, Santoro M, Vivacqua A, Bonofiglio D (2013). In vitro mechanism for downregulation of ER-alpha expression by epigallocatechin gallate in ER +/PR + human breast cancer cells. Mol Nutr Food Res.

[834] Pan X, Zhao B, Song Z, Han S, Wang M (2016). Estrogen receptor-alpha36 is involved in epigallocatechin-3-gallate induced growth inhibition of ER-negative breast cancer stem/progenitor cells. J Pharmacol Sci.

[835] Li X, Zhang A, Sun H, Liu Z, Zhang T, Qiu S (2017). Metabolic characterization and pathway analysis of berberine protects against prostate cancer. Oncotarget..

[836] Park JJ, Seo SM, Kim EJ, Lee YJ, Ko YG, Ha J (2012). Berberine inhibits human colon cancer cell migration via AMP-activated protein kinase-mediated downregulation of integrin beta1 signaling. Biochem Biophys Res Commun.

[837] Kim J-H, Ryu AR, Kang M-J, Lee M-Y (2016). Berberine-induced changes in protein expression and antioxidant enzymes in melanoma cells. Mol Cell Toxicol.

[838] Wu HL, Chuang TY, Al-Hendy A, Diamond MP, Azziz R, Chen YH (2015). Berberine inhibits the proliferation of human uterine leiomyoma cells. Fertil Steril.

[839] Pazhang Y, Ahmadian S, Mahmoudian M, Shafiezadeh M (2011). Berberine-induced apoptosis via decreasing the survivin protein in K562 cell line. Med Oncol (Northwood, London, England)..

[840] Ke R, Vishnoi K, Viswakarma N, Santha S, Das S, Rana A (2018). Involvement of AMP-activated protein kinase and death receptor 5 in TRAIL-berberine-induced apoptosis of cancer cells. Sci Rep..

[841] Li J, Li O, Kan M, Zhang M, Shao D, Pan Y (2015). Berberine induces apoptosis by suppressing the arachidonic acid metabolic pathway in hepatocellular carcinoma. Mol Med Rep..

[842] Jeong Y, You D, Kang HG, Yu J, Kim SW, Nam SJ (2018). Berberine suppresses fibronectin expression through inhibition of c-Jun phosphorylation in breast cancer cells. J Breast Cancer..

[843] Kim JS, Oh D, Yim MJ, Park JJ, Kang KR, Cho IA (2015). Berberine induces FasL-related apoptosis through p38 activation in KB human oral cancer cells. Oncol Rep.

[844] Marverti G, Ligabue A, Lombardi P, Ferrari S, Monti MG, Frassineti C (2013). Modulation of the expression of folate cycle enzymes and polyamine metabolism by berberine in cisplatin-sensitive and -resistant human ovarian cancer cells. Int J Oncol.

[845] He W, Wang B, Zhuang Y, Shao D, Sun K, Chen J (2012). Berberine inhibits growth and induces G1 arrest and apoptosis in human cholangiocarcinoma QBC939 cells. J Pharmacol Sci.

[846] Wang J, Peng Y, Liu Y, Yang J, Ding N, Tan W (2015). Berberine, a natural compound, suppresses Hedgehog signaling pathway activity and cancer growth. BMC Cancer..

[847] Park SH, Sung JH, Chung N (2014). Berberine diminishes side population and down-regulates stem cell-associated genes in the pancreatic cancer cell lines PANC-1 and MIA PaCa-2. Mol Cell Biochem.

[848] Wang C, Wang H, Zhang Y, Guo W, Long C, Wang J (2017). Berberine inhibits the proliferation of human nasopharyngeal carcinoma cells via an Epstein–Barr virus nuclear antigen 1-dependent mechanism. Oncol Rep.

[849] Puthdee N, Seubwai W, Vaeteewoottacharn K, Boonmars T, Cha’on U, Phoomak C (2017). Berberine induces cell cycle arrest in cholangiocarcinoma cell lines via inhibition of NF-kappaB and STAT3 pathways. Biol Pharm Bull.

[850] Raghav D, Ashraf SM, Mohan L, Rathinasamy K (2017). Berberine induces toxicity in HeLa cells through perturbation of microtubule polymerization by binding to tubulin at a unique site. Biochemistry.

[851] Barzegar E, Fouladdel S, Movahhed TK, Atashpour S, Ghahremani MH, Ostad SN (2015). Effects of berberine on proliferation, cell cycle distribution and apoptosis of human breast cancer T47D and MCF7 cell lines. Iran J Basic Med Sci.

[852] Li J, Cao B, Liu X, Fu X, Xiong Z, Chen L (2011). Berberine suppresses androgen receptor signaling in prostate cancer. Mol Cancer Ther.

[853] Qi HW, Xin LY, Xu X, Ji XX, Fan LH (2014). Epithelial-to-mesenchymal transition markers to predict response of Berberine in suppressing lung cancer invasion and metastasis. J Transl Med.

[854] Kim S, Han J, Lee SK, Choi MY, Kim J, Lee J (2012). Berberine suppresses the TPA-induced MMP-1 and MMP-9 expressions through the inhibition of PKC-alpha in breast cancer cells. J Surg Res.

[855] Zhang XZ, Wang L, Liu DW, Tang GY, Zhang HY (2014). Synergistic inhibitory effect of berberine and d-limonene on human gastric carcinoma cell line MGC803. J Med Food.

[856] Yip NK, Ho WS (2013). Berberine induces apoptosis via the mitochondrial pathway in liver cancer cells. Oncol Rep.

[857] Yi T, Zhuang L, Song G, Zhang B, Li G, Hu T (2015). Akt signaling is associated with the berberine-induced apoptosis of human gastric cancer cells. Nutr Cancer.

[858] Kuo H-P, Lee Y-J, Hsu C-Y, Lee S-L, Hsu S-C, Chuang T-C (2015). Growth-suppressive effect of berberine on endometrial carcinoma cells: role of mitochondrial and PI3K/Akt pathway. J Funct Foods..

[859] Wen C, Wu L, Fu L, Zhang X, Zhou H (2016). Berberine enhances the antitumor activity of tamoxifen in drugsensitive MCF7 and drugresistant MCF7/TAM cells. Mol Med Rep..

[860] Wang N, Wang X, Tan HY, Li S, Tsang CM, Tsao SW (2016). Berberine suppresses cyclin D1 expression through proteasomal degradation in human hepatoma cells. Int J Mol Sci..

[861] Liu Q, Xu X, Zhao M, Wei Z, Li X, Zhang X (2015). Berberine induces senescence of human glioblastoma cells by downregulating the EGFR-MEK-ERK signaling pathway. Mol Cancer Ther.

[862] Yang X, Yang B, Cai J, Zhang C, Zhang Q, Xu L (2013). Berberine enhances radiosensitivity of esophageal squamous cancer by targeting HIF-1alpha in vitro and in vivo. Cancer Biol Ther.

[863] Hu Q, Li L, Zou X, Xu L, Yi P (2018). Berberine attenuated proliferation, invasion and migration by targeting the AMPK/HNF4alpha/WNT5A pathway in gastric carcinoma. Front Pharmacol.

[864] Seo YS, Yim MJ, Kim BH, Kang KR, Lee SY, Oh JS (2015). Berberine-induced anticancer activities in FaDu head and neck squamous cell carcinoma cells. Oncol Rep.

[865] Wang J, Yang S, Cai X, Dong J, Chen Z, Wang R (2016). Berberine inhibits EGFR signaling and enhances the antitumor effects of EGFR inhibitors in gastric cancer. Oncotarget..

[866] Zhao Y, Jing Z, Lv J, Zhang Z, Lin J, Cao X (2017). Berberine activates caspase-9/cytochrome c-mediated apoptosis to suppress triple-negative breast cancer cells in vitro and in vivo. Biomed Pharmacother.

[867] Yu R, Zhang ZQ, Wang B, Jiang HX, Cheng L, Shen LM (2014). Berberine-induced apoptotic and autophagic death of HepG2 cells requires AMPK activation. Cancer Cell Int.

[868] Li DX, Zhang J, Zhang Y, Zhao PW, Yang LM (2015). Inhibitory effect of berberine on human skin squamous cell carcinoma A431 cells. Genet Mol Res.

[869] Liu X, Ji Q, Ye N, Sui H, Zhou L, Zhu H (2015). Berberine inhibits invasion and metastasis of colorectal cancer cells via COX-2/PGE2 mediated JAK2/STAT3 signaling pathway. PLoS ONE.

[870] Saxena S, Shukla S, Kakkar P (2018). Berberine induced modulation of PHLPP2-Akt-MST1 kinase signaling is coupled with mitochondrial impairment and hepatoma cell death. Toxicol Appl Pharmacol.

[871] Li X, Gu S, Sun D, Dai H, Chen H, Zhang Z (2018). The selectivity of artemisinin-based drugs on human lung normal and cancer cells. Environ Toxicol Pharmacol.

[872] Chen J, Zhang L, Hao M (2018). Effect of artemisinin on proliferation and apoptosis-related protein expression in vivo and in vitro. Saudi J Biol Sci.

[873] Tran KQ, Tin AS, Firestone GL (2014). Artemisinin triggers a G1 cell cycle arrest of human Ishikawa endometrial cancer cells and inhibits cyclin-dependent kinase-4 promoter activity and expression by disrupting nuclear factor-kappaB transcriptional signaling. Anticancer Drugs.

[874] Suberu JO, Romero-Canelon I, Sullivan N, Lapkin AA, Barker GC (2014). Comparative cytotoxicity of artemisinin and cisplatin and their interactions with chlorogenic acids in MCF7 breast cancer cells. ChemMedChem.

[875] Gao W, Xiao F, Wang X, Chen T (2013). Artemisinin induces A549 cell apoptosis dominantly via a reactive oxygen species-mediated amplification activation loop among caspase-9, -8 and -3. Apoptosis.

[876] Yang Y, Zhang X, Wang X, Zhao X, Ren T, Wang F (2014). Enhanced delivery of artemisinin and its analogues to cancer cells by their adducts with human serum transferrin. Int J Pharm.

[877] Chen G, Gong R, Shi X, Yang D, Zhang G, Lu A (2016). Halofuginone and artemisinin synergistically arrest cancer cells at the G1/G0 phase by upregulating p21Cip1 and p27Kip1. Oncotarget..

[878] Ganguli A, Choudhury D, Datta S, Bhattacharya S, Chakrabarti G (2014). Inhibition of autophagy by chloroquine potentiates synergistically anti-cancer property of artemisinin by promoting ROS dependent apoptosis. Biochimie..

[879] Zhu S, Liu W, Ke X, Li J, Hu R, Cui H (2014). Artemisinin reduces cell proliferation and induces apoptosis in neuroblastoma. Oncol Rep.

[880] Weifeng T, Feng S, Xiangji L, Changqing S, Zhiquan Q, Huazhong Z (2011). Artemisinin inhibits in vitro and in vivo invasion and metastasis of human hepatocellular carcinoma cells. Phytomedicine.

[881] Deng XR, Liu ZX, Liu F, Pan L, Yu HP, Jiang JP (2013). Holotransferrin enhances selective anticancer activity of artemisinin against human hepatocellular carcinoma cells. J Huazhong Univ Sci Technol Med Sci.

[882] Jia J, Qin Y, Zhang L, Guo C, Wang Y, Yue X (2016). Artemisinin inhibits gallbladder cancer cell lines through triggering cell cycle arrest and apoptosis. Mol Med Rep..

[883] Xiao F, Gao W, Wang X, Chen T (2012). Amplification activation loop between caspase-8 and -9 dominates artemisinin-induced apoptosis of ASTC-a-1 cells. Apoptosis.

[884] Sin S, Kim SY, Kim SS (2012). Chronic treatment with ginsenoside Rg3 induces Akt-dependent senescence in human glioma cells. Int J Oncol.

[885] Wu K, Li N, Sun H, Xu T, Jin F, Nie J (2015). Endoplasmic reticulum stress activation mediates Ginseng Rg3-induced anti-gallbladder cancer cell activity. Biochem Biophys Res Commun.

[886] Li J, Liu T, Zhao L, Chen W, Hou H, Ye Z (2015). Ginsenoside 20(S)Rg3 inhibits the Warburg effect through STAT3 pathways in ovarian cancer cells. Int J Oncol.

[887] Zhou Y, Zheng X, Lu J, Chen W, Li X, Zhao L (2018). Ginsenoside 20(S)-Rg3 inhibits the warburg effect via modulating DNMT3A/MiR-532-3p/HK2 pathway in ovarian cancer cells. Cell Physiol Biochem.

[888] Liu T, Zhao L, Zhang Y, Chen W, Liu D, Hou H (2014). Ginsenoside 20(S)-Rg3 targets HIF-1alpha to block hypoxia-induced epithelial–mesenchymal transition in ovarian cancer cells. PLoS ONE.

[889] Ge X, Zhen F, Yang B, Yang X, Cai J, Zhang C (2014). Ginsenoside Rg3 enhances radiosensitization of hypoxic oesophageal cancer cell lines through vascular endothelial growth factor and hypoxia inducible factor 1alpha. J Int Med Res.

[890] Luo Y, Zhang P, Zeng HQ, Lou SF, Wang DX (2015). Ginsenoside Rg3 induces apoptosis in human multiple myeloma cells via the activation of Bcl-2-associated X protein. Mol Med Rep..

[891] Choi YJ, Lee HJ, Kang DW, Han IH, Choi BK, Cho WH (2013). Ginsenoside Rg3 induces apoptosis in the U87MG human glioblastoma cell line through the MEK signaling pathway and reactive oxygen species. Oncol Rep.

[892] Aziz F, Wang X, Liu J, Yan Q (2016). Ginsenoside Rg3 induces FUT4-mediated apoptosis in *H. pylori* CagA-treated gastric cancer cells by regulating SP1 and HSF1 expressions. Toxicol In Vitro..

[893] Guo JQ, Zheng QH, Chen H, Chen L, Xu JB, Chen MY (2014). Ginsenoside Rg3 inhibition of vasculogenic mimicry in pancreatic cancer through downregulation of VEcadherin/EphA2/MMP9/MMP2 expression. Int J Oncol.

[894] He BC, Gao JL, Luo X, Luo J, Shen J, Wang L (2011). Ginsenoside Rg3 inhibits colorectal tumor growth through the down-regulation of Wnt/ss-catenin signaling. Int J Oncol.

[895] Liu T, Duo L, Duan P (2018). Ginsenoside Rg3 sensitizes colorectal cancer to radiotherapy through downregulation of proliferative and angiogenic biomarkers. Evid Based Complement Altern Med..

[896] Wang L, Li X, Song YM, Wang B, Zhang FR, Yang R (2015). Ginsenoside Rg3 sensitizes human non-small cell lung cancer cells to gamma-radiation by targeting the nuclear factor-kappaB pathway. Mol Med Rep..

[897] Shan X, Aziz F, Tian LL, Wang XQ, Yan Q, Liu JW (2015). Ginsenoside Rg3-induced EGFR/MAPK pathway deactivation inhibits melanoma cell proliferation by decreasing FUT4/LeY expression. Int J Oncol.

[898] Xia T, Wang YN, Zhou CX, Wu LM, Liu Y, Zeng QH (2017). Ginsenoside Rh2 and Rg3 inhibit cell proliferation and induce apoptosis by increasing mitochondrial reactive oxygen species in human leukemia Jurkat cells. Mol Med Rep..

[899] Yu Y, Zhang C, Liu L, Li X (2013). Hepatic arterial administration of ginsenoside Rg3 and transcatheter arterial embolization for the treatment of VX2 liver carcinomas. Exp Ther Med.

[900] Joo EJ, Chun J, Ha YW, Ko HJ, Xu MY, Kim YS (2015). Novel roles of ginsenoside Rg3 in apoptosis through downregulation of epidermal growth factor receptor. Chem Biol Interact.

[901] Cho J, Rho O, Junco J, Carbajal S, Siegel D, Slaga TJ (2015). Effect of combined treatment with ursolic acid and resveratrol on skin tumor promotion by 12-O-tetradecanoylphorbol-13-acetate. Cancer Prevent Res (Philadelphia, Pa)..

[902] Gao N, Cheng S, Budhraja A, Gao Z, Chen J, Liu EH (2012). Ursolic acid induces apoptosis in human leukaemia cells and exhibits anti-leukaemic activity in nude mice through the PKB pathway. Br J Pharmacol.

[903] Lin CC, Huang CY, Mong MC, Chan CY, Yin MC (2011). Antiangiogenic potential of three triterpenic acids in human liver cancer cells. J Agric Food Chem.

[904] Song YH, Jeong SJ, Kwon HY, Kim B, Kim SH, Yoo DY (2012). Ursolic acid from *Oldenlandia diffusa* induces apoptosis via activation of caspases and phosphorylation of glycogen synthase kinase 3 beta in SK-OV-3 ovarian cancer cells. Biol Pharm Bull.

[905] Wu B, Wang X, Chi ZF, Hu R, Zhang R, Yang W (2012). Ursolic acid-induced apoptosis in K562 cells involving upregulation of PTEN gene expression and inactivation of the PI3K/Akt pathway. Arch Pharmacal Res.

[906] Zhang Y, Huang L, Shi H, Chen H, Tao J, Shen R (2018). Ursolic acid enhances the therapeutic effects of oxaliplatin in colorectal cancer by inhibition of drug resistance. Cancer Sci.

[907] Zheng QY, Jin FS, Yao C, Zhang T, Zhang GH, Ai X (2012). Ursolic acid-induced AMP-activated protein kinase (AMPK) activation contributes to growth inhibition and apoptosis in human bladder cancer T24 cells. Biochem Biophys Res Commun.

[908] Hagelgans A, Nacke B, Zamaraeva M, Siegert G, Menschikowski M (2014). Silibinin down-regulates expression of secreted phospholipase A2 enzymes in cancer cells. Anticancer Res.

[909] Kumar S, Raina K, Agarwal C, Agarwal R (2014). Silibinin strongly inhibits the growth kinetics of colon cancer stem cell-enriched spheroids by modulating interleukin 4/6-mediated survival signals. Oncotarget..

[910] Kauntz H, Bousserouel S, Gosse F, Marescaux J, Raul F (2012). Silibinin, a natural flavonoid, modulates the early expression of chemoprevention biomarkers in a preclinical model of colon carcinogenesis. Int J Oncol.

[911] Subramaniam A, Shanmugam MK, Ong TH, Li F, Perumal E, Chen L (2013). Emodin inhibits growth and induces apoptosis in an orthotopic hepatocellular carcinoma model by blocking activation of STAT3. Br J Pharmacol.

[912] Cheng X, Shi W, Zhao C, Zhang D, Liang P, Wang G (2016). Triptolide sensitizes human breast cancer cells to tumor necrosis factoralphainduced apoptosis by inhibiting activation of the nuclear factorkappaB pathway. Mol Med Rep..

[913] Jiang C, Fang X, Zhang H, Wang X, Li M, Jiang W (2017). Triptolide inhibits the growth of osteosarcoma by regulating microRNA-181a via targeting PTEN gene in vivo and vitro. Tumour Biol.

[914] Li W, Liu BD, Liao K, Liu Y, Wan ZJ, Dong YF (2018). Alteration of androgen receptor protein stability by triptolide in LNCaP Cells. Medicina (Kaunas, Lithuania)..

[915] Meng G, Wang W, Chai K, Yang S, Li F, Jiang K (2015). Combination treatment with triptolide and hydroxycamptothecin synergistically enhances apoptosis in A549 lung adenocarcinoma cells through PP2A-regulated ERK, p38 MAPKs and Akt signaling pathways. Int J Oncol.

[916] Qiao Z, He M, He MU, Li W, Wang X, Wang Y (2016). Synergistic antitumor activity of gemcitabine combined with triptolide in pancreatic cancer cells. Oncol Lett.

[917] Sangwan V, Banerjee S, Jensen KM, Chen Z, Chugh R, Dudeja V (2015). Primary and liver metastasis-derived cell lines from KrasG12D; Trp53R172H; Pdx-1 Cre animals undergo apoptosis in response to triptolide. Pancreas.

[918] Tamgue O, Lei M (2017). Triptolide promotes senescence of prostate cancer cells through histone methylation and heterochromatin formation. Asian Pac J Cancer Prevent.

[919] Wen L, Chen Y, Zeng LL, Zhao F, Li R, Liu Y (2012). Triptolide induces cell-cycle arrest and apoptosis of human multiple myeloma cells in vitro via altering expression of histone demethylase LSD1 and JMJD2B. Acta Pharmacol Sin.

[920] Xie CQ, Zhou P, Zuo J, Li X, Chen Y, Chen JW (2016). Triptolide exerts pro-apoptotic and cell cycle arrest activity on drug-resistant human lung cancer A549/Taxol cells via modulation of MAPK and PI3K/Akt signaling pathways. Oncol Lett.

[921] Yan X, Ke XX, Zhao H, Huang M, Hu R, Cui H (2015). Triptolide inhibits cell proliferation and tumorigenicity of human neuroblastoma cells. Mol Med Rep..

[922] Yang Y, Zhang LJ, Bai XG, Xu HJ, Jin ZL, Ding M (2018). Synergistic antitumour effects of triptolide plus gemcitabine in bladder cancer. Biomed Pharmacother.

[923] Zhao X, Zhang Q, Chen L (2016). Triptolide induces the cell apoptosis of osteosarcoma cells through the TRAIL pathway. Oncol Rep.

[924] Pandey P, Sayyed U, Tiwari RK, Siddiqui MH, Pathak N, Bajpai P (2019). Hesperidin induces ROS-Mediated apoptosis along with cell cycle arrest at G2/M phase in human gall bladder carcinoma. Nutr Cancer.

[925] Zhu J, Wang H, Chen F, Lv H, Xu Z, Fu J (2018). Triptolide enhances chemotherapeutic efficacy of antitumor drugs in non-small-cell lung cancer cells by inhibiting Nrf2-ARE activity. Toxicol Appl Pharmacol.

[926] Cai F, Zhang L, Xiao X, Duan C, Huang Q, Fan C (2016). Cucurbitacin B reverses multidrug resistance by targeting CIP2A to reactivate protein phosphatase 2A in MCF-7/adriamycin cells. Oncol Rep.

[927] Dakeng S, Duangmano S, Jiratchariyakul W, Bogler O, Patmasiriwat P (2012). Inhibition of Wnt signaling by cucurbitacin B in breast cancer cells: reduction of Wnt-associated proteins and reduced translocation of galectin-3-mediated beta-catenin to the nucleus. J Cell Biochem..

[928] Duangmano S, Sae-Lim P, Suksamrarn A, Domann FE, Patmasiriwat P (2012). Cucurbitacin B inhibits human breast cancer cell proliferation through disruption of microtubule polymerization and nucleophosmin/B23 translocation. BMC Complement Altern Med.

[929] Guo J, Wu G, Bao J, Hao W, Lu J, Chen X (2014). Cucurbitacin B induced ATM-mediated DNA damage causes G2/M cell cycle arrest in a ROS-dependent manner. PLoS ONE.

[930] Gupta P, Srivastava SK (2014). Inhibition of Integrin-HER2 signaling by Cucurbitacin B leads to in vitro and in vivo breast tumor growth suppression. Oncotarget..

[931] Promkan M, Dakeng S, Chakrabarty S, Bogler O, Patmasiriwat P (2013). The effectiveness of cucurbitacin B in BRCA1 defective breast cancer cells. PLoS ONE.

[932] Chen GY, Shu YC, Chuang DY, Wang YC (2016). Inflammatory and apoptotic regulatory activity of tanshinone IIA in *Helicobacter pylori*-infected cells. Am J Chin Med.

[933] Huang ST, Huang CC, Huang WL, Lin TK, Liao PL, Wang PW (2017). Tanshinone IIA induces intrinsic apoptosis in osteosarcoma cells both in vivo and in vitro associated with mitochondrial dysfunction. Sci Rep..

[934] Ding L, Wang S, Qu X, Wang J (2016). Tanshinone IIA sensitizes oral squamous cell carcinoma to radiation due to an enhanced autophagy. Environ Toxicol Pharmacol.

[935] Zhang Y, Li S, He H, Han Q, Wang B, Zhu Y (2017). Influence of Tanshinone IIA on apoptosis of human esophageal carcinoma Eca-109 cells and its molecular mechanism. Thorac Cancer..

[936] Ji ZHE, Tang Q, Zhang J, Yang YAN, Liu Y, Pan Y (2011). Oridonin-induced apoptosis in SW620 human colorectal adenocarcinoma cells. Oncol Lett.

[937] Jiang P, Jin H, Jiang J, Yang F, Cai H, Yang P (2017). Single molecule force spectroscopy for in situ probing oridonin inhibited ROS-mediated EGF-EGFR interactions in living KYSE-150 cells. Pharmacol Res..

[938] Kwan HY, Yang Z, Fong WF, Hu YM, Yu ZL, Hsiao WL (2013). The anticancer effect of oridonin is mediated by fatty acid synthase suppression in human colorectal cancer cells. J Gastroenterol.

[939] Li X, Li X, Wang J, Ye Z, Li JC (2012). Oridonin up-regulates expression of P21 and induces autophagy and apoptosis in human prostate cancer cells. Int J Biol Sci.

[940] Liu JB, Yue JY (2014). Preliminary study on the mechanism of oridonin-induced apoptosis in human squamous cell oesophageal carcinoma cell line EC9706. J Int Med Res.

[941] Ming M, Sun FY, Zhang WT, Liu JK (2016). Therapeutic effect of oridonin on mice with prostate cancer. Asian Pac J Trop Med..

[942] Shi M, Ren X, Wang X, Wang H, Liu G, Yuan X (2016). A novel combination of oridonin and valproic acid in enhancement of apoptosis induction of HL-60 leukemia cells. Int J Oncol.

[943] Weng H, Huang H, Dong B, Zhao P, Zhou H, Qu L (2014). Inhibition of miR-17 and miR-20a by oridonin triggers apoptosis and reverses chemoresistance by derepressing BIM-S. Cancer Res.

[944] Yang I-H, Shin J-A, Lee K-E, Kim J, Cho N-P, Cho S-D (2017). Oridonin induces apoptosis in human oral cancer cells via phosphorylation of histone H2AX. Eur J Oral Sci.

[945] Zheng M, Zhu Z, Zhao Y, Yao D, Wu M, Sun G (2017). Oridonin promotes G2/M arrest in A549 cells by facilitating ATM activation. Mol Med Rep..

[946] Ye LH, Li WJ, Jiang XQ, Chen YL, Tao SX, Qian WL (2012). Study on the autophagy of prostate cancer PC-3 cells induced by oridonin. Anatomical Record (Hoboken, NJ: 2007)..

[947] Han X, Kang KA, Piao MJ, Zhen AX, Hyun YJ, Kim HM (2019). Shikonin exerts cytotoxic effects in human colon cancers by inducing apoptotic cell death via the endoplasmic reticulum and mitochondria-mediated pathways. Biomol Ther..

[948] Liang W, Cai A, Chen G, Xi H, Wu X, Cui J (2016). Shikonin induces mitochondria-mediated apoptosis and enhances chemotherapeutic sensitivity of gastric cancer through reactive oxygen species. Sci Rep..

[949] Jeung YJ, Kim HG, Ahn J, Lee HJ, Lee SB, Won M (2016). Shikonin induces apoptosis of lung cancer cells via activation of FOXO3a/EGR1/SIRT1 signaling antagonized by p300. Biochem Biophys Acta.

[950] Jang SY, Hong D, Jeong SY, Kim JH (2015). Shikonin causes apoptosis by up-regulating p73 and down-regulating ICBP90 in human cancer cells. Biochem Biophys Res Commun.

[951] Wiench B, Eichhorn T, Paulsen M, Efferth T (2012). Shikonin directly targets mitochondria and causes mitochondrial dysfunction in cancer cells. Evid Based Complement Altern Med.

[952] Chen J, Xie J, Jiang Z, Wang B, Wang Y, Hu X (2011). Shikonin and its analogs inhibit cancer cell glycolysis by targeting tumor pyruvate kinase-M2. Oncogene.

[953] Lee PN, Ho WS (2013). Antiproliferative activity of gambogic acid isolated from *Garcinia hanburyi* in Hep3B and Huh7 cancer cells. Oncol Rep.

[954] Zhang H, Lei Y, Yuan P, Li L, Luo C, Gao R (2014). ROS-mediated autophagy induced by dysregulation of lipid metabolism plays a protective role in colorectal cancer cells treated with gambogic acid. PLoS ONE.

[955] Wei J, Yang P, Li W, He F, Zeng S, Zhang T (2017). Gambogic acid potentiates the chemosensitivity of colorectal cancer cells to 5-fluorouracil by inhibiting proliferation and inducing apoptosis. Exp Ther Med.

[956] Gao G, Bian Y, Qian H, Yang M, Hu J, Li L (2018). Gambogic acid regulates the migration and invasion of colorectal cancer via microRNA-21-mediated activation of phosphatase and tensin homolog. Exp Ther Med.

[957] Pan H, Lu LY, Wang XQ, Li BX, Kelly K, Lin HS (2018). Gambogic acid induces cell apoptosis and inhibits MAPK pathway in PTEN(−/−)/p53(−/−) prostate cancer cells in vitro and ex vivo. Chin J Integr Med.

[958] Wang Q, Wei J, Wang C, Zhang T, Huang D, Wei F (2018). Gambogic acid reverses oxaliplatin resistance in colorectal cancer by increasing intracellular platinum levels. Oncol Lett.

[959] Youns M, ElKhoely A, Kamel R (2018). The growth inhibitory effect of gambogic acid on pancreatic cancer cells. Naunyn-Schmiedeberg’s Arch Pharmacol.

[960] Zhu M, Wang M, Jiang Y, Wu H, Lu G, Shi W (2018). Gambogic acid induces apoptosis of non-small cell lung cancer (NSCLC) cells by suppressing notch signaling. Med Sci Monit.

[961] Zhao K, Zhang S, Song X, Yao Y, Zhou Y, You Q (2017). Gambogic acid suppresses cancer invasion and migration by inhibiting TGFbeta1-induced epithelial-to-mesenchymal transition. Oncotarget..

[962] Wang S, Shao M, Zhong Z, Wang A, Cao J, Lu Y (2017). Co-delivery of gambogic acid and TRAIL plasmid by hyaluronic acid grafted PEI-PLGA nanoparticles for the treatment of triple negative breast cancer. Drug Delivery.

[963] Tang Q, Lu M, Zhou H, Chen D, Liu L (2017). Gambogic acid inhibits the growth of ovarian cancer tumors by regulating p65 activity. Oncol Lett.

[964] Huang J, Zhu X, Wang H, Han S, Liu L, Xie Y (2017). Role of gambogic acid and NaI(131) in A549/DDP cells. Oncol Lett.

[965] Thida M, Kim DW, Tran TTT, Pham MQ, Lee H, Kim I (2016). Gambogic acid induces apoptotic cell death in T98G glioma cells. Bioorg Med Chem Lett.

[966] Wen C, Huang L, Chen J, Lin M, Li W, Lu B (2015). Gambogic acid inhibits growth, induces apoptosis, and overcomes drug resistance in human colorectal cancer cells. Int J Oncol.

[967] Xu N, Zhou X, Wang S, Xu LL, Zhou HS, Liu XL (2015). Artesunate induces SKM-1 cells apoptosis by inhibiting hyperactive beta-catenin signaling pathway. Int J Med Sci.

[968] Verma S, Das P, Kumar VL (2017). Chemoprevention by artesunate in a preclinical model of colorectal cancer involves down regulation of beta-catenin, suppression of angiogenesis, cellular proliferation and induction of apoptosis. Chem Biol Interact.

[969] Ilamathi M, Santhosh S, Sivaramakrishnan V (2016). Artesunate as an anti-cancer agent targets Stat-3 and favorably suppresses hepatocellular carcinoma. Curr Top Med Chem.

[970] Eling N, Reuter L, Hazin J, Hamacher-Brady A, Brady NR (2015). Identification of artesunate as a specific activator of ferroptosis in pancreatic cancer cells. Oncoscience..

[971] Raza A, Ghoshal A, Chockalingam S, Ghosh SS (2017). Connexin-43 enhances tumor suppressing activity of artesunate via gap junction-dependent as well as independent pathways in human breast cancer cells. Sci Rep..

[972] Go JH, Wei JD, Park JI, Ahn KS, Kim JH (2018). Wogonin suppresses the LPSenhanced invasiveness of MDAMB231 breast cancer cells by inhibiting the 5LO/BLT2 cascade. Int J Mol Med.

[973] Xu X, Zhang X, Zhang Y, Yang L, Liu Y, Huang S (2017). Wogonin reversed resistant human myelogenous leukemia cells via inhibiting Nrf2 signaling by Stat3/NF-kappaB inactivation. Sci Rep..

[974] Huynh DL, Kwon T, Zhang JJ, Sharma N, Gera M, Ghosh M (2017). Wogonin suppresses stem cell-like traits of CD133 positive osteosarcoma cell via inhibiting matrix metallopeptidase-9 expression. BMC Complement Altern Med.

[975] Huang B, Liu H, Huang D, Mao X, Hu X, Jiang C (2016). Apoptosis induction and imaging of cadmium-telluride quantum dots with wogonin in multidrug-resistant leukemia K562/A02 cell. J Nanosci Nanotechnol.

[976] Zhao K, Wei L, Hui H, Dai Q, You QD, Guo QL (2014). Wogonin suppresses melanoma cell B16-F10 invasion and migration by inhibiting Ras-medicated pathways. PLoS ONE.

[977] Wang Z, Li Y, Zhou F, Piao Z, Hao J (2018). beta-elemene enhances anticancer bone neoplasms efficacy of paclitaxel through regulation of GPR124 in bone neoplasms cells. Oncol Lett.

[978] Ding L, Zhang G, Hou Y, Chen J, Yin Y (2018). Elemene inhibits osteosarcoma growth by suppressing the reninangiotensin system signaling pathway. Mol Med Rep..

[979] Li P, Zhou X, Sun W, Sheng W, Tu Y, Yu Y (2017). Elemene induces apoptosis of human gastric cancer cell line BGC-823 via extracellular signal-regulated kinase (ERK) 1/2 signaling pathway. Med Sci Monit.

[980] Zhou J, He L-L, Ding X-F, Yuan Q-Q, Zhang J-X, Liu S-C (2016). Combinatorial antitumor effect of rapamycin and β-elemene in follicular thyroid cancer cells. Biomed Res Int.

[981] Zhao S, Wu J, Zheng F, Tang Q, Yang L, Li L (2015). beta-elemene inhibited expression of DNA methyltransferase 1 through activation of ERK1/2 and AMPKalpha signalling pathways in human lung cancer cells: the role of Sp1. J Cell Mol Med.

[982] Liu JS, Che XM, Chang S, Qiu GL, He SC, Fan L (2015). beta-elemene enhances the radiosensitivity of gastric cancer cells by inhibiting Pak1 activation. World J Gastroenterol.

[983] Zhang J, Zhang H, Chen L, Sun DW, Mao C, Chen W (2014). beta-elemene reverses chemoresistance of breast cancer via regulating MDR-related microRNA expression. Cell Physiol Biochem.

[984] Liu JS, He SC, Zhang ZL, Chen R, Fan L, Qiu GL (2014). Anticancer effects of beta-elemene in gastric cancer cells and its potential underlying proteins: a proteomic study. Oncol Rep.

[985] Guan C, Liu W, Yue Y, Jin H, Wang X, Wang XJ (2014). Inhibitory effect of beta-elemene on human breast cancer cells. Int J Clin Exp Pathol..

[986] Li QQ, Wang G, Liang H, Li JM, Huang F, Agarwal PK (2013). beta-Elemene promotes cisplatin-induced cell death in human bladder cancer and other carcinomas. Anticancer Res.

[987] Li QQ, Wang G, Huang F, Li JM, Cuff CF, Reed E (2013). Sensitization of lung cancer cells to cisplatin by beta-elemene is mediated through blockade of cell cycle progression: antitumor efficacies of beta-elemene and its synthetic analogs. Med Oncol (Northwood, London, England)..

[988] Shahriyar SA, Woo SM, Seo SU, Min KJ, Kwon TK (2018). Cepharanthine enhances TRAIL-mediated apoptosis through STAMBPL1-mediated downregulation of survivin expression in renal carcinoma cells. Int J Mol Sci..

